# Poly(*p*‐phenylene phosphaborene): A Modified Poly(*p*‐phenylene vinylene) with π‐Conjugated B═P Linkages

**DOI:** 10.1002/anie.202510272

**Published:** 2025-08-06

**Authors:** Julian Glock, Jonas Klopf, Merian Crumbach, Johannes Chorbacher, Johannes S. Schneider, Alexandra Friedrich, Emilia Buchsteiner, Tobias Bischof, Maik Finze, Bernd Engels, Holger Helten

**Affiliations:** ^1^ Institute of Inorganic Chemistry and Institute for Sustainable Chemistry & Catalysis with Boron (ICB), Julius‐Maximilians‐Universität Würzburg Am Hubland 97074 Würzburg Germany; ^2^ Rigaku Europe SE Hugenottenallee 167 63263 Neu‐Isenburg Germany; ^3^ Institute of Physical and Theoretical Chemistry Julius‐Maximilians‐Universität Würzburg Emil‐Fischer‐Str. 42 97074 Würzburg Germany

**Keywords:** BP compounds, Conjugated polymers, Hybrid polymers, Phosphinoboranes, Poly(*p*‐phenylene vinylene)

## Abstract

While the isoelectronic substitution of selected C═C by B═N units in π‐conjugated organic compounds has evolved into a well‐established concept to create novel hybrid materials with modified properties, the use of valence isoelectronic B═P units for such purposes has been hardly explored. Herein, we demonstrate substitutional doping of the famous optoelectronic organic polymer poly(*p*‐phenylene vinylene) (PPV) with B═P linking groups for the first time. We report on the successful synthesis of a soluble polymer and a series of monodisperse oligomers of BP‐PPV type. Our structural and photophysical investigations, supported by in‐depth DFT calculations, evidence effective π‐conjugation along their backbone, which is similary pronounced as in their previously reported BN congeners. In addition, the novel BCP compounds show distinct fluorescence emission in solution and emission enhancement upon aggregation (AIEE), occurring from a P‐pyramidalized excited state.

## Graphical abstract

An unprecedented poly(*p*‐phenylene phosphaborene) (BP‐PPV), derived from poly(*p*‐phenylene vinylene) (PPV) by substitution of the bridging C═C moieties by valence isoelectronic B═P units, has been prepared. The polymer and related monodisperse oligomers feature an effectively π‐conjugated backbone—involving the B═P bonds—and they show fluorescence from a pyramidalized excited state as well as aggregation‐induced emission enhancement (AIEE).

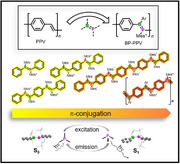

Conjugated polymers are today among the most extensively explored classes of organic materials due to their broad spectrum of important applications ranging from organic (opto)electronics to biomedicine.^[^ ^1^, ^2^ ^]^ Poly(*p*‐phenylene vinylene) (PPV) is one of the prototypes of conjugated polymers (Figure 1); and PPV and substituted derivatives thereof are of both fundamental and applied interest.^[^ ^3^ ^]^ In recent years, the inclusion of several main group elements beyond the standard repertoire of organic chemistry in organic electronic materials’ research has significantly increased.^[^ ^4^, ^5^, ^6^, ^7^, ^8^, ^9^, ^10^ ^]^ The use of phosphorus has been particularly fruitful in this regard.^[^ ^11^, ^12^, ^13^, ^14^, ^15^, ^16^, ^17^, ^18^, ^19^, ^20^, ^21^, ^22^, ^23^, ^24^, ^25^, ^26^, ^27^, ^28^ ^]^ For instance, Gates and Protasiewicz and their respective coworkers reported seminal works on unprecedented analogues of PPV wherein the vinylene bridges are replaced with P═C or P═P linkages, respectively (i.e., PC‐ and PP‐PPV).^[^ ^29^, ^30^, ^31^, ^32^, ^33^, ^34^, ^35^, ^36^ ^]^

**Figure 1:**
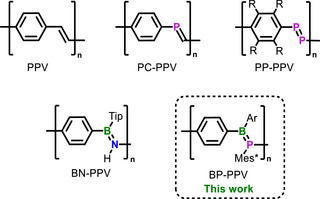
Poly(*p*‐phenylene vinylene) (PPV) and main group element modifications thereof.

In recent times, the deployment of trivalent boron as a dopant for π‐conjugated materials has attracted considerable research attention.^[^ ^4^, ^5^, ^6^, ^7^, ^8^, ^9^, ^10^, ^37^, ^38^, ^39^, ^40^, ^41^, ^42^, ^43^ ^]^ The combination of tricoordinate boron and nitrogen atoms gives a pair that is isoelectronic and isosteric to a C═C unit. By substitution of selected CC by BN units in conjugated organic compounds, new hybrid materials with modified, often intriguing properties can be obtained.^[^ ^44^, ^45^, ^46^, ^47^, ^48^, ^49^, ^50^, ^51^, ^52^, ^53^, ^54^ ^]^ We recently presented a novel BN‐bridged congener of PPV (i.e., BN‐PPV)^[^ ^55^, ^56^, ^57^ ^]^ and some further related BCN polymers and oligomers.^[^ ^58^, ^59^, ^60^, ^61^, ^62^, ^63^, ^64^, ^65^, ^66^, ^67^, ^68^, ^69^, ^70^, ^71^ ^]^ When tricoordinate boron is combined with σ^3^‐phosphorus, the resulting BP pair is valence isoelectronic to CC and BN.^[^ ^72^, ^73^, ^74^ ^]^ Compared with the use of B═N, substitutional doping of conjugated organic compounds with a B═P moiety has been significantly less extensively explored so far. However, the resulting BCP hybrid materials recently obtained offer promising potential in various fields of application,^[^ ^26^, ^75^, ^76^, ^77^, ^78^, ^79^, ^80^, ^81^, ^82^, ^83^, ^84^, ^85^, ^86^ ^]^ for example, as fluorescence dyes,^[^ ^75^, ^76^, ^77^ ^]^ building blocks for hybrid inorganic/organic conjugated materials,^[^ ^78^, ^79^, ^80^ ^]^ or the development of new synthetic procedures^[^ ^26^, ^82^, ^83^, ^84^, ^85^, ^86^ ^]^ (e.g., phosphaborenes as the first B═P transfer reagents^[^ ^81^, ^84^, ^85^ ^]^).

Herein, we present the first BP congener of PPV, namely, poly(*p*‐phenylene phosphaborene) (**P1**). Additionally, we prepared a series of monodisperse oligomers, i.e., differently substituted monomer **1** ^**Ar**^, two regioisomeric dimers **2** ^**a**^ and **2** ^**b**^, and tetramer **4**, providing valuable insights into the effects of chain elongation. We demonstrate that the attachment of the bulky supermesityl (Mes*) substituent to P effectively planarizes the phosphorus center and favors B–P π‐bonding. The B═P units participate in extended π‐conjugation along the BP‐PPV backbone, as evidenced by continuous redshifts in the absorption with increasing chain length, which is even more pronounced than in the BN‐congener BN‐PPV. The BP compounds reported here show distinct fluorescence emission, which occurs from a pyramidalized excited state.

While the coordination environment of trivalent phosphorus typically is trigonal‐pyramidal, direct bonding to a π‐acidic boron atom with its vacant p‐orbital induces (partial) planarization toward trigonal‐planar conformation at the phosphorus center.^[^ ^72^, ^73^, ^74^ ^]^ Sterically demanding substituents at P tend to further support this planarization.^[^ ^72^, ^73^, ^74^, ^87^ ^]^ For the “monomers” **1^Ar^**, we applied three different bulky aryl groups, i.e., mesityl (Mes), tri‐*iso*‐propylphenyl (tripyl, Tip), and tri‐*tert*‐butylphenyl (supermesityl, Mes*) at phosphorus; and we attached Mes at B to provide kinetic stabilization. We thus synthesized phosphinoboranes **1^Mes^**, **1^Tip^**, and 1^Mes*^ by metathesis reactions of bromoborane **3** with the respective potassium phosphides **5^Ar^** in the presence of TMEDA (Figure 2). We obtained the desired products in 53%–77% yield after crystallization, and we accomplished to determine their crystal structures by single‐crystal X‐ray diffraction (SXRD). In **1^Tip^**, the sum of the C─P─C/B bond angles of 347.4(1)° is intermediate between that for a trigonal‐planar and a pyramidal conformation. For **1^Mes^**, the position of the weakly scattering boron atom and, hence, the structural characteristics of the molecule could not reliably be derived due to whole molecule disorder via inversion symmetry. The atom position of the heavier, stronger scattering phosphorus atom, on the other hand, could be well determined. This center definitely exhibits an intermediate, nonplanar conformation, similarly to that in **1^Tip^**. Application of the bulkier Mes* substituent, finally, results in complete planarization of the phosphorus environment with the sum of the C─P─C/B angles being close to 360° in 1^Mes*^. Concomitantly, the B─P bond length shortens from 1.839(2) Å in **1^Tip^** to 1.814(2) Å in 1^Mes*^, indicative of significantly increased B═P double bond character.^[^ ^72^, ^73^, ^74^ ^]^ The B─P bond of 1^Mes*^ is also relatively short compared to other phosphinoboranes (e.g., Mes_2_B═PPh_2_, 1.859(3) Å;^[^ ^88^ ^]^ Mes_2_B═PMes_2_, 1.839(8) Å^[^ ^89^ ^]^).^[^ ^72^, ^73^, ^74^ ^]^ Furthermore, in 1^Mes*^ the >B═P< moiety and the phenyl substituents at B and P adopt a largely coplanar arrangement with a twist of 13.6(1)°, pointing to effective π‐conjugation over the Ph─B═P─Ph backbone of the molecule. Note for comparison the strong twist between the phenyl groups observed in **1^Tip^** (∠Ph,Ph = 75.5(1)°; Figure 2).

**Figure 2:**
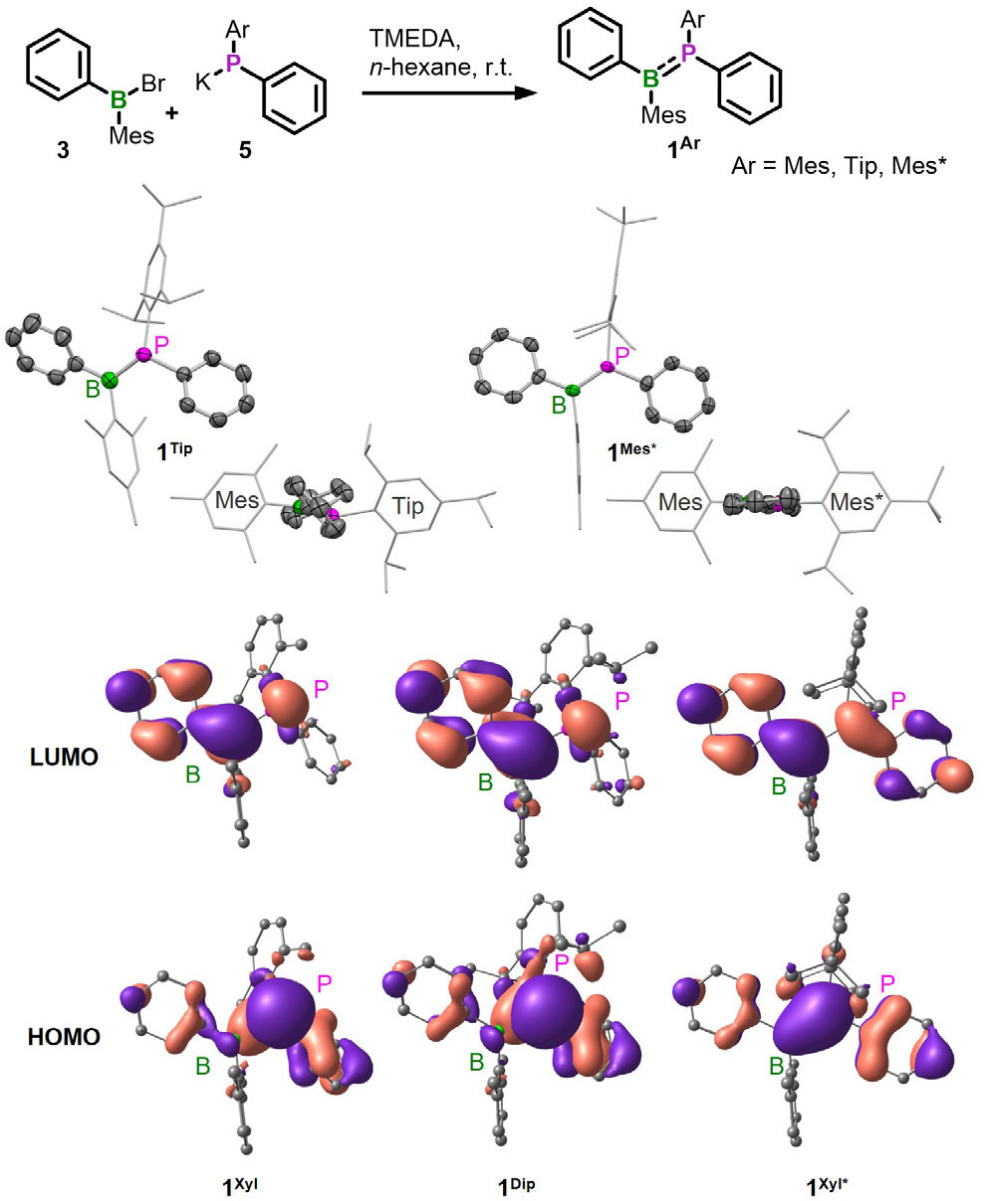
Top: Synthesis of phosphinoboranes **1^Mes^**, **1^Tip^**, and 1^Mes*^. Center: Solid‐state molecular structures of **1^Tip^** and 1^Mes*^ as determined by SXRD at 100 K. Hydrogen atoms are omitted for clarity. Bottom: Calculated frontier orbitals (isovalue 0.03 a.u.) of **1^Xyl^**, **1^Dip^**, and 1^Xyl*^ (ω_T_B97X‐D3/def2‐SVP, CPCM (THF), *ω*_T_ = 0.105).

To get deeper insight into the bonding situation, we performed DFT calculations on model systems for the three monomers where we omitted the *para*‐substituents at the *B*‐ and *P*‐aryl substituents for computational convenience, i.e., *m*‐xylyl (Xyl) instead of Mes, 2,6‐diisopropylphenyl (Dip) instead of Tip, and 2,6‐di‐*tert*‐butylphenyl (Xyl*) to mimic Mes*. Our calculations support complete planarization of the phosphorus center when the largest aryl substituent, Xyl*, is attached to P (Σ∡(RPR) = 360°), while a slightly pyramidal conformation of phosphorus (Σ∡(RPR) = 332°) is calculated for **1^Xyl^** and **1^Dip^**, which is in agreement with our experimental results. The calculated frontier orbitals indicate an increase in π‐bond character, consistent with a transition from a B─P single to a B═P double bond (B─P = 1.813 Å in 1^Xyl*^, Table S10) with donor(P)−acceptor(B)‐type π‐bonding with increasing steric bulk of the substituent (Figure 2). Whereas the HOMO of **1^Xyl^** still has significant phosphorus lone pair character—to some extent delocalized into the π‐system of the phenyl group attached to it—and the LUMO has the largest contribution from the boron's vacant p‐orbital and the phenyl group at this center, the frontier orbitals of 1^Xyl*^ both clearly show the characteristics of extended π‐orbitals delocalized over the entire Ph─B═P─Ph backbone. The HOMO of 1^Xyl*^ is mainly characterized as a B═P‐bonding π‐orbital, nearly equally shared between both atoms. Additional π‐type contributions from both phenyl substituents are clearly visible. The LUMO of 1^Xyl*^ is fully delocalized and antibonding with respect to the B═P bond, having a nodal plane between both heteroatoms. The characteristics of the frontier orbitals of **1^Dip^** are intermediate between those of **1^Xyl^** and 1^Xyl*^. The HOMO and the LUMO of **1^Xyl^**, **1^Dip^**, and 1^Xyl*^ are also involved in the excitation assigned to the major absorption band in their UV–vis spectra (vide infra). Therefore, the character of this transition changes from an n(P)→p(B)* process for **1^Xyl^** and **1^Dip^** to a π−π* transition for 1^Xyl*^ involving the entire molecular backbone.

These results encouraged us to consider Mes* as the P‐substituent for our further investigations into the preparation of conjugated oligomers and polymers. We prepared dimers **2^a^** and **2^b^**, which have the heteroatom sequences PBBP and BPPB along their backbone, respectively, as well as the tetramer **4** (Scheme 1). Unlike **2^a^**, we prepared **2^b^** in two steps, as this substantially increased the yield from 20% to 74%. Both dimers were obtained as bright‐yellow solids and **4** as a deep‐orange solid, all with conspicuous fluorescent behavior. The two isomers, **2^a^** and **2^b^**, exhibit pronounced differences in terms of their solubility. Compound **2^a^** dissolves well in THF and moderately in less‐polar solvents such as toluene, whereas **2^b^** is only in traces soluble in THF and virtually insoluble in all other common organic solvents. All oligomers **2^a^**, **2^b^**, **4**, as well as 1^Mes*^ proved to be long‐term inert toward air and moisture in the solid state. We tested the chemical stability of **2^a^** and **4** toward diluted acetic acid, phosphoric acid, and sulfuric acid in solution and did not detect any degradation within 1 day of exposure. We observed rapid degradation in hydrogen chloride as well as hydrogen bromide solutions. Furthermore, we tested the thermal stability of **2^a^** and **4** by thermogravimetric analysis (TGA). Compound **2^a^** showed thermal stability up to 222 °C (5% mass loss) with small mass changes starting at 87 °C and **4** up to 328 °C (5% mass loss) with a first onset at 129 °C (Figures S96 and S97).

**Scheme 1:**
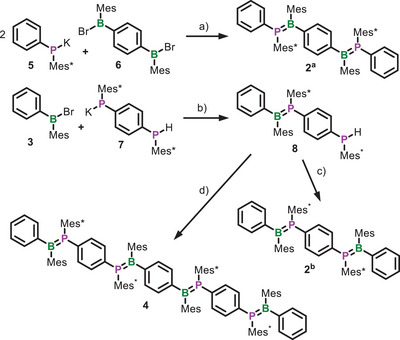
Synthesis of oligomers **2^a^**, **2^b^**, and **4**. a) 2 TMEDA, *n*‐hexane, r.t., 24 h; b) TMEDA, *n*‐hexane, r.t., 24 h; c) BzK, TMEDA, *n*‐hexane, r.t., 3 h, then **3**, r.t., 24 h; and d) 2 equiv **8**, 2 BzK, TMEDA, *n*‐hexane, r.t., 3 h, then **6**, r.t., 24 h.

We determined the crystal structure of **2^a^** by SXRD and those of **2^b^** and **4** using 3D electron diffraction (3D ED), respectively (Figure 3). Both dimers and the tetramer exhibit, like 1^Mes*^, a perfectly planar phosphorus environment with a sum of the C─P─C/B angles of 360° within the standard uncertainties (Table S3). Compound **2^a^** exhibits a similarly short B─P bond (1.811(2) Å) as 1^Mes*^. The B─P bond lengths of **2^b^** (1.83(2) Å) and **4** (1.840(13) and 1.848(12) Å) could not be determined with a high accuracy from the 3D ED data but are still in the range of the bond lengths observed for the other BP‐PPV oligomers pointing to double bond character.^[^ ^74^ ^]^ The planar phosphorus environment and short B═P double bonds were further supported by the computed ground‐state geometries of the truncated dimers (B─P = 1.812 Å and 1.813 Å for **2^a^′** and **2^b^′**, respectively, having *B*‐Xyl and *P*‐Xyl*, Table S10).

**Figure 3:**
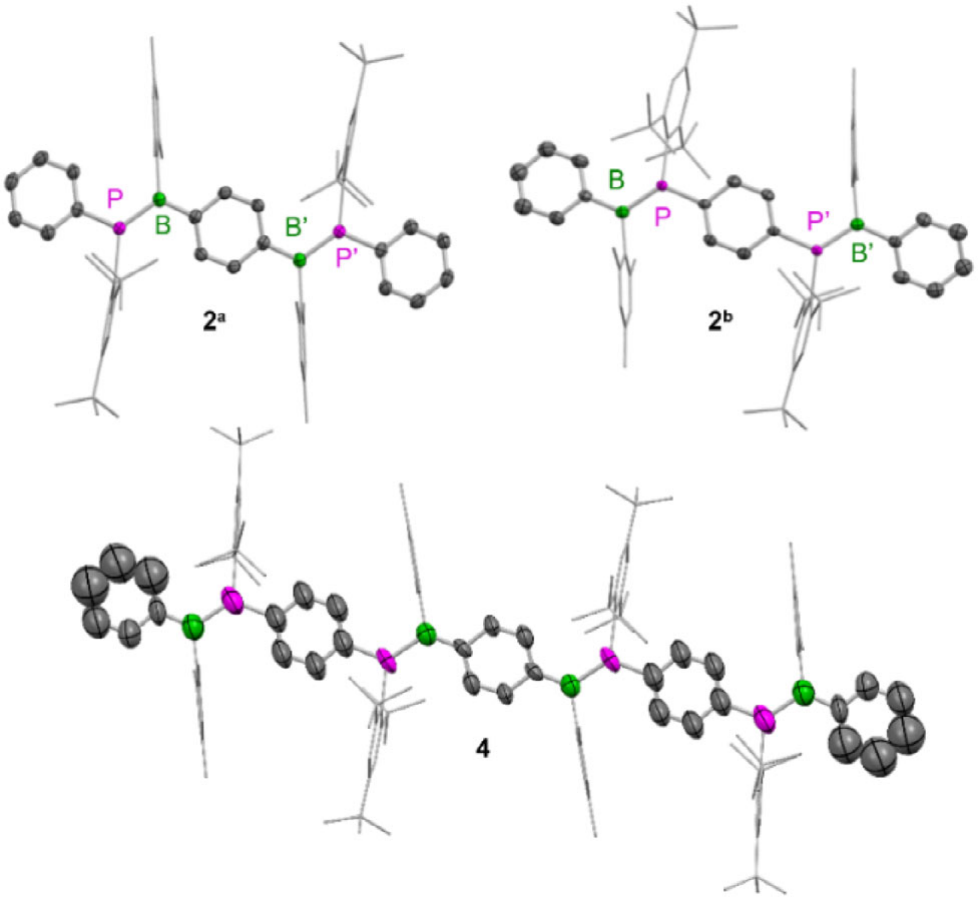
Solid‐state molecular structures of **2^a^**, **2^b^**, and **4** as determined by SXRD at 100 K (**2^a^**) and 3D ED at 175 K (**2^b^**, **4**). Hydrogen atoms are omitted for clarity. Element color code: carbon (grey), boron (green), and phosphorus (pink).

In the solid‐state structures, the flanking phenyl units are exactly coplanar via inversion symmetry in **2^a^** and **4** and close to coplanar in **2^b^** with a twist of 25.4(5)° between the planes. For **2^a^**, **2^b^**, and **4**, also the adjacent phenyl and phenylene rings as well as the >B═P< moieties along the backbone are nearly coplanar (Table S3). The Mes and Mes* groups in **2^a^**, **2^b^**, and **4** are largely perpendicular to the BR_3_ and PR_3_ planes with angles in the range of 81.9(1)−89.1(3)° between the planes, thus providing effective steric shielding of the boron atom and planarization of the phosphorus center, respectively. It is furthermore noteworthy that we observed only *trans* configuration for the phenyl/phenylene moieties across the B═P bonds in the backbones of **1**, **2^a^**, **2^b^**, and **4**.

In a first attempt to synthesize a BP‐PPV, we reacted **6** with equal amounts of dipotassium bisphosphide **9**. The product, however, proved to be virtually insoluble in all common solvents. Therefore, we applied solubility‐enhancing groups at the B‐substituents. By exchanging the Mes group at boron for 1,5‐dimethyl‐4‐octylphenyl, we accomplished to obtain **P1** as a soluble, deep orange polycondensation product (Scheme 2). The polymerization was terminated after 24 h by the addition of TMS‐NMe_2_ to deactivate eventually remaining reactive B−Br and P−K end groups. The recorded GPC (gel permeation chromatography) trace revealed an oligomeric to low polymeric product with molar mass averages of *M*_n_ = 3.07 and *M*_w_ = 6.65 kDa, respectively.

**Scheme 2:**
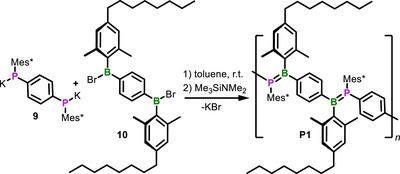
Synthesis of BP‐PPV **P1**.

The UV–vis spectra of all isolated compounds in THF display a low‐energy absorption band that undergoes a gradual bathochromic shift with increasing number of BP units and increasing chain length from the monomer 1^Mes*^ at *λ*_max_ = 374 nm to the polymer **P1** at *λ*_max_ = 490 nm (Figure 4 and Table 1). According to our TD‐DFT calculations, this band is assigned to the HOMO→LUMO excitation in each case, which is characterized as π–π* transition involving the whole BP‐PPV backbone. A certain degree of charge transfer is additionally attributed to this process, as the largest contribution to the respective HOMO comes from the phosphorus atoms and the arene rings they are attached to, while the LUMOs are more polarized toward the boron atoms and the B‐bound arene rings (Figure 5). Overall, these results demonstrate that BP‐PPV and the monodisperse oligomers of that type have an effectively π‐conjugated backbone involving the B═P moieties.

**Figure 4:**
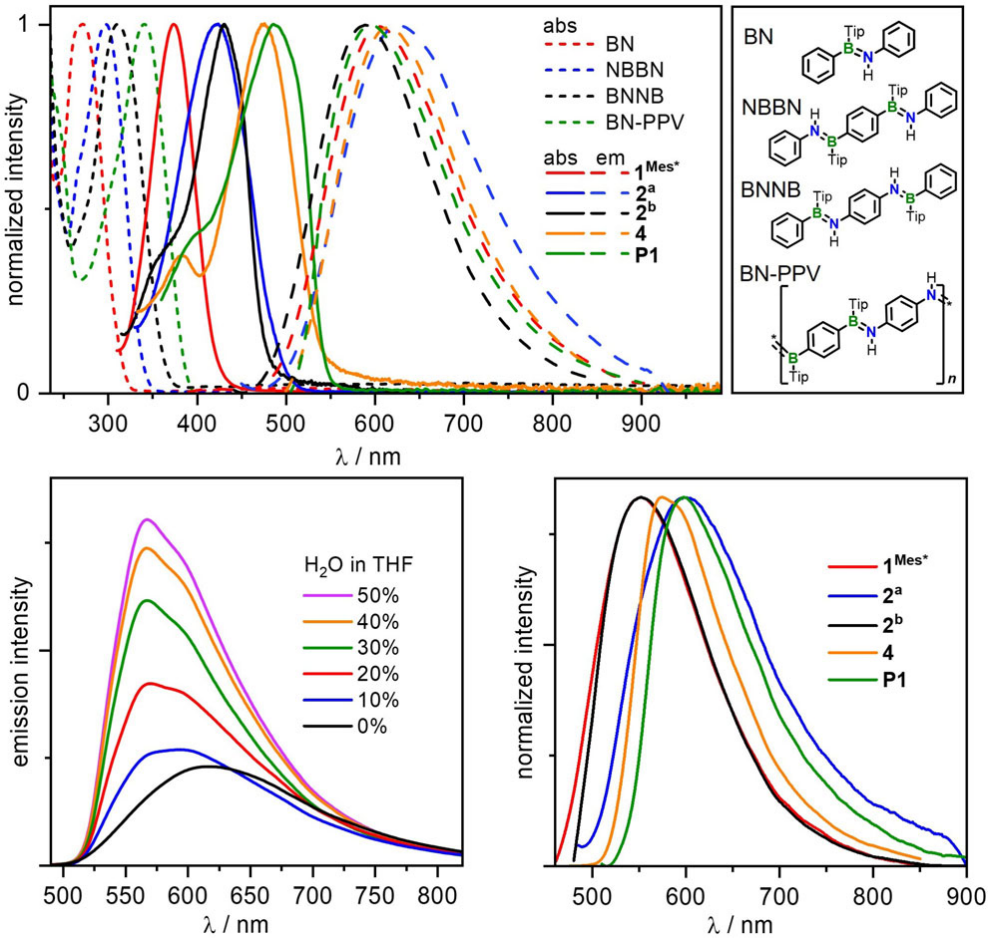
Top: Normalized UV–vis absorption and fluorescence emission spectra of 1^Mes*^, **2^a^**, **2^b^**, **4**, and **P1** compared to the UV–vis spectra of corresponding BN‐congeners in THF.^[^ ^55^, ^56^ ^]^ Bottom left: Emission spectra of **4** in different THF–water mixtures (conc. 5 × 10^−5^ M) with different water ratios (0%−50%). Bottom right: Solid‐state emission spectra of 1^Mes*^, **2^a^**, **2^b^**, **4**, and **P1**.

**Table 1:** UV–vis absorption and fluorescence emission data of **1^Mes^**, **1^Tip^**, 1^Mes*^, dimers **2^a^**, **2^b^**, tetramer **4**, and the polymerization product **P1** measured in THF.

Compd.	*λ*_abs_ ^a)^ (nm)	*λ*_em_ ^a)^ (nm)	*Φ*_fl_ ^b)^ (%) THF | solid state	Stokes shift^a)^ (cm^−1^)	*τ*_fl_ (ns)
**1** ^**Mes**^	368	–	–	–	–
**1** ^**Tip**^	372	544	2 | –	8499	<1
**1** ^Mes*^	374	603	6 | 21	10 154	<1
**2^a^**	423	628	14 | 9	7717	<1
**2^b^**	432	591	22 | 30	6228	2.1
**4**	474	620	10 | 27	4968	<1
**P1**	490	595	15 | 21	3601	<1

^a)^ In THF solution.

^b)^ Fluorescence quantum yield determined with an integration sphere.

**Figure 5:**
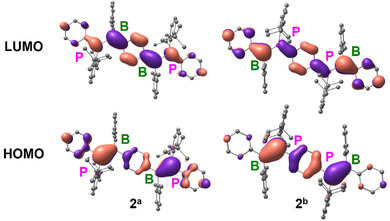
Calculated frontier orbitals (isovalue 0.03 a.u.) of **2^a^′** and **2^b^′** (*ω*_T_B97X‐D3/def2‐SVP, CPCM (THF), *ω*_T_ = 0.105).

The BN‐PPV and the BCN oligomers we reported recently^[^ ^55^, ^56^, ^57^ ^]^ likewise showed continuous redshifts in their absorption with chain elongation; however, interestingly, the absorption maxima of the BP compounds presented herein are even further bathochromic shifted—by about 100 nm—from those of their BN congeners (i.e., monomer, dimers, or polymer, respectively). This difference can be explained by the higher electronegativity of nitrogen compared to phosphorus and thus the higher HOMO→LUMO gap. The shift between the absorption maxima of **2^a^** and 1^Mes*^ (0.38 eV) is close to that of their BN congeners (dimer “NBBN” versus monomer “BN,” 0.40 eV). This indicates that π‐conjugation over the B═P bonds is similarly pronounced as over B═N moieties in such species.

The P‐Mes* substituted BP compounds (and very weakly also **1^Tip^**) show distinct fluorescence emission in solution with large Stokes shifts (Figure 4 and Table 1). This was not observed for their BN congeners having the analogous BBNN sequence, as those compounds were only emissive in the aggregated state.^[^ ^55^, ^56^ ^]^ The variant of BN‐PPV and the respective oligomers we reported recently, comprising BNBN sequence along the main chain, showed emission from either locally excited (LE) or twisted intramolecular charge transfer (TICT) states depending on the environment and external stimuli.^[^ ^57^ ^]^ The observed emission bands of the BP‐PPV oligomers, unlike their absorption bands, show no correlation between the size of the conjugated π‐system and the wavelength of the corresponding maximum.

The fluorescence lifetimes in solution are shorter than 1 ns for all BP‐PPV oligomers except for **2^b^**, which has a lifetime of 2.1 ns (Table 1). This agrees well with the fluorescence quantum yields, found to be highest for **2^b^**. Furthermore, all BP‐PPV oligomers and the polymer are emissive in the solid state (Figure 4, bottom right), where we observed a slight hypsochromic shift and, moreover, in most cases an increase in the fluorescence quantum yield compared to the respective experiment in THF solution. This points to an aggregation‐induced emission enhancement (AIEE) effect, which we confirmed by measuring the spectra in THF/water and THF/*n*‐pentane mixtures at varying ratios (Figures 4 (bottom left) and S66−S71) as well as dynamic light scattering (DLS) (Figure S98). In addition, we observed a distinct emission enhancement at low temperatures (Figures S80, S81) and phosphorescence of **4** below 100 K (Figures S82, S83). **P1** and **2^b^** exhibit a small mechanochromic effect upon grinding (Figures S77 and S79), which was also observed for BN‐PPV.

To better understand the emission properties of these compounds, we performed additional TD‐DFT computations, including optimizations of electronically excited states for the monomer 1^Xyl*^. The results are sketched in Figure 6. We found two minimum geometries for the first excited states (S_1_). The global one is a TICT state (TICT S_1_) where the molecule is twisted by about 90° about the B─P bond with respect to the S_0_ geometry. The relaxation toward this minimum is slightly hampered by a small barrier of less than 3 kcal mol^−1^. Emission from this state is predicted to be forbidden and to occur in the NIR region (e.g., 0.68 eV, 1820 nm for 1^Xyl*^). Additionally, for this geometry, S_1_ and T_1_ are nearly degenerate so that an S–T transition is very likely. Hence, if this minimum is reached, nonradiative decay is expected.

**Figure 6:**
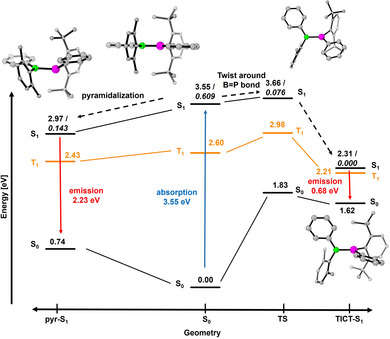
Geometrical and energetical change (in eV) for 1^Xyl*^ going from the S_0_ geometry to the pyr‐S_1_ geometry (left) or to the TICT‐S_1_ geometry with a barrier at TS geometry (right). The italic numbers are the oscillator strengths of the transitions.

Relaxation to the second minimum, denoted as pyr‐S_1_, is not hampered by any barrier. In pyr‐S_1_, the phosphorus center is pyramidalized. The sum of C─P─C/B angles is 346° compared to Σ_∠_ = 360° in the minimum S_0_ geometry. The predicted vertical de‐excitation from this minimum is 2.23 eV (557 nm), which is in good agreement with the experimentally measured emission maximum. Hence, we expect that the experimentally observed emission takes place from the pyr‐S_1_ minimum. The change in geometry is visualized in Figure 6, along with the corresponding energy levels for S_0_ and S_1_. In pyr‐S_1_, the phosphorus center is pyramidalized. The sum of C─P─C/B angles is 346° compared to Σ_∠_ = 360° in the minimum S_0_ geometry. The π‐bond is broken in pyr‐S_1_, as is indicated by the significantly increased B─P bond length of 2.044 Å (cf. S_0_: 1.813 Å). Inspection of the frontier orbitals reveals that pyr‐S_1_ corresponds to a charge‐separated state, where one electron is promoted into a B─Ph‐centered orbital, while the remaining electron resides in a more P─Ph‐centered orbital. The bonds to the adjacent phenyl groups are slightly shortened, on the other hand (B─C: from 1.574 to 1.527 Å, P─C: from 1.812 to 1.799 Å). In the pyr‐S_1_ states of the dimers **2^a^′** and **2^b^′** only one of the two phosphorus atoms is pyramidalized. Similarly, rotation about one of both B─P bonds has occurred in the TICT‐S_1_ states of these molecules. A detailed discussion of the photophysics of 1^Xyl*^ and additional information for **2^a^′** and **2^b^′** is given in the Supporting Information.

In this study, we demonstrated successful incorporation of the >B═P< moiety as a valence isoelectronic substitute for C═C into the structural motif of poly(*p*‐phenylene vinylene) for the first time. We achieved monodisperse oligomers and a soluble polymer with nearly perfectly planarized phosphorus centers, significant B═P double bond character, and a highly planar conjugated backbone. Their major UV–vis absorption bands are progressively redshifted with increasing chain length—indicative of extended π‐conjugation along their backbone—moreover, they are further redshifted compared to those of their previously reported BN analogues. In addition, the BP compounds show distinct fluorescence emission in solution, which is even enhanced in the solid state or, in general, upon aggregation. They emit from an excited state where the phosphorus center is pyramidalized. Furthermore, emission enhancement occurs at low temperatures accompanied by phosphorescence below 100 K. These characteristics further distinguish the novel BP‐PPV derivatives from their BN congeners.

## Supporting Information

The authors have cited additional references within the Supporting Information. Full structural information has been deposited with the Cambridge Crystallographic Data Centre. CCDC‐2440917 (**1^Mes^**), CCDC‐2440918 (**1^Tip^**), CCDC‐2440919 (1^Mes*^), CCDC‐2440920 (**2^a^**), CCDC‐2440921 (**2^b^**), CCDC‐2440922 (**4**), CCDC‐2440923 (**7H**), and CCDC‐2440924 (**8**). These data can be obtained free of charge from CCDC via www.ccdc.cam.ac.uk/data_request/cif.

## Conflict of Interests

The authors declare no conflict of interest.

## Supporting information



Supporting Information

Supporting Information

## Data Availability

The data that support the findings of this study are available in the Supporting Information of this article.
