# Supplementary material for: Poly(p‐phenylene phosphaborene): A Modified Poly(p‐phenylene vinylene) with π‐Conjugated B═P Linkages
Source: Angew Chem Int Ed Engl. 2025 Aug 6;64(38):e202510272. doi: 10.1002/anie.202510272 (PMC12435409; doi:10.1002/anie.202510272)
Supplement: Supplementary file 1 — Supporting Information [file ANIE-64-e202510272-s002.pdf]

Supporting Information

Poly(*p*-phenylene phosphaborene): A Modified Poly(*p*-phenylene vinylene) with π-Conjugated B=P Linkages

Julian Glock,[a] Jonas Klopf,[a] Merian Crumbach,[a] Johannes Chorbacher,[a] Johannes S. Schneider,[a] Alexandra Friedrich,[a] Emilia Buchsteiner,[b] Tobias Bischof,[a] Maik Finze,[a] Bernd Engels,[c] and Holger Helten*[a]

[a] *Julius-Maximilians-Universität Würzburg, Institute of Inorganic Chemistry and Institute of Sustainable Chemistry & Catalysis with Boron (ICB), Am Hubland, 97074 Würzburg, Germany.*

[b] *Rigaku Europe SE, Hugenottenallee 167, 63263 Neu-Isenburg, Germany.*

[c] *Julius-Maximilians-Universität Würzburg, Institute of Physical and Theoretical Chemistry, Emil-Fischer-Str. 42, 97074 Würzburg, Germany*

holger.helten@uni-wuerzburg.de

Table of Contents

1. Experimental Section 3

1.1. General Procedures 3

1.2. Syntheses 5

1.3. Crystallographic Data 11

1.4. NMR Spectra 21

1.5. High Resolution Mass Spectra 43

1.6. UV/Vis and Fluorescence Spectra 46

1.7. GPC-Traces 61

1.8. Cyclic Voltammetry 62

1.9. Thermogravimetric Analysis (TGA) 69

1.10. Dynamic light scattering (DLS) 70

2. Computational Information 71

3. References 112

1. Experimental Section

1.1. General Procedures

Unless otherwise noted, all reactions were performed under an inert gas atmosphere (dry argon) using standard Schlenk techniques. All air and moisture sensitive materials were stored in an MBraun or an Innovative Technology glovebox under dry argon, with an integrated refrigerator. The mainly used solvents, such as diethylether, toluene, *n*-hexane, dichloromethane (DCM), and tetrahydrofuran (THF), were dried and degassed by means of an Innovative Technology solvent purification system (SPS). *n*-Pentane was dried over sodium and distilled freshly prior to use. Petroleum ether was distilled freshly prior to use. All deuterated solvents, such as DCM-d2, $CDCl_3$ and C_6D_6, for NMR spectroscopy were purchased from Sigma Aldrich, dried over molecular sieve, degassed at reflux or freeze-pumped, and distilled prior to use. Tetramethylethylenediamine (TMEDA), diisopropylamine, trichlorophosphane (PCl_3), 2-bromothiophene, furan, and trimethyl-phosphate were purchased from commercial sources, freshly distilled prior to use, and stored over molecular sieve. Trimethylsilylchloride (TMS-Cl) was purchased from Sigma Aldrich, dried over CaH_2, and distilled prior to use. Boron tribromide, supermesitylene, *n*-butyllithium (2.5 M in *n*-hexane and 1.6 M in hexane), and *t*-butyllithium (1.7 M in pentane) were purchased from commercial sources and used without further purification. Supermesitylene lithium (Mes*Li),[1] 1-bromosupermesitylene (Mes*Br)[2], and 1-bromo-4-octylxylole[3] were synthesized according to literature procedures.

All NMR data were recorded at 298 K on a Bruker Avance III FT NMR Spectrometer (operating at ^{1}H: 300 MHz, ^{11}B: 96 MHz, ^{13}C: 75 MHz, ^{31}P: 122 MHz) or a Bruker Avance 500 FT NMR spectrometer (operating at ^{1}H: 500 MHz, ^{11}B: 160 MHz, ^{13}C: 126 MHz, ^{31}P: 202 MHz). All chemical shifts (δ) were referenced to residual solvent peaks for ^{1}H-NMR spectra. The NMR-data was evaluated and plotted with Bruker TopSpin in the version 4.1.4.

Mass spectroscopy was performed on a Thermo Scientific Exactive Plus Orbitrap MS system, by either atmospheric pressure chemical ionization (APCI) or by liquid injection field desorption ionization (LIFDI). Thermogravimetric analysis (STA: DTA & TG) measurements were performed with a STA 449 F3 Perseus (Netzsch), connected to a QMS 403 Aeolos Quadro Mass Spectrometer for the analysis of the gaseous compounds in the temperature range of 30 to 700 °C with heating rate of 10 K min–1 in an argon atmosphere (flow rate: 70 mL min–1).

All optical measurements were performed under dry argon atmosphere in standard quartz cuvettes (1 cm x 1 cm cross-section). UV–visible absorption spectra were recorded using an Agilent 8453 diode array UV-visible spectrophotometer or a Mettler Toledo MT UV7 spectrophotometer under nitrogen-atmosphere in a MBraun Labmaster Pro glovebox. All solutions that were prepared for optoelectronic measurements were solved in THF and had a concentration lower than 2×10^{-5} M to minimize inner filter effects. All measured spectra were evaluated and plotted with Origin 2018b.

Cyclic voltammetry (CV) and square wave (SW) experiments were conducted in a nitrogen-filled MBraun Labmaster Pro glovebox using a Gamry Instruments 1010B potentiostat. A standard three-electrode cell configuration was employed using a platinum working electrode (d = 3 mm), a platinum wire counter electrode, and a silver wire reference electrode separated by a Vycor® frit, serving as a pseudoreference electrode. The redox potentials are referenced to the ferrocene/ferrocenium [Fc/Fc+] redox couple as an internal standard. Tetra-*n*-butylammonium hexafluorophosphate [nBu$_4$N][PF$_6$] was employed as the supporting electrolyte with a concentration of 0.1 mol/L in THF.

All analytical gel permeation chromatography (GPC) chromatograms were recorded on an Agilent 1260 Infinity II Series GPC equipped with a PSS SDV 3 µm precolumn, two PSS SDV 3 µm 1000 Å columns, and one PSS SDV 3 µm 10000 Å in series. All measurements were conducted in THF at 298 K with a flow rate of 1.0 mL/min and toluene as internal standard (calibrated against polystyrene standards). The injection volume was 50.0 µL. Three different detectors were applied for observation, i.e., two variable wavelength detectors (VWD 1 and 2) and a refraction index detector (RID). All measured chromatograms were evaluated using WinGPC software and plotted with Origin 2018b.

1.2. Syntheses

Synthesis of (2,4,6-trimethylphenyl)bromoborylbenzene (3). To a stirred suspension of MesLi (1.2547 g, 9.95 mmol) in toluene (10 mL) was added dropwise a solution of dibromoborylbenzene (2.6065 g, 10.52 mmol) in toluene (10 mL) at −78 °C. Subsequently, the mixture was warmed to room temperature and stirred overnight. The solid was filtered off and all volatiles were removed in vacuo. The slight yellowish oil was purified via distillation (105 °C, $4 \cdot 10^{-2}$ mbar) to obtain the product as a colourless oil. Yield: 2.473 g (8.62 mmol, 87 %). ^{1}H NMR (400 MHz, CDCl3): *δ* = 8.06 (d, *J* = 7.53 Hz, 2H, *o*-Ph-H), 7.67 (t, *J* = 7.53 Hz, 1H, *p*-Ph-H), 7.49 (dd, *J* = 7.53 Hz, 2H, *m*-Ph-H), 6.92 (s, 2H, Mes-CH arom.), 2.39 (s, 3H, *p*-Mes-CH_3), 2.19 ppm (s, 6H, *o*-Mes-CH_3); ^{11}B{^{1}H} NMR (128 MHz, $CDCl_3$): *δ* = 70.8 ppm (s).

Synthesis of 1,4-bis[(2,4,6-trimethylphenyl)bromoboryl]benzene (6). To a suspension of 1,4-bis(dibromoboryl)-benzene (1.6531 g, 3.96 mmol) in toluene (20 mL) was slowly added a suspension of MesLi (1.00 g, 7.93 mmol) in toluene (20 mL) at −78 °C. Subsequently, the mixture was warmed to room temperature and stirred overnight. The solid was filtered off and all volatiles were removed in vacuo. The residue was recrystallized from DCM to obtain the product as an off-white solid. Yield: 1.659 g (3.28 mmol, 83 %). ^{1}H NMR (500 MHz, C_6D_6): *δ* = 7.99 (s, 4H, Ph-H), 6.71 (s, 4H, Mes-CH arom.), 2.17 (s, 6H, *p*-Mes-CH_3), 2.05 ppm (s, 12H, *o*-Mes-CH_3); ^{11}B{^{1}H} NMR (160 MHz, C_6D_6): 73.3 ppm (s).

Synthesis of 1,4-bis[(2,4-dimethyl-6-*n*-octylphenyl)bromoboryl]benzene (10). To a solution of 1,4-bis(dibromo-boryl)benzene (125 mg, 0.3 mmol) in DCM (3 mL) was added 2,4-dimethyl-6-*n*-octyl-1-trimethylsilylbenzene (174 mg, 0.6 mmol) at room temperature. TMS-NTf_2 catalyst (10 mg, 10mol%) was added at r.t. After 24 h all volatiles were removed in vacuo and the cure product was extracted with *n*-pentane. The solvent was removed under reduced pressure for 24 h yielding **10** as sticky solid (113 mg, 0.16 mmol, 54%). ^{1}H NMR (300 MHz, $CDCl_3$): *δ* = 8.03 (s, 4H, Ph-H), 6.84 (s, 4H, Mes-CH arom.), 2.57 (t, 4H, c1-octyl-CH_2), 2.05 (s, 12H, *o*-Xyl-CH_3), 1.63 (m, 6H, c8-octyl-CH_3), 1.30 ppm (m, 28H, c2-c7-octyl-CH_2).

General synthesis of aryl(phenyl)chlorophosphines. To a stirred suspension of the corresponding aryl lithium compound (10.00 mmol) in toluene (30 mL), *P*,*P*-dichlorophenylphosphine (11.00 mmol) was added dropwise at −78 °C. Subsequently, the mixture was warmed to room temperature and stirred overnight. All volatiles were removed in vacuo. The solid residue was extracted with *n*-hexane (30 mL). After removing all volatiles in vacuo, the products were obtained as yellowish oils. Yields: 95 % (Mes); 95 % (Tip); 96 % (Mes*).

Mes: ^{1}H NMR (400 MHz, $CDCl_3$): δ = 7.36 (m, 5H, Ph-H), 6.93 (d, *J* = 3.07 Hz, 2H, Mes-CH arom.), 2.43 (d, *J* = 1.97 Hz, 6H, *o*-Mes-CH_3), 2.32 ppm (s, 3H, *p*-Mes-CH_3). ^{31}P{^{1}H} NMR (202 MHz, $CDCl_3$): δ = 82.1 ppm (s).

Tip: ^{1}H NMR (400 MHz, $CDCl_3$): δ = 7.36 (m, 5H, Ph-H), 7.11 (d, *J* = 2.89 Hz, 2H, Tip-CH arom.), 3.76 (oct, *J* = 6.51 Hz, 2H, *o*-Tip-CH), 2.93 (sept, *J* = 6.97 Hz, 1H, *p*-Tip-CH), 1.30 (d, *J* = 6.92 Hz, 6H, *o*-Tip-CH_3), 1.24 (d, *J* = 6.79 Hz, 6H, *o*-Tip-CH_3), 1.01 ppm (d, *J* = 6.74 Hz, 6H, *p*-Tip-CH_3); ^{31}P{^{1}H} NMR (202 MHz, $CDCl_3$): δ = 78.6 ppm (s).

Mes*: ^{1}H NMR (400 MHz, $CDCl_3$): δ = 7.47 (d, *J* = 2.52 Hz, 2H, Mes*-CH arom.), 7.14 (m, 3H, Ph-H), 6.75 (m, 2H, Ph-H), 1.41 (s, 18H, *o*-Mes*-CH_3), 1.36 ppm (s, 9H, *p*-Mes*-CH_3); ^{31}P{^{1}H} NMR (202 MHz, $CDCl_3$): δ = 75.3 ppm (s).

General synthesis of aryl(phenyl)phosphines. To a stirred suspension of lithium aluminium hydride (11.00 mmol) in diethyl ether (20 mL) was added dropwise a solution of the corresponding aryl(phenyl)chlorophosphine (10.00 mmol) in diethyl ether (20 mL) at 0 °C. The mixture was stirred for 24 h. Subsequently, degassed water was added at 0 °C in small portions until the remaining lithium aluminium hydride clumped together. The supernatant solution was separated and dried in vacuo. The products were obtained as colourless oils (Mes and Tip) or as white solid (Mes*), respectively. Yields: 85 % (Mes); 80 % (Tip); 80 % (Mes*).

Mes: ^{1}H NMR (400 MHz, $CDCl_3$): δ = 7.24 (m, 5H, Ph-H), 6.96 (s, 2H, Mes-CH arom.), 5.37 (d, *J* = 223.56 Hz, 1H, PH), 2.42 (s, 6H, *o*-Mes-CH_3), 2.31 ppm (s, 3H, *p*-Mes CH_3). ^{31}P{^{1}H} NMR (202 MHz, $CDCl_3$): δ = −76.6 ppm (s).

Tip: ^{1}H NMR (400 MHz, $CDCl_3$): δ = 7.24 (m, 5H, Ph-H), 7.10 (d, *J* = 2.32 Hz, 2H, Tip-CH arom.), 5.39 (d, *J* = 221.85 Hz, 1H, PH), 3.60 (oct, *J* = 6.79 Hz, 2H, *o*-Tip-CH), 2.93 (sept, *J* = 6.91 Hz, 1H, *p*-Tip-CH), 1.30 (d, *J* = 6.91 Hz, 6H, *p*-Tip-CH_3), 1.19 (d, *J* = 6.79 Hz, 6H, *o*-Tip-CH_3), 1.15 ppm (d, *J* = 6.85 Hz, 6H, *o*-Tip-CH_3); ^{31}P{^{1}H} NMR (202 MHz, $CDCl_3$): δ = −82.8 ppm (s).

Mes*: ^{1}H NMR (400 MHz, $CDCl_3$): δ = 7.53 (d, *J* = 2.26 Hz, 2H, Mes*-CH arom.), 7.12 (m, 3H, Ph-H), 6.70 (m, 2H, Ph-H), 6.10 (br d, *J* = 200.03 Hz, 1H, PH), 1.49 (s, 18H, *o*-Mes*-CH_3), 1.38 ppm (s, 9H, *p*-Mes*-CH_3); ^{31}P{^{1}H} NMR (202 MHz, $CDCl_3$): δ = −65.1 ppm (s).

General synthesis of potassium aryl(phenyl)phosphanides (5). Benzyl potassium (2.00 mmol) and the corresponding aryl(phenyl)phosphine (2.05 mmol) were placed in a Schlenk flask and *n*-hexane (10 mL) and TMEDA (2.20 mmol) were added. After stirring for 2 h, all volatiles were removed in vacuo. The solid crude product was washed with *n*-hexane (40 mL). Drying the precipitate in vacuo gave the products as yellow (Mes and Tip) and orange (Mes*) solids.

Mes: ^{1}H NMR (400 MHz, THF-d8): δ = 6.71 (d, 2H, Mes-CH arom.), 6.69 (t, 2H, *o*-Ph-H), 6.48 (t, 2H, *m*-Ph-H), 6.06 (t, 1H, *p*-Ph-H), 2.48 (s, 6H, *o*-Mes-CH_3), 2.16 ppm (s, 3H, *p*-Mes-CH_3); ^{31}P{^{1}H} NMR (202 MHz, THF-d8): δ = −34.5 ppm (s);

Tip: ^{1}H NMR (400 MHz, THF-d8): *δ* = 6.86 (d, *J* = 1.04 Hz, 2H, Tip-CH arom.), 6.59 (t, 2H, *o*-Ph-H), 6.40 (t, 2H, *m*-Ph-H), 5.95 (t, 1H, *p*-Ph-H), 4.48 (oct, *J* = 6.91 Hz, 2H, *o*-Tip-CH), 2.79 (sept, *J* = 6.91 Hz, 1H, *p*-Tip-CH), 1.23 (d, *J* = 6.91 Hz, 6H, *p*-Tip-CH_3), 1.10 ppm (d, *J* = 6.91 Hz, 12H, *o*-Tip-CH_3); ^{31}P{^{1}H} NMR (202 MHz, THF-d8): *δ* = −56.1 ppm (s);

Mes*: ^{1}H NMR (400 MHz, THF-d8): *δ* = 7.33 (d, *J* = 1.04 Hz, 2H, Mes*-CH arom.), 6.75 (br, 1H, Ph-H), 6.24 (br, 2H, Ph-H), 5.69 (t, 1H, *p*-Ph-H), 5.08 (br, 1H, Ph-H), 1.70 (d, *J* = 1.28 Hz, 18H, *o*-Mes*-CH_3), 1.30 ppm (s, 9H, *p*-Mes*-CH_3); ^{31}P{^{1}H} NMR (202 MHz, THF-d8): *δ* = −14.5 ppm (s).

Synthesis of *p*-phenylene-bis(*N,N,N′,N′*-tetramethyl-phosphinediamine). To a suspension of 1,4-dilithiobenzene (60 mmol) in diethyl ether (120 mL), bis(*N,N*-dimethylamino)chlorophosphine (18.25 g, 118.06 mmol) was added at −40 °C. After stirring for 30 min at −40 °C the mixture was warmed to room temperature and stirred overnight. The solid was filtered off and all volatiles were removed in vacuo. Recrystallization from diethyl ether gave the product as an off-white solid. Yield: 15.2 g (48.3 mmol, 81 %). ^{1}H NMR (400 MHz, $CDCl_3$): *δ* = 7.38 (t, *J* = 3.92 Hz, 4H, Ph-H), 2.78 ppm (d, *J* = 9.17 Hz, 24H, N-CH_3); ^{31}P{^{1}H} NMR (122 MHz, $CDCl_3$): *δ* = 100.1 ppm (s).

Synthesis of *p*-phenylene-bis(dichlorophosphine). To a solution of *p*-phenylene-bis(*N*,*N*,*N'*,*N'*-tetramethyl-phosphinediamine) (6.80 g, 21.63 mmol) in diethyl ether (200 mL) was added hydrogen chloride (2.0 M in diethyl ether, 88 mL, 176.0 mmol) at 0 °C. Subsequently, the mixture was warmed to room temperature and stirred overnight. The solid was filtered off and all volatiles were removed in vacuo. Subsequently, the residue was taken up in DCM, precipitated by adding *n*-pentane and cooled to −20 °C. The precipitate was filtered and dried in vacuo to obtain the product as a yellow solid. Yield: 4.68 g (16.7 mmol, 76 %). ^{1}H NMR (300 MHz, C_6D_6): δ = 7.31 ppm (q, 4H, Ph-H); ^{31}P{^{1}H} NMR (122 MHz, C_6D_6): δ = 157.1 ppm (s).

Synthesis of *p*-phenylene-bis[(2,4,6-tri-*tert*-butylphenyl)chlorophosphine]. To a suspension of Mes*Li (4.86 g, 19.26 mmol) in toluene (70 mL) was added a suspension of *p*-phenylene-bis(dichlorophosphine) (2.625 g, 9.38 mmol) in toluene (30 mL) at −78 °C. Subsequently, the mixture was warmed to room temperature and stirred overnight. The solid was filtered off and all volatiles were removed in vacuo. The product was obtained as a white solid. Yield: 5.45 g (7.79 mmol, 83 %). ^{1}H NMR (300 MHz, C_6D_6): δ = 7.54 (d, *J* = 6.05 Hz, 4H, Mes*-CH arom.), 6.76 (d, *J* = 16.32 Hz, 4H, Ph-H), 1.50 (d, *J* = 2.57 Hz, 36H *o*-Mes*-CH_3), 1.24 ppm (d, *J* = 3.85 Hz, 18H, *p*-Mes*-CH_3); ^{31}P{^{1}H} NMR (122 MHz, C_6D_6): δ = 74.9 (d, *J* = 1.94 Hz), 74.0 ppm (d, *J* = 1.94 Hz).

Synthesis of *p*-phenylene-bis[(2,4,6-tri-*tert*-butylphenyl)phosphine] (7H). To a stirred suspension of lithium aluminium hydride (1.2 g, 31.62 mmol) in THF (60 mL) was added a suspension of *p*-phenylene-bis((2,4,6-tri-*tert*-butylphenyl)chlorophosphine) (4.901 g, 7.00 mmol) in THF (55 mL) at −78 °C. After stirring for 2 h at −78 °C the mixture was warmed to room temperature and stirred overnight. Degassed water was added at 0 °C until the remaining lithium aluminium hydride clumped together. The solid was washed with *n*-hexane (2 x 50 mL), the solution dried over $MgSO_4$, filtered, and dried in vacuo. Recrystallization from diethyl ether gave the product as a white solid. Yield: 3.14 g (4.97 mmol, 71 %). ^{1}H NMR (500 MHz, C_6D_6): 7.62 (d, *J* = 2.09 Hz, 4H, Mes*-CH arom.), 6.64 (m, 4H, Ph-H), 6.12 (dd, *J* = 227.70 Hz, 2H, PH), 1.49 (br, 36H, *o*-Mes*-CH_3), 1.30 ppm (s, 18H, *p*-Mes*-CH_3); ^{31}P{^{1}H} NMR (202 MHz, C_6D_6): δ = −64.9 ppm (d, *J* = 11.56 Hz).

Synthesis of potassium *p*-phenylene-bis[(2,4,6-tri-*tert*-butylphenyl) phosphinide] (9). Benzyl potassium (0.0801 g, 0.62 mmol) and *p*-phenylene-bis((2,4,6-tri-*tert*-butylphenyl)-phosphine) (189.2 mg, 0.30 mmol) were placed in a Schlenk flask and THF (12 mL) was added. The mixture was stirred for 42 h at 50 °C. All volatiles were removed in vacuo and the residue was taken up in toluene (10 mL). After filtration and removing all volatiles in vacuo the product was obtained as a dark red solid. Yield: 136.0 mg (0.19 mmol, 63 %). ^{1}H NMR (400 MHz, THF-d8): δ = 7.24 (s, 4H, Mes*-CH arom.), 7.19 (br, 4H, Ph-H), 1.31 (s, 36H, *o*-Mes*-CH_3), 1.25 ppm (s, 18H, p-Mes*-CH_3); ^{31}P{^{1}H} NMR (162 MHz, THF-d8): δ = −22.7 ppm (s).

General synthesis of 1^{Ar}. Compound **3** (0.50 mmol) and the corresponding PhArPK (**5**) (0.50 mmol) were placed in a Schlenk flask, *n*-hexane (10 mL) and TMEDA (0.50 mmol) were added, and the mixture was stirred for 3 h. All volatiles were removed in vacuo, the residue dissolved in *n*-hexane and crystallized. The products were obtained as yellow solids.

Mes: ^{1}H NMR (500 MHz, THF-d8): δ = 7.24 (m, 2H, B-*o*-Ph-H), 7.20 (m, 1H, B-*p*-Ph-H), 7.09 (d, *J* = 2.52 Hz, 2H, P-Mes-CH arom.), 7.06 (t, *J* = 7.57 Hz, 2H, B-*m*-Ph-H), 6.98 (m, 1H, P-*p*-Ph-H), 6.93 (m, 2H, P-*o*-Ph-H), 6.84 (s, B-Mes-CH arom.), 6.62 (m, 2H, P-*m*-Ph-H), 2.46 (s, 6H, P-*o*-Mes-CH_3), 2.35 (s, 3H, P-*p*-Mes-CH_3), 2.31 (s, 3H, B-*p*-Mes-CH_3), 2.14 ppm (d, *J* = 1.58 Hz, 3H, B-*o*-Mes-CH_3); ^{11}B{^{1}H} NMR (160 MHz, THF-d8): δ = 65.1 ppm; ^{13}C{^{1}H} NMR (126 MHz, THF-d8): δ = 144.9 (d, *J* = 9.54 Hz, quart. P-*o*-Mes-C), 140.9 (br, quart. Ph-C-B), 140.7 (d, *J* = 2.15 Hz, quart. P-*p*-Mes-C), 139.6 (br, quart. Mes-C-B), 138.1 (d, *J* = 8.73 Hz, quart. B-*o*-Mes-C), 136.8 (d, *J* = 2.28 Hz, quart. B-*p*-Mes-C), 133.6 (d, *J* = 12.90 Hz, B-*o*-Ph-C), 132.8 (d, *J* = 36.94 Hz, quart. Ph-C-P), 130.3 (d, *J* = 2.69 Hz, B-*p*-Ph-C), 129.1 (d, *J* = 7.79 Hz, P-Mes-CH arom.), 128.7 (d, *J* = 10.61 Hz, P-*m*-Ph-CH), 127.8 (d, *J* = 10.34 Hz, P-*o*-Ph-CH), 127.5 (s, B-*m*-Ph-CH), 127.4 (d, *J* = 1.34 Hz, B-Mes-CH arom.), 126.1 (s, P-*o*-Ph-CH), 126.0 (d, *J* = 41.51 Hz, quart. Mes-C-P), 23.5 (d, *J* = 10.17 Hz, P-*o*-Mes-CH_3), 22.3 (d, *J* = 2.42 Hz, B-*o*-Mes-CH_3), 20.4 ppm (dd, B-*p*-Mes-CH_3 and P-*p*-Mes-CH_3); ^{31}P{^{1}H} NMR (202 MHz, THF-d8): δ = –8.9 ppm (s); HRMS (LIFDI): m/z (%) = 434.2328, calcd. for $C_{30}H_{32}BP$: 434.2335; UV/vis (THF): $\lambda_{abs,max}$ 368 nm (ε = 20698 L*mol^{-1}*cm^{-1}).

Tip: ^{1}H NMR (500 MHz, THF-d8): δ = 7.25 (d, *J* = 3.31 Hz, 2H, Tip-CH arom.), 7.16 (m, 1H, B-*p*-Ph-H), 7.13 (m, 2H, B-*o*-Ph-H), 7.00 (t, *J* = 7.57 Hz, 2H, B-*m*-Ph-H), 6.95 (m, 3H, P-Ph-H), 6.88 (s, 2H, Mes-CH arom.), 6.65 (m, 2H, P-Ph-H), 3.92 (oct, *J* = 6.78 Hz, 2H, *o*-Tip-CH), 3.00 (sept, *J* = 6.78 Hz, 1H, *p*-Tip-CH), 2.33 (s, 3H, *p*-Mes-CH_3), 2.19 (d, *J* = 1.73 Hz, 6H, *o*-Mes-CH_3), 1.34 (d, *J* = 6.94 Hz, 6H, *p*-Tip-CH_3), 1.10 (d, *J* = 6.94 Hz, 6H, *o*-Tip-CH_3), 1.01 ppm (d, *J* = 6.94 Hz, 6H, *o*-Tip-CH_3); ^{11}B{^{1}H} NMR (160 MHz, THFd8): δ = 62.3 ppm; ^{13}C{^{1}H} NMR (126 MHz, THF-d8): δ = 155.0 (d, *J* = 9.40 Hz, quart. *o*-Tip-C), 152.4 (d, *J* = 2.28 Hz, quart. *p*-Tip-C), 140.7 (br, quart. Ph-C-B), 139.8 (br, quart. Mes-C-B), 138.2 (d, *J* = 8.87 Hz, quart. *o*-Mes-C), 136.9 (s, quart. *p*-Mes-C), 134.3 (d, *J* = 40.97 Hz, quart. Ph-C-P), 134.2 (d, *J* = 13.16 Hz, P-*o*-Ph-CH), 129.9 (d, *J* = 2.96 Hz, P-*p*-Ph-CH), 128.8 (d, *J* = 10.07 Hz, B-*o*-Ph-CH), 127.7 (d, *J* = 10.61 Hz, B-*m*-Ph-CH), 127.5 (d, *J* = 1.34 Hz, Mes-CH), 127.2 (s, P-*m*-Ph-CH), 126.0 (d, *J* = 2.82 Hz, B-*p*-Ph-CH), 124.6 (d, *J* = 45.40 Hz, Tip-C-P), 122.4 (d, *J* = 8.19 Hz, Tip-CH), 35.1 (d, *J* = 10.21 Hz, *o*-Tip-CH), 34.4 (s, *p*-Tip-CH), 24.2 (s, *o*-Tip-CH_3), 23.6 (s, *o*-Tip-CH_3), 23.2 (s, *p*-Tip-CH_3), 22.1 (d, *J* = 2.42 Hz, *o*-Mes-CH_3), 20.4 ppm (d, *J* = 0.81 Hz, *p*-Mes-CH_3); ^{31}P{^{1}H} NMR (202 MHz, THF-d8): *δ* = –12.3 ppm (s); HRMS (LIFDI): m/z (%) = 518.3268, calcd. for $C_{36}H_{44}BP$: 518.3274; UV/vis (THF): $\lambda_{abs,max}$ 372 nm (ε = 19594 L*mol^{-1}*cm^{-1}); fluorescence (THF): λ_{em}= 544 nm (for λ_{ex} = 372 nm; Φf = 0.02).

Mes*: ^{1}H NMR (500 MHz, THF-d8): δ = 7.83 (m, 2H, Mes*-CH arom.), 7.12 (m, 3H, B-Ph-H), 7.00 (m, 2H, B-*m*-Ph-H), 6.95 (s, Mes-CH arom.), 6.90 (m, 3H, P-Ph-H), 6.50 (m, 2H, P-*m*-Ph-H), 2.37 (s, 3H, *p*-Mes-CH_3), 2.27 (s, 6H, *o*-Mes-CH_3), 1.54 (d, *J* = 2.52 Hz, 18H, *o*-Mes*-CH_3), 1.45 ppm (d, *J* = 1.42 Hz, 9H, *p*-Mes*-CH_3); ^{11}B{^{1}H} NMR (160 MHz, THF-d8): δ = 49.7 ppm; ^{13}C{^{1}H} NMR (126 MHz, THF-d8): δ = 157.7 (d, *J* = 5.64 Hz, quart. *o*-Mes*-C), 153.5 (d, *J* = 2.96 Hz, quart. *p*-Mes*-C), 140.0 (br, quart. Mes-C-B), 139.6 (br, quart. Ph-C-B), 139.4 (d, *J* = 9.80 Hz, quart. *o*-Mes-C), 137.7 (d, *J* = 66.22 Hz, quart. Ph-CP), 136.8 (d, *J* = 2.28 Hz, quart. *p*-Mes-C), 135.8 (d, *J* = 12.90 Hz, B-*o*-Ph-CH), 129.2 (d, *J* = 2.69 Hz, B-*p*-Ph-CH), 128.8 (d, *J* = 7.25 Hz, P-*m*-Ph-CH), 127.8 (s, Mes-CH arom.), 127.6 (d, *J* = 12.49 Hz, P-*o*-Ph-CH), 126.8 (s, B-*m*-Ph-CH), 125.7 (d, *J* = 3.90 Hz, P-*p*-Ph-CH), 124.4 (d, *J* = 10.48 Hz, Mes*-CH arom.), 121.3 (d, *J* = 52.79 Hz, quart. Mes*-C-P), 39.6 (s, quart. *o*-Mes*-*t*-Bu-C), 35.2 (s, quart. *p*-Mes*-*t*-Bu-C), 32.7 (s, *o*-Mes*-CH_3), 30.6 (s, *p*-Mes*-CH_3), 22.7 (s, *o*-Mes-CH_3), 20.5 ppm (s, *p*-Mes-CH_3); ^{31}P{^{1}H} NMR (202 MHz, THF-d8): δ = 16.4 ppm (s); HRMS (LIFDI): m/z (%) = 560.3735, calcd. for $C_{39}H_{50}BP$: 560.3743; UV/vis (THF): $\lambda_{abs,max}$ 374 nm (ε = 35324 L*mol^{-1}*cm^{-1}); fluorescence (THF): λ_{em}= 603 nm (for λ_{ex} = 374 nm; Φ_f = 0.06).

Synthesis of 2^a. Compound **6** (198 mg, 0.40 mmol) and **5^Mes*** (314.1 mg, 0.80 mmol) were placed in a Schlenk flask, *n*-hexane (8 mL) was added, and the mixture was stirred for 72 h at ambient temperature. All volatiles were removed in vacuo and the residue was taken up in DCM. After filtration and removing DCM in vacuo **2^a** was obtained as a yellow solid (281 mg, 68 %, 0.27 mmol).

^{1}H NMR (500 MHz, 75°C, toluene-d8): *δ* = 7.76 (d, 4H, Mes*-CH arom.), 7.03 (s, 4H, B-Ph-H), 6.88 (s, 4H, Mes-CH arom.), 6.70 (m, 10H, P-Ph-H), 2.35 (s, 6H, *p*-Mes-CH_3), 2.29 (s, 12H, *o*-Mes-CH_3), 1.56 (s, 36H, *o*-Mes*-CH_3), 1.31 ppm (s, 18H, *p*-Mes*-CH_3); ^{11}B{^{1}H} NMR (160 MHz, 75 °C, toluene-d8): δ = 50.7 ppm; ^{13}C{^{1}H} NMR (126 MHz, toluene-d8): δ = 158.2 (d, *J* = 5.79 Hz, quart. *o*-Mes*-C), 153.1 (d, *J* = 2.95 Hz, quart. *p*-Mes*-C), 139.4 (d, *J* = 10.00 Hz, quart. *o*-Mes-C), 138.3 (s. Mes-C-B), 137.8 (s, Ph-C-B), 137.0 (d, *J* = 57.10 Hz, quart. Ph-CP), 136.6 (d, *J* = 2.28 Hz, quart. *p*-Mes-C), 134.3 (d, *J* = 12.20 Hz, B-*o*-Ph-CH), 129.2 (d, *J* = 7.50 Hz, P-*m*-Ph-CH), 128.1 (s, Mes-CH arom.), 127.6 (d, *J* = 12.49 Hz, P-*o*-Ph-CH), 125.7 (d, *J* = 4.00 Hz, P-*p*-Ph-CH), 124.4 (d, *J* = 10.63 Hz, Mes*-CH arom.), 121.3 (d, *J* = 51.51 Hz, quart. Mes*-C-P), 39.8 (d, *J* = 2.58 Hz, quart. *o*-Mes*-*t*-Bu-C), 35.0 (s, quart. *p*-Mes*-*t*-Bu-C), 33.2 (s, *o*-Mes*-CH_3), 30.9 (s, *p*-Mes*-CH_3), 23.1 (s, *o*-Mes-CH_3), 20.8 ppm (s, *p*-Mes-CH_3); ^{31}P NMR (202 MHz, 75 °C, toluene-d8): δ = 18.1 ppm. HRMS (LIFDI): m/z (%) = 1042.7017, calcd. for $C_{72}H_{94}B_2P_2$: 1042.6995. UV/vis (THF): $\lambda_{abs,max}$ = 423 nm; fluorescence (THF): $\lambda_{em,max}$ = 628 nm.

Synthesis of 8. Compound **7H** (189.3 mg, 0.30 mmol) and **BzK** (39.1 mg, 0.30 mmol) were placed in a Schlenk flask, *n*-hexane (2 mL) and TMEDA (46.5 mg, 0.40 mmol) were added, and the mixture was stirred for 4 h at 25 °C. PhBMesBr (**3**) (86.1 mg, 0.30 mmol) was slowly added dropwise. The mixture was stirred for 24 h. The solvent was filtered off and the bright yellow crude product was extracted with DCM. After evaporation of DCM, **8** was obtained as a bright yellow solid. Yield: 191.0 g (0.23 mmol, 76 %). ^{1}H NMR (300 MHz, $CDCl_3$): *δ* = 7.85 (d, *J* = 3.58 Hz, 2H, Mes*-CH arom.), 7.56 (d, *J* = 2.16 Hz, 2H, Mes*-CH arom.), 7.45 (m, 2H, phenylene-CH), 6.99 (s, 2H, Mes-CH arom.), 6.93 (m, 3H, phenyl-CH), 6.59 (m, 2H, penyl-CH), 6.47 (m, 2H, phenylene-CH), 6.00 (d, *J* = 226 Hz, 1H, P-H), 2.46 (s, 6H, *o*-Mes CH_3), 2.37 (s, 3H, *p*-Mes CH_3), 1.62 (s, 18H, Mes* CH_3), 1.44 (broad s, 1H, Mes* CH_3), 1.29 ppm (d, *J* = 4.08 Hz, 18H, Mes* CH_3); ^{11}B{^{1}H} NMR (128 MHz, $CDCl_3$): δ = 49.6 ppm; ^{31}P NMR (122 MHz, $CDCl_3$): δ = 17.5 ppm (s, 1P), –64.5 ppm (d, 1P, *J* = 226 Hz).

Synthesis of 2^b. Compound **8** (167.0 mg, 0.20 mmol) and BzK (57.7 mg, 0.20 mmol) were placed in a Schlenk flask, *n*-hexane (1.5 mL) and TMEDA (23.0 mg, 0.20 mmol) were added, and the mixture was stirred for 24 h at 25 °C. The solid was filtered off and all volatiles were removed in vacuo. The residue was washed with THF 5 times, and with *n*-pentane 3 times. **2^b** was obtained as a yellow solid. Yield: 155 mg, 0.15 mmol, 74%. ^{1}H solid-state NMR (400 MHz, MAS = 14800 Hz): *δ* = 7.7 (broad s for all aromatic protons), 1.8 ppm (broad s for all aliphatic protons); ^{11}B{^{1}H} solid-state NMR (128 MHz, MAS = 14800 Hz): *δ* = 36 ppm; ^{13}C solid-state NMR (101 MHz, MAS = 14800 Hz): *δ* = 157.0 (d, *J* = 38.1 Hz, quart. *o*-Mes*-C), 153.9 (s, quart. *p*-Mes*-C), 139.5 (d, *J* = 60.0 Hz, quart. *o*-Mes-C), 138.8 (s. Mes-C-B), 138.1 (d, *J* = 270.1 Hz, quart. Ph-CP), 137.8 (s, Ph-C-B), 136.3 (broad s, quart. *p*-Mes-C), 130.5 (s, Mes-CH arom.), 129.2 (d, *J* = 7.50 Hz, B-*m*-Ph-CH), 129.1 (d, *J* = 45.5 Hz, P-*o*-Ph-CH), , 127.1 (s, B-*o*-Ph-CH), 125.9 (s, B-*p*-Ph-CH), 123.7 (s, Mes*-CH arom.), 121.9 (d, *J* = 47.9 Hz, quart. Mes*-C-P), 39.9 (s, quart. *o*-Mes*-*t*-Bu-C), 35.0 (s, quart. *p*-Mes*-*t*-Bu-C), 34.0 (d, *J* = 31.8 Hz *o*-Mes*-CH_3), 30.4 (s, *p*-Mes*-CH_3), 24.8 (d, *J* = 70.7 Hz, *o*-Mes-CH_3), 21.2 ppm (s, *p*-Mes-CH_3); ^{31}P{^{1}H} solid-state NMR (162 MHz, MAS = 14800 Hz): *δ* = 19.5 ppm. HRMS (LIFDI): m/z (%) = 1042.6991, calcd. for $C_{72}H_{94}B_2P_2$: 1042.6995; UV/vis (THF): $\lambda_{abs,max}$ = 432 nm; fluorescence (THF): $\lambda_{em,max}$ = 591 nm.

Synthesis of 4. Compound **8** (83.7 mg, 0.10 mmol) was dissolved in a mixture of *n*-hexane (3.0 mL) and TMEDA (11.6 mg, 0.10 mmol). At ambient temperature BzK (13.0 mg, 0.10 mmol) was added and stirred for 2 h. 1,4-bis(dibromoboryl)benzene (**6**) (24.8 mg, 0.05 mmol) was added and the mixture was stirred for 14 h. All solvents were removed at high vacuum and the residue was suspended in THF and centrifugated 3 times. The product was obtained as an orange solid. Yield: 64.0 mg, 32 %.

^{1}H NMR (500 MHz, THF-d8): δ = 7.77 (d, J = 3.68 Hz, 4H, Mes*-CH arom.), 7.67 (d, J = 3.65 Hz, 4H, Mes*-CH arom.), 7.03 (m, 2H, B-Ph-H), 7.06 (m, 4H, Ph-H), 6.98 (m, 4H, Ph-H), 6.88 (s, 4H, Mes-CH arom.), 6.81 (s, 4H, Mes-CH arom.), 6.07 (m, 8H, Ph-H), 2.39 (s, 6H, *p*-Mes-CH_3), 2.36 (s, 6H, *p*-Mes-CH_3), 2.18 (s, 12H, *o*-Mes-CH_3), 2.12 (s, 12H, *o*-Mes-CH_3), 1.46 (s, 36H, *o*-Mes*-CH_3), 1.42 (s, 18H, *p*-Mes*-CH_3), 1.39 (s, 36H, *o*-Mes*-CH_3), 1.35 ppm (s, 18H, *p*-Mes*-CH_3); ^{11}B{^{1}H} solid-state NMR (128 MHz, MAS = 14800 Hz): δ = 38.7, 30.8 ppm; ^{31}P NMR{^{1}H, ^{11}B} (243 MHz, THF-d8): δ = 18.3, 16.8 ppm; ^{31}P{^{1}H} solid-state NMR (162 MHz, MAS = 14800 Hz): δ = 21.8, 19.8, 18.9 ppm; HRMS (LIFDI): m/z (%) = 2007.3600, calcd. for $C_{128}H_{182}B_4P_4$: 2007.3629. UV/vis (THF): $\lambda_{abs,max}$ = 474 nm; fluorescence (THF): $\lambda_{em,max}$ = 620 nm.

Synthesis of P1. *p*-Ph(Mes*PH)$_2$ (**7H**) (78.5 mg, 0.12 mmol) was dissolved in a mixture of toluene (2.0 mL) and TMEDA (36.2 mg, 0.31 mmol). At r.t. BzK (32.4 mg, 0.25 mmol) was added and stirred for 6 h. **10** (86.2 mg, 0.12 mmol) was added and the suspension was stirred for 16 h at ambient temperature. The polymerization was terminated with TMS-NMe_2. All solids were filtered off and the volatiles were removed at high vacuum. The crude product was dissolved in THF, precipitated in methanol and washed with two portions of dry methanol. The residue was dissolved in THF, precipitated in *n*-pentane and washed with two portions of *n*-pentane. The solid and liquid phases were separated by centrifugation. All solvents were removed at high vacuum yielding **P1** as orange solid. Yield: 85.3 mg, 59 %.

^{31}P NMR (122 MHz, $CDCl_3$): δ = 18.0 ppm, GPC (THF, vs. polystyrene, detection by UV signal): M_n = 3.07 kDa, M_w = 6.65 kDa; UV/vis (THF): $\lambda_{abs,max}$ = 490 nm; fluorescence (THF): $\lambda_{em,max}$ = 595 nm, Φ_f = 0.15.

1.3. Crystallographic Data

Crystals of compounds **1**^{Mes}, **1**^{Tip}, **1**^{Mes*}, **2**^{a}, **7H**, and **8** suitable for single-crystal X-ray diffraction were selected, coated in perfluoropolyether oil, and mounted on MiTeGen or polyimide microloops. Diffraction data were collected on a Bruker X8 Apex II 4-circle diffractometer with a CCD area detector (**2**^{a}) or an a Bruker D8-Quest 4-circle diffractometerwith a CPA area detector (Photon II) (**1**^{Mes}, **7H**, **8**) using multi-layer mirror monochromated Mo-K$_{\alpha}$ radiation, or on a Rigaku XtaLAB Synergy-S 4-circle diffractometer with an HPA area detector (HyPix-6000) using multi-layer mirror monochromated Cu-K$_{\alpha}$ radiation generated by a PhotonJet X-ray source (**1**^{Tip}, **1**^{Mes*}). The crystals were cooled using an Oxford Cryostreams low-temperature device. Data were collected at 100 K. The images were processed and corrected for Lorentz-polarization effects and absorption as implemented in the Bruker or CrysAlis^{Pro} (Rigaku Oxford Diffraction) software packages. The structures were solved using the intrinsic phasing method (SHELXT)[4] and Fourier expansion technique. All non-hydrogen atoms were refined in anisotropic approximation, with hydrogen atoms 'riding' in idealized positions, by full-matrix least squares against F^2 of all data, using SHELXL[5] software and the SHELXLE graphical user interface.[6] Other structural information was extracted using OLEX2 software.[7]

On compounds **2**^{b} and **4**, 3D electron diffraction data were collected for structure analysis. Powder of **2**^{b} was slightly smashed between two glass slides and a lacey carbon-supported copper grid was placed into the powder from both sides. For the preparation of **4**, a drop of a suspension of powder of **4** in ethanol was dropped onto a lacey carbon-supported copper grid and remaining liquid was soaked off. The grids were loaded via a Gatan Elsa cryo holder into a Rigaku XtaLAB Synergy-ED electron diffractometer, operated at 200 kV (λ = 0.0251 Å) and equipped with a Rigaku HyPix-ED hybrid pixel array area detector. Data on multiple crystallites of the order of 0.5 µm size were collected using continuous rotation 3D electron diffraction at 175(2) K under high vacuum. For **2**^{b}, data on five individual crystals were collected over a tilt range of 120°, and for **4**, data on four crystals over a tilt range between 95° and 120° (Table S1). These datasets were individually indexed and integrated before being scaled and merged into a single dataset using CrysAlis^{Pro} (version 1.171.43.124a; Rigaku Oxford Diffraction, 2024). By merging the data of all crystallites, a completeness of 100% (**2**^{b}) or 89% (**4**), respectively, up to a resolution of 0.83 Å was achieved. The structures were solved using SHELXT[4] implemented in Olex2[7] (version 1.5) and refined in the kinematic approximation using SHELXL[5] as implemented in the SHELXLE[6] graphical user interface using published scattering factors.[8] For the refinement of **2**^{b}, an extinction correction was applied to decrease the impact of dynamical effects. All non-hydrogen atoms were refined in anisotropic approximation, with hydrogen atoms 'riding' in idealized positions. A detailed description of the 3D ED method using the Rigaku electron diffractometer is given by Ito *et al.*[9] and Truong *et al.*[10]

Crystal data and experimental details are listed in Table S2; full structural information has been deposited with the Cambridge Crystallographic Data Centre. CCDC-2440917 (**1**^{Mes}), CCDC-2440918 (**1**^{Tip}), CCDC-2440919 (**1**^{Mes*}), CCDC-2440920 (**2**^{a}), CCDC-2440921 (**2**^{b}), CCDC-2440922 (**4**), CCDC-2440923 (**7H**), CCDC-2440924 (**8**).

Table S1. Experimental conditions of the individual 3D ED datasets of compounds **2[b]** and **4**.

Data collection No.	Scan range [°]	Scan width [°]	Exposure time/frame [s]	Total exposure time [s]	Dose rate [$e^-/(Å^2 \cdot s)$]	Dose [$e^-/(Å^2)$]
			Compound **2[b]**			
1	-60 to +60	0.5	1.0	240	8.76E-03	2.10
2	-60 to +60	0.5	1.0	240	8.76E-03	2.10
3	-60 to +60	0.5	1.0	240	8.76E-03	2.10
4	-60 to +60	0.5	1.0	240	8.76E-03	2.10
5	-60 to +60	0.5	1.0	240	5.44E-03	1.31
			Compound **4**			
1	-60 to +60	0.5	0.5	120	1.27E-01	15.24
2	-60 to +60	0.5	0.5	120	1.27E-01	15.24
3	-35 to +60	0.5	0.5	95	1.27E-01	12.06
4	-60 to +60	0.5	0.5	120	2.04E-01	24.48

Table S2. Single crystal X-ray and 3D-electron diffraction data and structure refinements of **1**^{Mes}, **1**^{Tip}, **1**^{Mes*}, **2**^{a}, **2**^{b}, **4**, **7H** and **8**.

Compound	**1**^{Mes}	**1**^{Tip}	**1**^{Mes*}
CCDC number	2440917	2440918	2440919
Empirical formula	$C_{30}H_{32}BP$	$C_{36}H_{44}BP$	$C_{45}H_{64}BP$
M_r	434.33	518.49	646.74
T / K	100(2)	100(2)	100(2)
Radiation, λ / Å	Mo-K$_\alpha$, 0.71073	Cu-K$_\alpha$, 1.54184	Cu-K$_\alpha$, 1.54184
Crystal size / mm³	0.363×0.227×0.168	0.232×0.114×0.041	0.159×0.120×0.053
Crystal color, habit	yellow block	colorless plate	colorless block
Crystal system	monoclinic	monoclinic	triclinic
Space group	$P2_1/n$	$C2/c$	P
a / Å	7.0692(15)	15.12310(10)	10.39980(10)
b / Å	9.0541(13)	12.70490(10	10.43580(10)
c / Å	18.975(4)	32.6233(2)	18.8046(2)
α / °	90	90	90.8600(10)
β / °	93.140(6)	92.2170(10)	90.6730(10)
γ / °	90	90	101.8830(10)
Volume / Å^3	1212.7(4)	6263.47(8)	1996.68(4)
Z	2	8	2
ρ_{calc} / g cm^{-3}	1.190	1.100	1.076
μ / mm^{-1}	0.129	0.918	0.802
F(000)	464	2240	708
θ range / °	2.150 – 28.323	2.711 – 72.127	2.350 – 72.121
Completeness	0.998	1.00	1.000
Reflections collected	19209	80638	43779
Unique reflections: [all data] / [I ≥ 2σ(I)]	3016 / 2576	6180 / 5976	7852 / 7375
R_{int}	0.0622	0.0347	0.0314
Parameters / restraints	299 / 648	352 / 0	676 / 612
GooF on F^2	1.040	1.110	1.028
R_1 [I ≥ 2σ(I)]	0.0579	0.0528	0.0430
wR_2 [all data]	0.1603	0.1394	0.1165
Max. / min. residual electron density / e Å^{-3}	0.702 / –0.605	0.543, –0.369	0.406 / –0.275

Table S2. Continued.

Compound	**2**[a]	**2**[b]	**4**
CCDC number	2440920	2440921	2440922
Empirical formula	$C_{72}H_{94}B_2P_2$	$C_{72}H_{94}B_2P_2$	$C_{138}H_{182}B_4P_4$
M_r	1043.03	1043.11	2008.11
T / K	101(2)	175(2)	175(2)
Radiation, λ / Å	Mo-K_α, 0.71073	electron, 0.0251	electron, 0.0251
Crystal size / mm³	0.303 × 0.244 × 0.136	< 0.001 x 0.001 x 0.001	< 0.001 x 0.001 x 0.001
Crystal color, habit	yellow block	yellow plates	orange plates / needles
Crystal system	orthorhombic	monoclinic	triclinic
Space group	*Pbca*	*P*2/*c*	*P*
a / Å	13.042(2)	16.54(19)	10.411(2)
b / Å	18.953(6)	10.99(12)	17.522(5)
c / Å	25.540(5)	18.22(17)	19.215(4)
α / °	90	90	115.36(2)
β / °	90	108.1(6)	90.684(19)
γ / °	90	90	98.32(2)
Volume / Å^3	6313(3)	3149(59)	3123.5(14)
Z	4	2	1
ρ_{calc} / g cm^{-3}	1.097	1.100	1.068
μ / mm^{-1}	0.109	0.000	0.000
F(000)	2264	494	475
θ range / °	1.595 – 26.430	0.046 – 0.866	0.042 – 0.866
Completeness	0.999	1.00	0.891
Reflections collected	83232	71722	53796
Unique reflections: [all data] / [I ≥ 2σ(I)]	6483 / 5074	5761 / 3664	10201 / 2126
R_{int}	0.0735	0.2426	0.3301
Parameters / restraints	355 / 0	356 / 228	608 / 757
GooF on F^2	1.023	1.466	1.255
R_1 [I ≥ 2σ(I)]	0.0490	0.1652	0.2194
wR_2 [all data]	0.1297	0.4440	0.5438
Max. / min. residual electron density / e Å^{-3}	0.473 / –0.309	0.352 / –0.193	0.234 / –0.126

Table S2. Continued.

Compound	**[*p*-Ph(PMes*H)$_2$], 7H**	**8**
CCDC number	2440923	2440924
Empirical formula	$C_{42}H_{64}P_2$	$C_{57}H_{79}BP_2$
M_r	630.87	836.95
T / K	100(2)	100(2)
Radiation, λ / Å	Mo-K$_\alpha$, 0.71073	Mo-K$_\alpha$, 0.71073
Crystal size / mm³	0.313×0.184×0.152	0.284 × 0.263 × 0.112
Crystal color, habit	orange block	yellow block
Crystal system	triclinic	triclinic
Space group	*P*	*P*
a / Å	10.104(2)	11.420(4)
b / Å	10.147(2)	14.100(5)
c / Å	19.653(4)	16.219(5)
α / °	79.722(9)	88.275(10)
β / °	76.977(12)	89.050(12)
γ / °	84.219(11)	89.098(10)
Volume / Å^3	1927.7(7)	2609.7(15)
Z	2	2
ρ_{calc} / g cm^{-3}	1.087	1.065
μ / mm^{-1}	0.139	0.117
F(000)	692	912
θ range / °	2.044 – 28.425	1.886 – 26.506
Completeness	0.975	0.993
Reflections collected	40426	69613
Unique reflections: [all data] / [I ≥ 2σ(I)]	9481 / 6689	10750 / 8456
R_{int}	0.0779	0.0694
Parameters / restraints	428 / 4	570 / 2
GooF on F^2	1.031	1.051
R_1 [I ≥ 2σ(I)]	0.0567	0.0476
wR_2 [all data]	0.1507	0.1211
Max. / min. residual electron density / e Å^{-3}	0.581 / –0.444	0.271 / –0.269

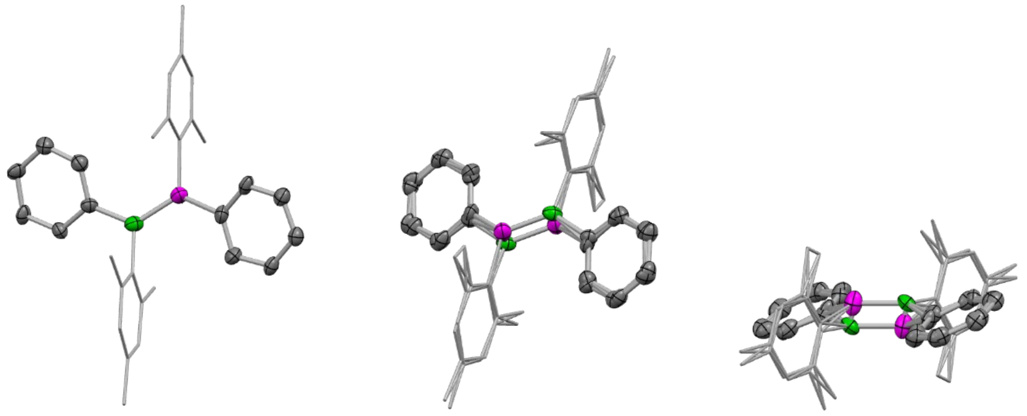

Figure S1. Three different projections of the solid-state molecular structure of **1**^{Mes} as determined from single-crystal X-ray diffraction at 100 K. H atoms were omitted for clarity. Element color code: carbon (grey), boron (green), phosphorus (pink). All ellipsoids are drawn at the 50% probability level. The whole molecule is disordered by inversion symmetry and the disorder is shown in the middle and right drawings.

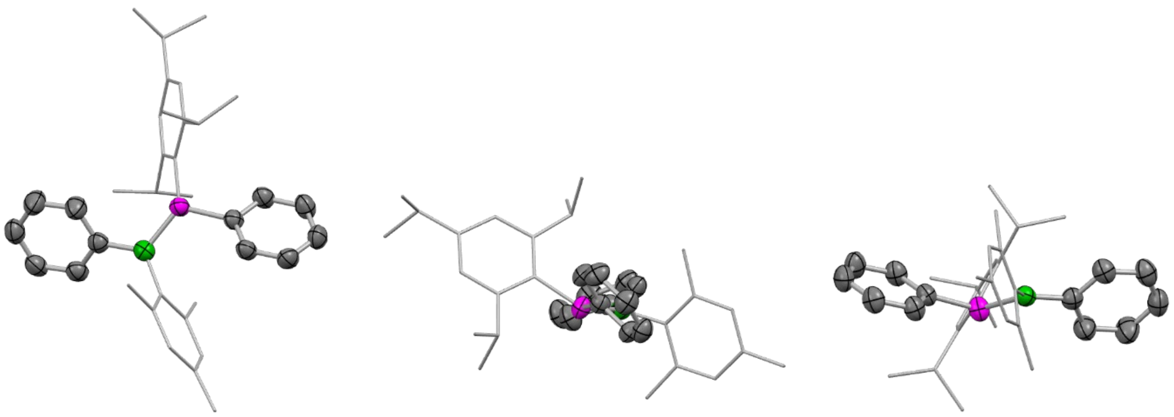

Figure S2. Three different projections of the solid-state molecular structure of **1**^{Tip} as determined from single-crystal X-ray diffraction at 100 K. H atoms were omitted for clarity. Element color code: carbon (grey), boron (green), phosphorus (pink). All ellipsoids are drawn at the 50% probability level.

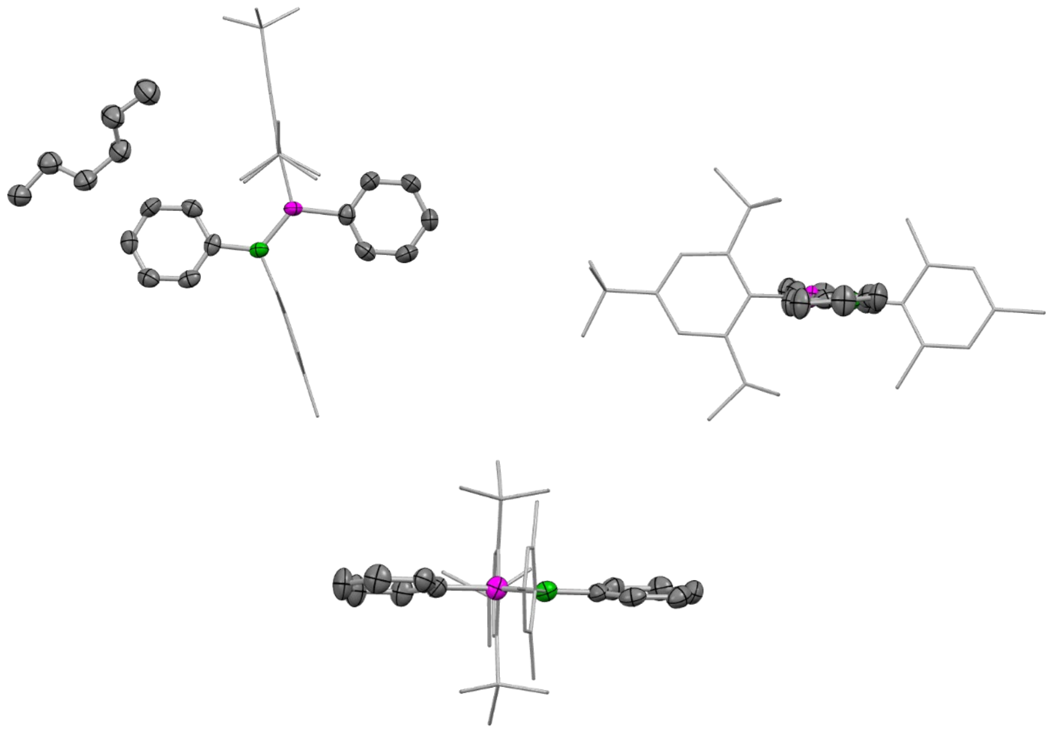

Figure S3. Three different projections of the solid-state molecular structure of **1**$^{\mathbf{Mes*}}$ as determined from single-crystal X-ray diffraction at 100 K. H atoms were omitted for clarity. The whole molecule of **1**$^{\mathbf{Mes*}}$ is disordered with an occupancy of ca. 12 % for the second part. The left top projection also shows *n*-hexane that co-crystallized with severe disorder. Only the major parts of the molecules are shown here. Element color code: carbon (grey), boron (green), phosphorus (pink). All ellipsoids are drawn at the 50% probability level.

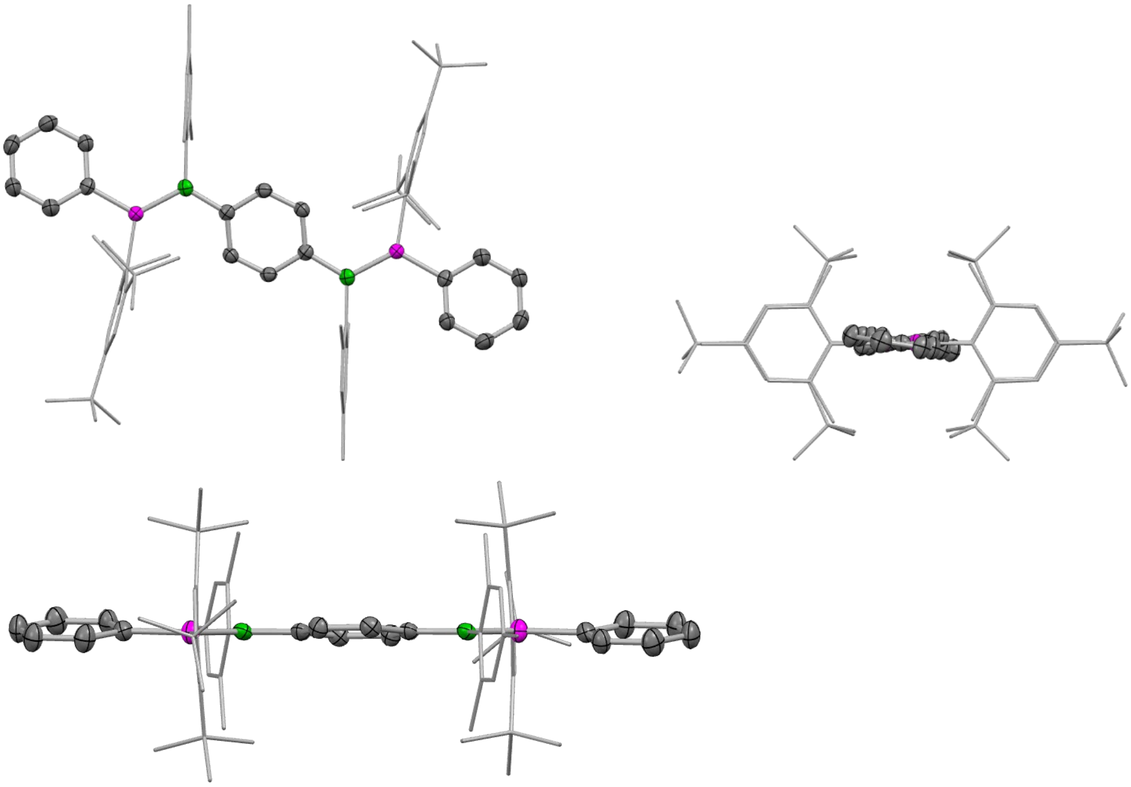

Figure S4. Three different projections of the solid-state molecular structure of **2**[a] as determined from single-crystal X-ray diffraction at 100 K. H atoms were omitted for clarity. The molecule has inversion symmetry. Element color code: carbon (grey), boron (green), phosphorus (pink). All ellipsoids are drawn at the 50% probability level.

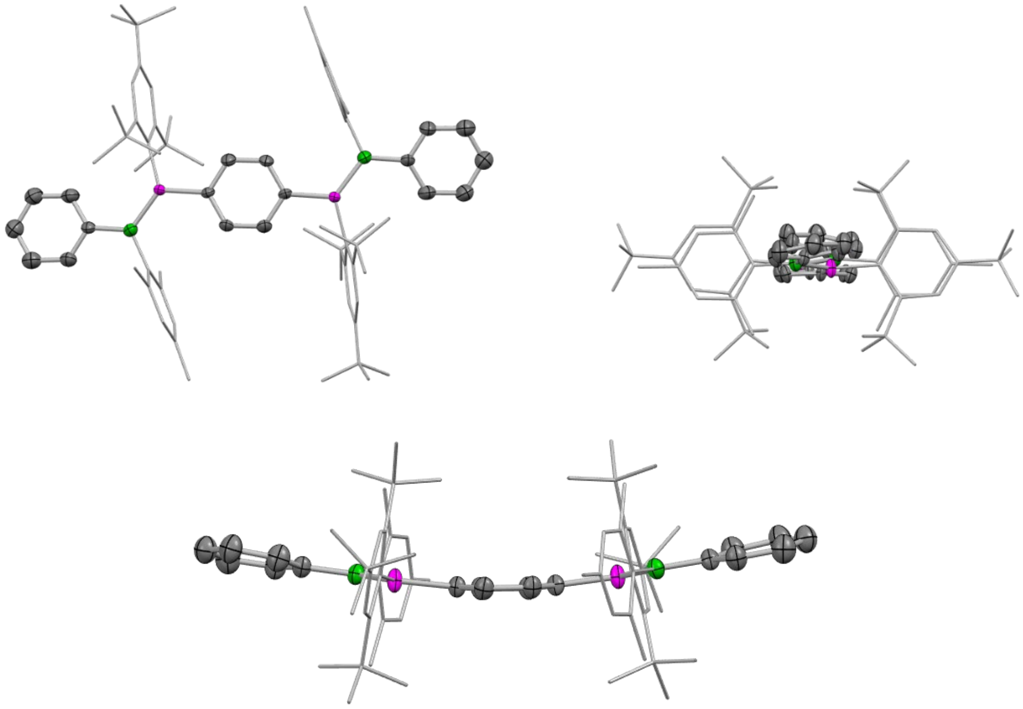

Figure S5. Three different projections of the solid-state molecular structure of **2**[b] as determined from 3D ED at 175 K. H atoms were omitted for clarity. The molecule has 2-fold rotational symmetry. Element color code: carbon (grey), boron (green), phosphorus (pink). All ellipsoids are drawn at the 50% probability level.

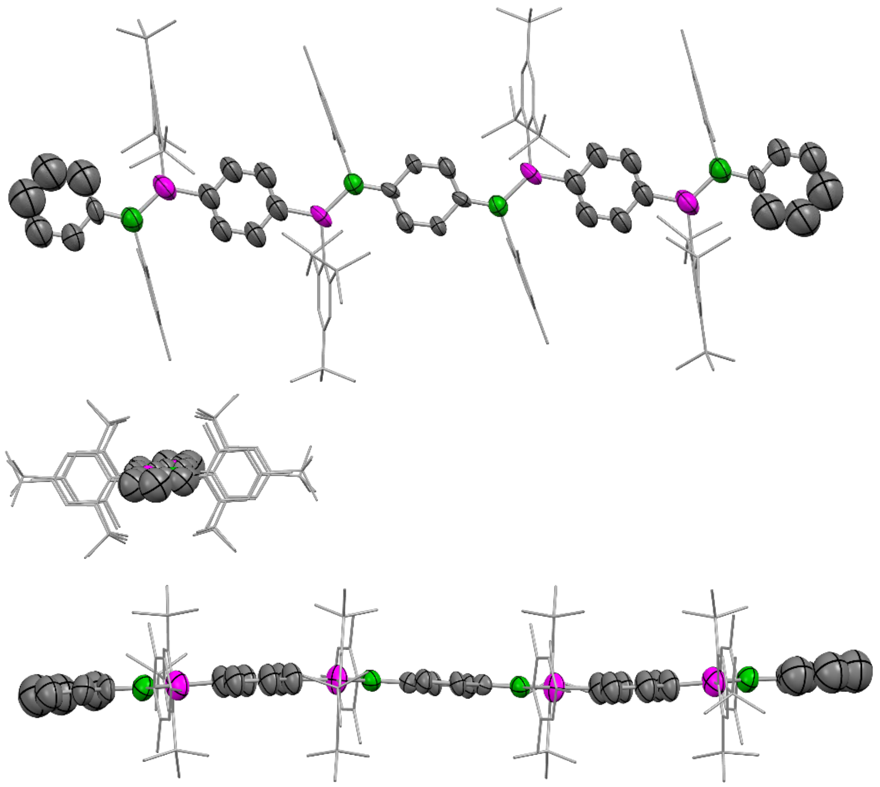

Figure S6. Three different projections of the solid-state molecular structure of **4** as determined from 3D ED at 175 K. H atoms were omitted for clarity. The molecule has inversion symmetry. Element color code: carbon (grey), boron (green), phosphorus (pink). All ellipsoids are drawn at the 50% probability level.

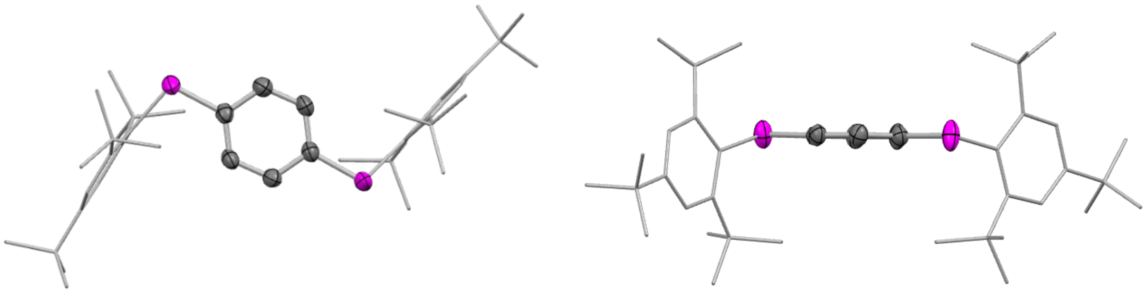

Figure S7. Two different projections of the solid-state molecular structure of **7H** as determined from single-crystal X-ray diffraction at 100 K. H atoms were omitted for clarity. Element color code: carbon (grey), boron (green), phosphorus (pink). All ellipsoids are drawn at the 50% probability level.

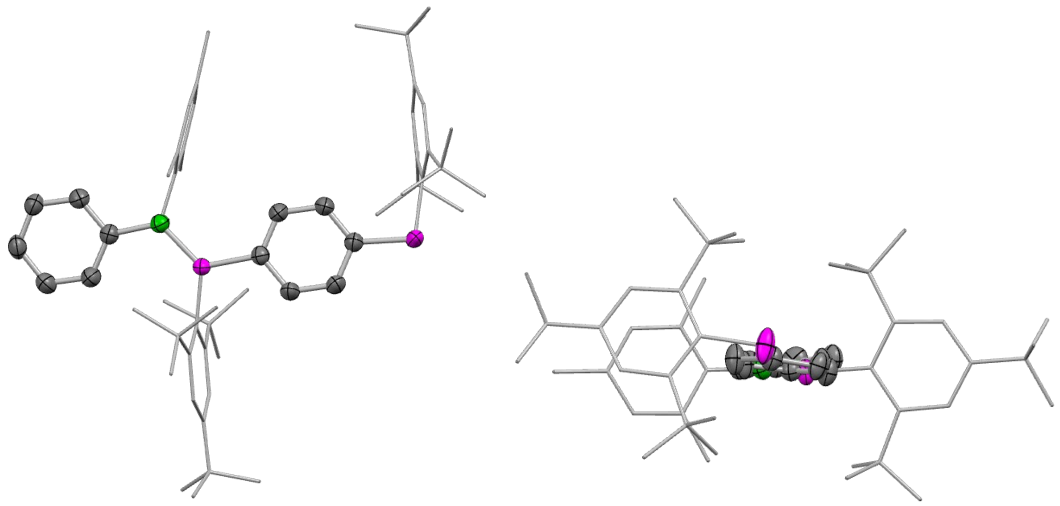

Figure S8. Two different projections of the solid-state molecular structure of **8** as determined from single-crystal X-ray diffraction at 100 K. H atoms were omitted for clarity. Element color code: carbon (grey), boron (green), phosphorus (pink). All ellipsoids are drawn at the 50% probability level.

Table S3. Selected bond lengths (Å), bond angles (°), and interplanar angles (°) derived from SXRD and 3D ED data.

Compound	**1**$^{Mes\ (a)}$	**1**^{Tip}	**1**$^{Mes*\ (b)}$	**2**$^{a\ (c)}$	**2**$^{b\ (d)}$	**4** $^{(c)}$
B1–P1	1.741(4)	1.839(2)	1.8136(15)	1.811(2)	1.83(2)	1.840(13)
B2–P2						1.848(12)
Σ(RB1R)	359.4(5)	359.3(2)	359.72(8)	360.0(1)	360.0(9)	360.0(12)
Σ(RB2R)						360.0(11)
Σ(RP1R)	353.0(4)	347.4(1)	359.9(1)	360.0(1)	359.7(9)	359.8(9)
Σ(RP2R)						359.8(8)
∠Ph-Ph[°]	2.3(5)	75.46(7)	13.63(6)	0.0	25.4(5)	0.0(7)
∠Ph-phenylene				15.50(8)	12.7(3)	3.8(3), 8.0(4)
∠ phenylene-phenylene						5.3(3), 0.00(7)
∠Ph_P-C_2BPC_2	21.5(4)	32.81(6)	7.72(5)	9.23(6)		
∠Ph_B-C_2BPC_2	20.1(3)	43.50(6)	6.27(5)		6.8(2)	4.6(3)
∠ phenylene-C_2BPC_2				6.32(7)	8.5(2)	2.11(9), 4.9(2), 4.0(3)
∠BR_3-Mes	87.3(3)	81.86(6)	89.30(6)	85.59(6)	86.8(3)	89.0(3), 84.8(3)
∠PR_3-Mes/Tip/Mes*	83.7(3)	61.15(7)	88.97(7)	86.68(7)	86.8(4)	88.5(3), 89.1(3)

(a) The whole molecule is disordered by inversion.

(b) The whole molecule is disordered, and values are given for the major part (88 %).

(c) The molecule has inversion symmetry.

(d) The molecule has two-fold rotational symmetry.

1.4. NMR Spectra

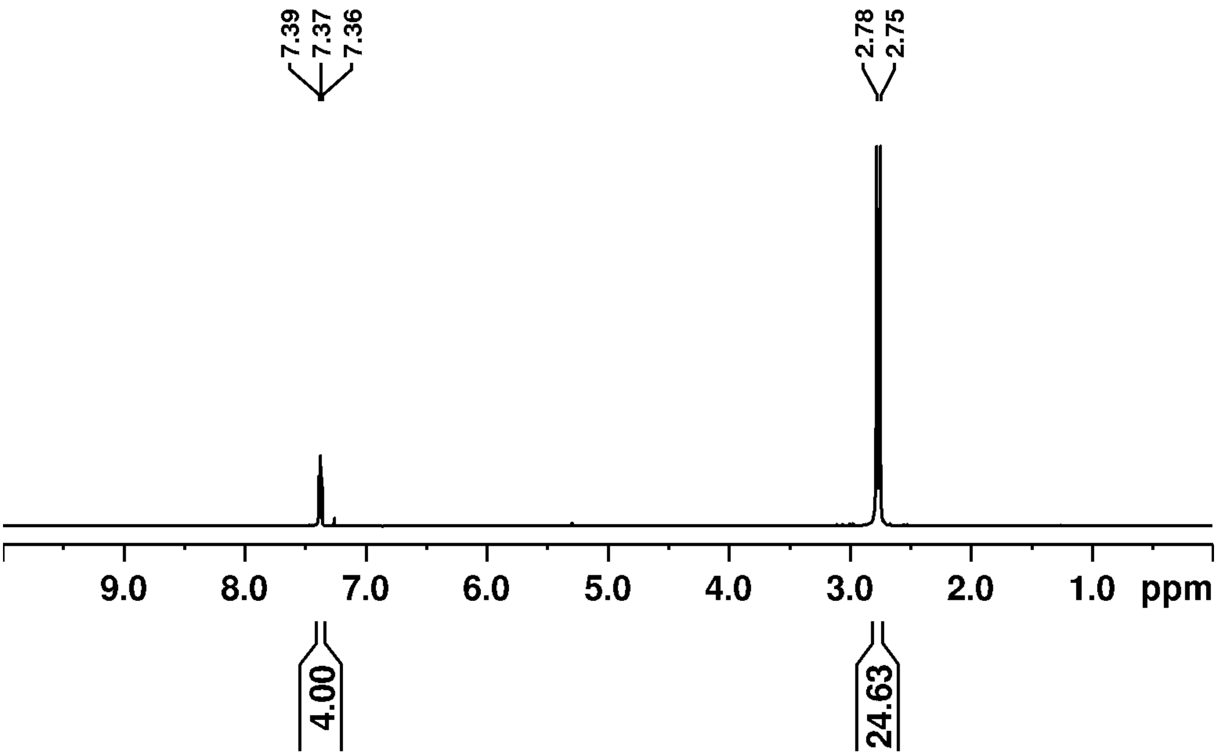

Figure S9. ^{1}H NMR spectrum of $Ph(P(NMe_2)_2)_2$ (300 MHz, $CDCl_3$).

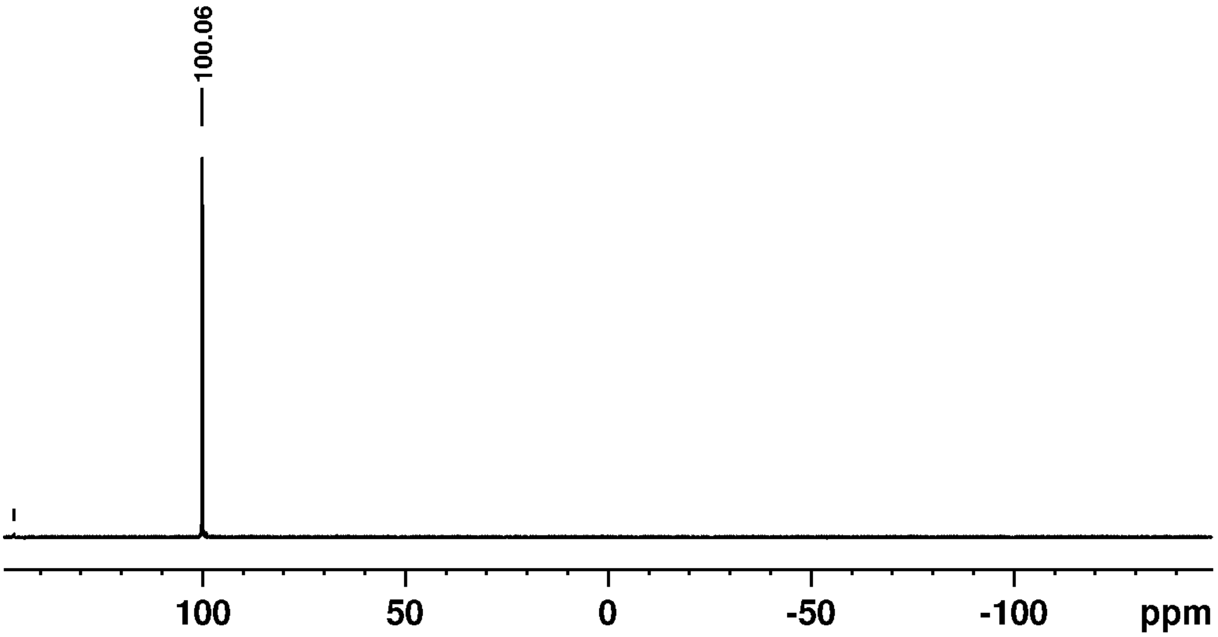

Figure S10. $^{31}P\{^{1}H\}$ spectrum of $Ph(P(NMe_2)_2)_2$ (162 MHz, $CDCl_3$).

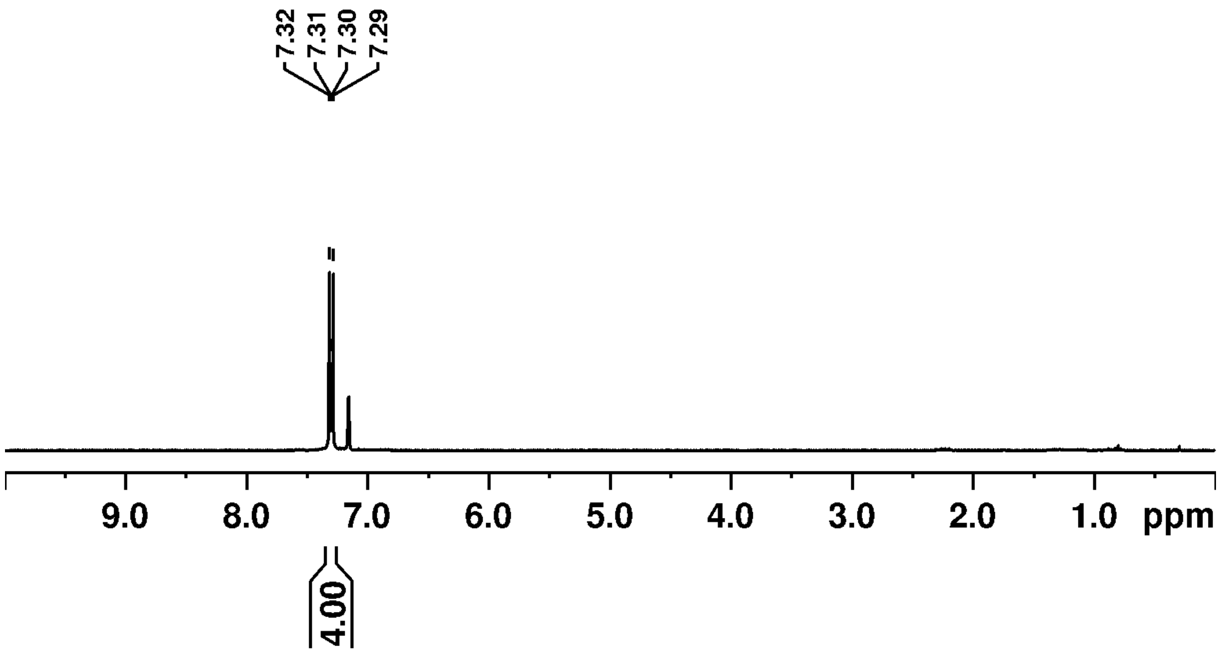

Figure S11. ^{1}H NMR spectrum of *p*-Ph(PCl_2)$_2$ (300 MHz, C_6D_6)

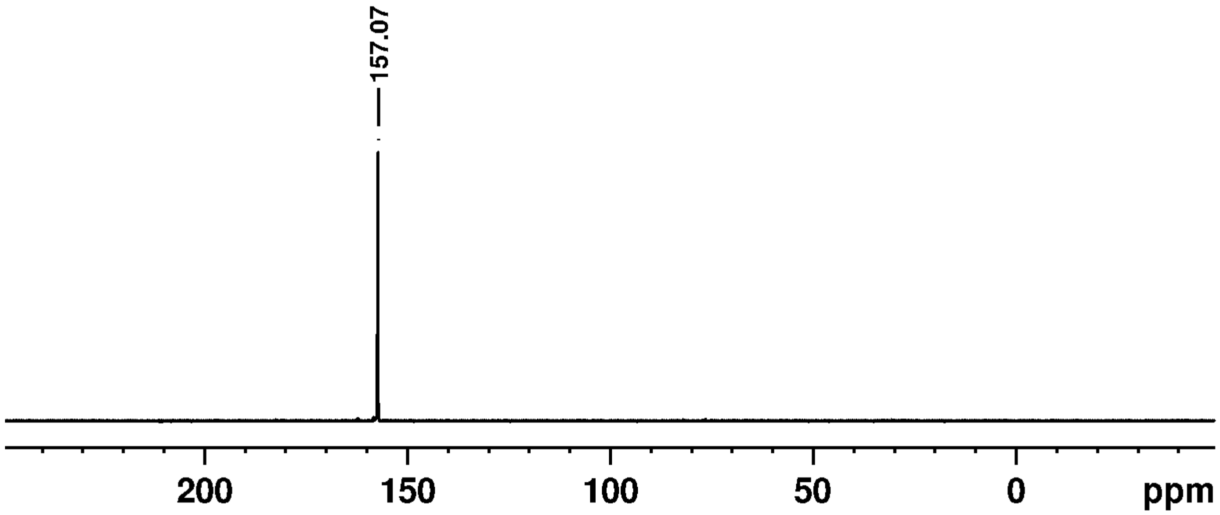

Figure S12. $^{31}P\{^1H\}$ spectrum of *p*-Ph(PCl_2)$_2$ (300 MHz, C_6D_6)

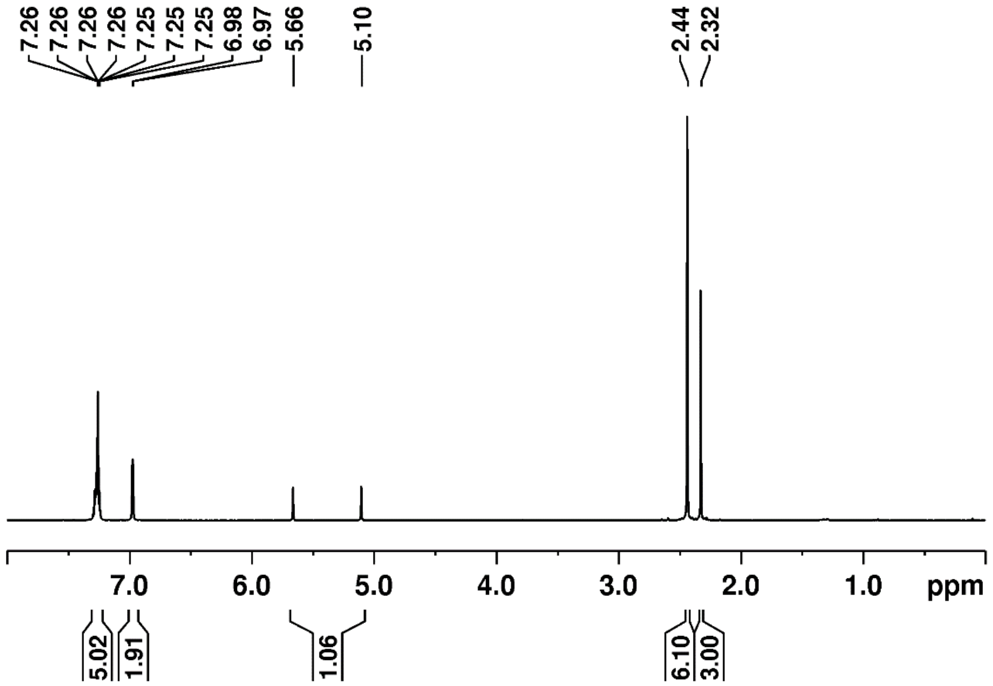

Figure S13. ^{1}H NMR spectrum of PhMesPH (400 MHz, $CDCl_3$).

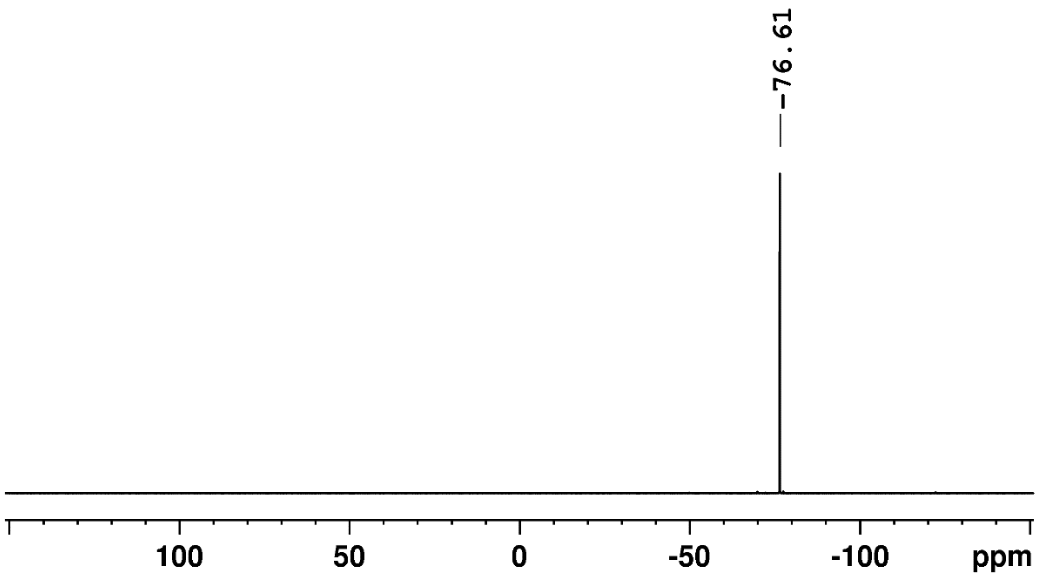

Figure S14. $^{31}P\{^{1}H\}$ spectrum of PhMesPH (162 MHz, $CDCl_3$).

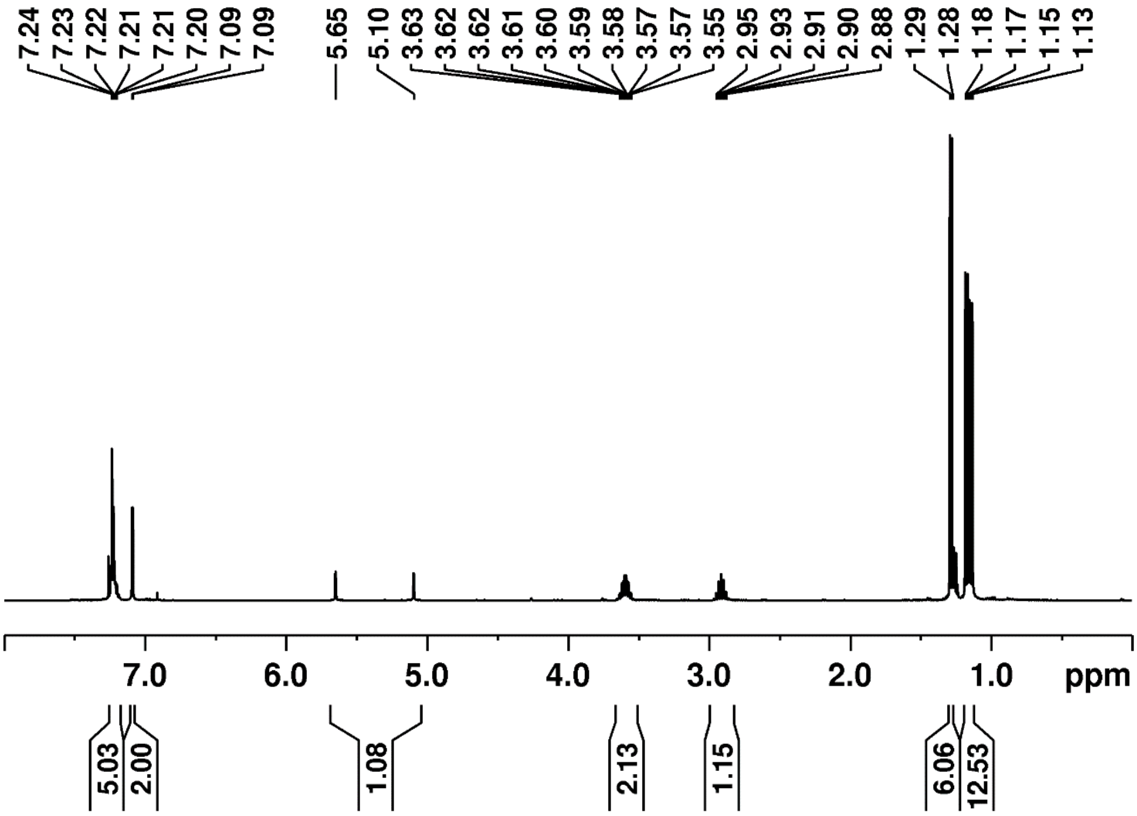

Figure S15. ^{1}H NMR spectrum of PhTipPH (400 MHz, $CDCl_3$).

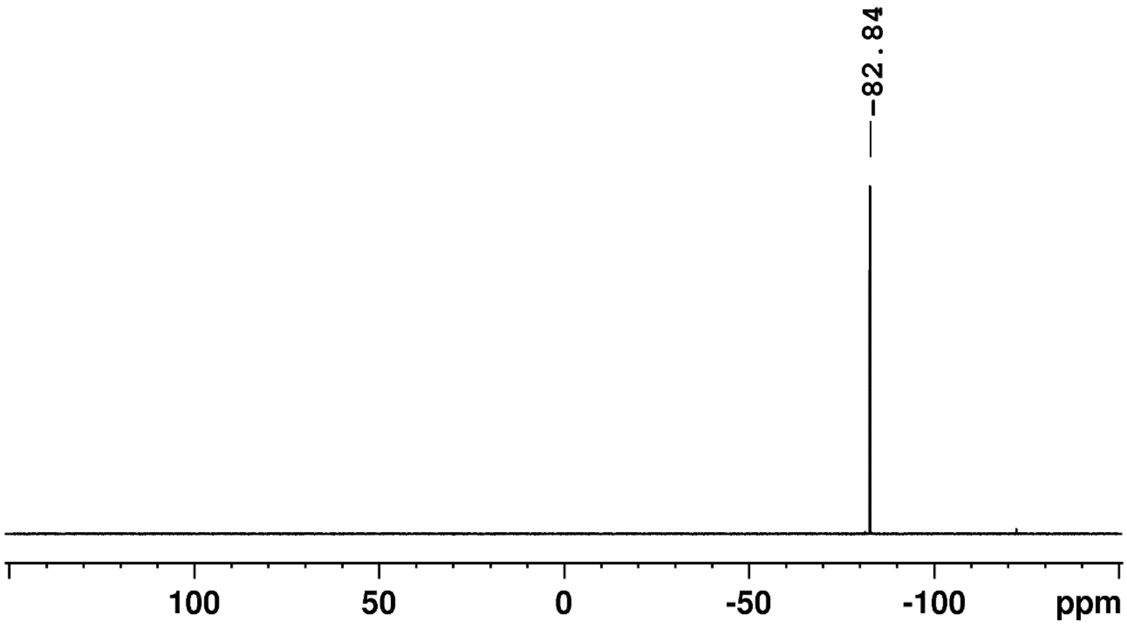

Figure S16. $^{31}P\{^{1}H\}$ spectrum of PhTipPH (162 MHz, $CDCl_3$).

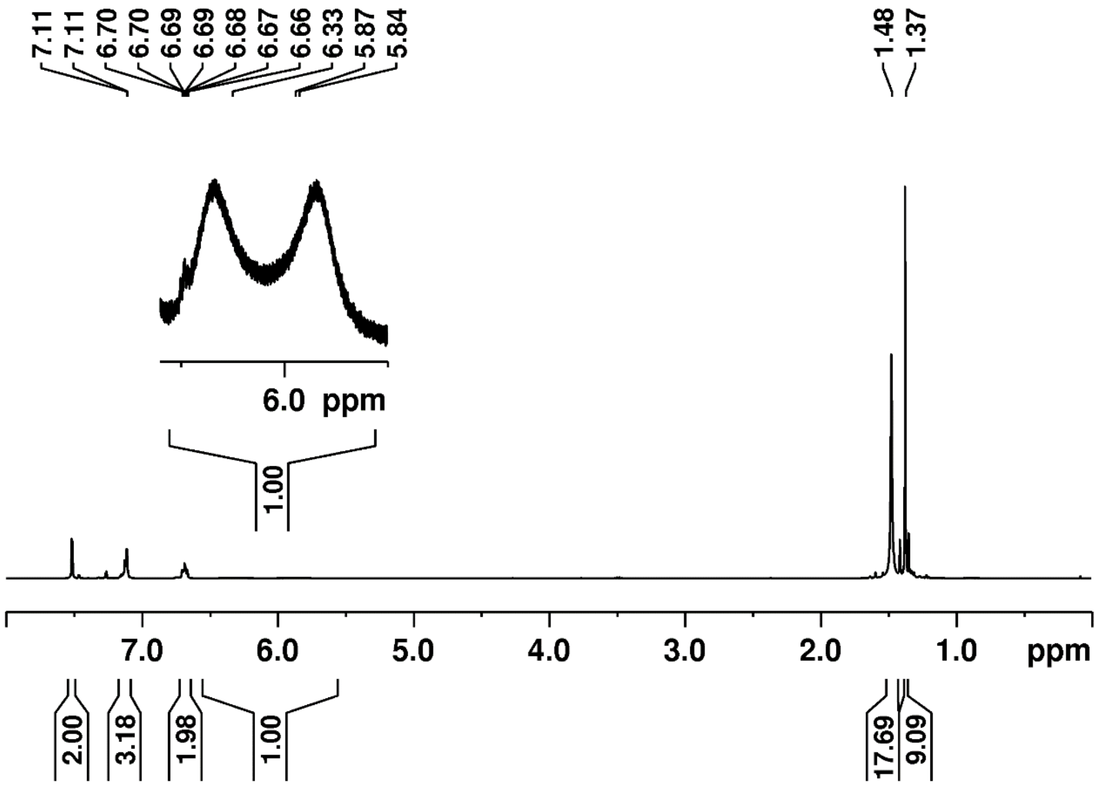

Figure S17. ^{1}H NMR spectrum of PhMes*PH (400 MHz, $CDCl_3$).

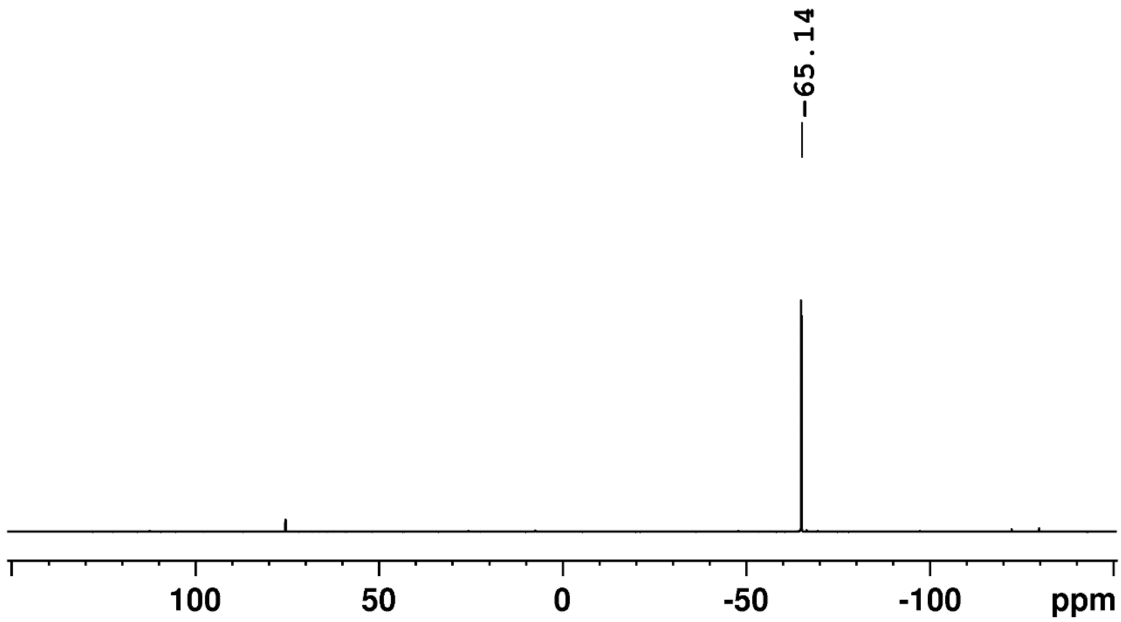

Figure S18. ^{31}P{^{1}H} spectrum of PhMes*PH (162 MHz, $CDCl_3$).

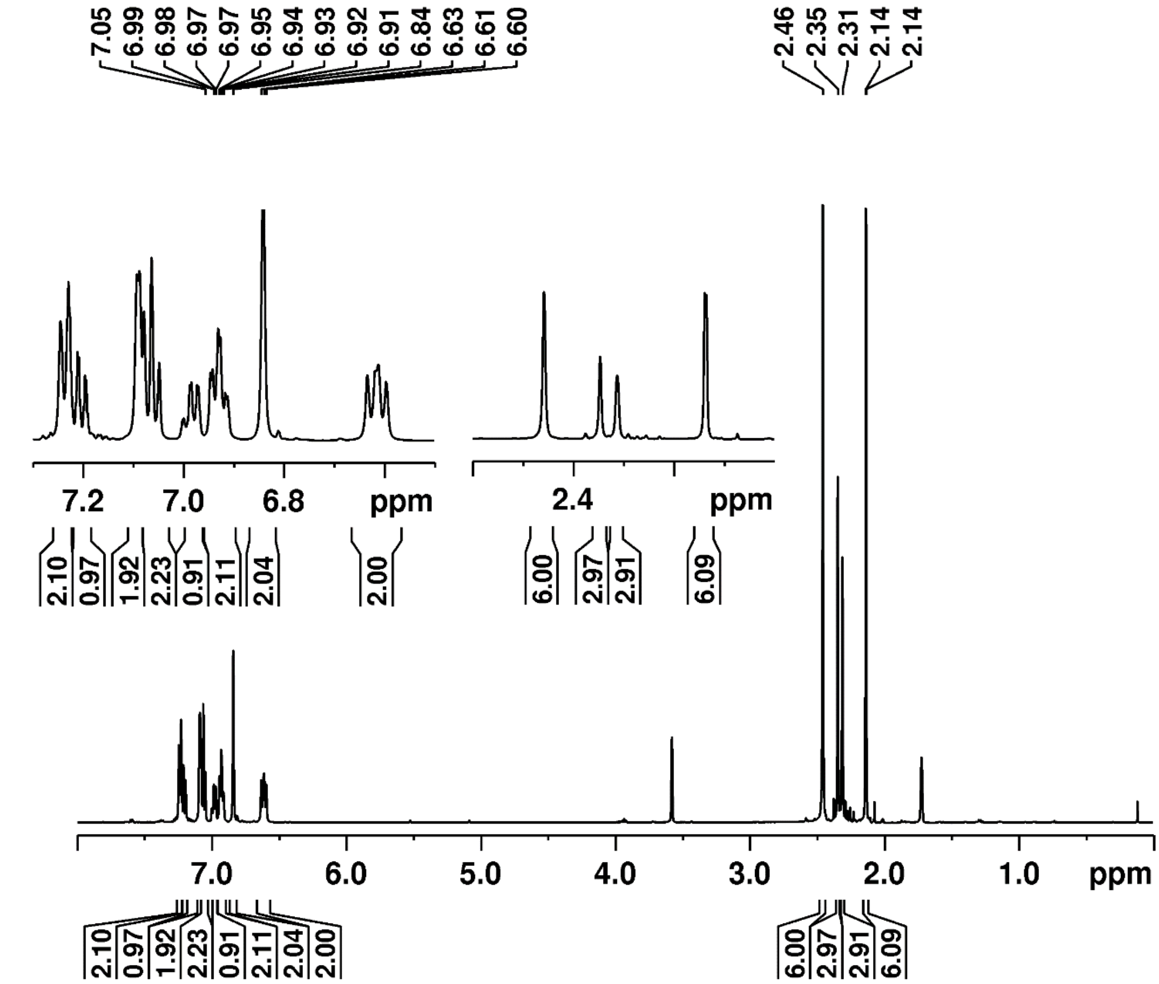

Figure S19. ^{1}H NMR spectrum of MesBPMes (**1^{Mes}**) (500 MHz, THF-d8).

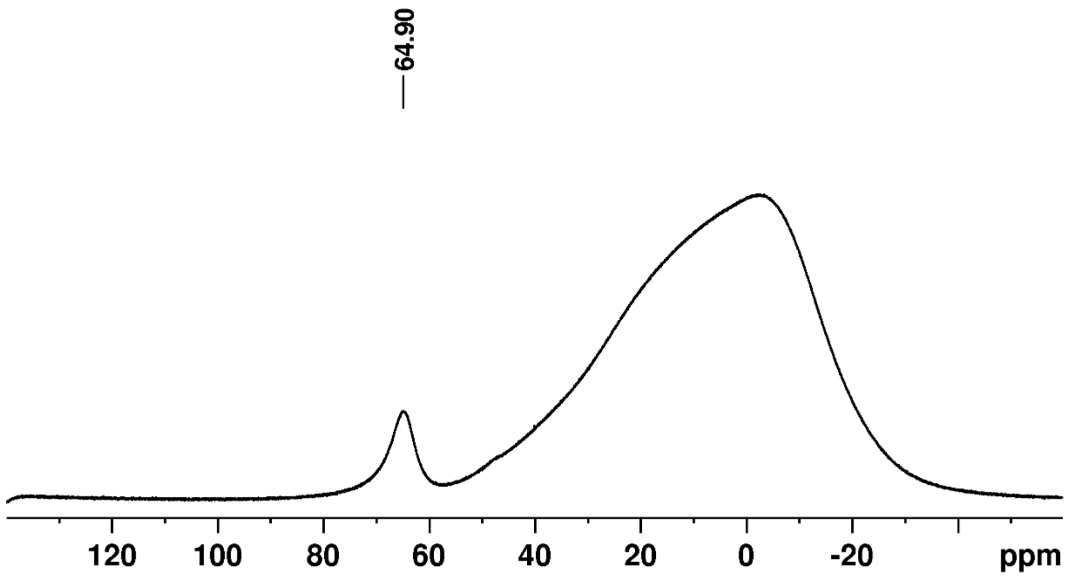

Figure S20. $^{11}B\{^{1}H\}$ NMR spectrum of MesBPMes (**1^{Mes}**) (160 MHz, THF-d8).

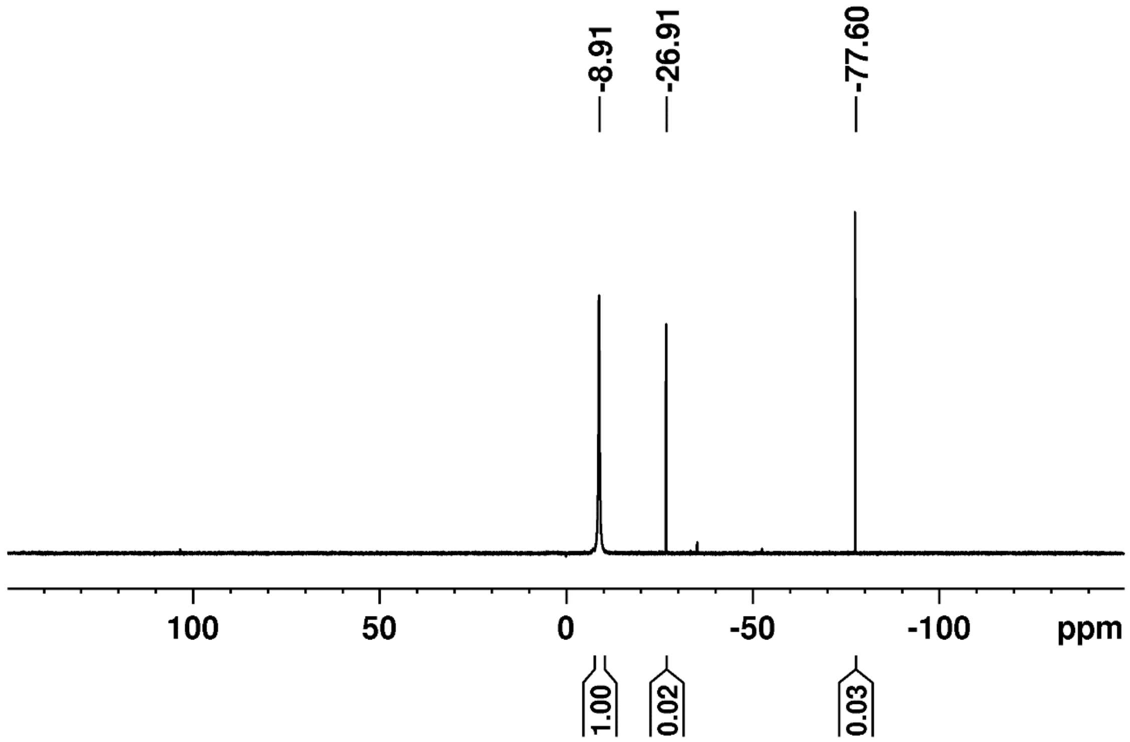

Figure S21. $^{31}P\{^{1}H\}$ NMR spectrum of MesBPMes (**1^Mes^**) (202 MHz, THF-d8) Small impurities of protonated starting material (**5^Mes^**) at 77.60 ppm.

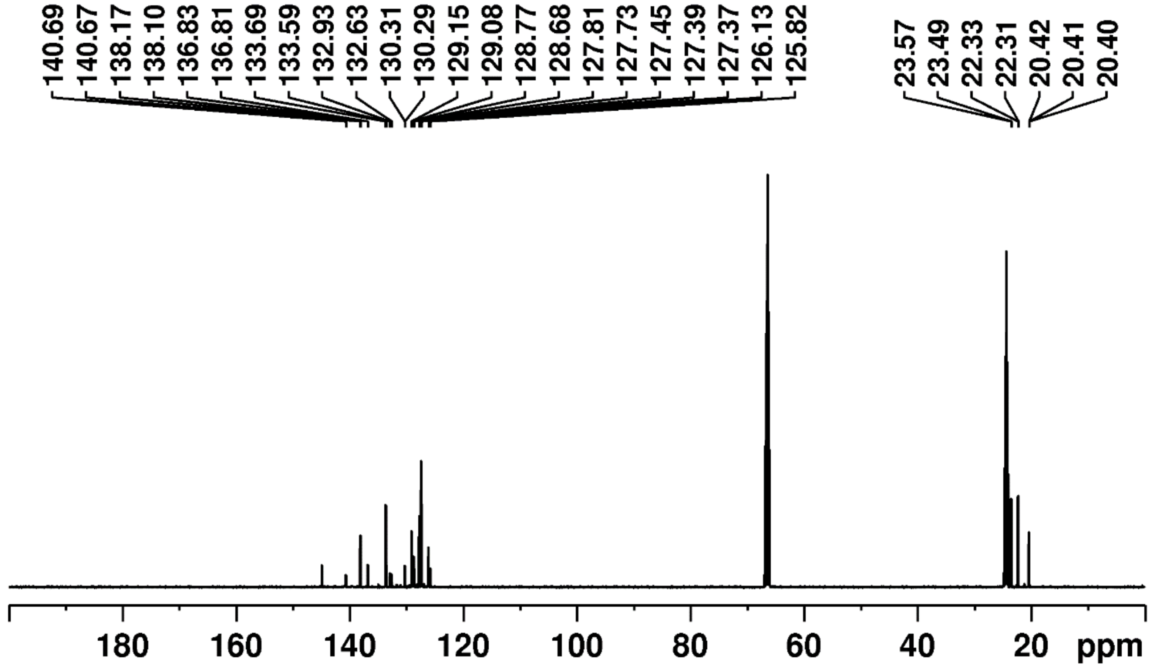

Figure S22. $^{13}C\{^{1}H\}$ NMR spectrum of MesBPMes (**1^Mes^**) (126 MHz, THF-d8).

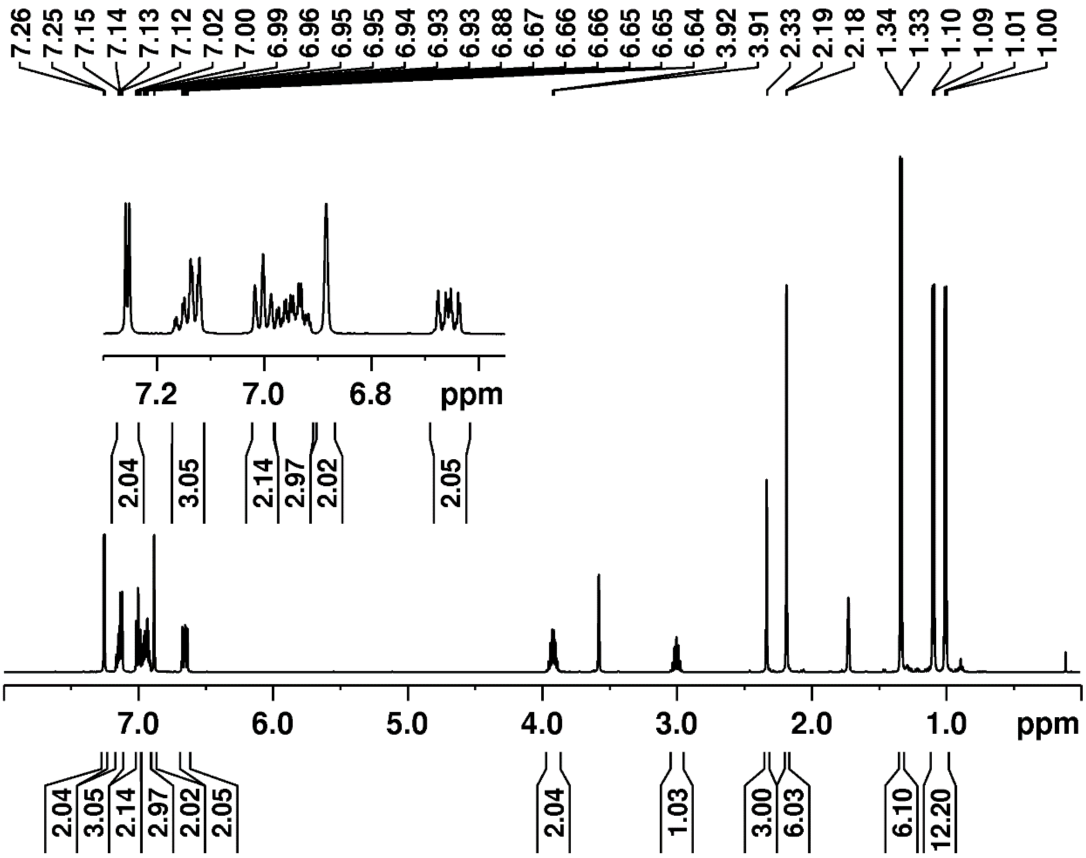

Figure S23. ^{1}H NMR spectrum of MesBPTip (1^{Tip}) (500 MHz, THF-d8).

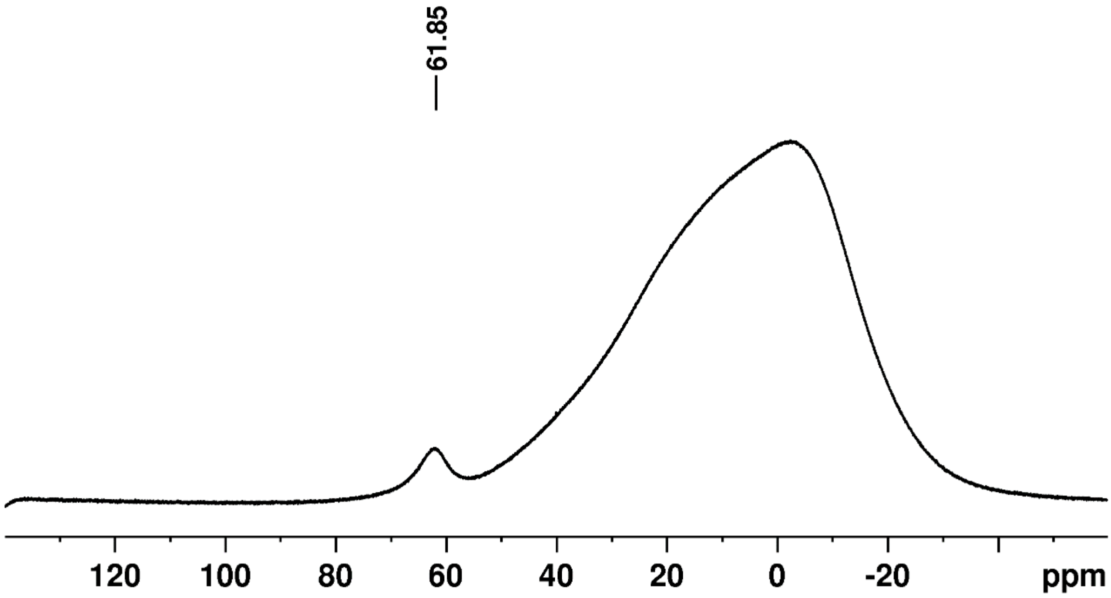

Figure S24. ^{11}B{^{1}H} NMR spectrum of MesBPTip (1^{Tip}) (160 MHz, THF-d8).

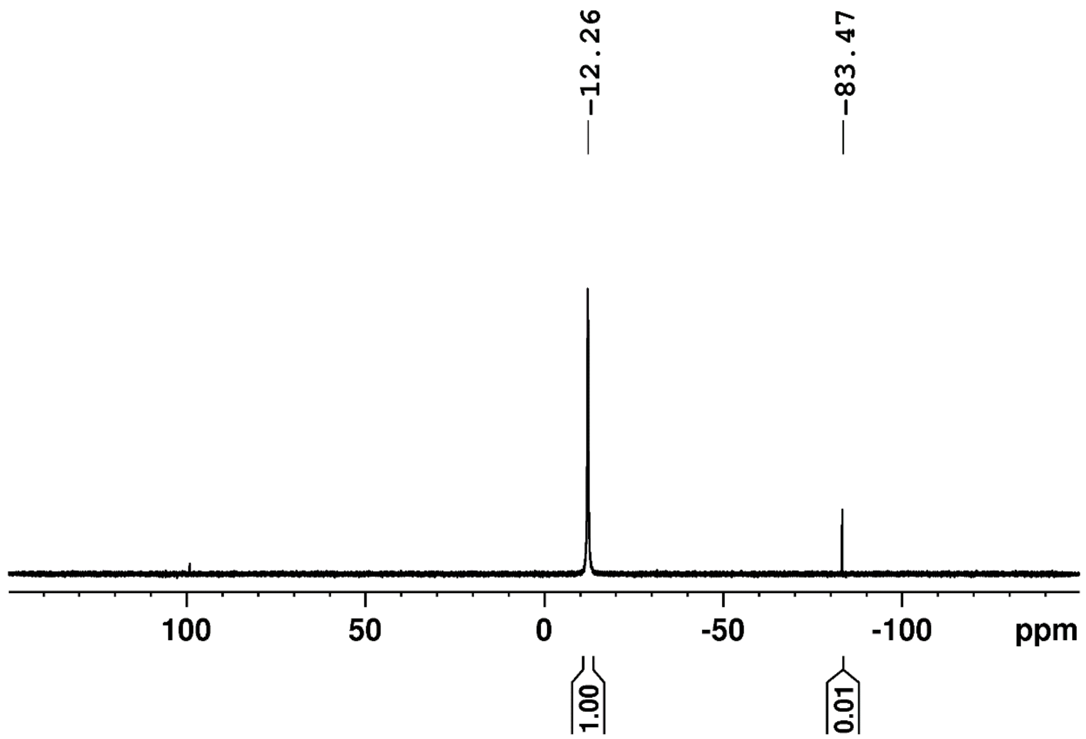

Figure S25. $^{31}P\{^1H\}$ NMR spectrum of MesBPTip (**1^{Tip}**) with small amounts of starting material (**5^{Tip}**) at −83.47 ppm (202 MHz, THF-d8).

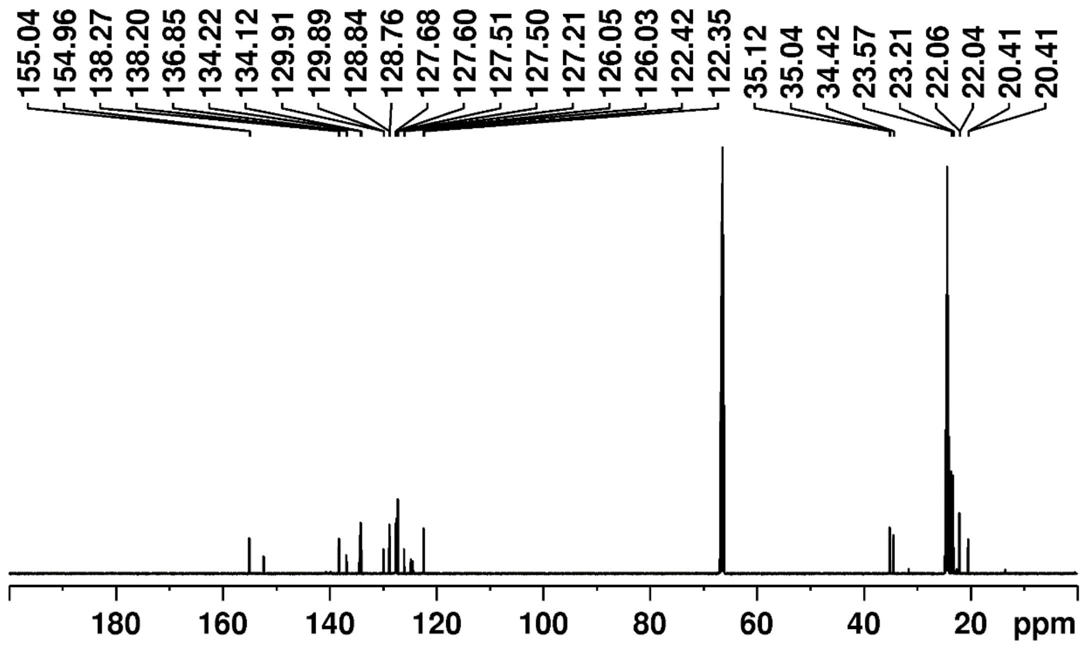

Figure S26. $^{13}C\{^1H\}$ NMR spectrum of MesBPTip (**1^{Tip}**) (126 MHz, THF-d8).

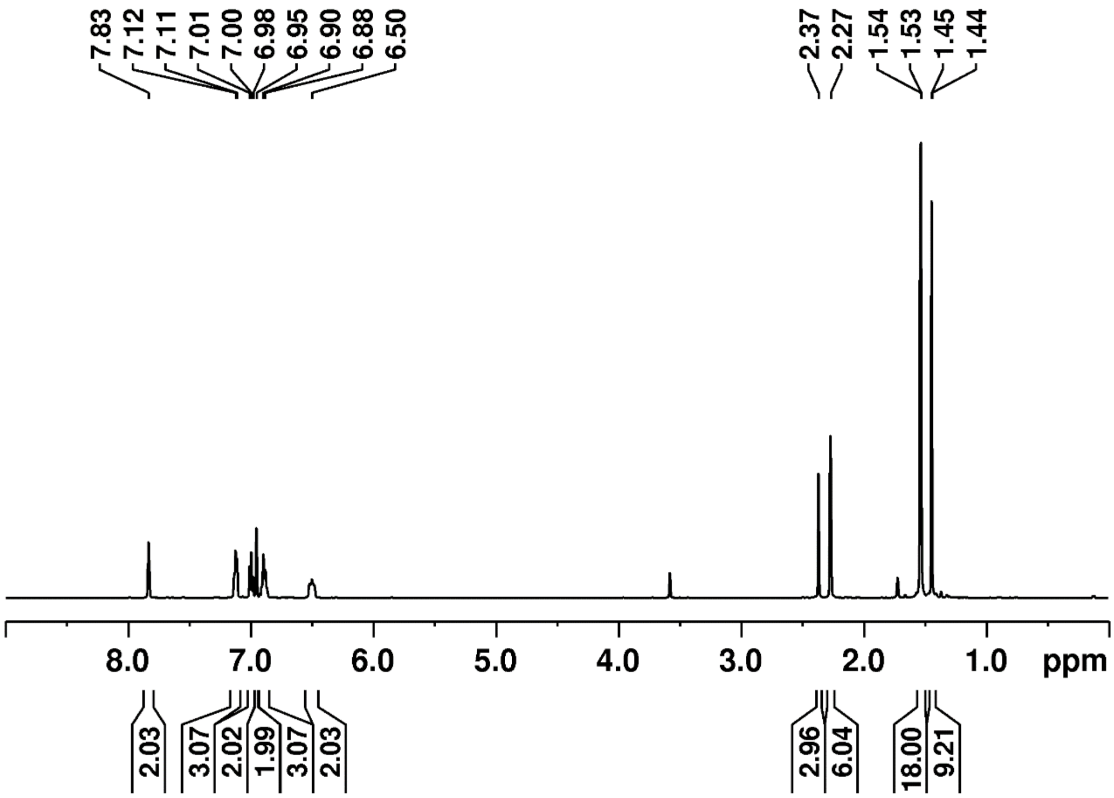

Figure S27. ^{1}H NMR spectrum of MesBPMes* (**1**$^{\mathbf{Mes*}}$) (500 MHz, THF-d8).

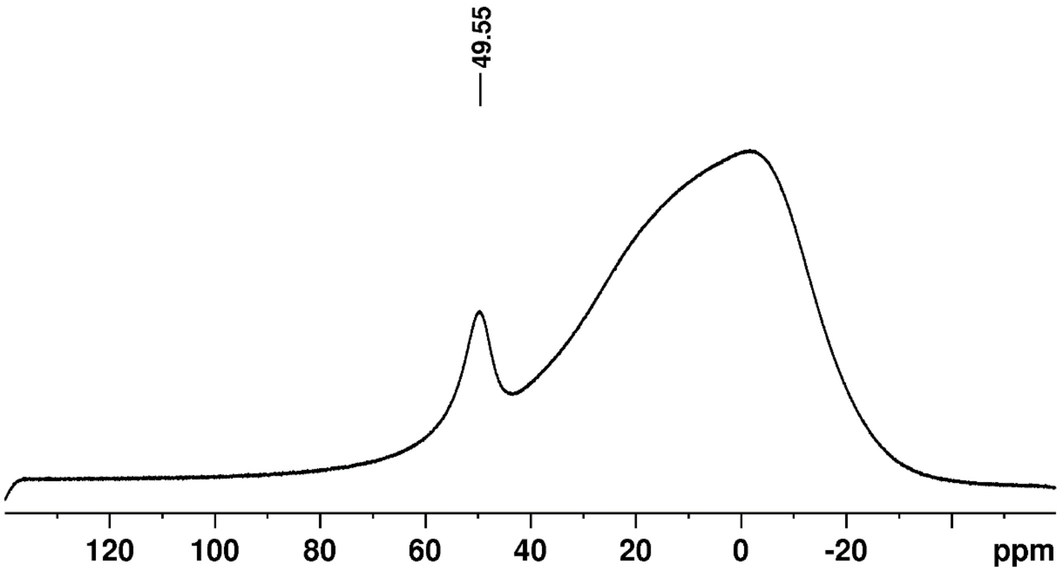

Figure S28. ^{11}B{^{1}H} NMR spectrum of MesBPMes* (**1**$^{\mathbf{Mes*}}$) (160 MHz, THF-d8).

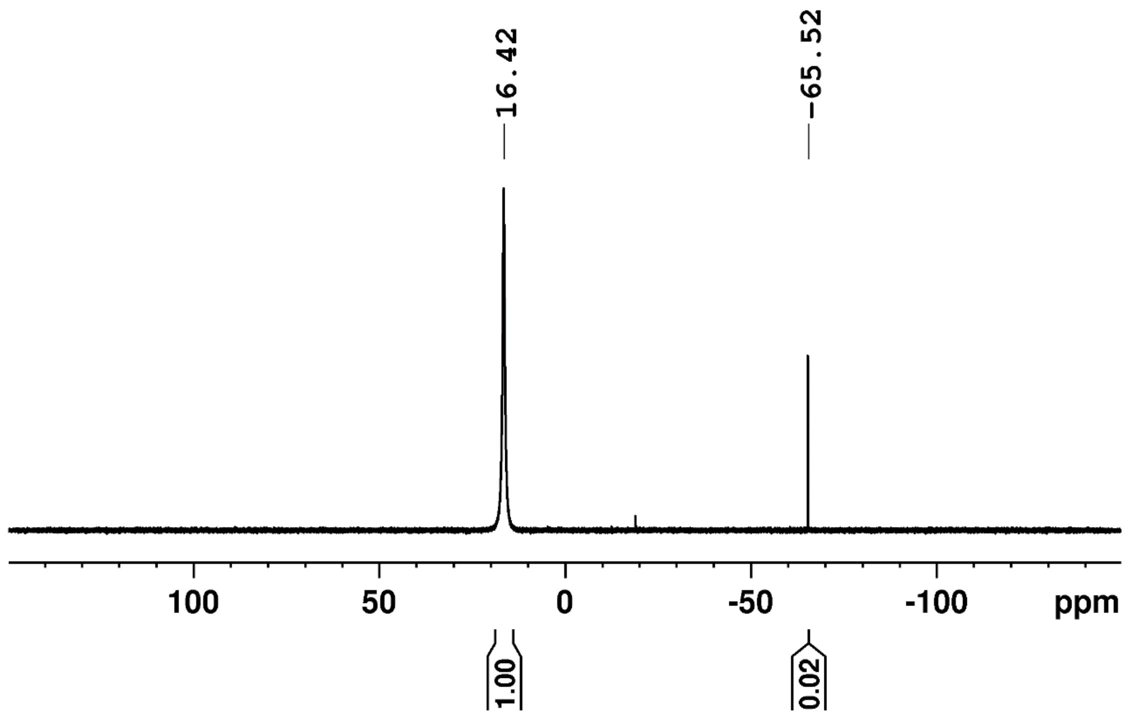

Figure S29. $^{31}P\{^1H\}$ NMR spectrum of MesBPMes* (**1**^{Mes*}) (202 MHz, THF-d8). Small Impurities of starting material (**5**^{Mes*}) at 65.52 ppm.

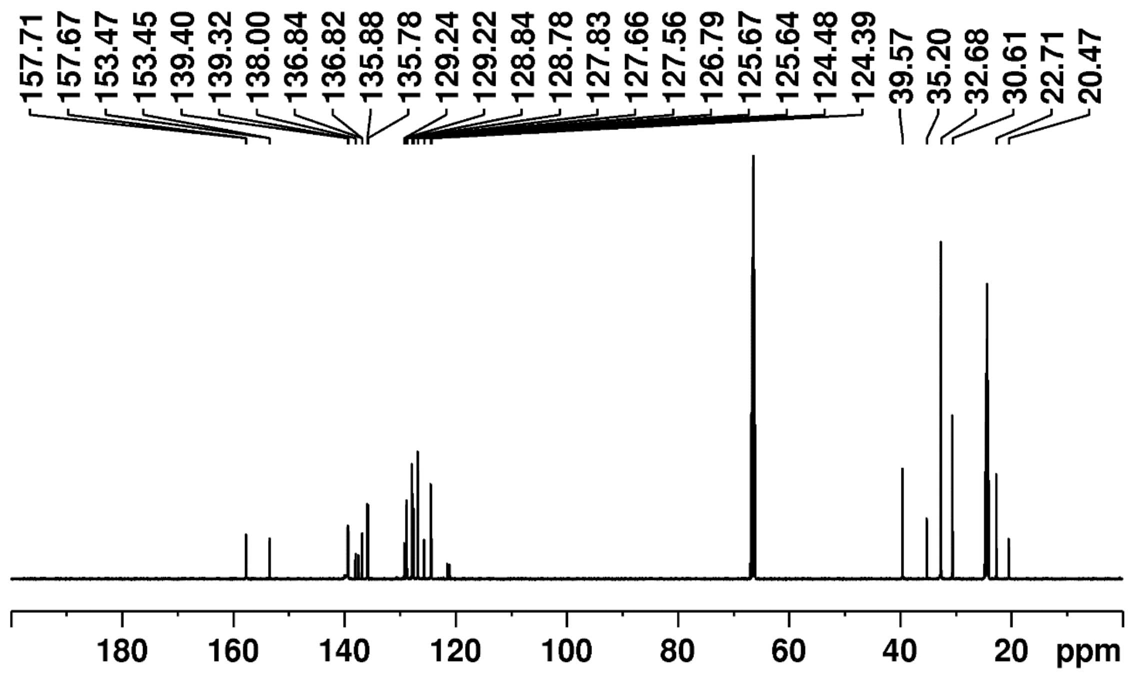

Figure S30. $^{13}C\{^1H\}$ NMR spectrum of MesBPMes* (**1**^{Mes*}) (126 MHz, THF-d8).

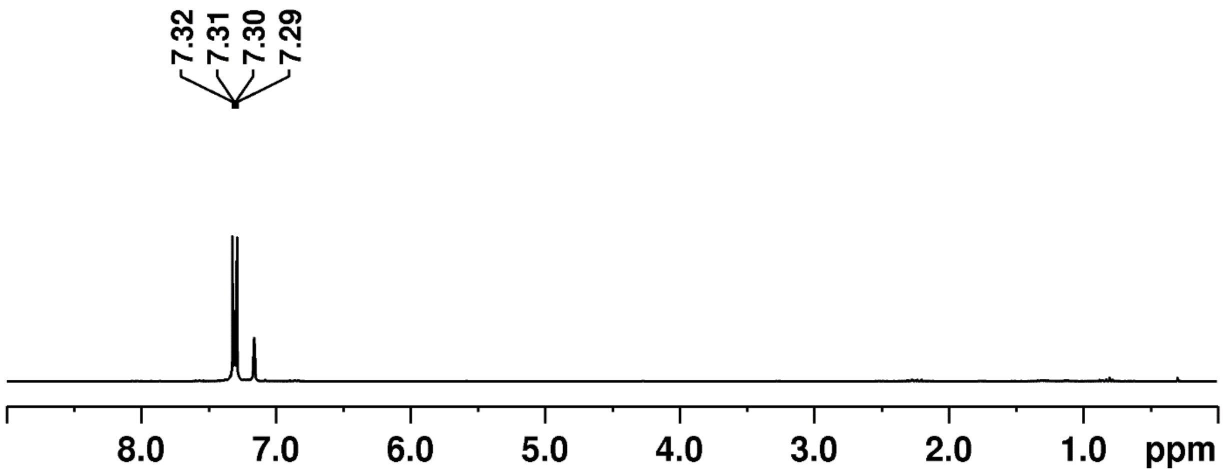

Figure S31. ^{1}H NMR spectrum of *p*-Ph(PCl_2)$_2$ (**1**$^{\mathbf{Mes*}}$) (300 MHz, C_6D_6).

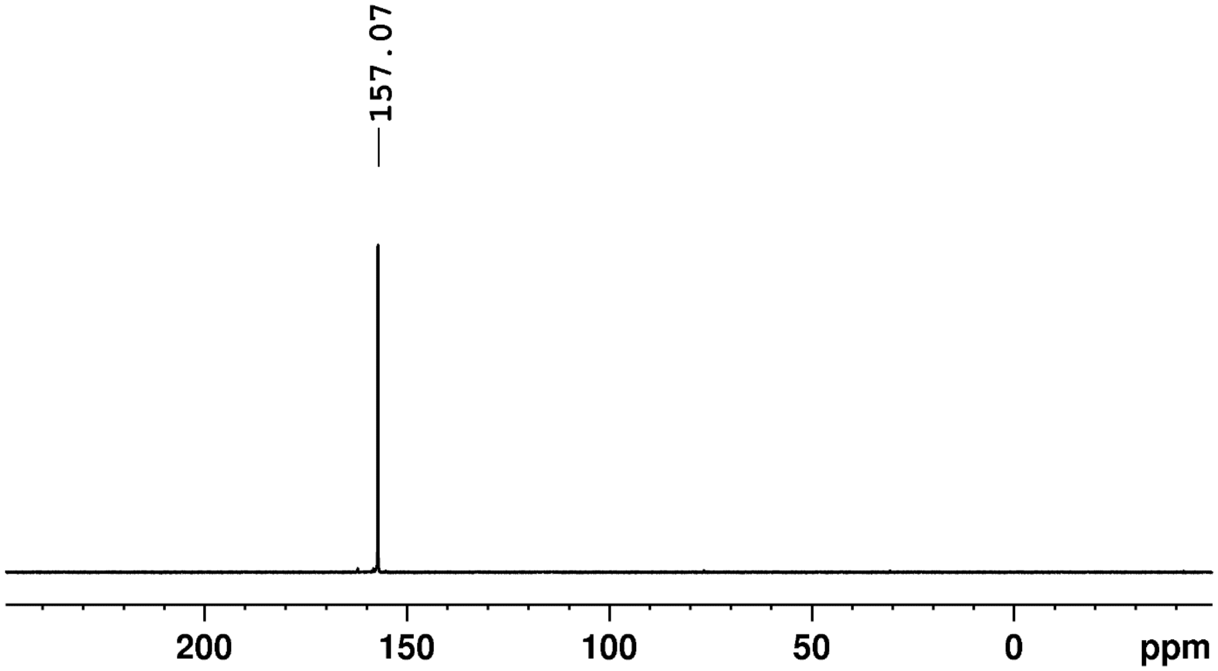

Figure S32. ^{31}P{^{1}H} NMR spectrum of *p*-Ph(PCl_2)$_2$ (122 MHz, C_6D_6).

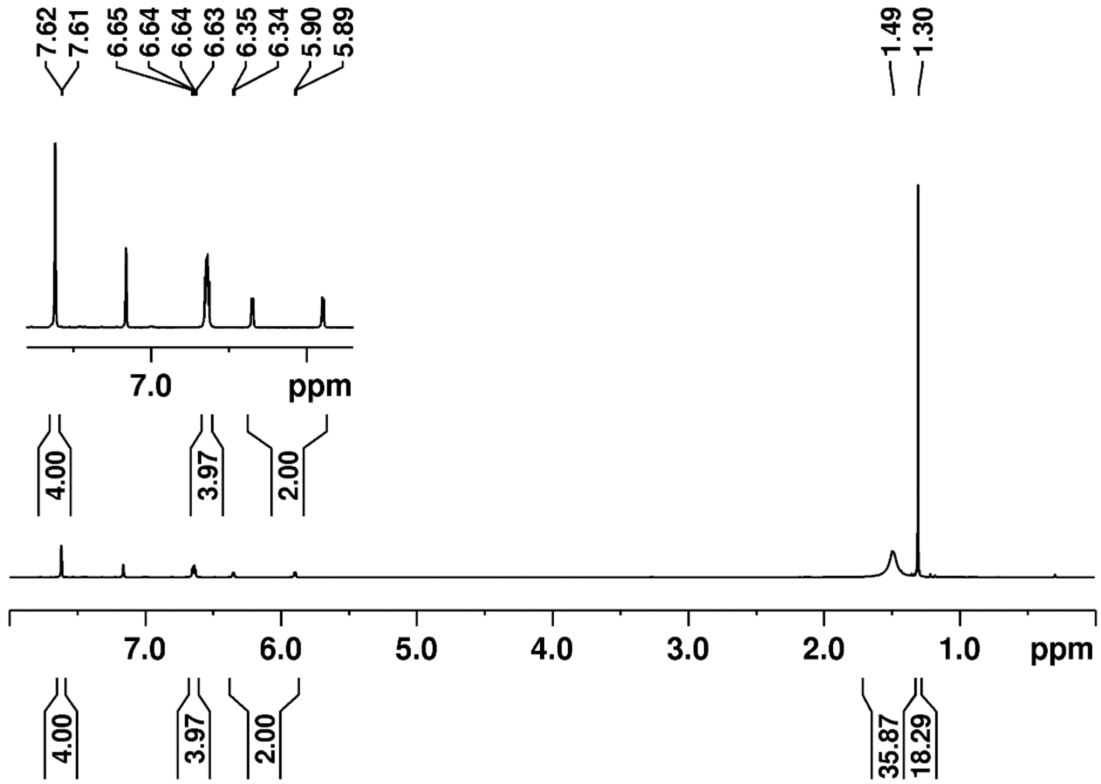

Figure S33. ^{1}H NMR spectrum of *p*-Ph(Mes*PH)$_2$ (**7H**) (500 MHz, C_6D_6).

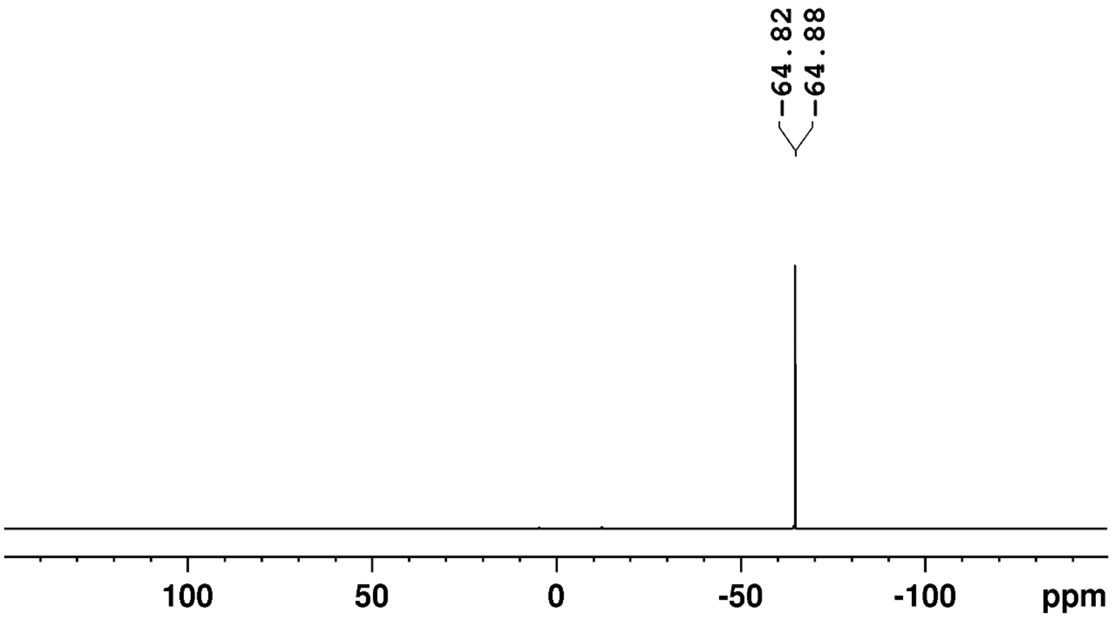

Figure S34. $^{31}P\{^{1}H\}$ NMR spectrum of *p*-Ph(Mes*PH)$_2$ (**7H**) (202 MHz, C_6D_6).

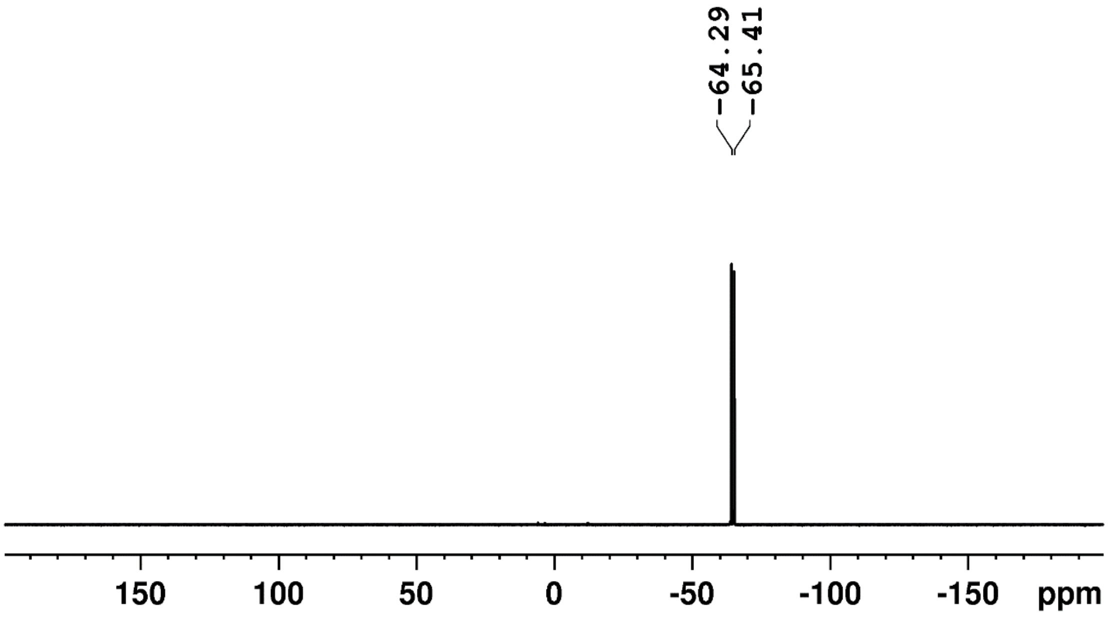

Figure S35. ^{31}P NMR spectrum of *p*-Ph(Mes*PH)$_2$ (**7H**) (202 MHz, C_6D_6).

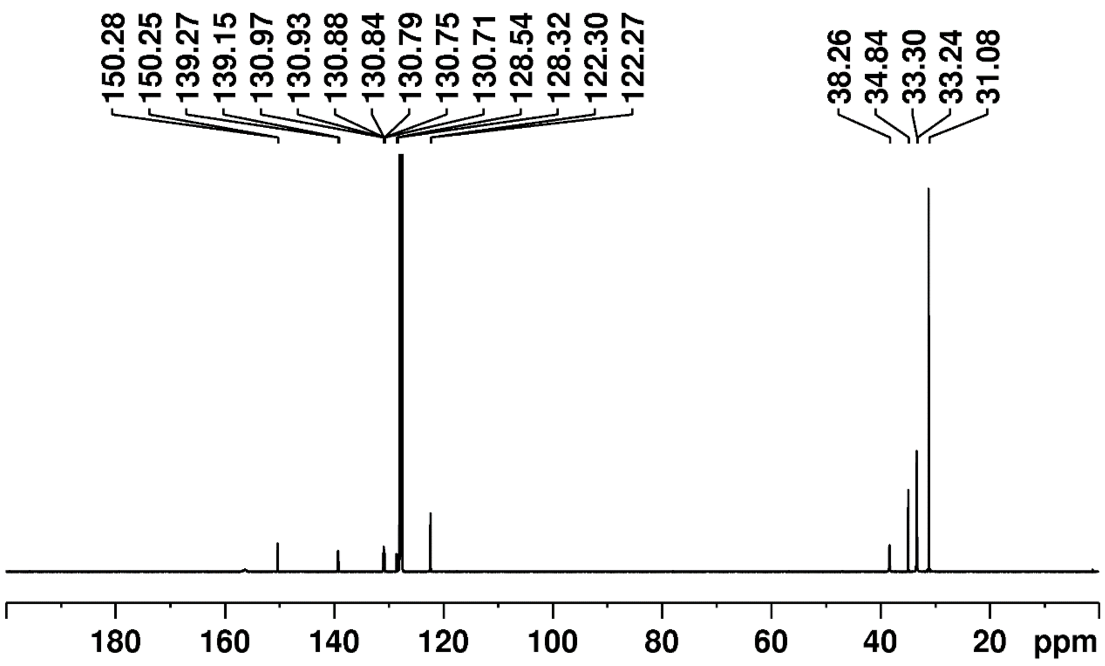

Figure S36. $^{13}C\{^1H\}$ NMR spectrum of *p*-Ph(Mes*PH)$_2$ (**7H**) (126 MHz, C_6D_6).

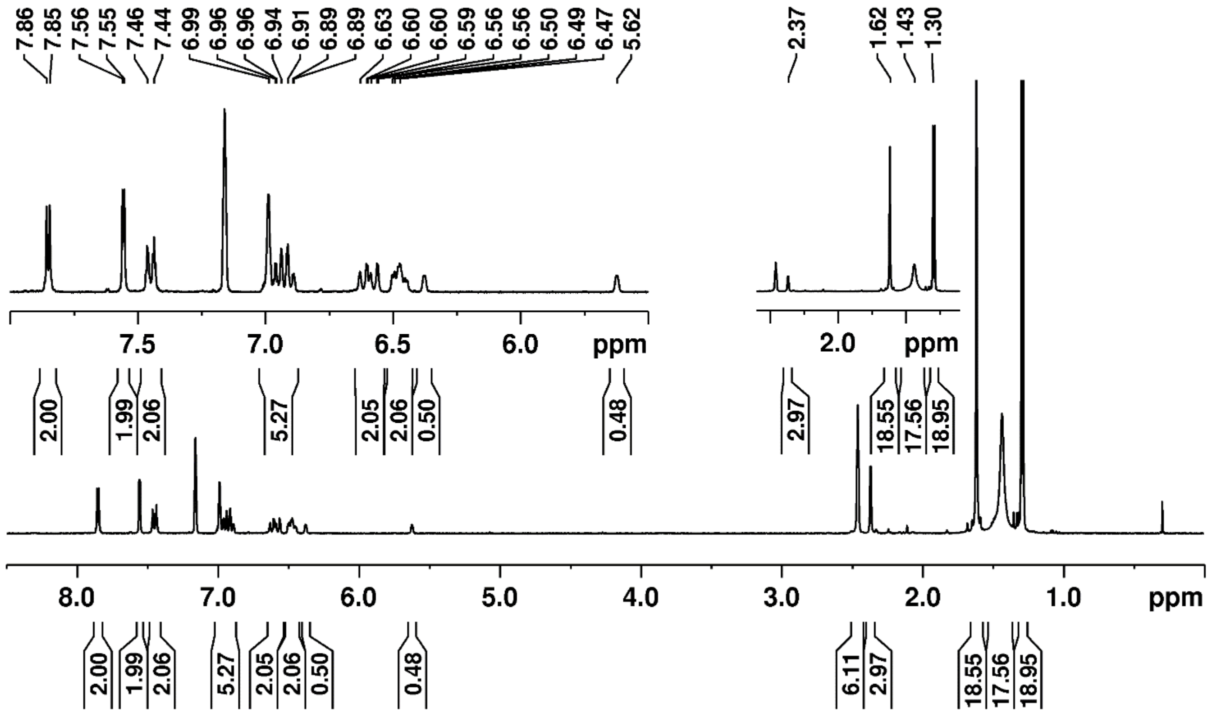

Figure S37. ^{1}H NMR spectrum of **8** (300 MHz, C_6D_6).

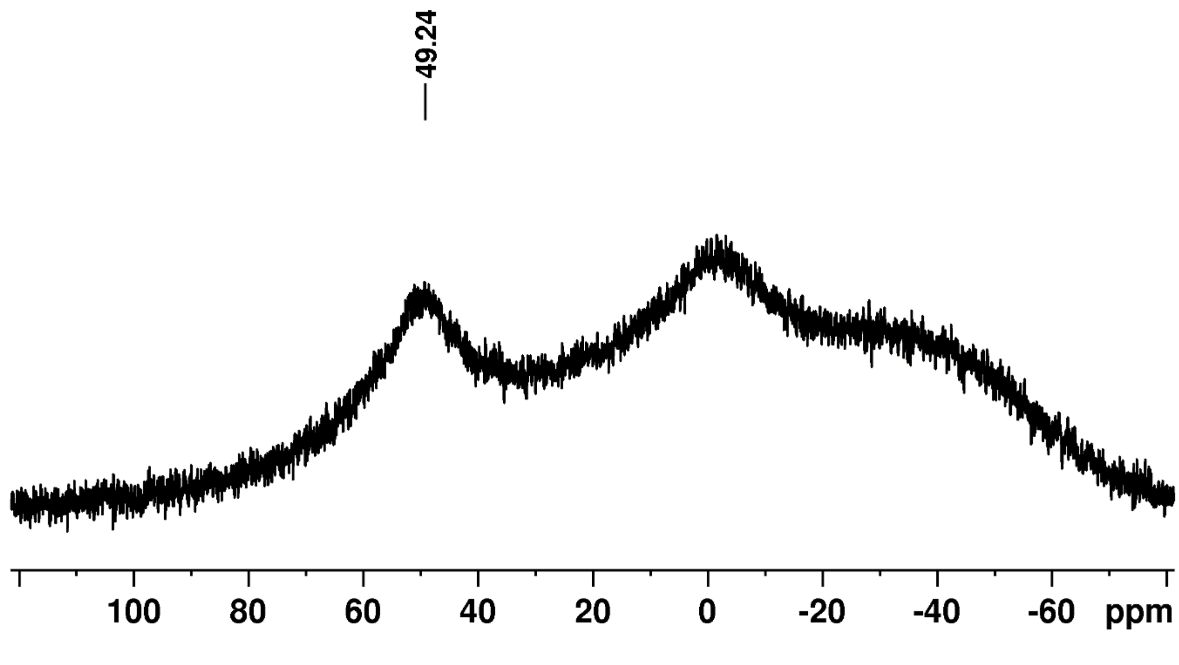

Figure S38. ^{11}B NMR spectrum with reduced background of **8** (96 MHz, $CDCl_3$).

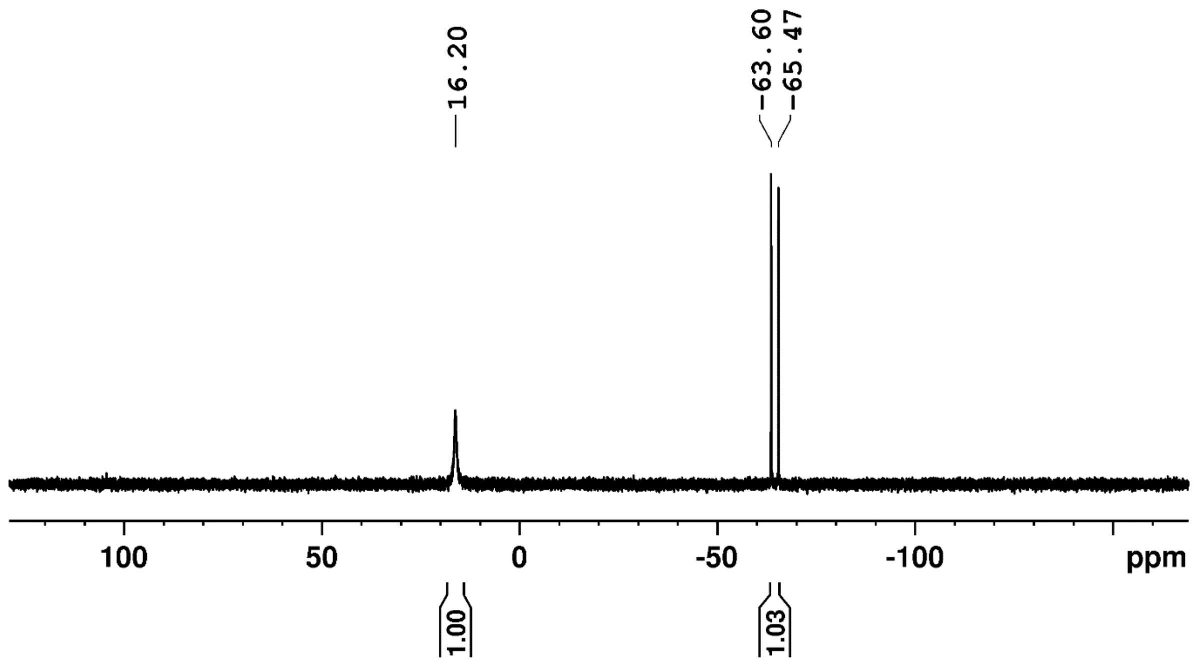

Figure S39. ^{31}P NMR spectrum of **8** (122 MHz, C_6D_6).

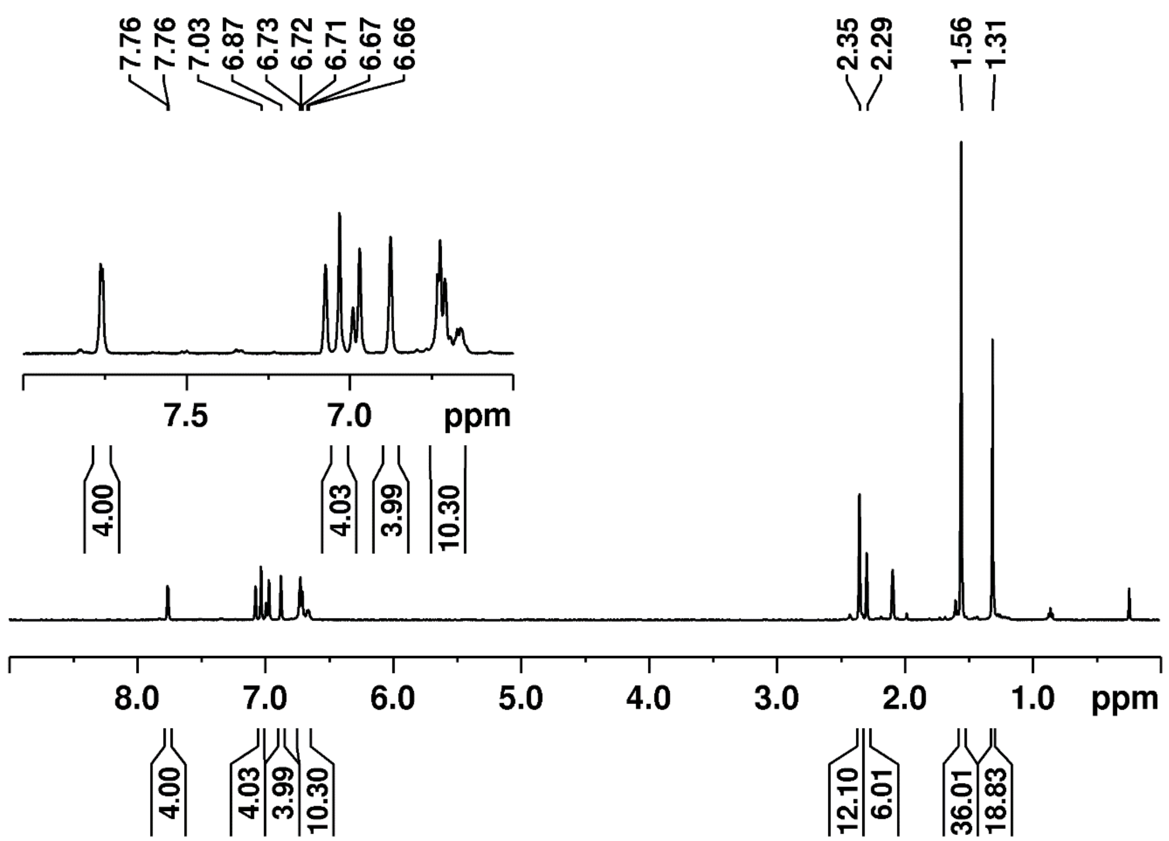

Figure S40. ^{1}H NMR spectrum of PBBP (**2ª**) (500 MHz, toluene-d8).

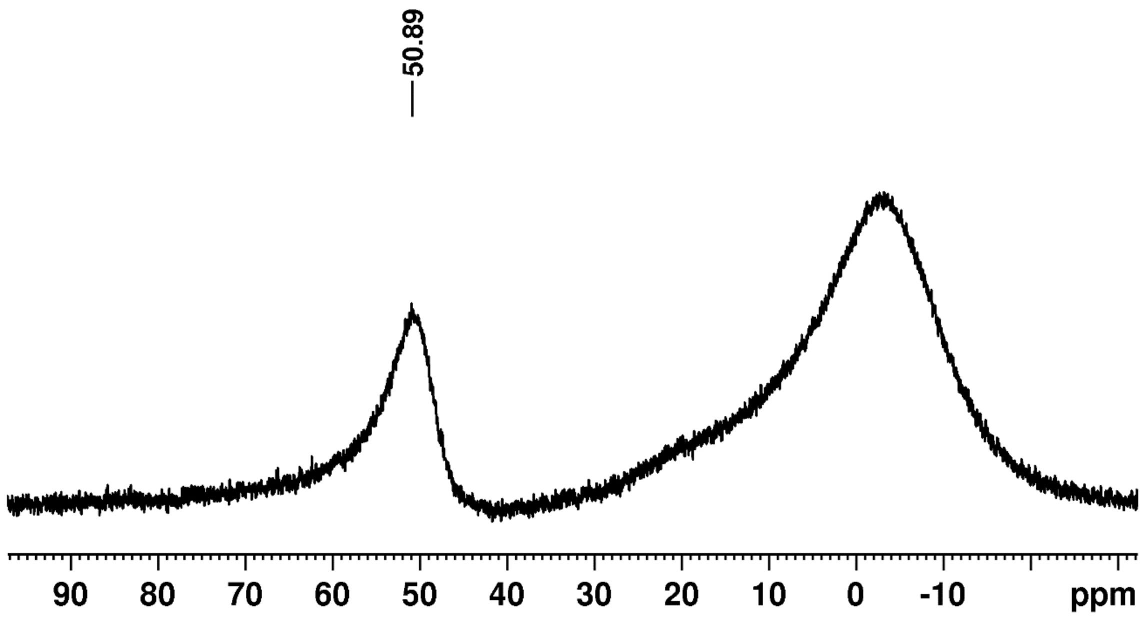

Figure S41. ^{11}B NMR spectrum of PBBP (**2ª**) (160 MHz, toluene-d8, background reduction).

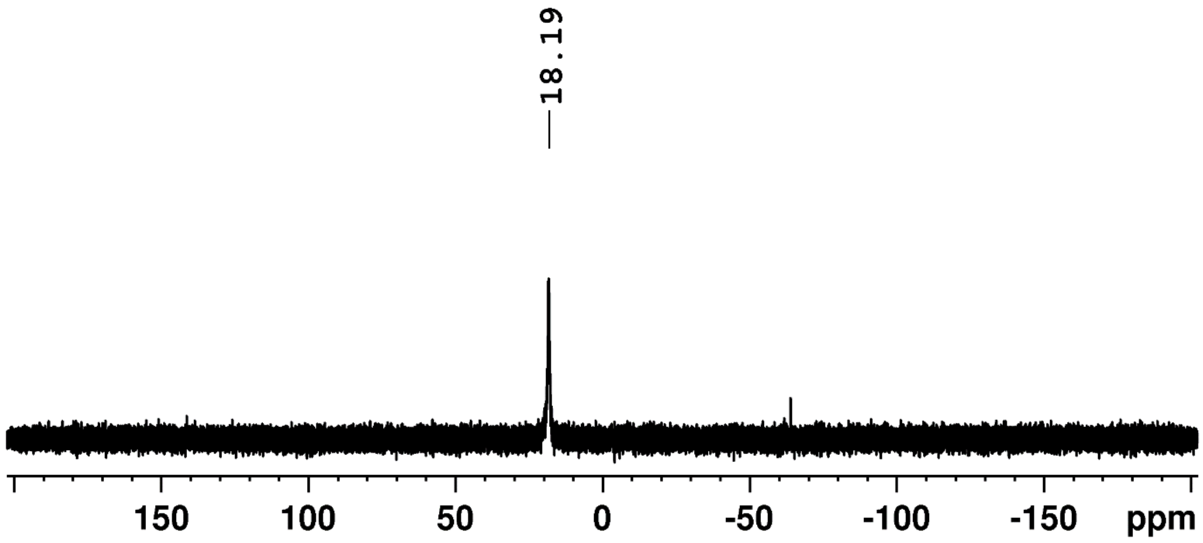

Figure S42. $^{31}P\{^{1}H\}$ NMR spectrum of PBBP (**2**[a]) (202 MHz, toluene-d8).

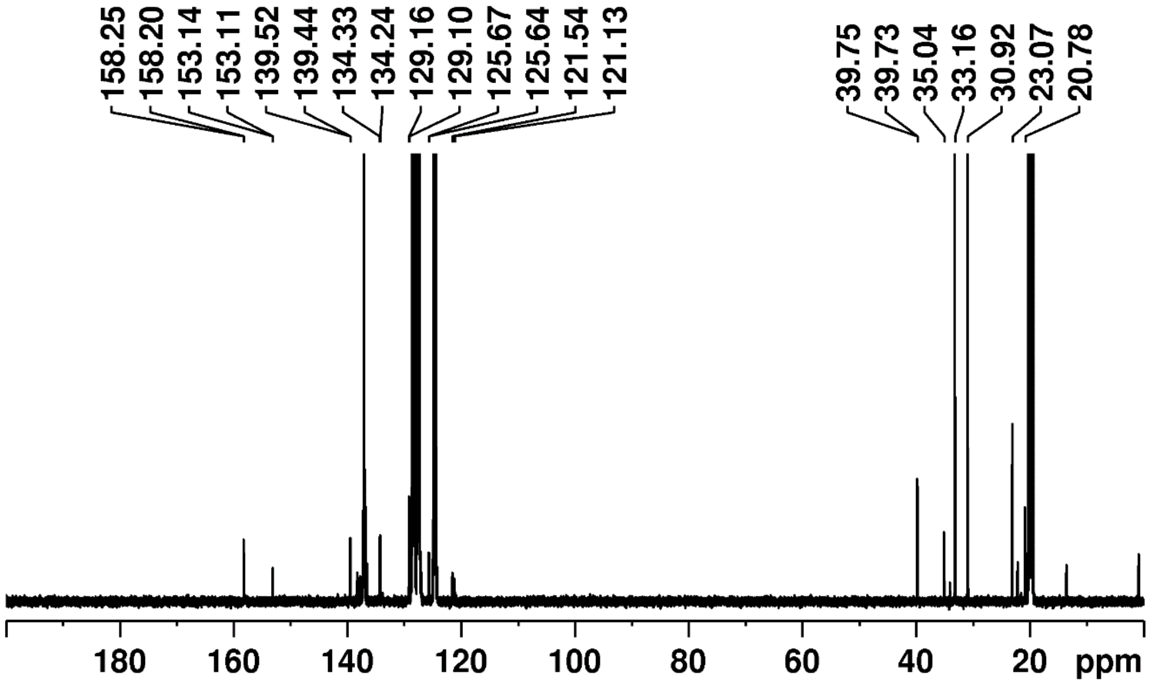

Figure S43. $^{13}C\{^{1}H\}$ NMR spectrum of PBBP (**2**[a]) (126 MHz, toluene-d8).

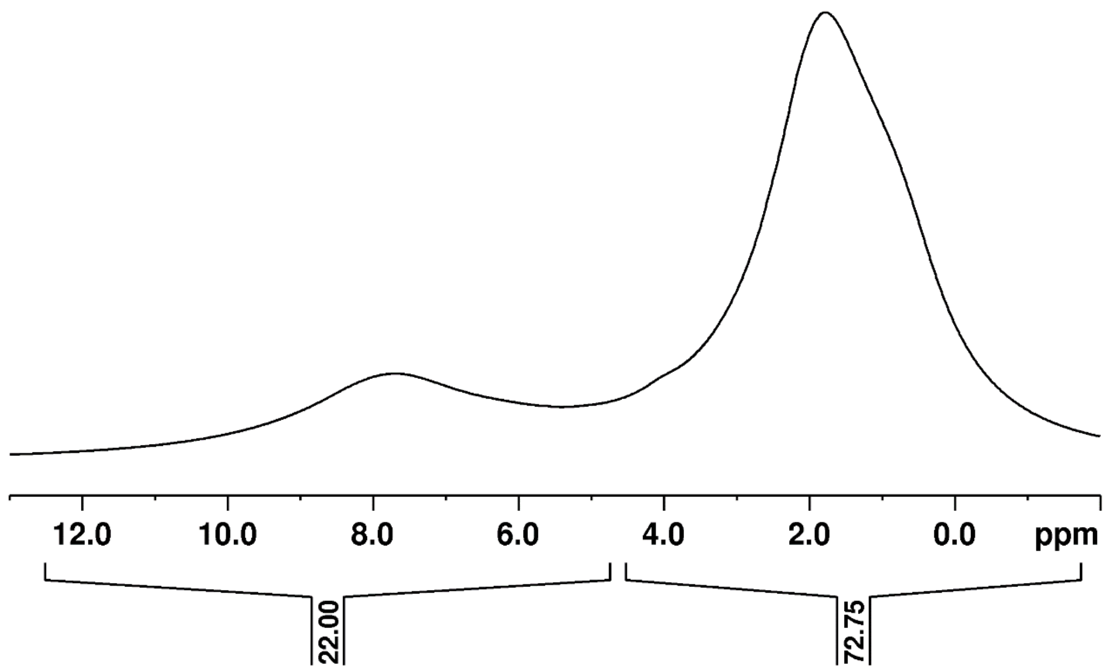

Figure S44. ^{1}H solid-state NMR spectrum of BPPB (**2^b^**) (400 MHz, MAS = 14800 Hz).

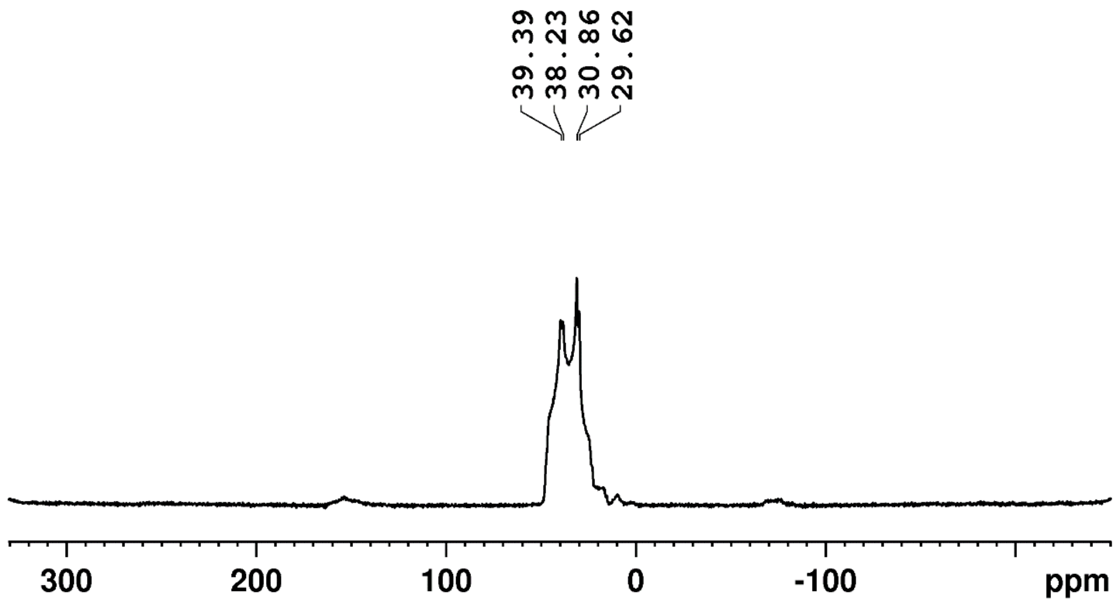

Figure S45. $^{11}B\{^{1}H\}$ solid-state NMR spectrum of BPPB (**2^b^**) (128 MHz, MAS = 14800 Hz).

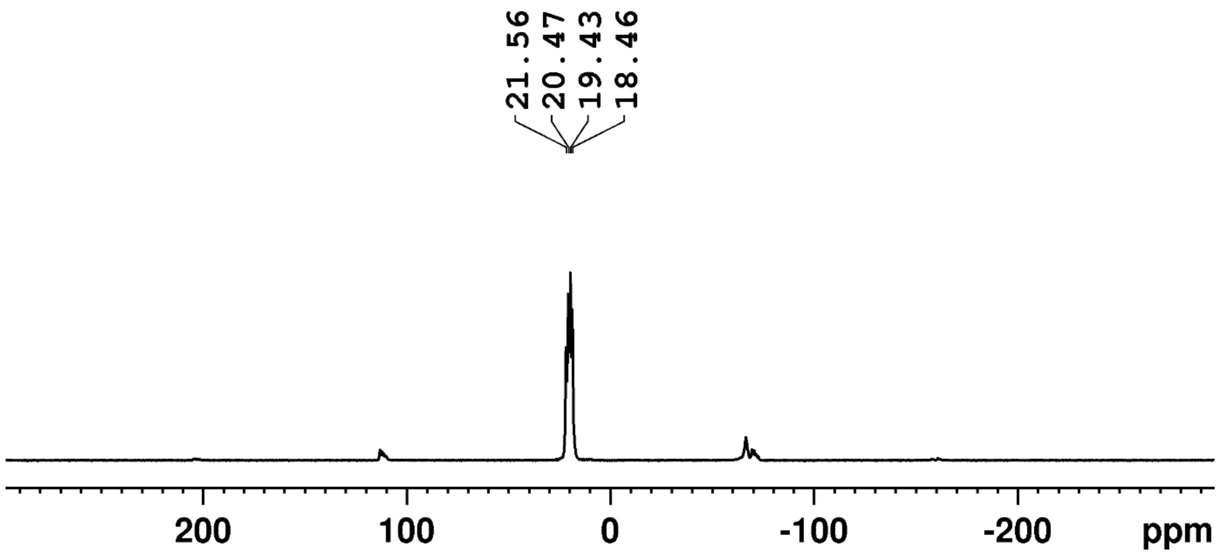

Figure S46. $^{31}P\{^{1}H\}$ solid-state NMR spectrum of BPPB (**2**^{b}) (162 MHz, MAS = 14800 Hz).

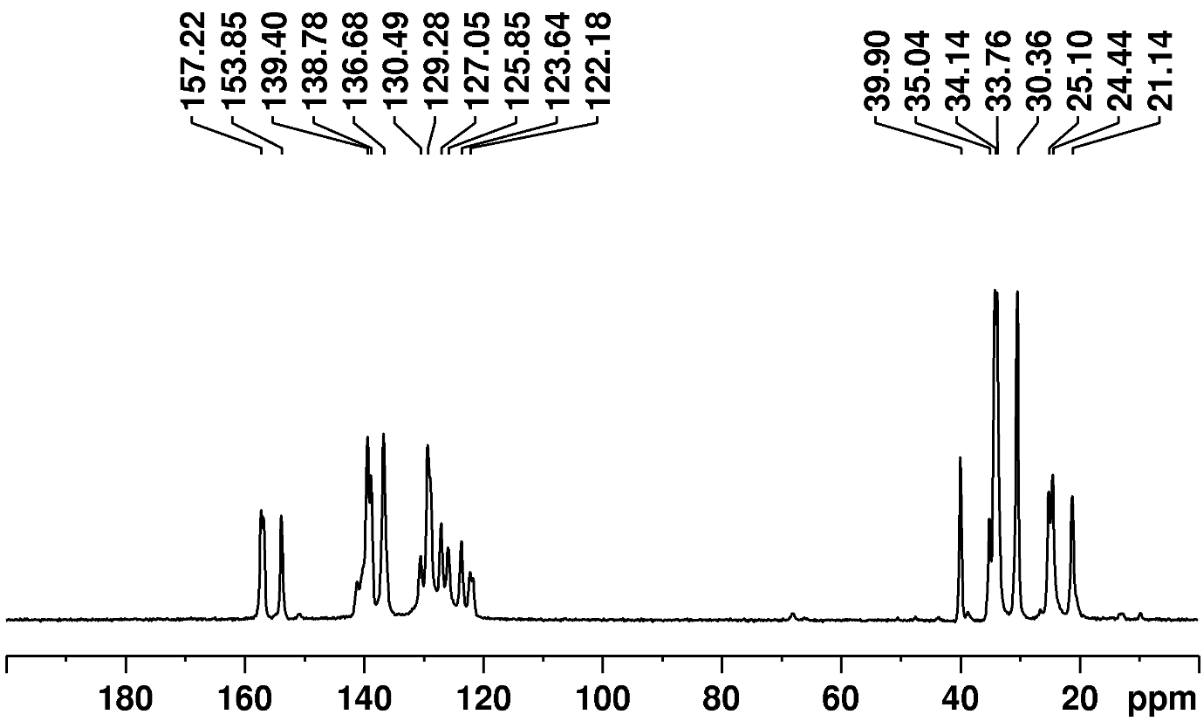

Figure S47. $^{13}C\{^{1}H\}$ solid-state NMR spectrum of BPPB (**2**^{b}) (101 MHz, MAS = 14500 Hz).

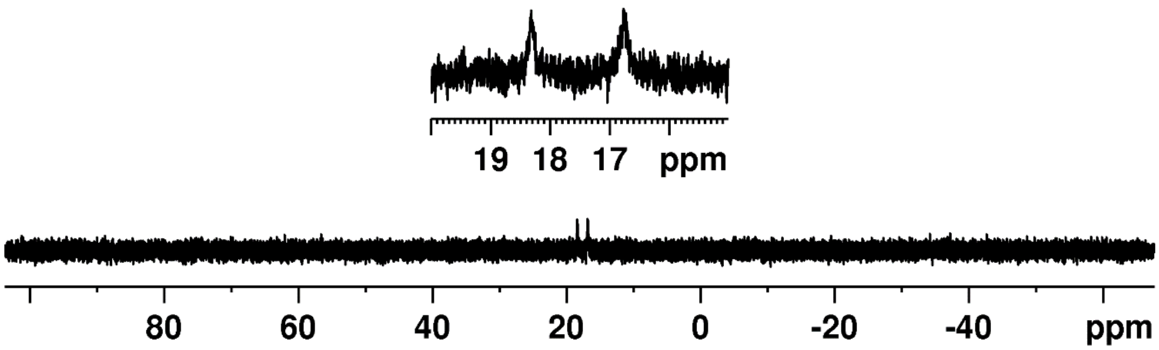

Figure S48. $^{31}P\{^{1}H, ^{11}B\}$ NMR spectrum of $(BPPB)_2$ (**4**) (243 MHz, THF-d8).

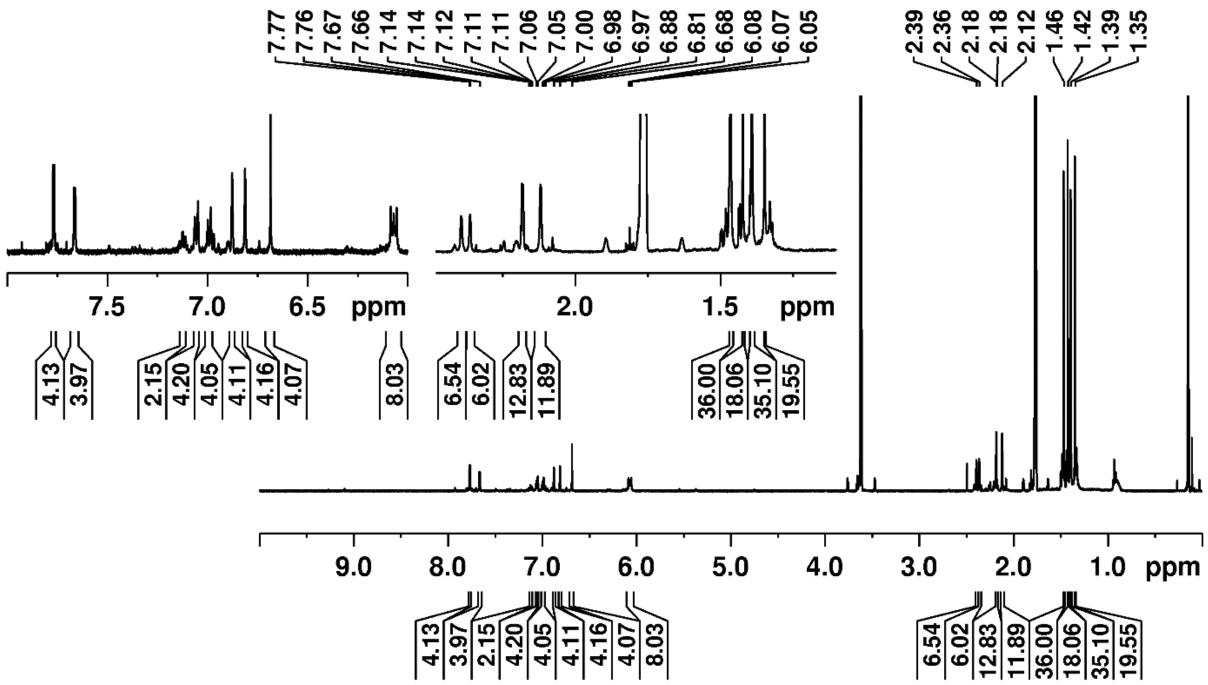

Figure S49. ^{1}H NMR spectrum of $(BPPB)_2$ (**4**) (500 MHz, THF-d8) very noisy due to bad solubility of compound **4** in THF.

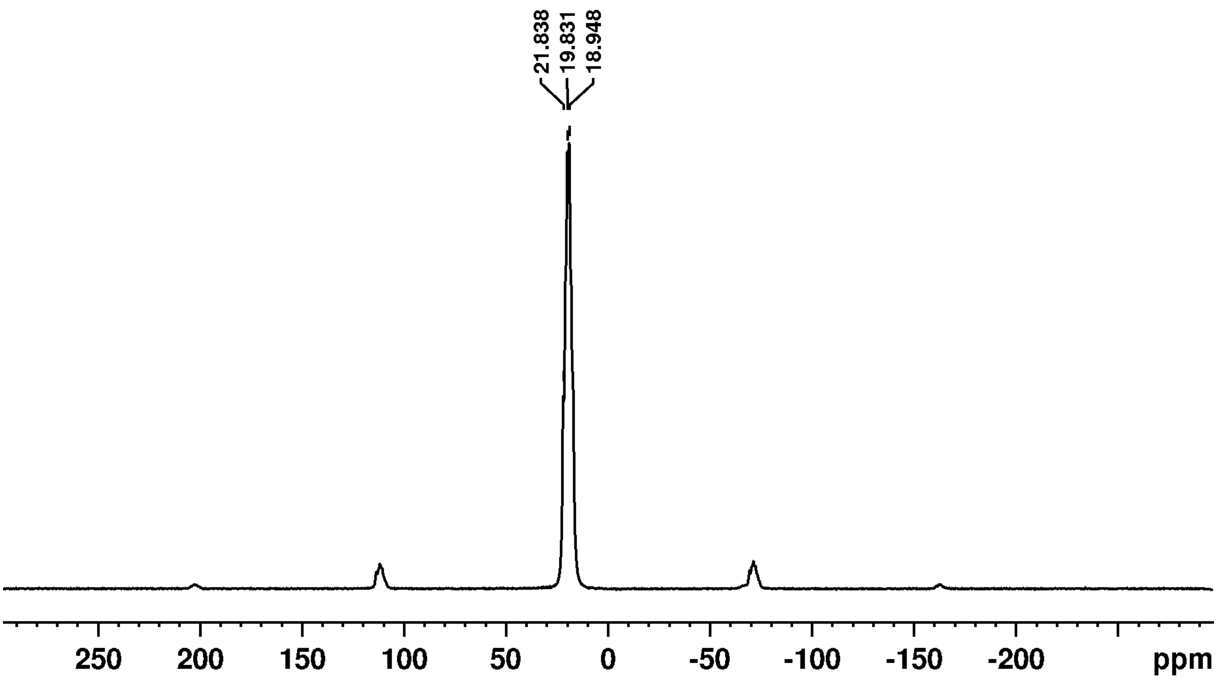

Figure S50. $^{31}P\{^{1}H\}$ NMR spectrum of $(BPPB)_2$ (**4**) (162 MHz, MAS = 14800 Hz). All other signals in the spectrum can be assigned to rotational side bands with 14800 Hz shift.

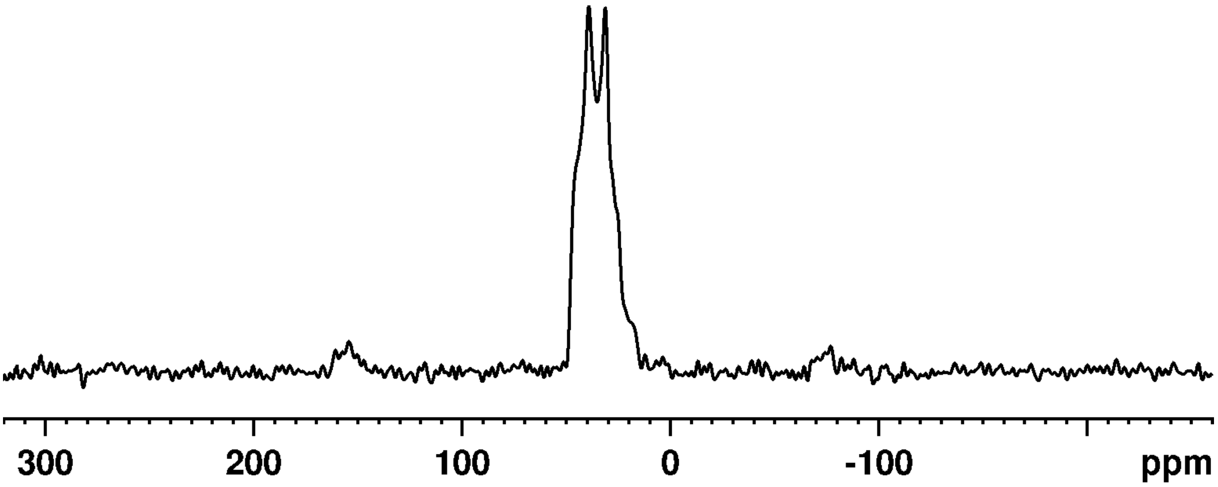

Figure S51. $^{11}B\{^{1}H\}$ solid-state NMR spectrum of $(BPPB)_2$ (**4**) (128 MHz, MAS = 14800 Hz).

1.5. High Resolution Mass Spectra

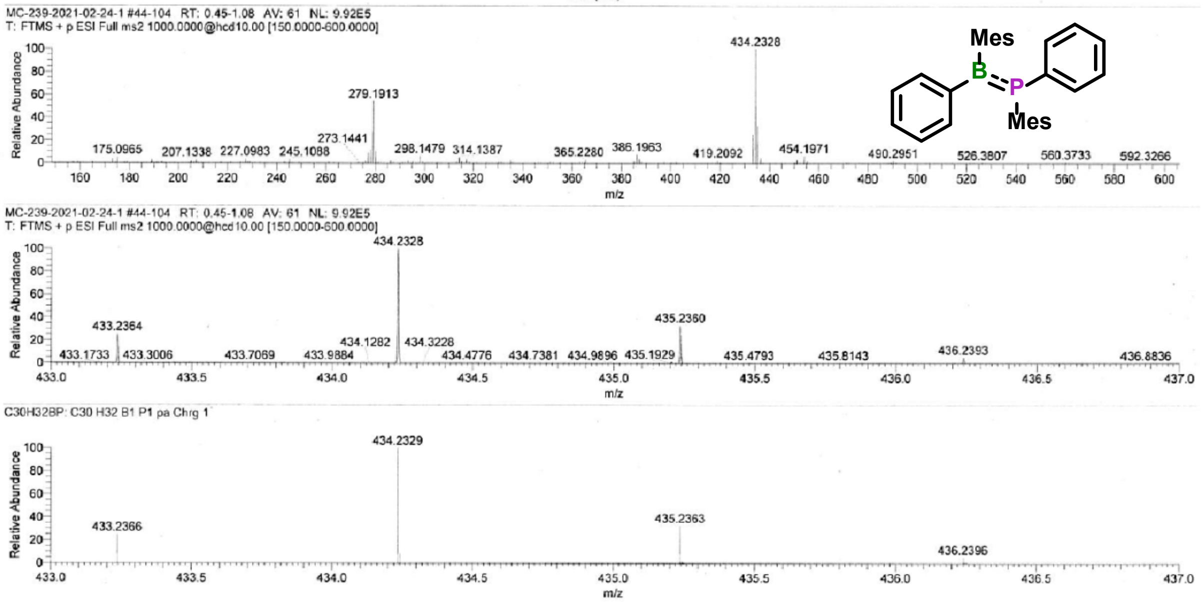

Figure S52. HRMS spectrum of **1^Mes^** (MesPhBPMesPh) (LIFDI).

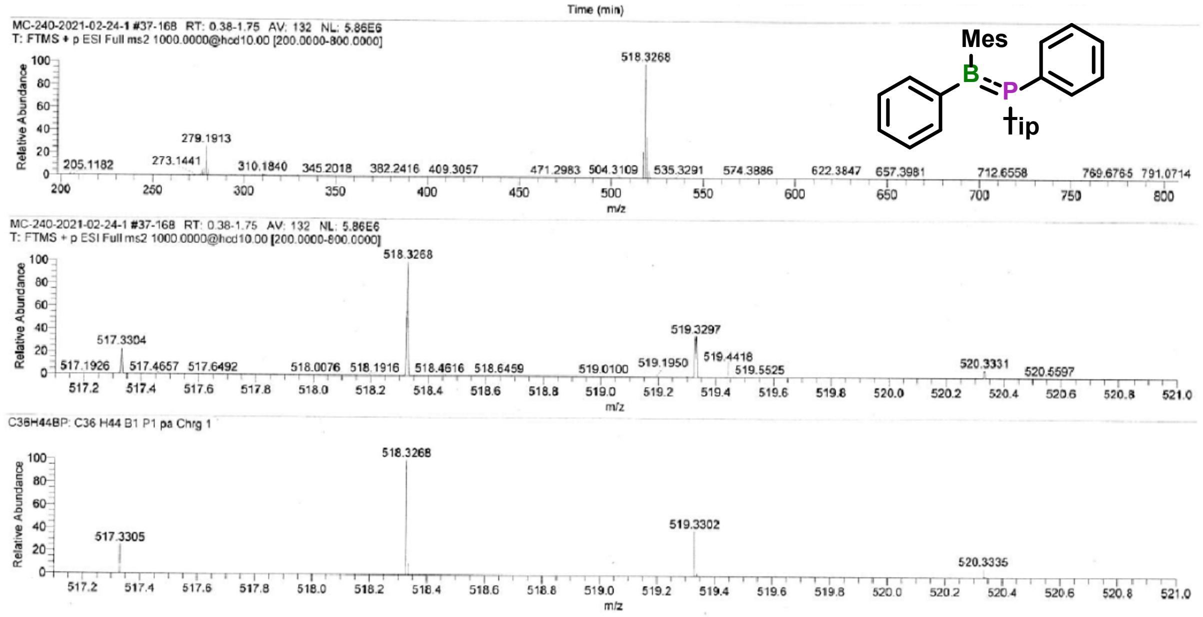

Figure S53. HRMS spectrum of **1^Tip^** (MesPhBPTipPh) (LIFDI).

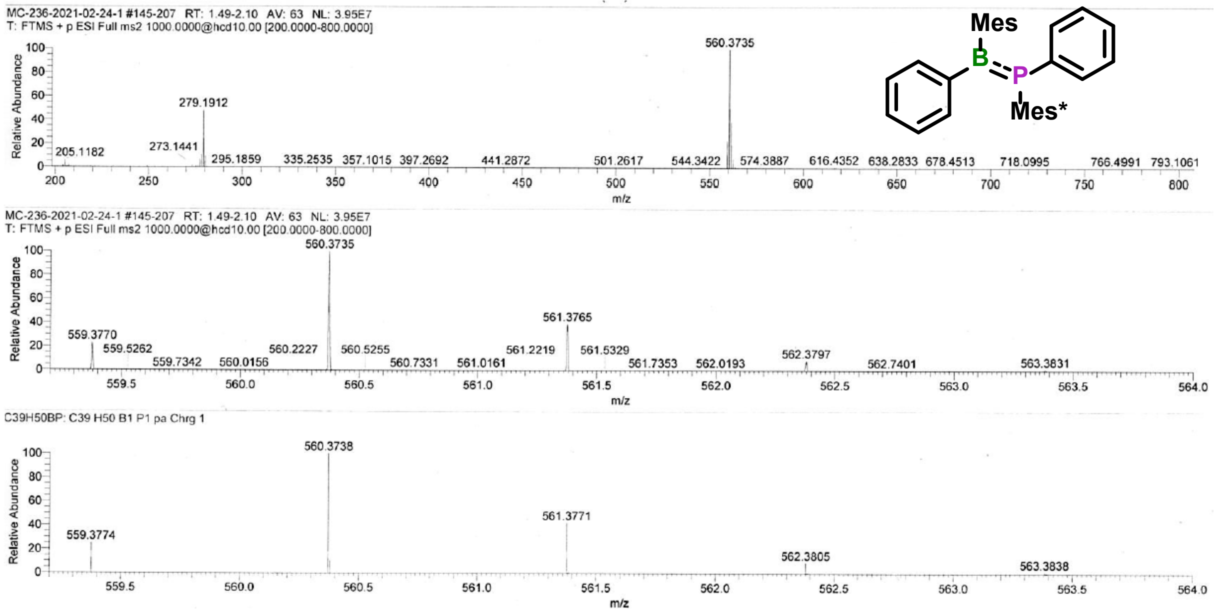

Figure S54. HRMS spectrum of **1**$^{Mes^*}$ (MesPhBPMes*Ph) (LIFDI).

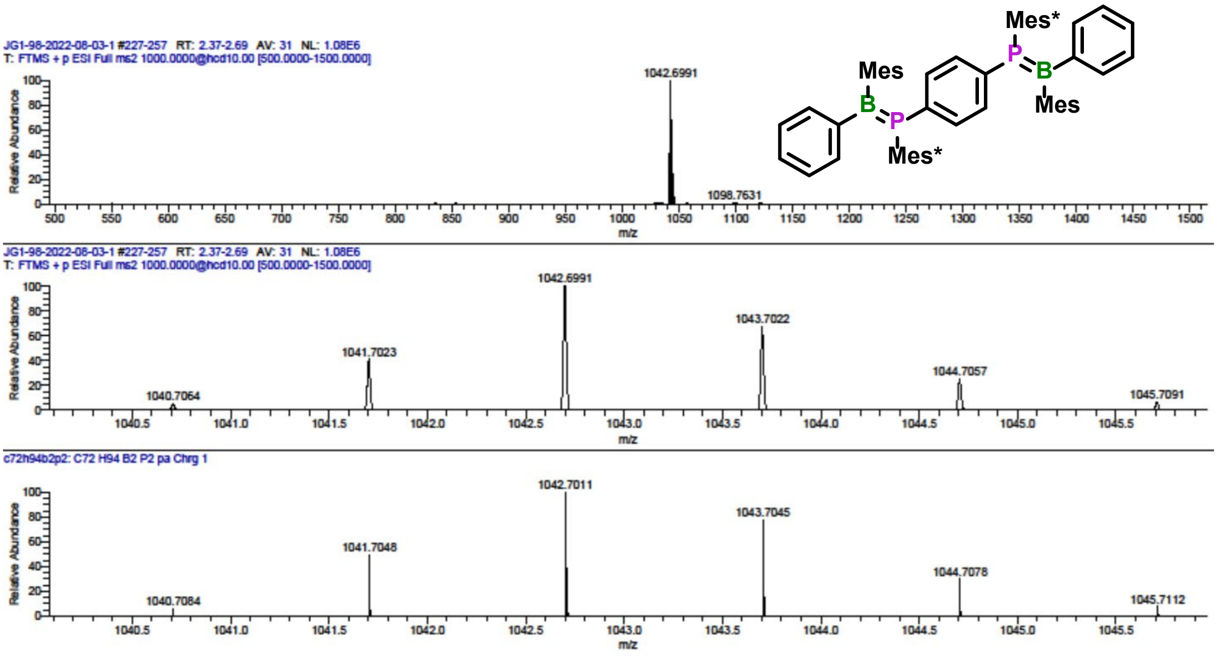

Figure S55. HRMS spectrum of **2**^{b} (LIFDI).

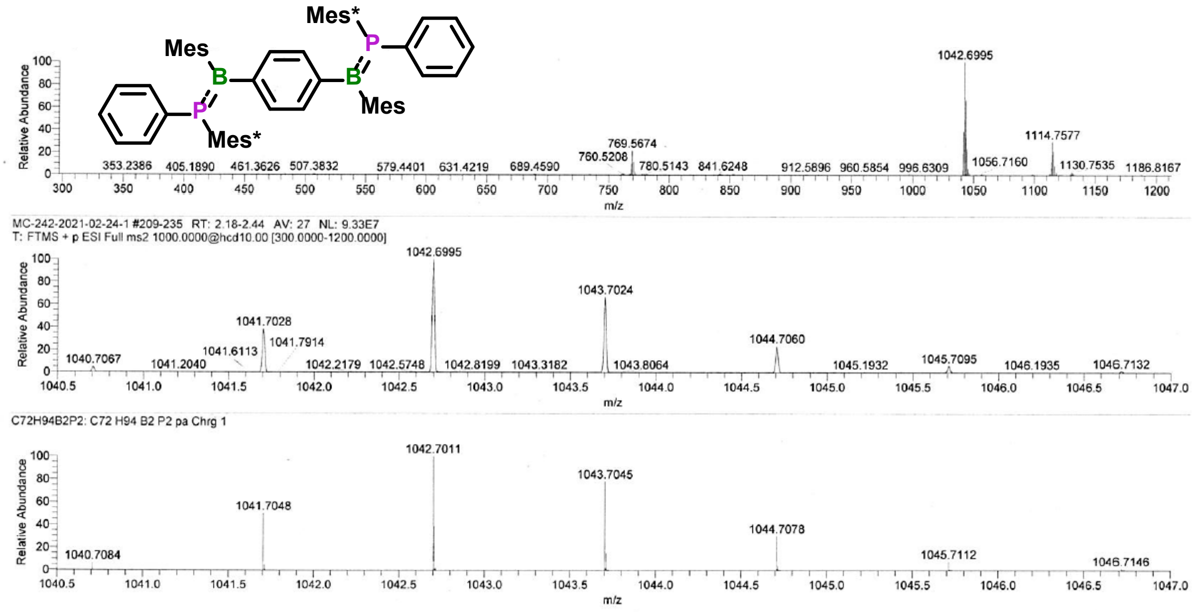

Figure S56. HRMS spectrum of **2**[a] (LIFDI).

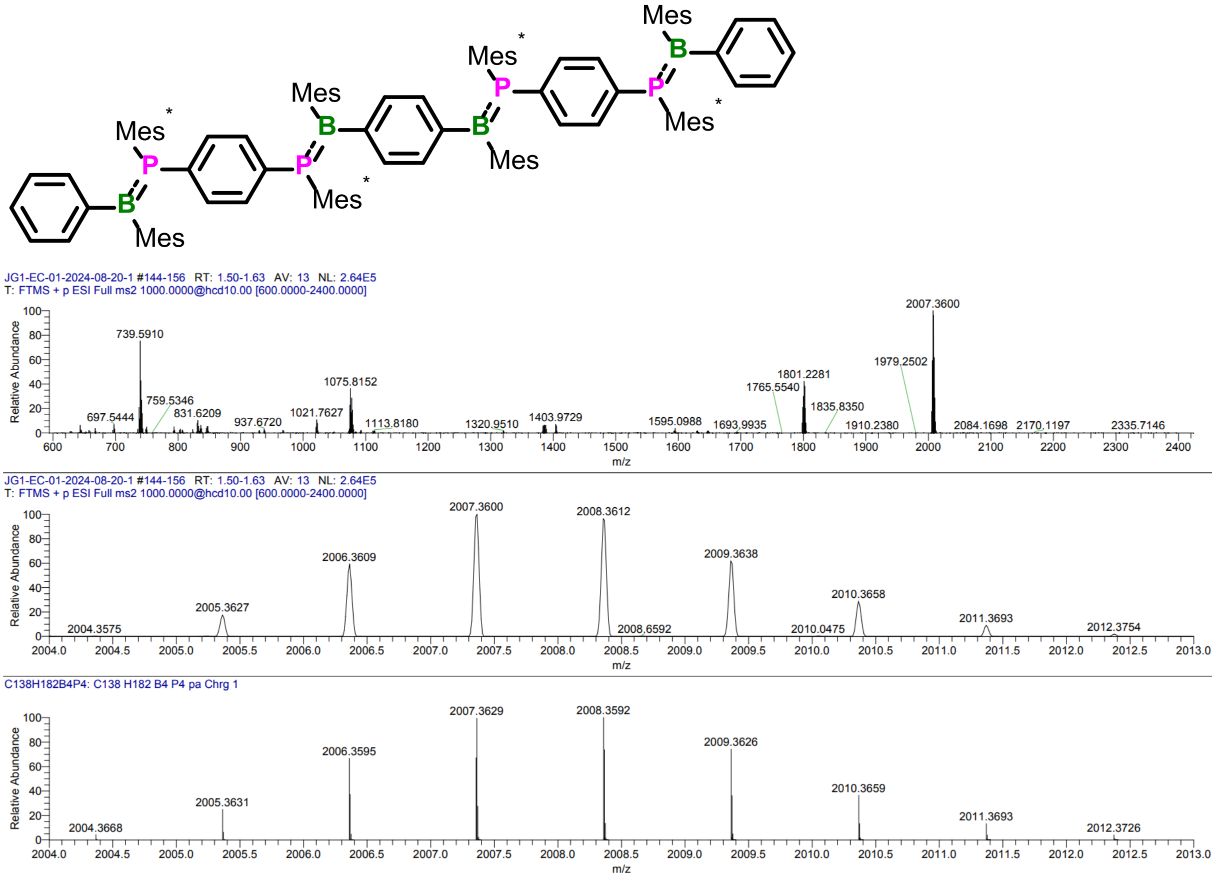

Figure S57. HRMS spectrum of **4** (LIFDI).

1.6. UV/Vis and Fluorescence Spectra

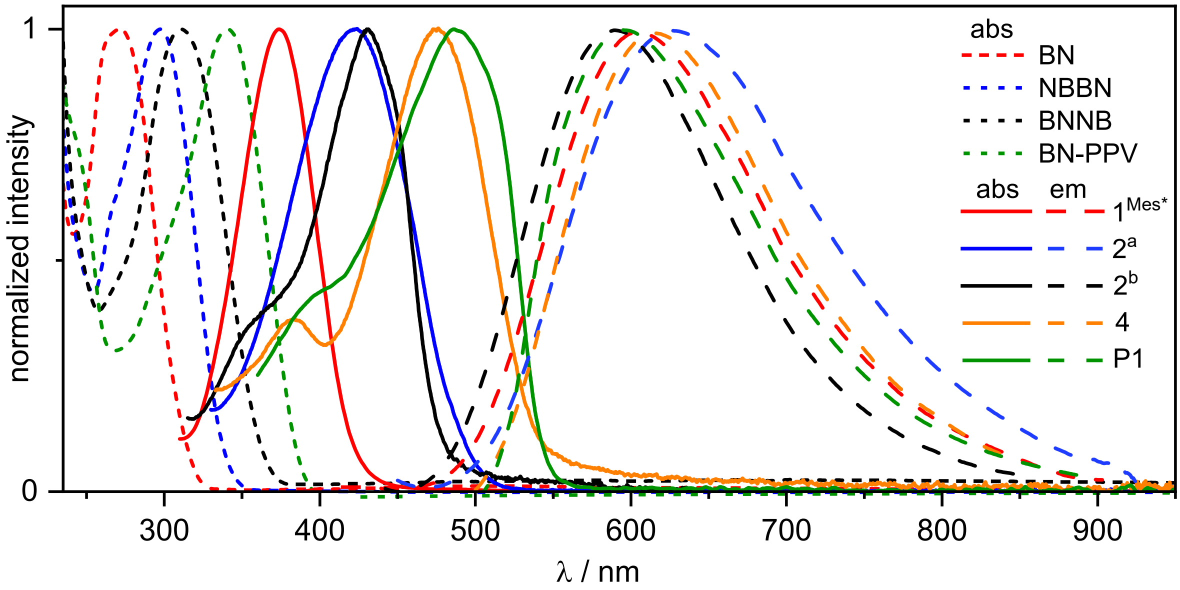

Figure S58. Normalized UV-vis absorption and fluorescence emission spectra of **1**$^{\mathbf{Mes*}}$, **2**$^{\mathbf{a}}$, **2**$^{\mathbf{b}}$, **4**, and **P1** compared to the absorption spectra of corresponding BN-congeners in THF.

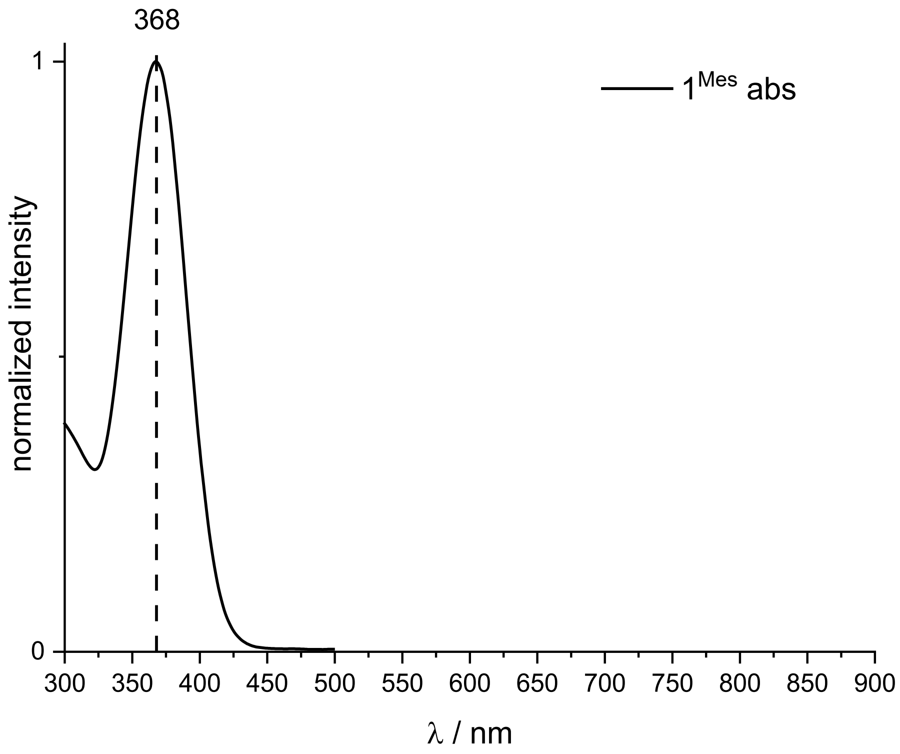

Figure S59: Normalized UV-vis absorption spectrum of **1**$^{\mathbf{Mes}}$ in THF.

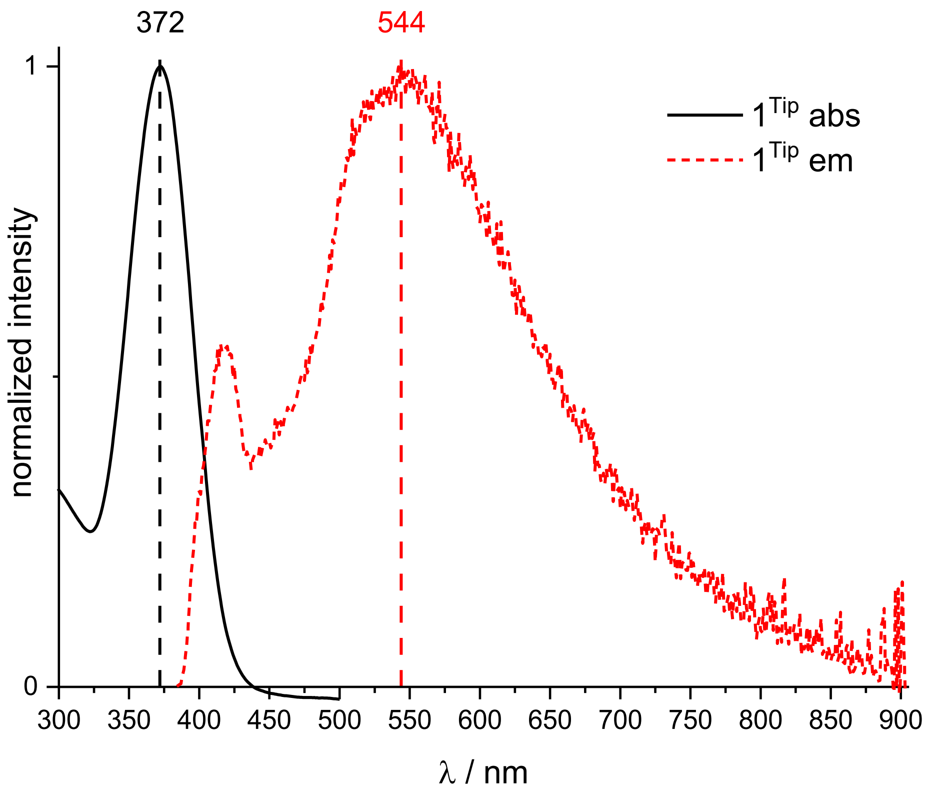

Figure S60. Normalized UV-vis absorption and fluorescence spectra of $\mathbf{1}^{\mathbf{Tip}}$ in THF.

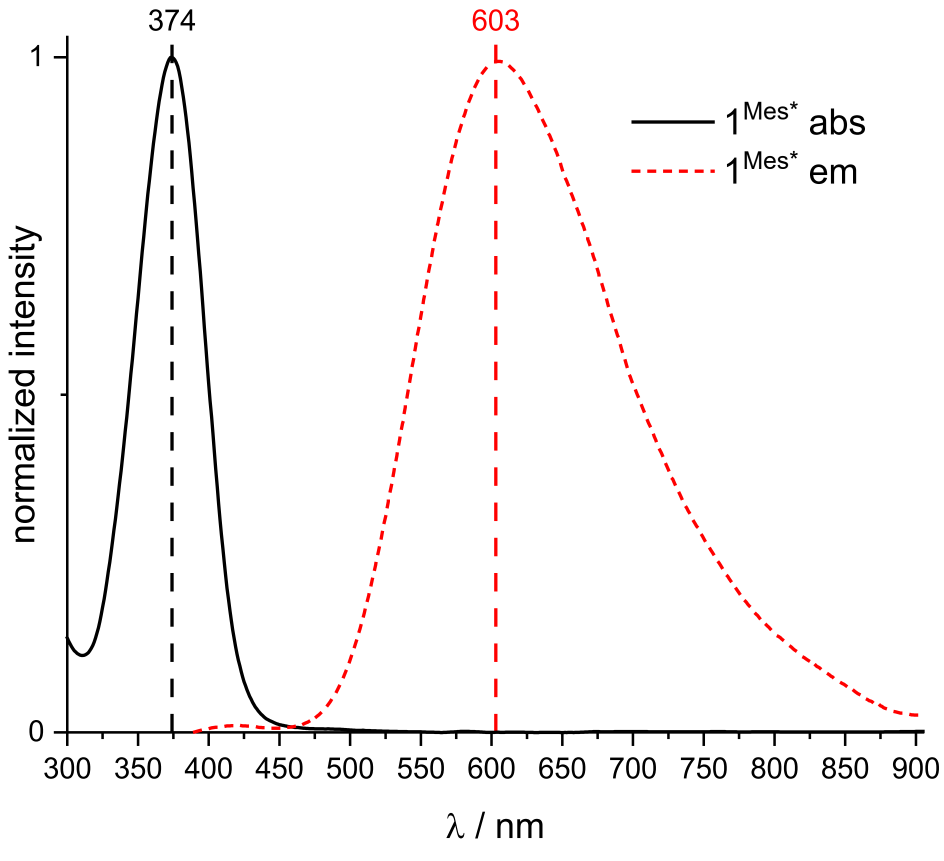

Figure S61. Normalized UV-vis absorption and fluorescence spectra of $\mathbf{1}^{\mathbf{Mes*}}$ in THF.

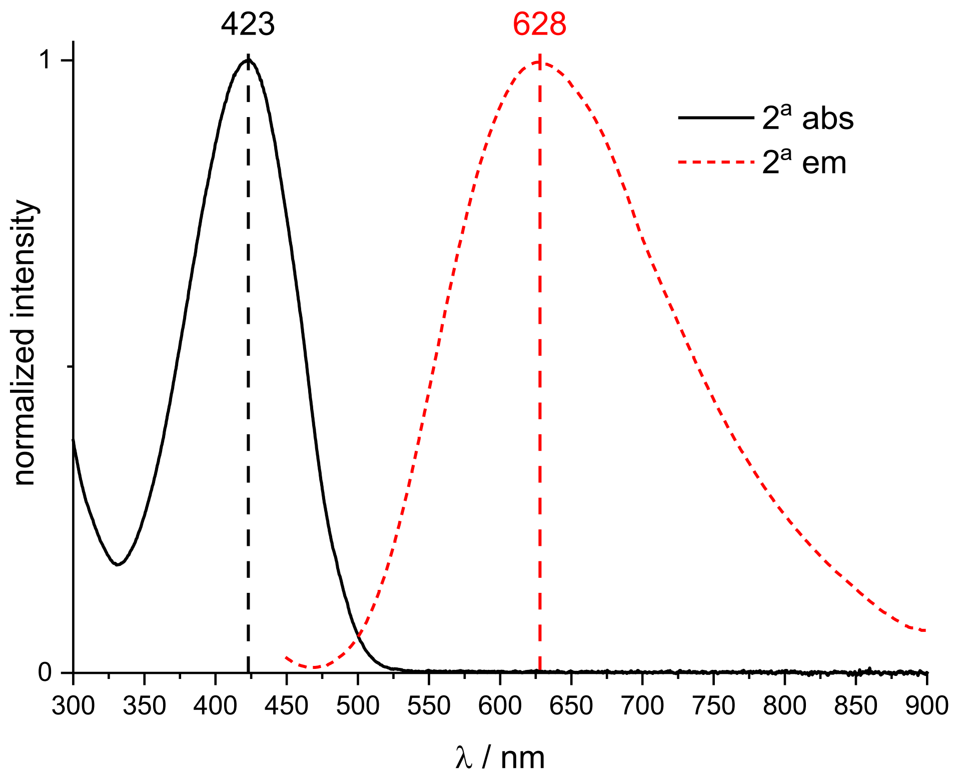

Figure S62. Normalized UV-vis absorption and fluorescence spectra of **2^a^** in THF.

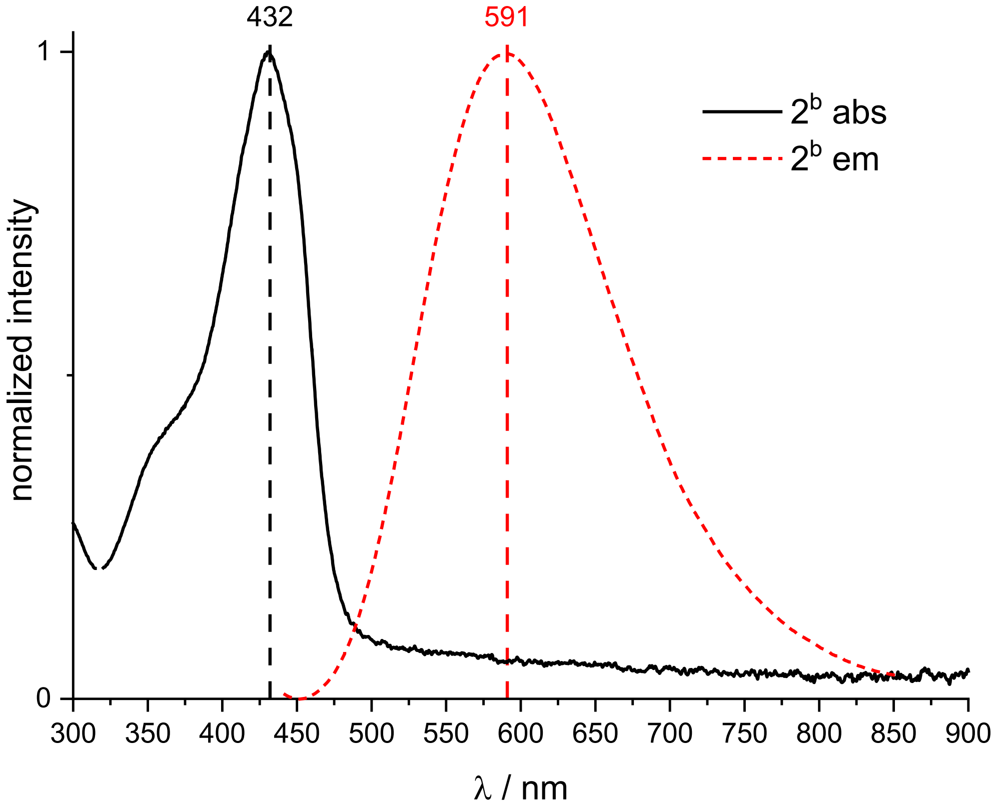

Figure S63. Normalized UV-vis absorption and fluorescence spectra of **2^b^** in THF.

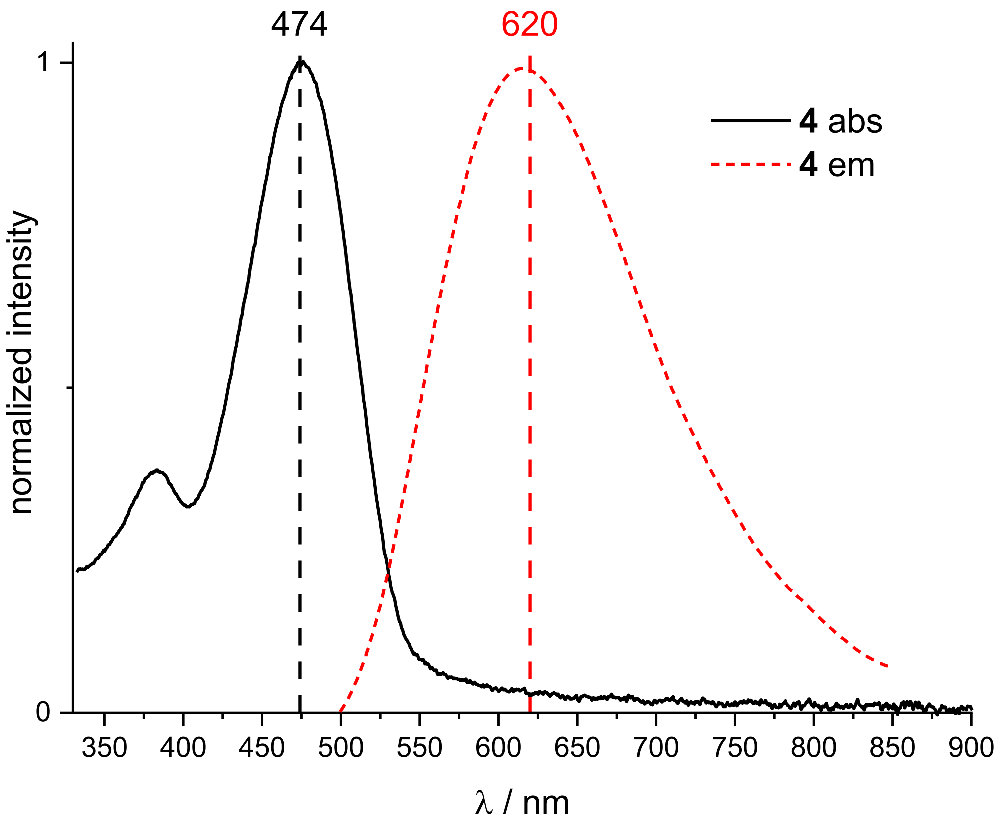

Figure S64. Normalized UV-vis absorption and fluorescence spectra of **4** in THF.

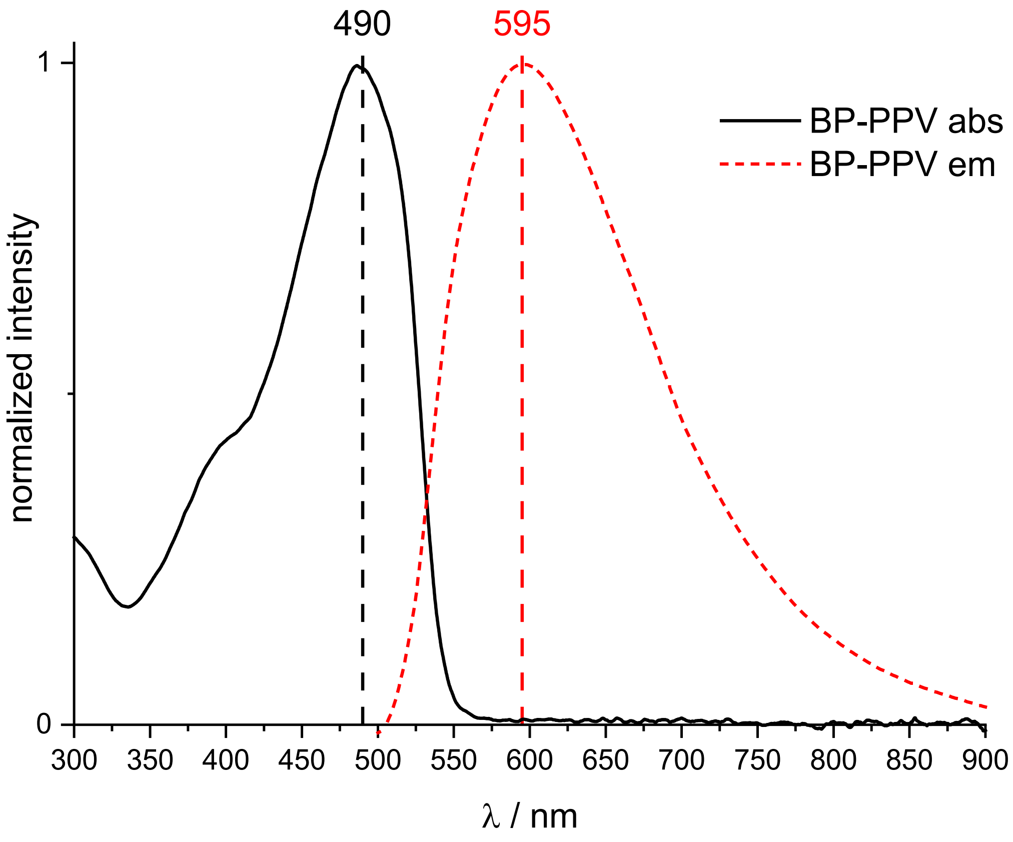

Figure S65. Normalized UV-vis absorption and fluorescence spectra of **P1** in THF.

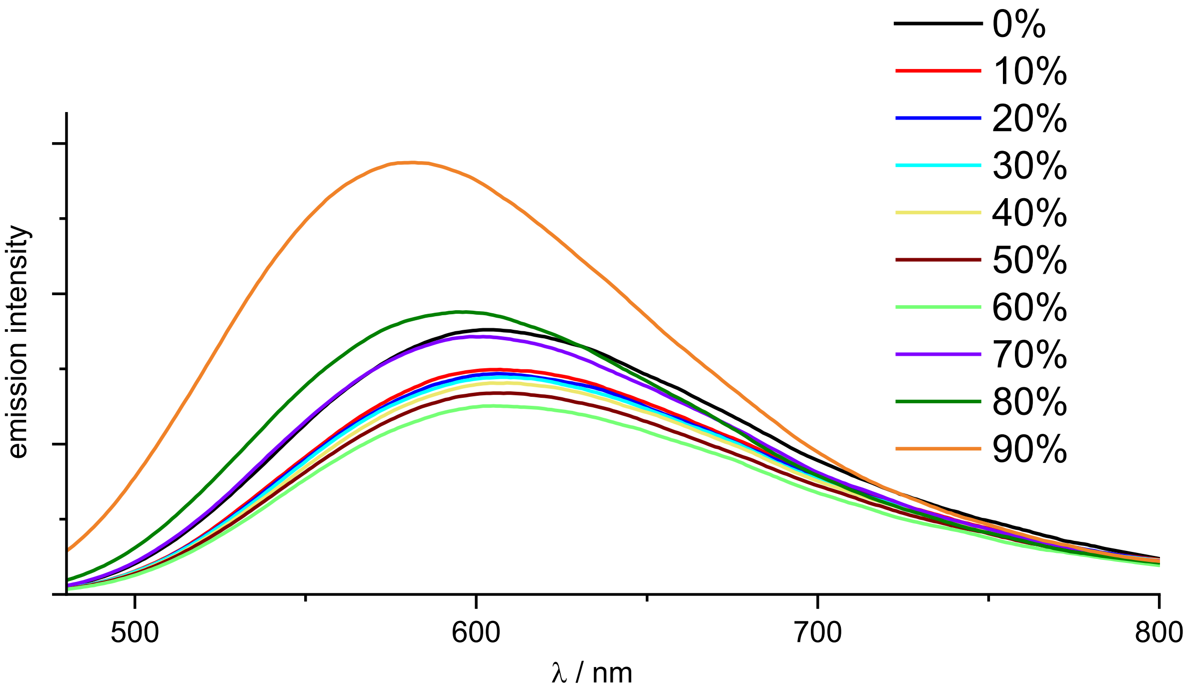

Figure S66. Emission spectra of **1**^{Mes*} in different THF / water mixtures (conc. 5×10^{-5} M) with different water ratios (0−90%).

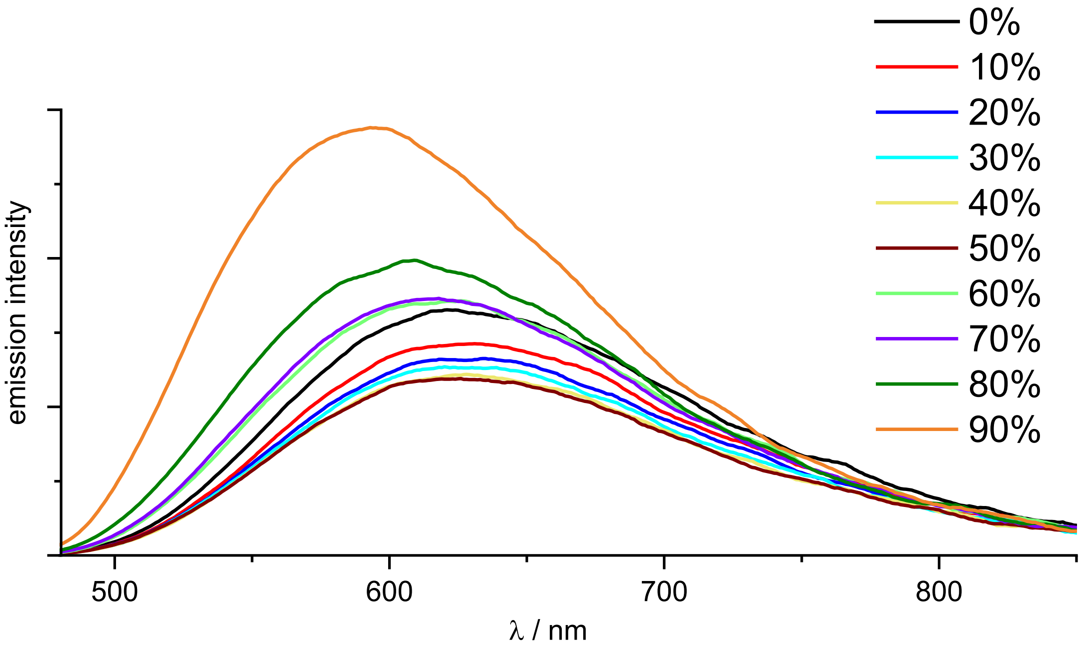

Figure S67. Emission spectra of **2**^{a} in different THF / water mixtures (conc. 5×10^{-5} M) with different water ratios (0−90%).

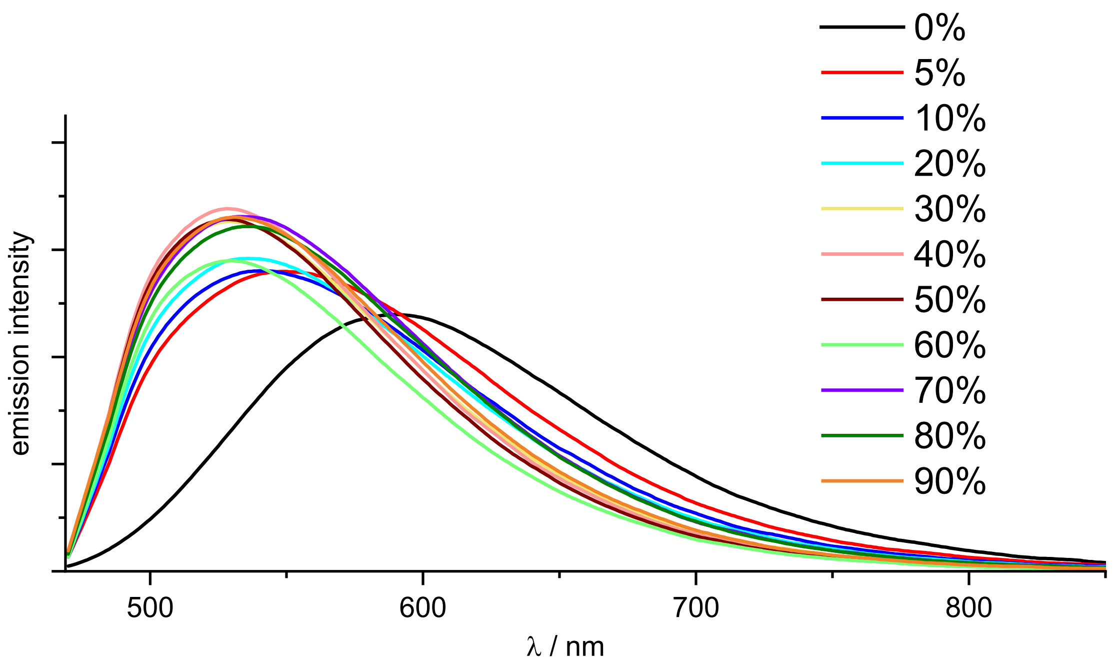

Figure S68. Emission spectra of **2**^{b} in different THF / water mixtures (conc. 5×10^{-5} M) with different water ratios (0−90%).

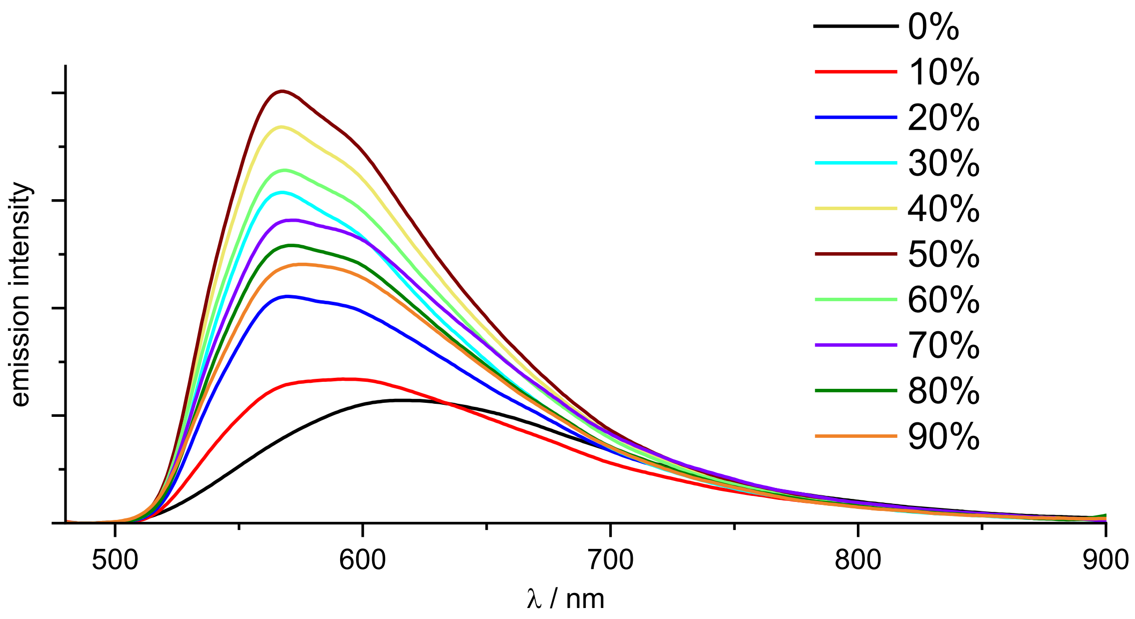

Figure S69. Emission spectra of **4** in different THF / water mixtures (conc. 5×10^{-5} M) with different water ratios (0−90%).

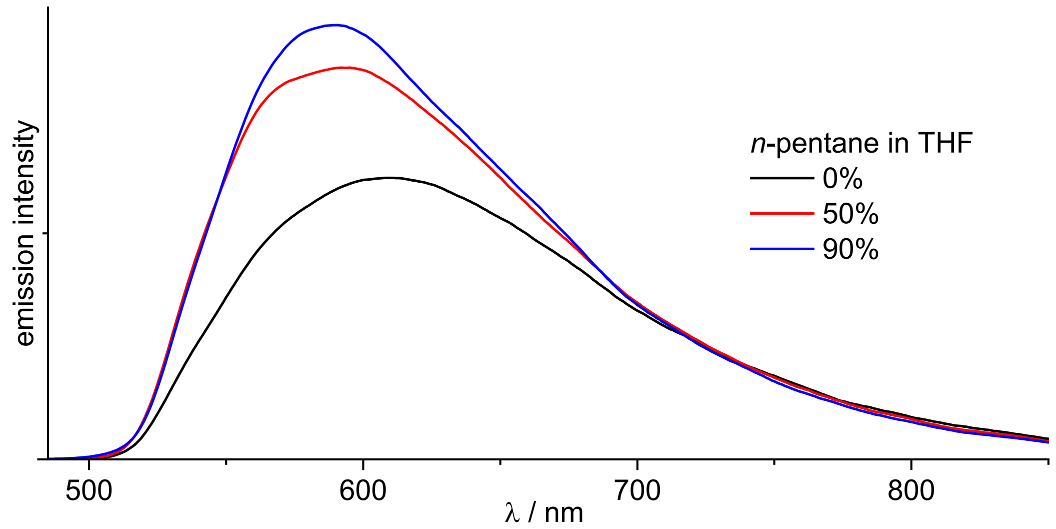

Figure S70. Emission spectra of **4** in different THF / *n*-pentane mixtures (conc. 5×10^{-5} M) with different *n*-pentane ratios (0−90%).

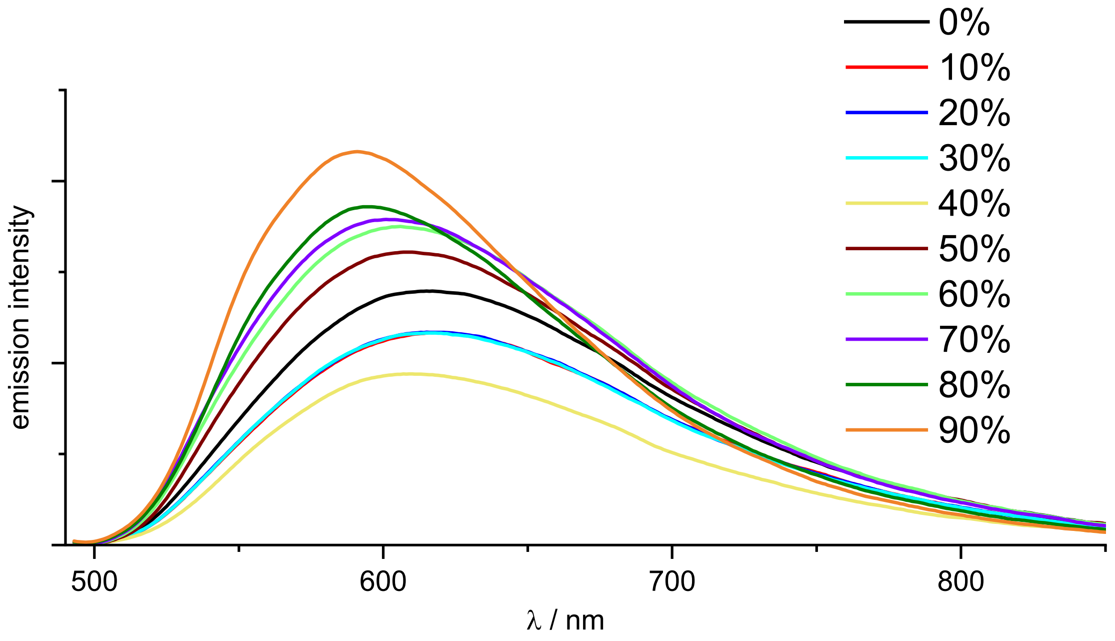

Figure S71. Emission spectra of **P1** in different THF / water mixtures (conc. 5×10^{-5} M) with different water ratios (0−90%).

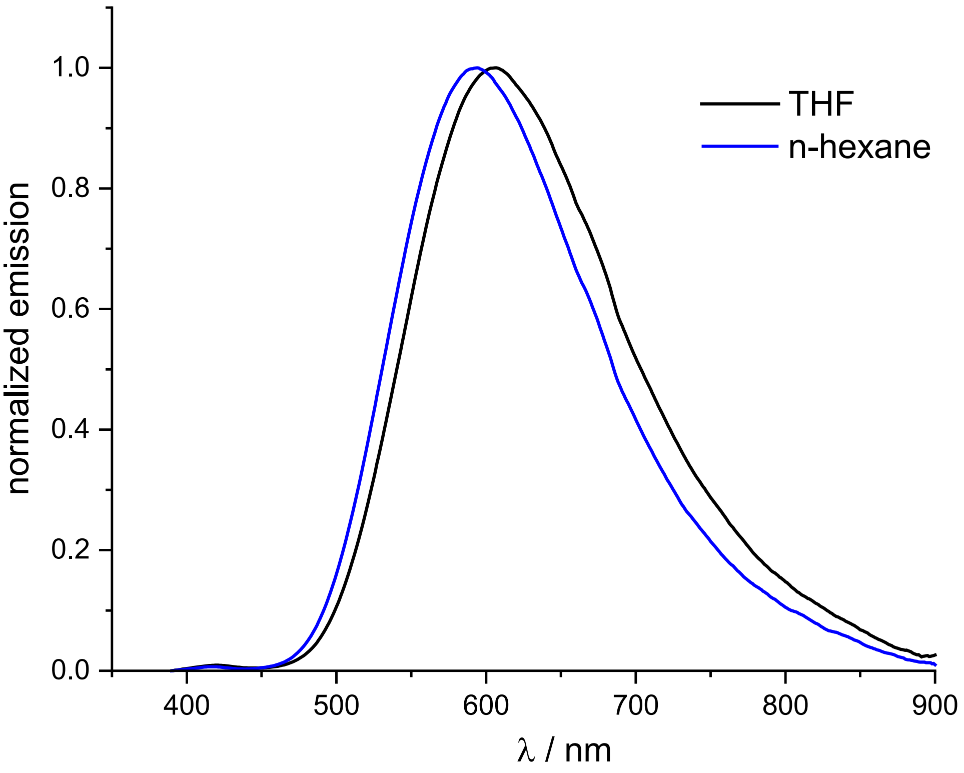

Figure S72. Emission spectra of $\mathbf{1}^{Mes*}$ in different solvents (THF, *n*-hexane).

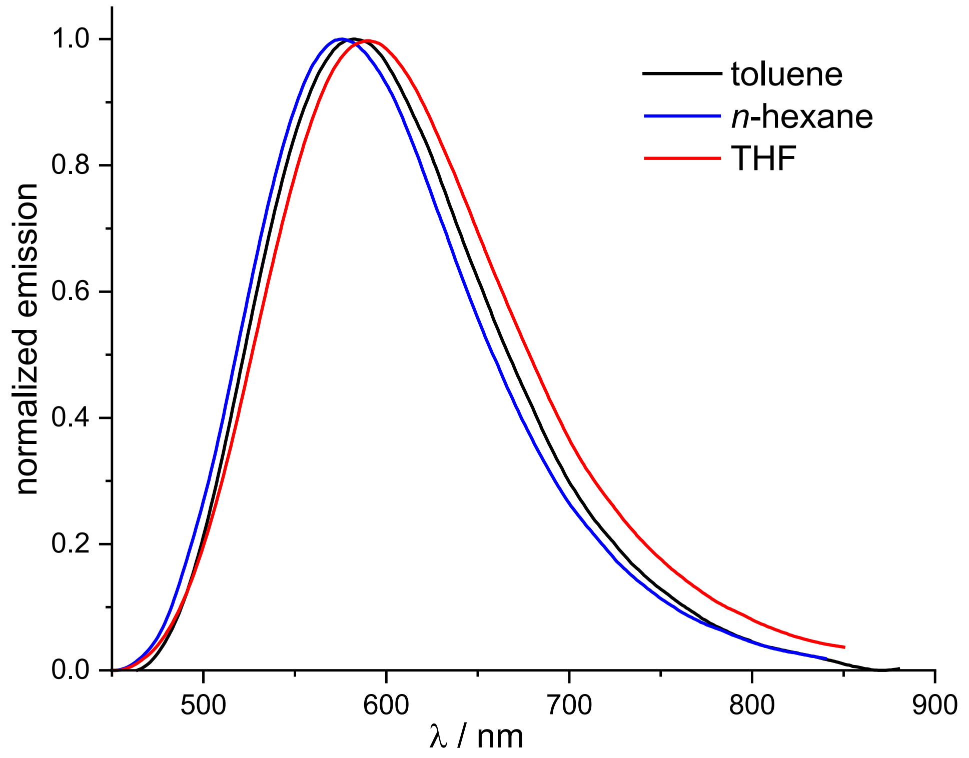

Figure S73. Emission spectra of **2^b^** in different solvents (THF, toluene, *n*-hexane).

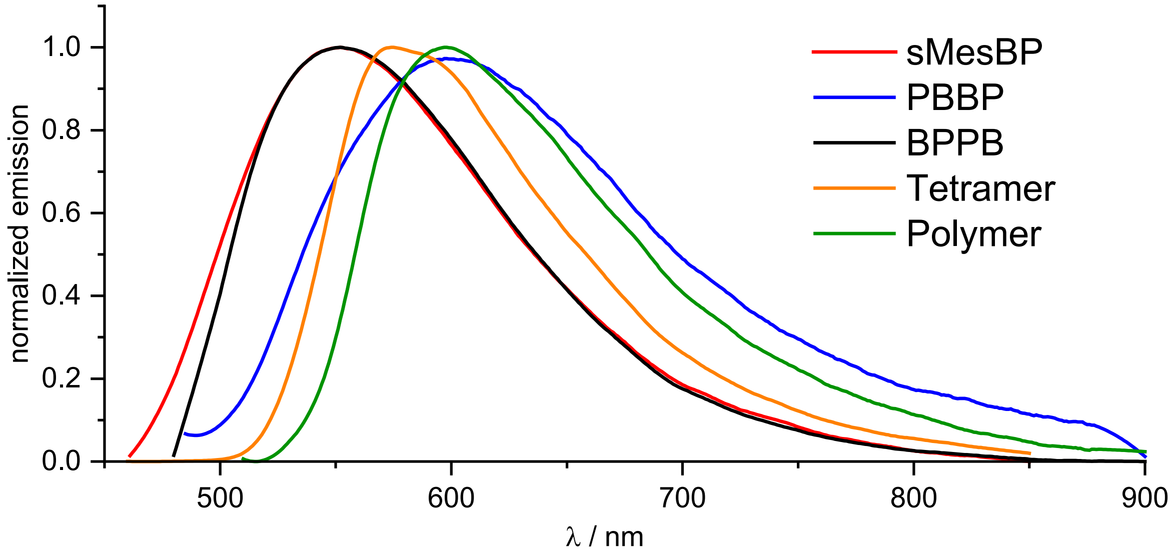

Figure S74. Solid state emission spectra of $\mathbf{1}^{\mathbf{Mes*}}$, $\mathbf{2}^{\mathbf{a}}$, $\mathbf{2}^{\mathbf{b}}$, **4** and **P1**.

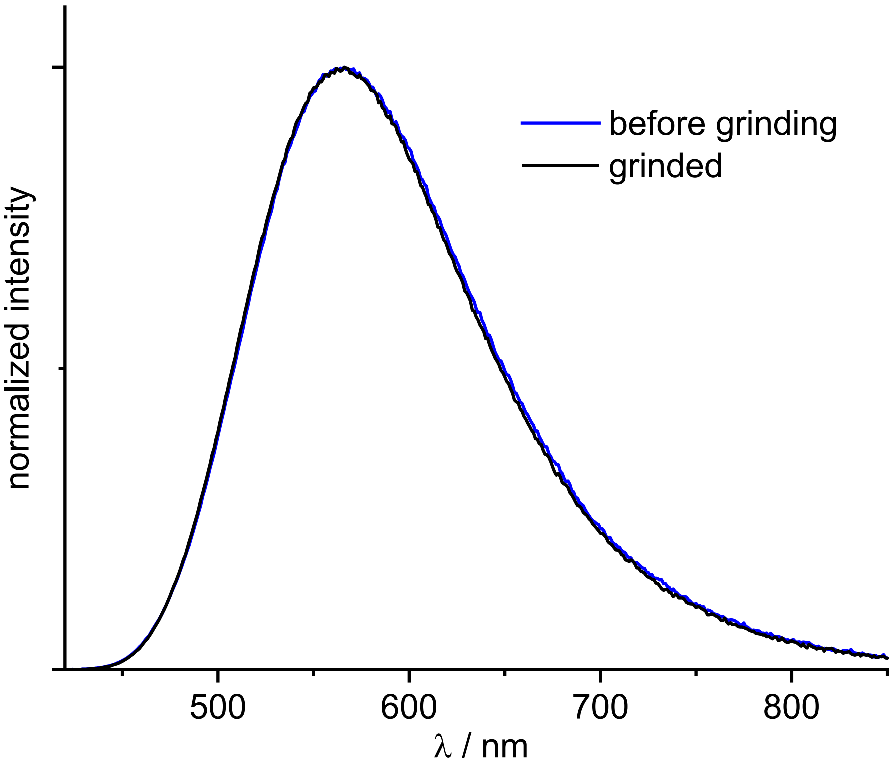

Figure S75. Solid state emission spectra of **1**^{Mes*}before and after grinding to investigate mechanochromic behaviour.

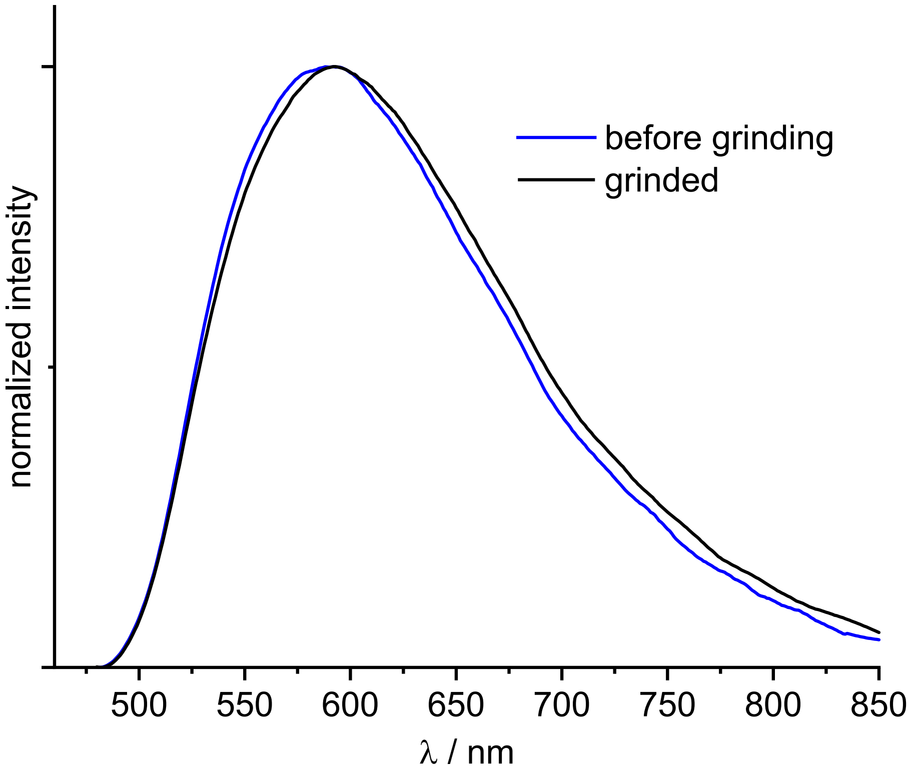

Figure S76. Solid state emission spectra of **2**[a] before and after grinding to investigate mechanochromic behavior.

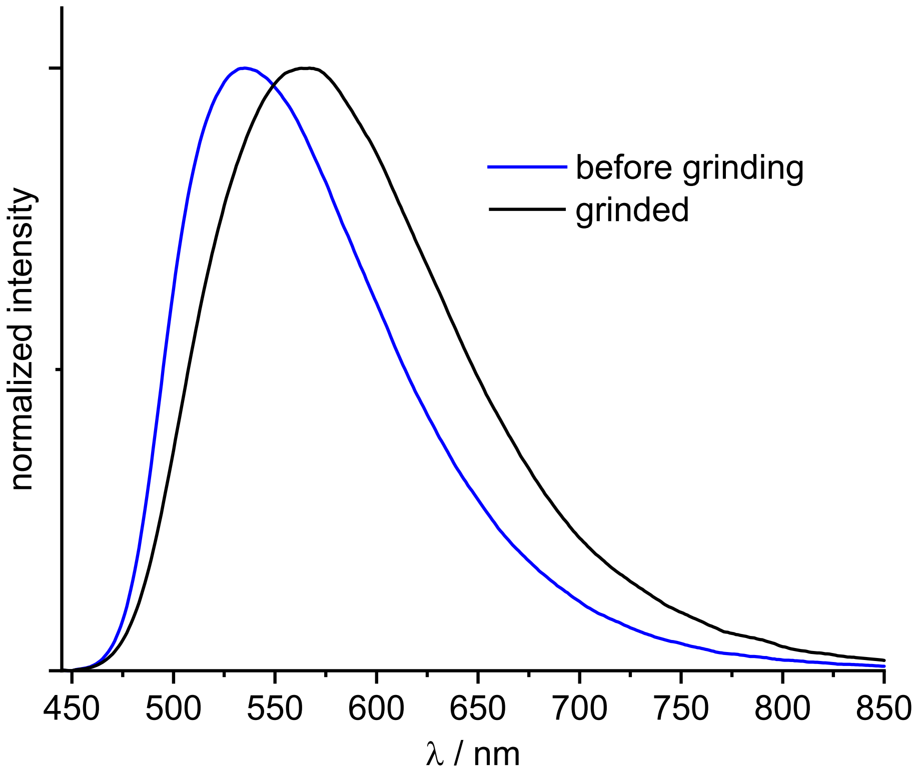

Figure S77. Solid state emission spectra of **2**[b] before and after grinding to investigate mechanochromic behavior.

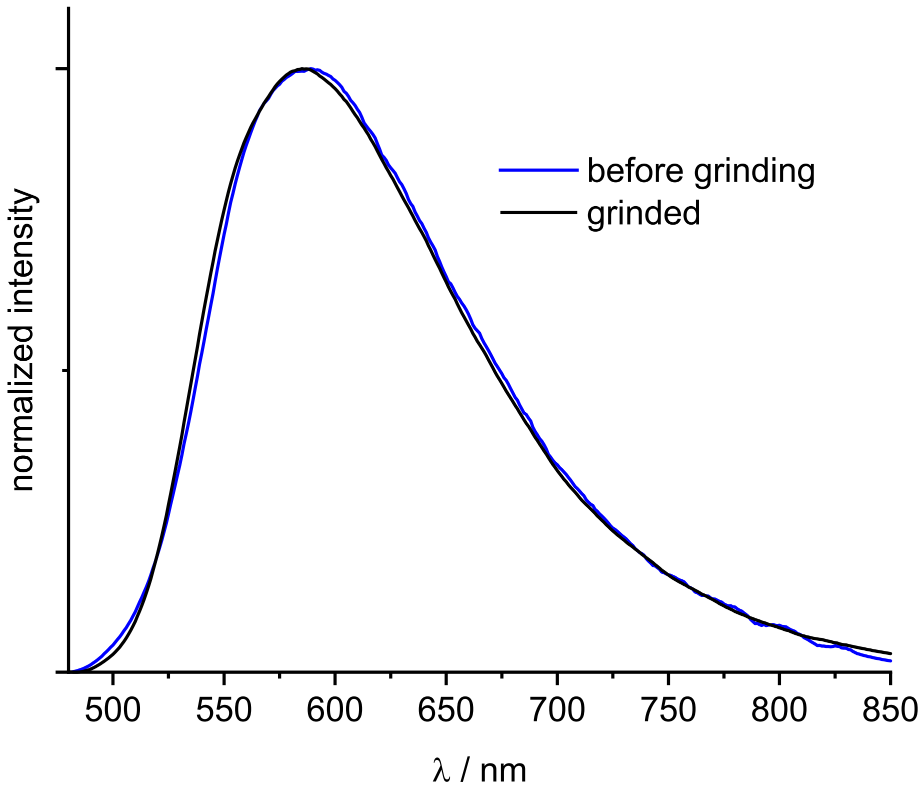

Figure S78. Solid state emission spectra of **4** before and after grinding to investigate mechanochromic behavior.

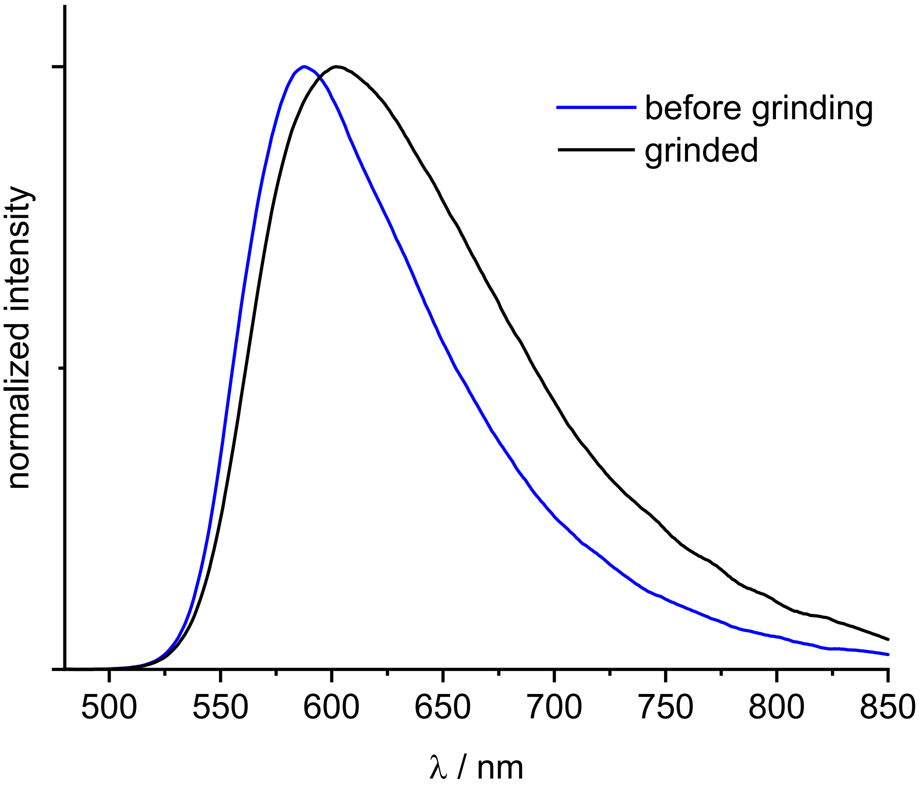

Figure S79. Solid state emission spectra of **P1** before and after grinding to investigate mechanochromic behaviour.

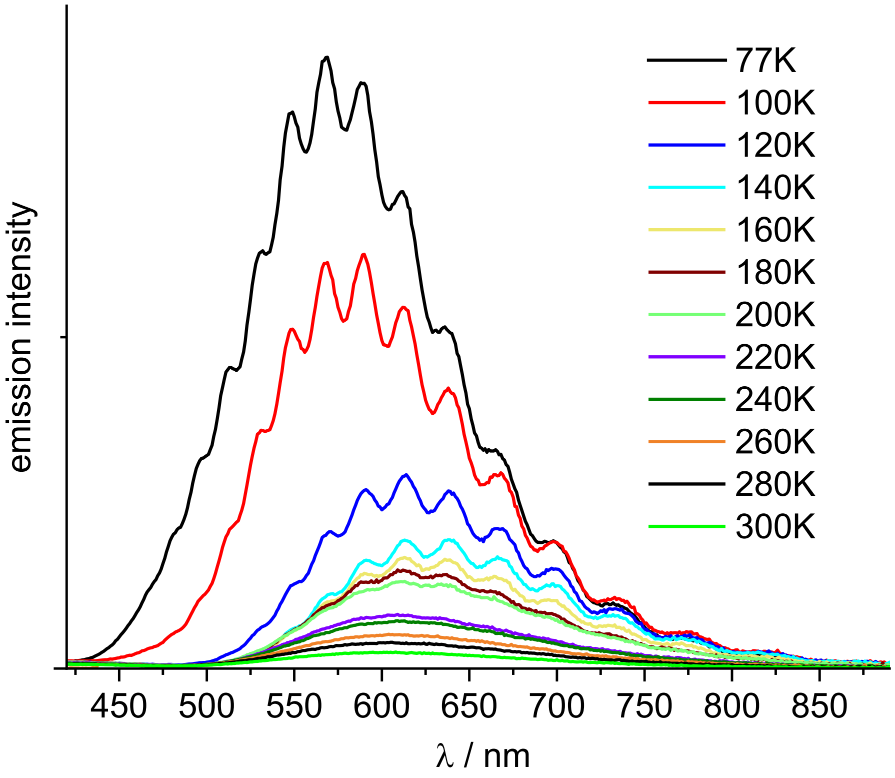

Figure S80. Emission spectra of $\mathbf{1}^{\mathbf{Mes*}}$ at different temperatures between 77 K and 300 K in 2-MeTHF.

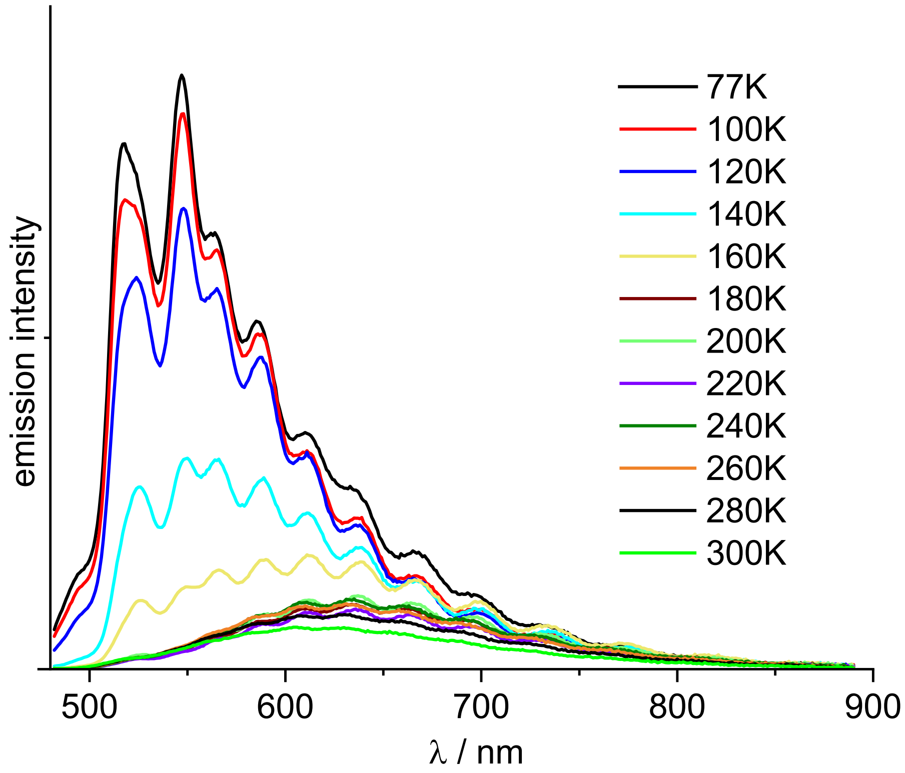

Figure S81. Emission spectra of **4** at different temperatures between 77 K and 300 K in 2-MeTHF.

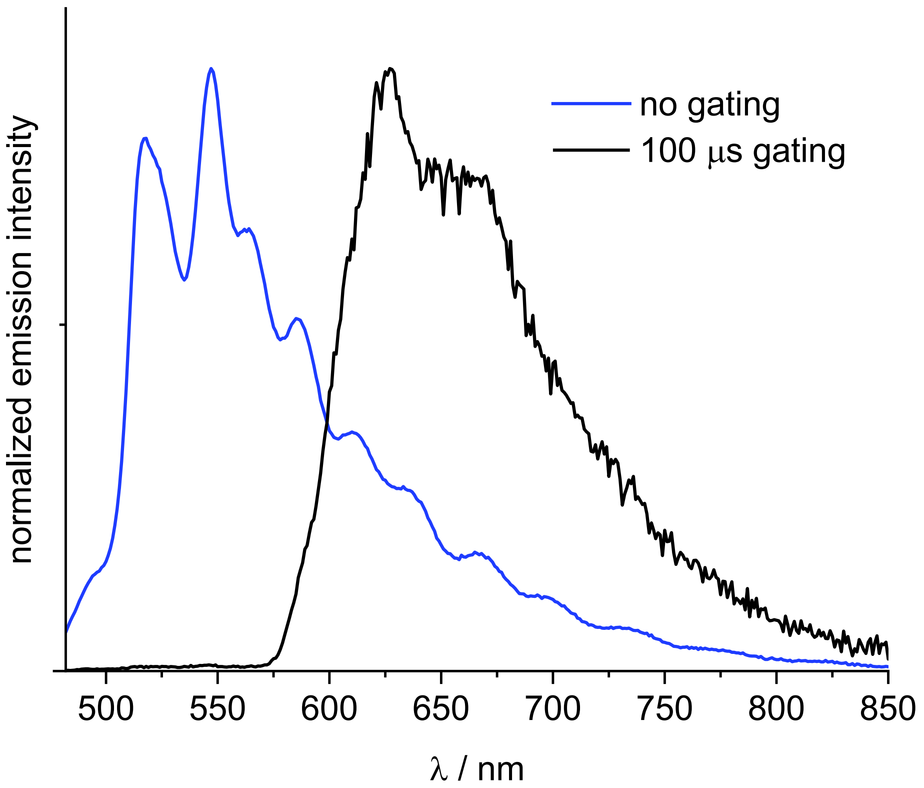

Figure S82. Emission spectra of **4** at 77 K in 2-MeTHF. The blue signal is the overall emission, and the black signal is the 100 μs time gated phosphorescence.

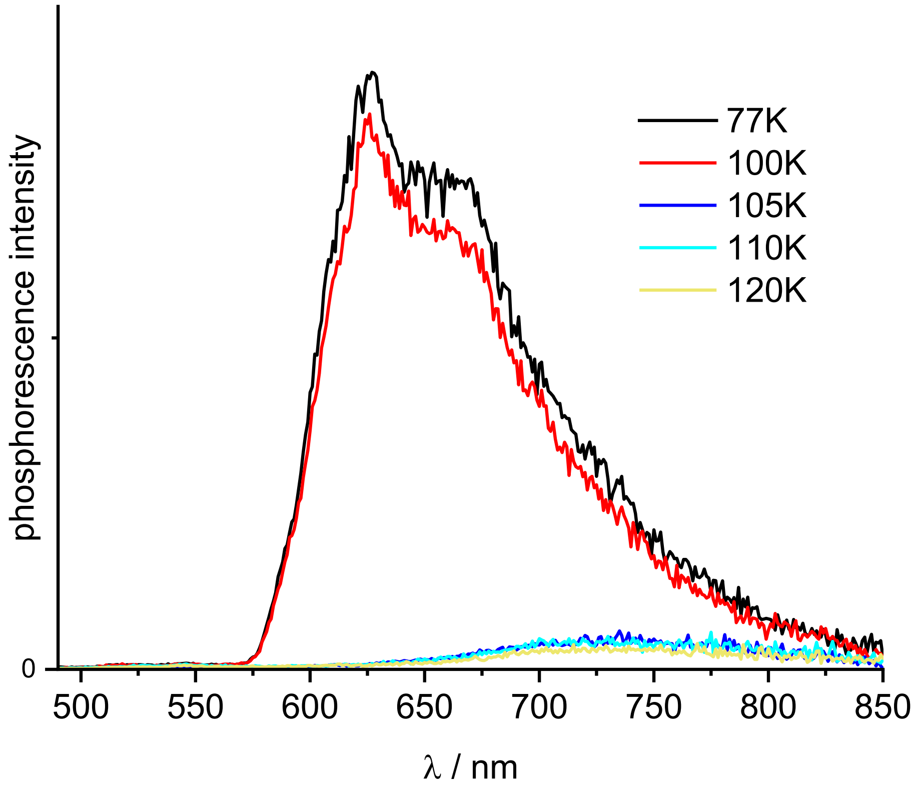

Figure S83. Phosphorescence spectra of **4** at different temperatures between 77 K and 100 K with 100 μs time gated measurement.

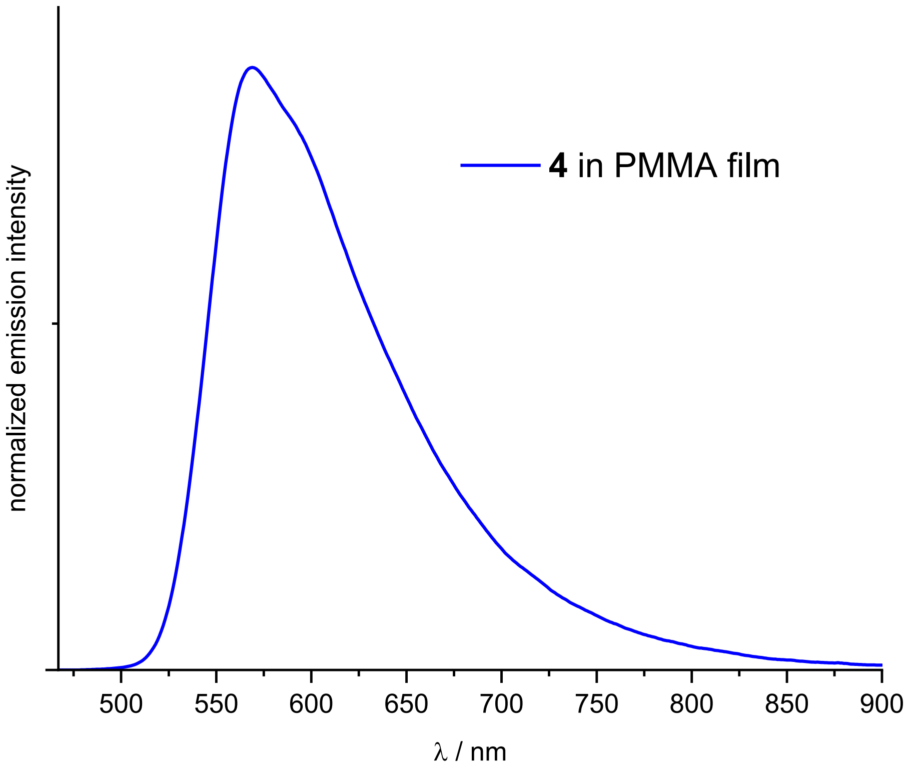

Figure S84. Emission spectra of **4** as PMMA-film (60 mg PMMA and 0.3 mg of **4** (evaporated from 1 mL THF)).

Table S4. UV-vis absorption and fluorescence emission data of $\mathbf{1}^{\mathbf{Mes}}$, $\mathbf{1}^{\mathbf{Tip}}$, $\mathbf{1}^{\mathbf{Mes*}}$, dimers $\mathbf{2}^{\mathbf{a}}$, $\mathbf{2}^{\mathbf{b}}$, tetramer **4**, and the polymerization product **P1** measured in THF, solid state and aggregation (in water/THF).

Compound	λ_{abs} [nm]	λ_{em} [nm]	λ_{em} [nm] (solid)	λ_{em} [nm] (aggr.)	Φ_{fl}-THF [a]	Φ_{fl}-solid-state [a]	Stokes shift (THF) [cm^{-1}]	τ_{fl} [ns]
$\mathbf{1}^{\mathbf{Mes}}$	368	–	–	–	–	–	–	–
$\mathbf{1}^{\mathbf{Tip}}$	372	544	–	–	0.02	–	8499	<1
$\mathbf{1}^{\mathbf{Mes*}}$	374	603	552	582	0.06	0.21	10154	<1
$\mathbf{2}^{\mathbf{a}}$	423	628	598	593	0.14	0.09	7717	<1
$\mathbf{2}^{\mathbf{b}}$	432	591	552	528	0.22	0.30	6228	2.1
**4**	474	620	574	568	0.10	0.27	4968	<1
**P1**	490	595	597	591	0.15	0.21	3601	<1

[a] Fluorescence quantum yield determined with an integration sphere.

1.7. GPC-Traces

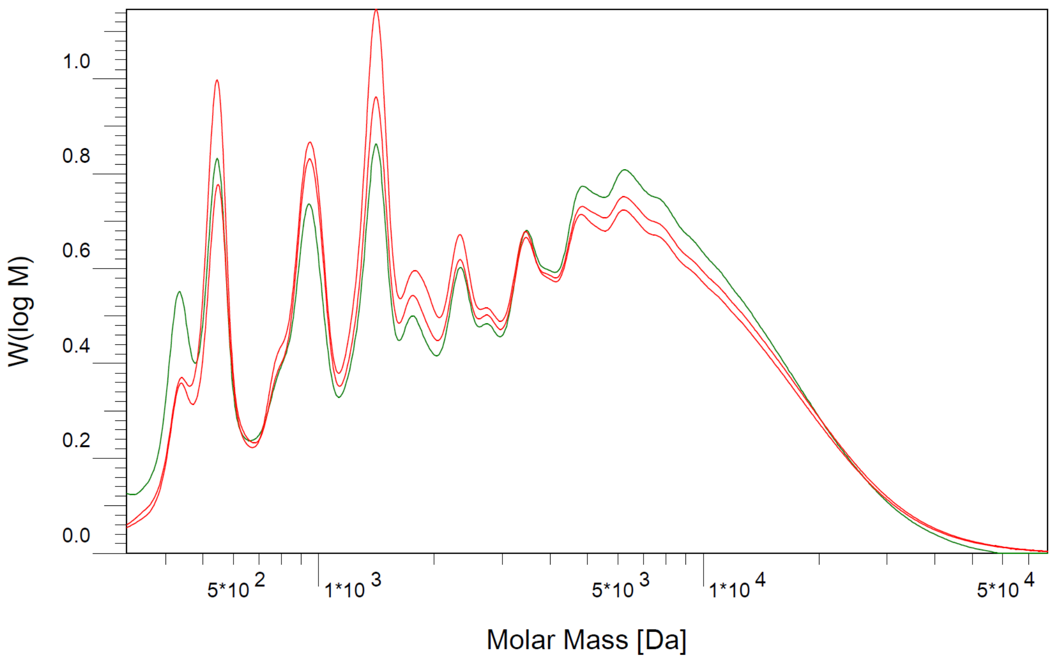

Figure S85. GPC trace of the soluble fraction from the reaction of **6** with **9** (UV-vis detector in red and RID green).

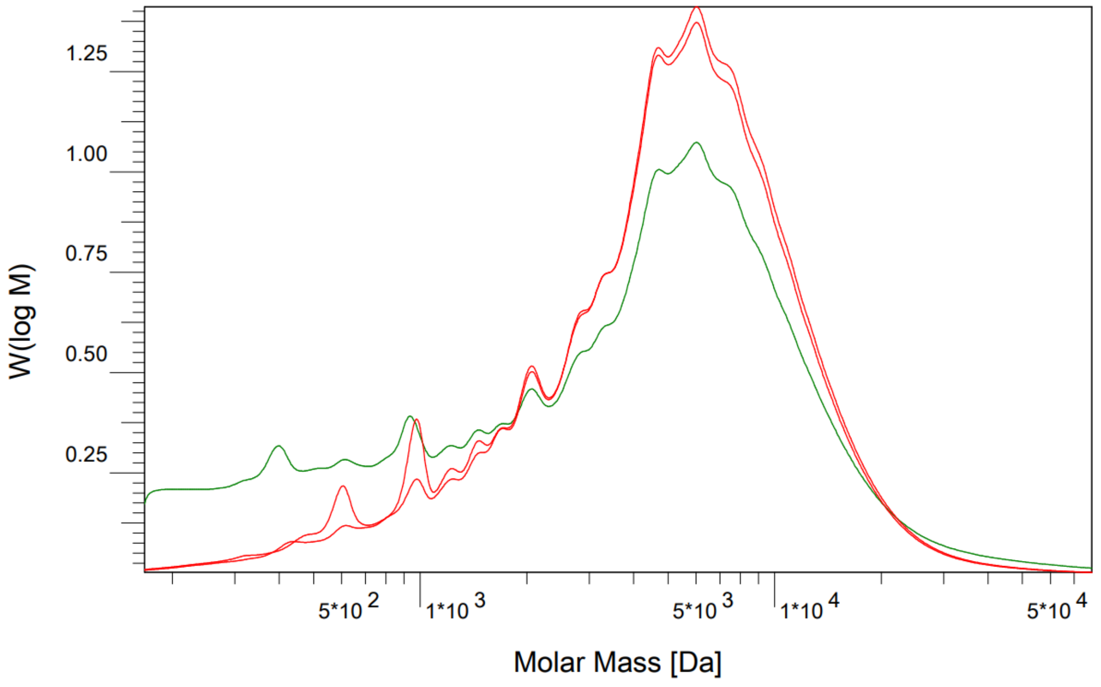

Figure S86. GPC trace of BP-PPV (**P1**) (UV-vis detector in red and RID green).

1.8. Cyclic Voltammetry

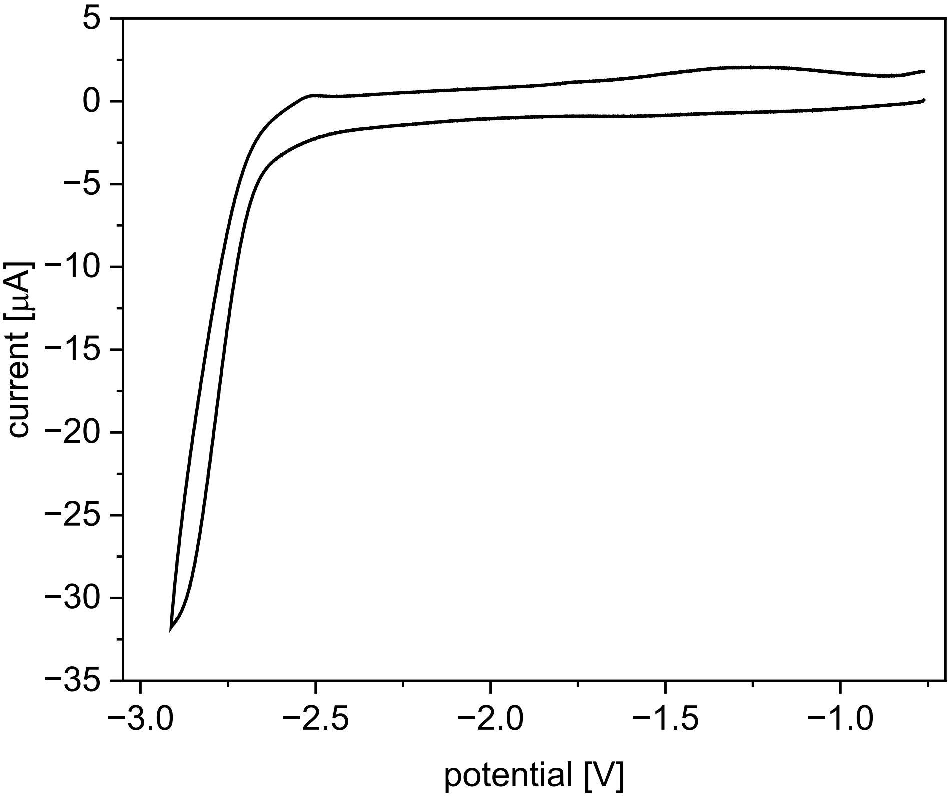

Figure S87. Cyclic voltammograms of **1**$^{\mathbf{Mes*}}$ in THF vs. $[Cp_2Fe]^{0/+}$ (scan-rate: 150 mVs^{-1}).

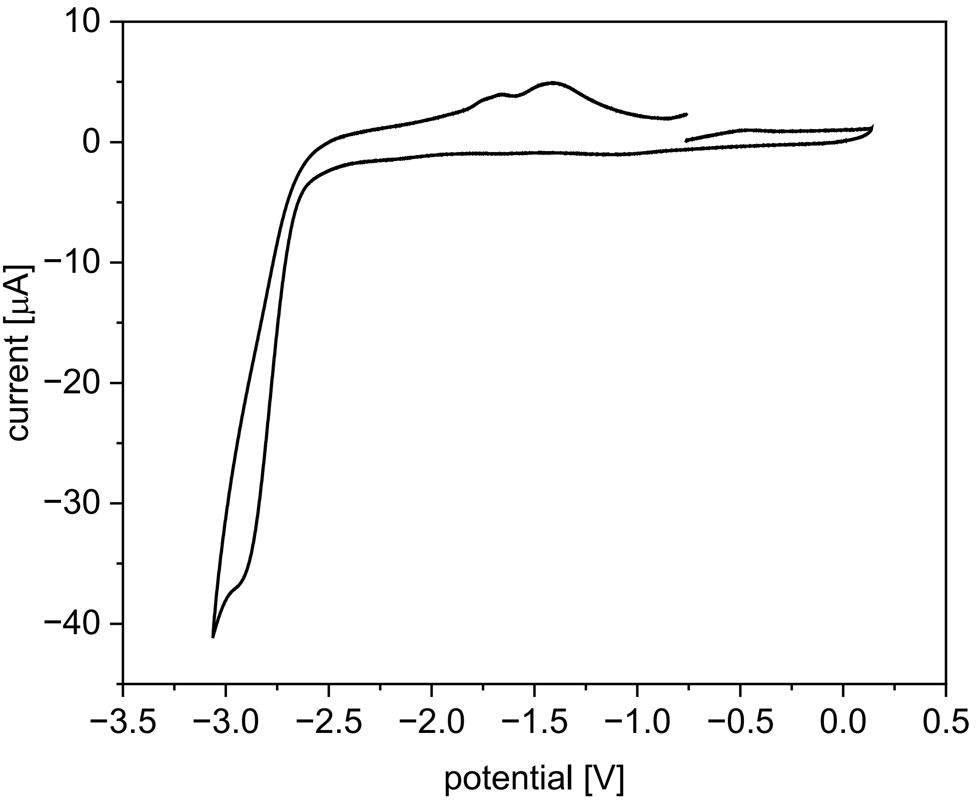

Figure S88. Cyclic voltammograms of **1**^{Mes*} in THF vs. $[Cp_2Fe]^{0/+}$ (scan-rate: 150 mVs^{-1}). At low potential (–2.8 V) an irreversible reduction event close to the THF limit was observed. The signal between –1.2 and –1.8 V can be assigned either to the product of the irreversible reduction of **1**^{Mes*} or the degradation due to overreduction of THF at low potentials.

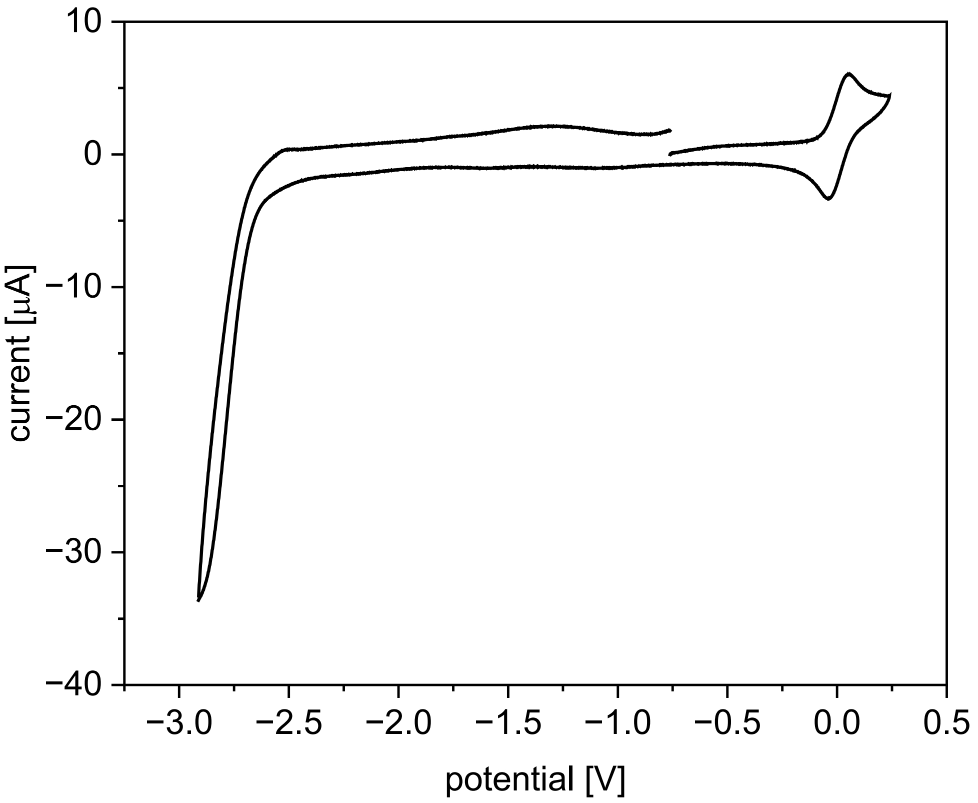

Figure S89. Cyclic voltammograms of **1**^{Mes*} in THF vs. $[Cp_2Fe]^{0/+}$ (scan-rate: 150 mVs^{-1}). (Ferrocene added in this figure).

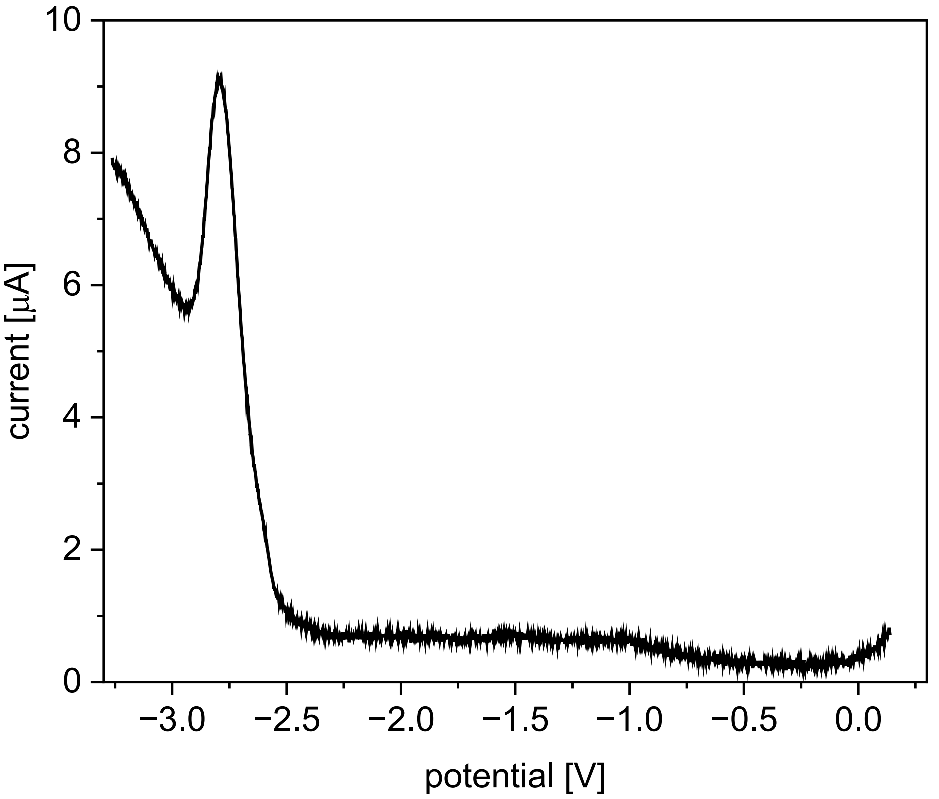

Figure S90. Square-wave voltammogram of **1**^{Mes*} in THF (vs. $[Cp_2Fe]^{0/+}$), 20 MHz, 2.0 mV step rate. The square-wave reveals a reduction event close to the limit of THF.

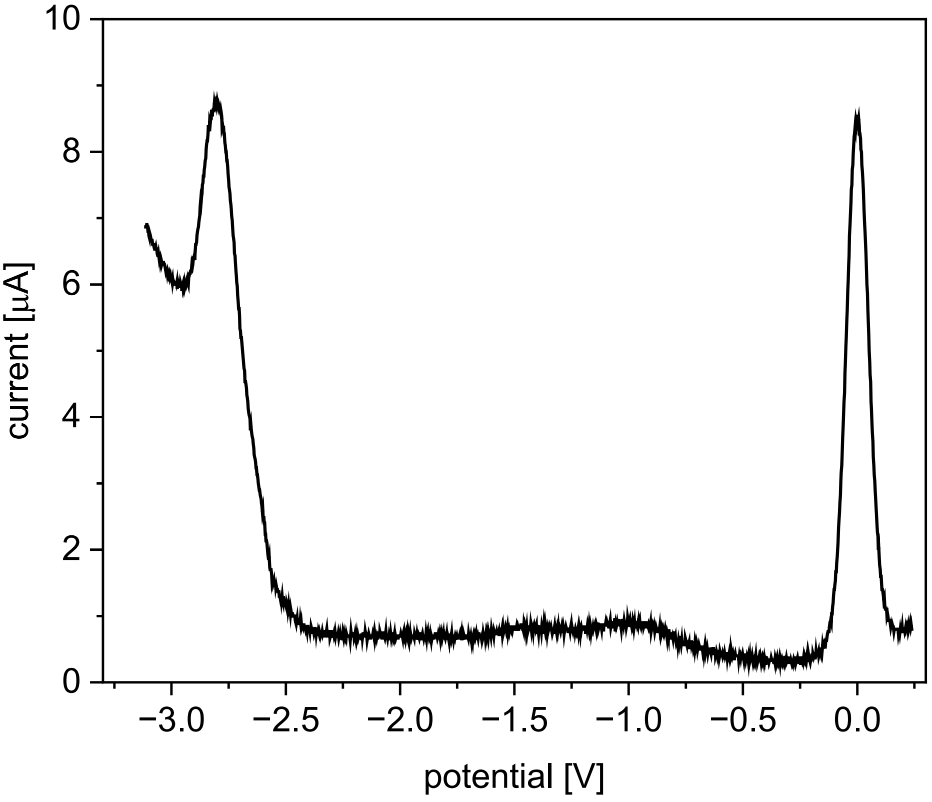

Figure S91. Square-wave voltammogram of **1**$^{\mathbf{Mes*}}$ in THF (vs. $[Cp_2Fe]^{0/+}$), 20 MHz, 2.0 mV step rate (with ferrocene).

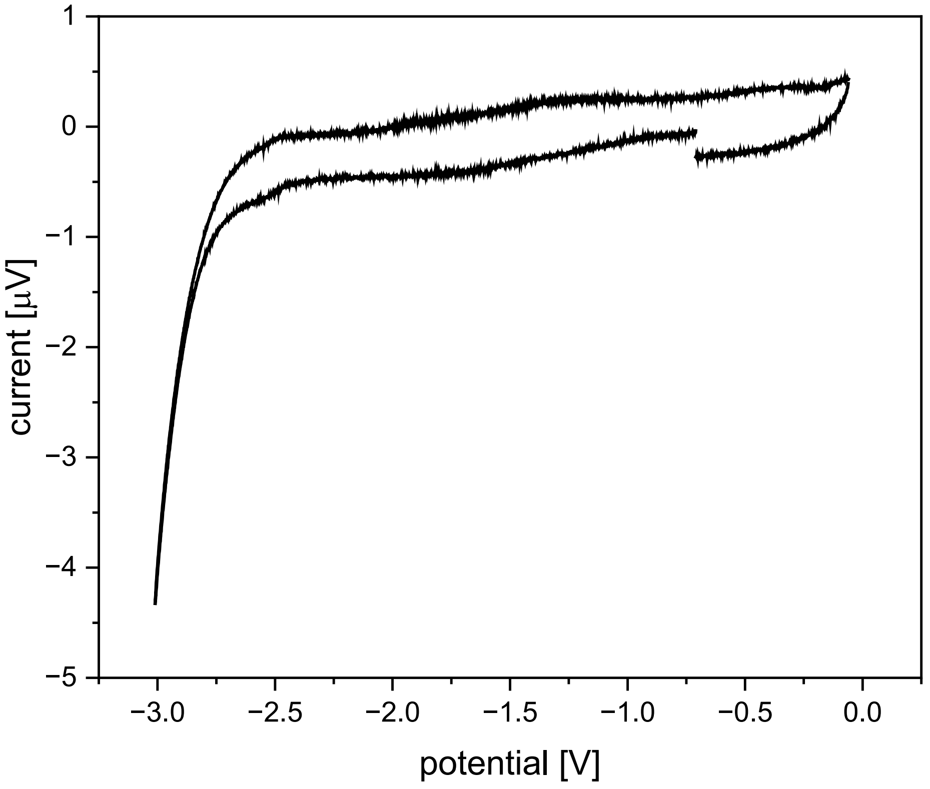

Figure S92. Cyclic voltammograms of **4** in THF vs. $[Cp_2Fe]^{0/+}$ (scan-rate: 150 mVs^{-1}).

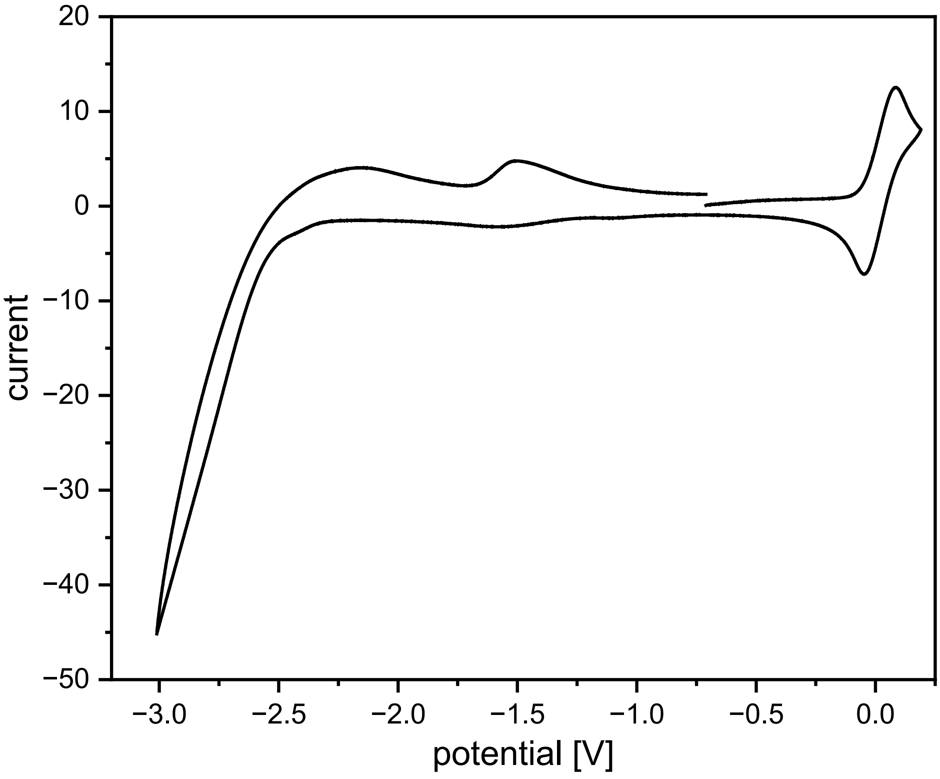

Figure S93. Cyclic voltammograms of **4** in THF vs. $[Cp_2Fe]^{0/+}$ (scan-rate: 150 mVs^{-1}). Ferrocene was added to this measurement. The signal between –1.2 and –1.8 V can be assigned either to the product of the irreversible reduction of **4** or the degradation due to overreduction of THF at low potentials.

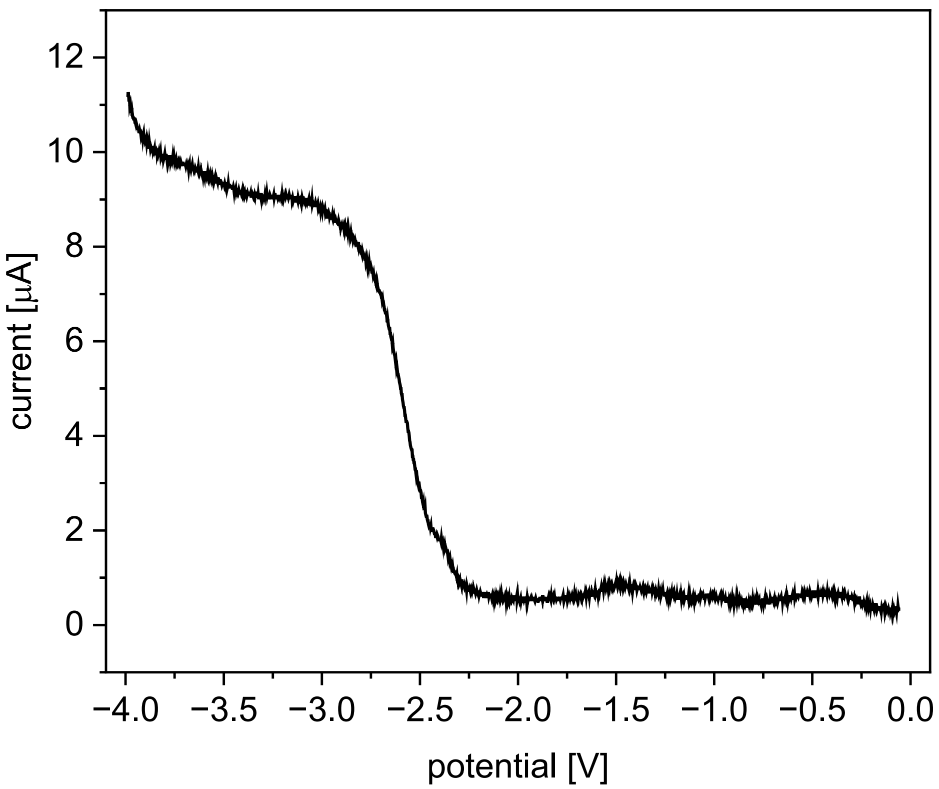

Figure S94. Square-wave voltammogram of **4** in THF (vs. $[Cp_2Fe]^{0/+}$), 20 MHz, 2.0 mV step rate. The voltammogram indicates a reduction event at –3.0 V which is superimposed by the reduction event of THF.

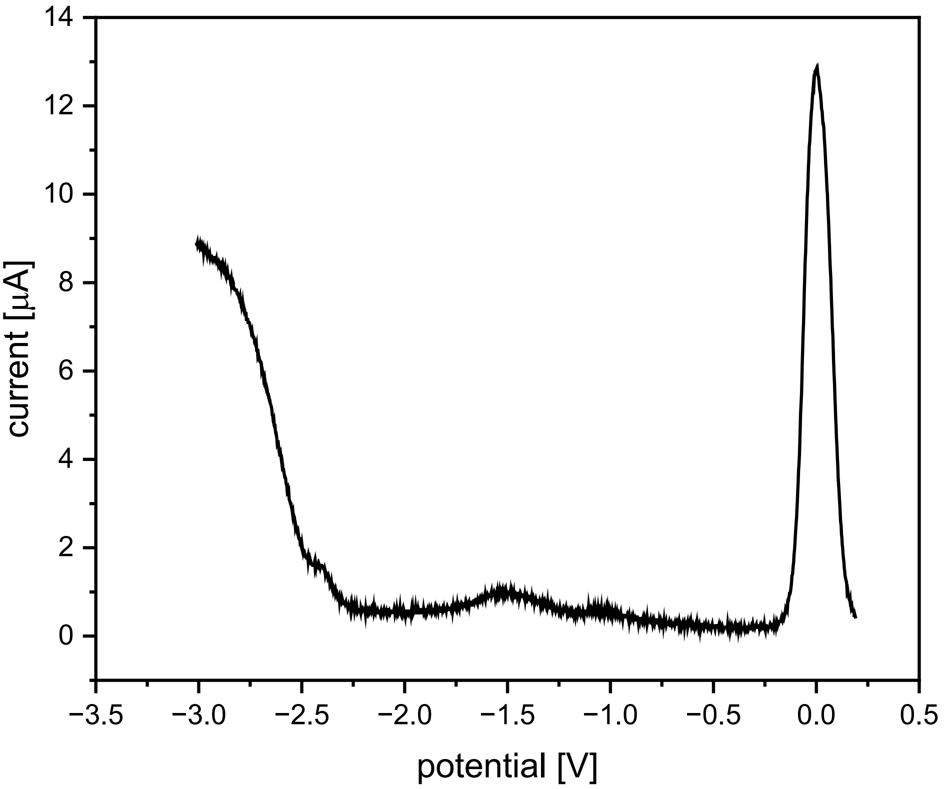

Figure S95. Square-wave voltammogram of **4** in THF (vs. $[Cp_2Fe]^{0/+}$), 20 MHz, 2.0 mV step rate (with ferrocene added).

1.9. Thermogravimetric Analysis (TGA)

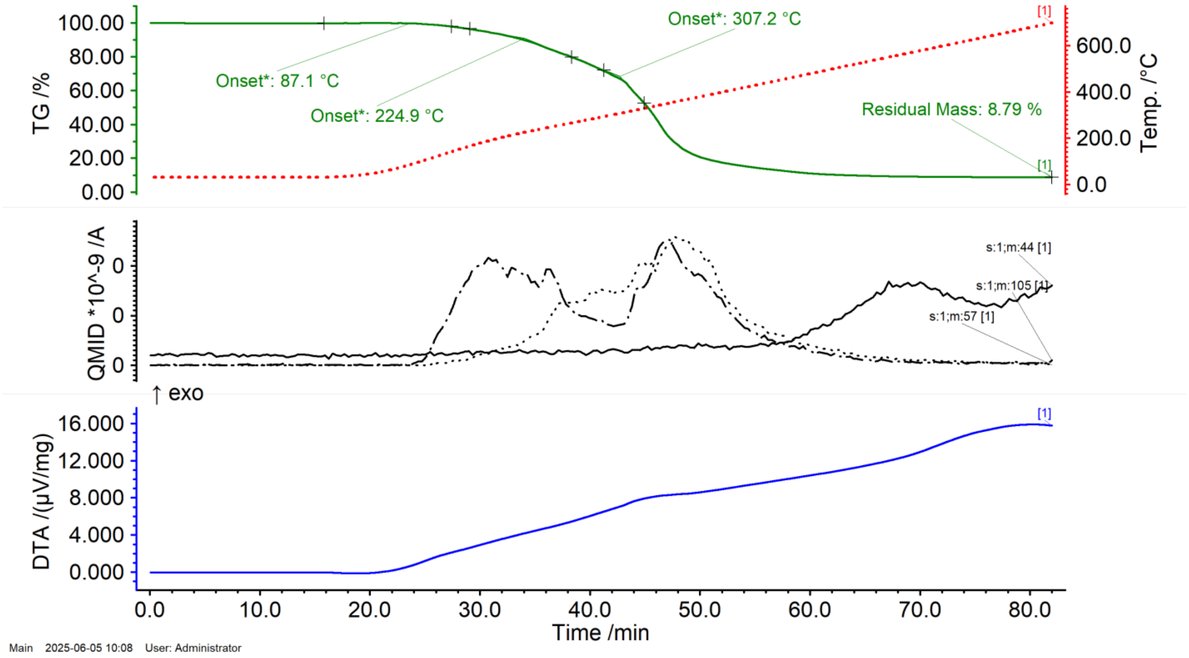

Figure S96. TG curve of **2**[a] measured under argon atmosphere (70 mL / min) with a heating rate of 10 K / min from 30 to 700 °C equipped with IR and MS detector. The TGA revealed several degradation processes starting at 87.1 °C.

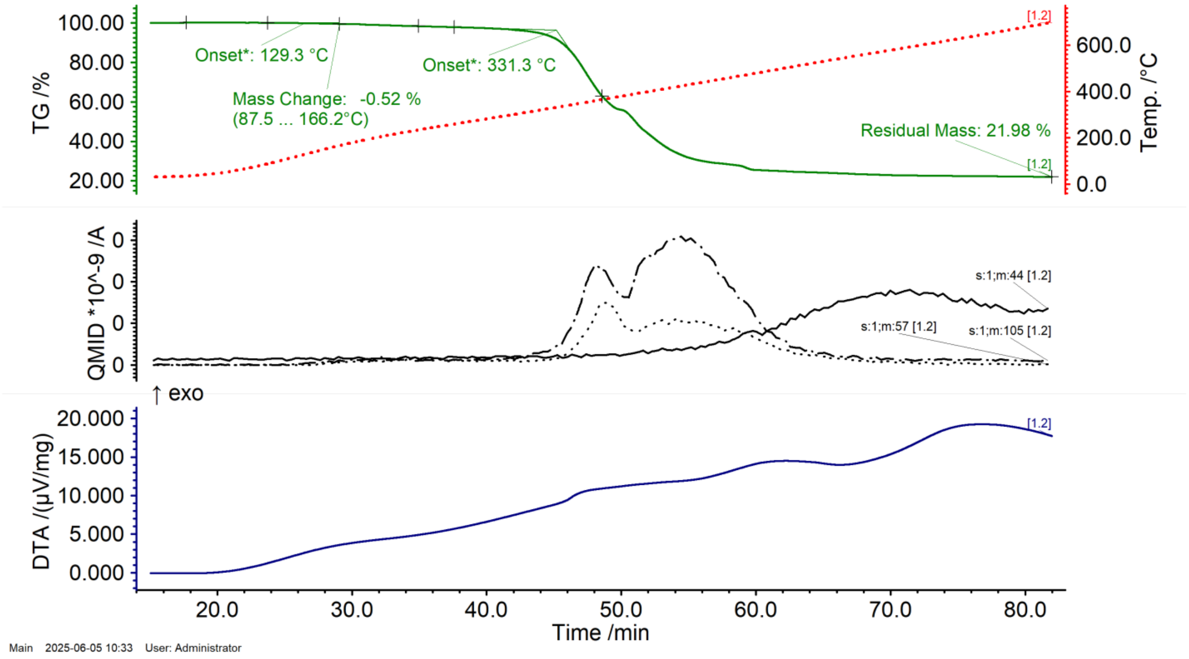

Figure S97. TG curve of **4** measured under argon atmosphere (70 mL / min) with a heating rate of 10 K / min from 30 to 700 °C equipped with IR and MS detector. The TGA revealed several degradation processes starting at 129.3 °C with a small mass change of 0.52 % and a strong mass change starting at 331.3 °C.

1.10. Dynamic light scattering (DLS)

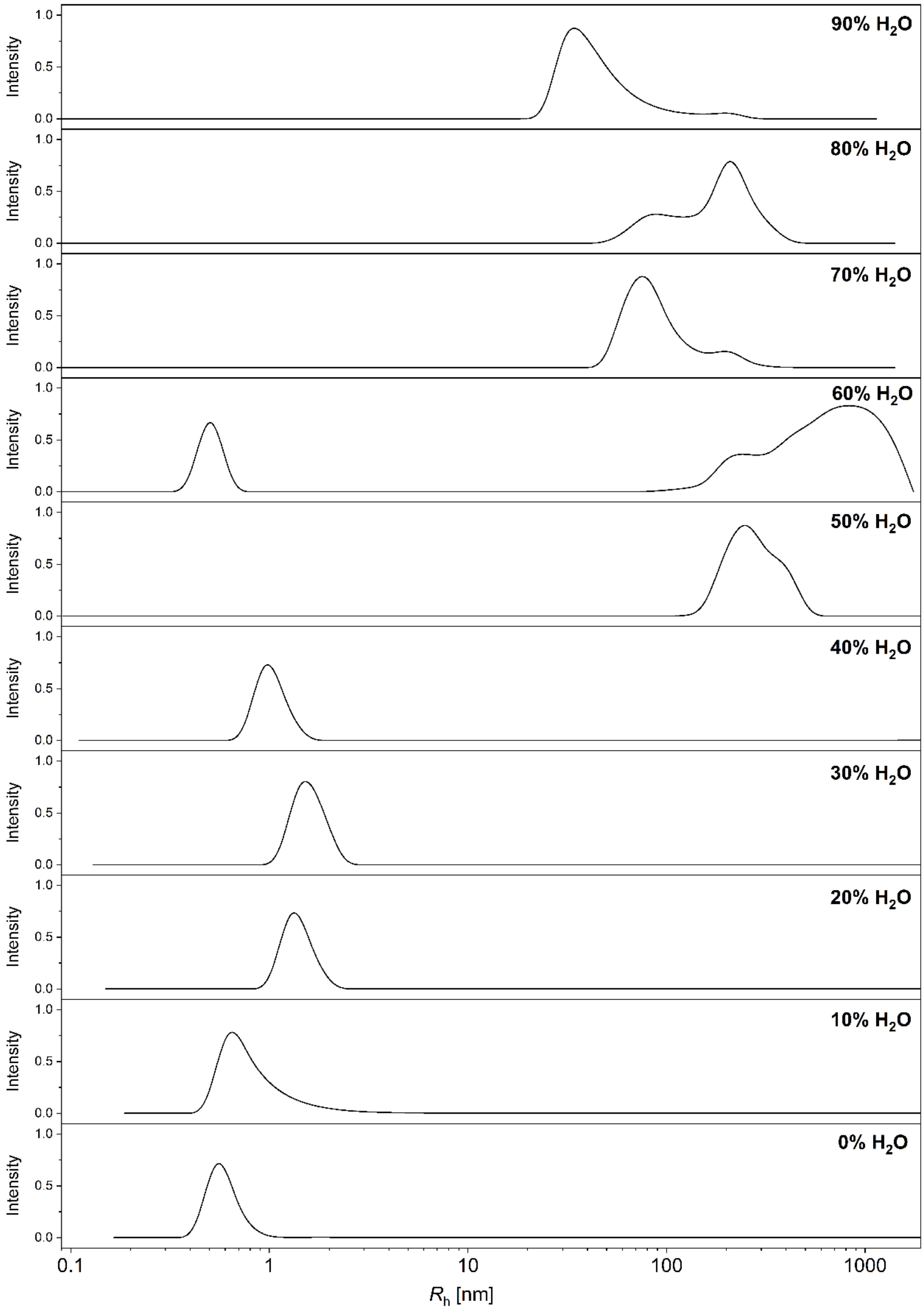

Figure S98. Mass weighted distribution (log) of aggregates of **4** in THF / water mixtures (conc. 5 · 10–5 M) at different water ratios (0 – 90 %).

2. Computational Information

DFT geometry optimizations were carried out with the Gaussian 16, Revision C.01 program package[11] using the ωB97X-D[12] functional in combination with the 6-31G(d)[13] basis set in gas phase. Vertical singlet excitations were calculated by means of time-dependent DFT with the program ORCA 5[14] using the ωB97X-D3[15] functional with optimal tuned ω parameters,[16] and the def2-SVP[17] basis set with the CPCM[18] solvation model mimicking tetrahydrofuran (ε = 7.25) as solvent. Wiberg[19] (WBO) and Mayer bond orders (MBO)[20] were computed using Gaussian 16, Revision C.01 program package[11] and Multiwfn[21] with ωB97X-D/6-31G(d)[12,13].

Table S5. Vertical S_1 energies from TD-DFT calculations (B-Xyl, P-Xyl*) using ω_TB97X-D3/def2-SVP, CPCM (THF).

Compound	Geometry	λ / nm	Oscillator strength *f*	Orbital contributions	$\lvert c\rvert^2$ / %
**1^{Xyl}**	**S_0**	339	0.284	**HOMO → LUMO**	94.9
**1^{Dip}**	**S_0**	347	0.291	**HOMO → LUMO**	95.9
**1^{Xyl*}**	**S_0**	350	0.609	**HOMO → LUMO**	97.7
**1^{Xyl*}**	**pyr-S_1**	557	0.143	**HOMO → LUMO**	97.4
**1^{Xyl*}**	**pyr-T_1**	501	0.279	**HOMO → LUMO**	97.5
**1^{Xyl*}**	**TICT-S_1^x**	1820	0.000	**HOMO → LUMO**	97.2
**1^{Xyl*}**	**TICT-T_1**	932	0.056	**HOMO → LUMO**	97.3
**$2^{a'}$ (Xyl, Xyl*)**	**S_0**	407	1.303	**HOMO → LUMO**	93.8
**$2^{a'}$ (Xyl, Xyl*)**	**pyr-S_1**	596	0.311	**HOMO → LUMO**	94.3
**$2^{a'}$ (Xyl, Xyl*)**	**pyr-T_1**	562	0.583	**HOMO → LUMO**	95.0
**$2^{a'}$ (Xyl, Xyl*)**	**TICT-S_1^x**	2195	0.003	**HOMO → LUMO**	93.7
**$2^{a'}$ (Xyl, Xyl*)**	**TICT-T_1**	865	0.160	**HOMO → LUMO**	93.8
**$2^{b'}$ (Xyl, Xyl*)**	**S_0**	403	1.308	**HOMO → LUMO**	91.7
**$2^{b'}$ (Xyl, Xyl*)**	**pyr-S_1^x**	571	0.273	**HOMO → LUMO**	90.5
**$2^{b'}$ (Xyl, Xyl*)**	**pyr-T_1**	539	0.475	**HOMO → LUMO**	92.2
**$2^{b'}$ (Xyl, Xyl*)**	**TICT-S_1^x**	1847	0.000	**HOMO → LUMO**	93.5
**$2^{b'}$ (Xyl, Xyl*)**	**TICT-T_1**	1001	0.076	**HOMO → LUMO**	93.3

^{x}Imaginary frequency.

To get deeper insight into the bonding situation, we performed DFT calculations on model systems for the three monomers where we omitted the *para*-substituents at the *P*-aryl substituents for computational convenience, i. e., *m*-xylyl (Xyl) instead of Mes, 2,6-diisopropylphenyl (Dip) instead of Tip, and 2,6-di-*tert*-butylphenyl (Xyl*) to mimic Mes*. Our DFT calculations support the experimentally observed planarization of the phosphorus center with growing steric demand between the Tip and Mes* substituent at the P-atom (see Figure 2 in the main text). Between **1**$^{\mathbf{Xyl}}$ ($S_\angle$ = 332.0°) and **1**$^{\mathbf{Dip}}$ ($S_\angle$ = 331.9°) no significant change in geometry was predicted by our calculations. Only for **1**$^{\mathbf{Xyl*}}$ ($S_\angle$ = 360.0°) a complete planarization was predicted. With increasing bulkiness of the substituents, the B–P bond length decreases slightly from Xyl (B−P = 1.882 Å) to Dip (B−P = 1.878 Å) and strongly to Xyl* (B−P = 1.813 Å). The calculated frontier orbitals indicate an increase in π-bond character, consistent with a transition from a B−P single to a B=P double bond with donor(P)−acceptor(B)-type π-bonding. Whereas the HOMO of **1**$^{\mathbf{Xyl}}$ still has significant phosphorus lone pair character – to some extent delocalized into the π-system of the phenyl group attached to it – and the LUMO has the largest contribution from the boron's vacant p-orbital and the phenyl group at this center, the frontier orbitals of **1**$^{\mathbf{Xyl*}}$ both clearly show the characteristics of extended π-orbitals delocalized over the entire Ph-B=P-Ph backbone of the molecule. The HOMO of **1**$^{\mathbf{Xyl*}}$ is mainly characterized as a B=P-bonding π-orbital, approximately equally shared between both atoms. Additional π-type contributions from both phenyl substituents are clearly visible. The LUMO of **1**$^{\mathbf{Xyl*}}$ is fully delocalized and antibonding with respect to the B=P bond, having a nodal plane between both heteroatoms. The characteristics of the frontier orbitals of **1**$^{\mathbf{Dip}}$ are intermediate between those of **1**$^{\mathbf{Xyl}}$ and **1**$^{\mathbf{Xyl*}}$. The HOMO and the LUMO of **1**$^{\mathbf{Xyl}}$, **1**$^{\mathbf{Dip}}$, and **1**$^{\mathbf{Xyl*}}$ are also involved in the major absorption band of their UV-vis spectra (*vide infra*). Therefore, the character of this transition changes from an n(P)→p(B)* charge transfer for **1**$^{\mathbf{Xyl}}$ and **1**$^{\mathbf{Dip}}$ to a π−π* transition for **1**$^{\mathbf{Xyl*}}$ involving the entire backbone of the molecule. Based on these results, Mes* proved to be the best choice for planarization of the phosphinoborane **1**$^{\mathbf{Mes*}}$ and thus a promising substituent for the preparation of conjugated oligomers and polymers.

To better understand the emission properties of the compounds presented herein, we performed additional TD-DFT computations. This includes the optimizations of electronically excited singlet and triplet states. The latter one was done because in some cases imaginary frequencies of the excited singlet geometries could not be eliminated so that the usage of the triplet geometries can be beneficial. The triplet geometries are similar to those of the S1 geometries but have no imaginary frequencies. The S_1 energies in the triplet geometries are thus similar to the energies in the S_1 geometries. We found two minimum geometries for the first excited states (S_1). The most favorable one is a TICT state where the molecule is twisted by about 90° about the B−P bond with respect to the S_0 geometry. However, emission from this state is predicted to occur in the NIR region. This excited state, if it is populated, probably undergoes nonradiative decay. We assume that the second minimum, denoted as pyr-S_1 is responsible for the emission seen in the experimental spectrum. The computed vertical energy for **1**$^{\mathbf{Xyl*}}$ is 3.55 eV while the corresponding adiabatic energies from the S_0 minimum to the minima of pyr-S_1 and TICT-S_1 states are 2.97 eV and 2.31 eV (TICT-T_1: 2.56 eV), respectively. The vertical excitation of these S_1 states to the ground states are 2.23 eV and 0.68 eV (TICT-T_1: 1.33 eV). Overall, the TICT-S_1 (TICT-T_1) is more favorable than the pyramidal one because it lies about 0.66 eV (0.41 eV) below the pyr-S_1 state. The change in geometry is visualized in Figure 6 in the main text along with the corresponding energy levels for S_0 and S_1 In pyr-S_1 the phosphorus center is pyramidalized. The sum of C−P−C/B angles is 346° compared to $\Sigma_\angle$ = 360° in the minimum S_0 geometry. This deformation leads to a more pronounced charge transfer for the pyr-S_1 state which is also seen in the frontier orbitals (Figure S101). The π-bond is broken in pyr-S_1, as is indicated by the significantly increased B−P bond length of 2.044 Å (cf. S_0: 1.813 Å). On the other hand, the bonds to the adjacent phenyl groups are slightly shortened

(B−C: from 1.574 to 1.527 Å, P−C: from 1.812 to 1.799 Å). In the more favorable TICT-S_1 and TICT-T_1 geometries the BXylPh unit is twisted by about 90° around the B−P bond in comparison to the S_0 geometry. A nearly perpendicular arrangement is found for the BXylPh unit with respect to the PXyl*Ph fragment (TICT-S_1: 85.4°, TICT-T_1: 69.4°) while the ground state is completely planar (0.0°). Consequently, the B−P bond is elongated with 1.902 Å in the twisted S_1 geometry (TICT-T1: 1.915 Å). On the other hand, the B−C/P−C bonds to the adjacent phenyl groups are shortened. While for the S_0 geometry these bond lengths are 1.574 Å (B−C) and 1.812 Å (P−C), values of 1.556 Å (B−C) and 1.812 Å (P−C) are found for the TICT-S_1 geometry and values of 1.547 Å (B−C) and 1.795 Å (P−C) for the TICT-T_1 geometry.

In the following, $\mathbf{2^a}$ and $\mathbf{2^b}$ stand for the model systems $\mathbf{2^{a'}}$ **(Xyl, Xyl*)** and $\mathbf{2^{b'}}$ **(Xyl, Xyl*)**. For $\mathbf{2^{a'}}$ and $\mathbf{2^{b'}}$, like for $\mathbf{1^{Xyl*}}$, in the HOMOs of the S_0 geometries, the B-P bonds show bonding character, while in the LUMOs there are nodal planes, respectively (Figure S102 and S103). The HOMO represents a π-orbital that is delocalized over the whole system with strong contribution at the central benzene ring that shows nodal planes to the adjacent B and P atoms bound to it. The LUMO show antibonding character between the B and P centers while a bonding one is present between those heteroatoms and the adjacent phenyl groups. While the vertical excitations are mainly delocalized transitions, we find CT transitions for the pyramidal geometries of $\mathbf{2^{a'}}$ and $\mathbf{2^{b'}}$. In the S_0 geometry, HOMO and LUMO orbitals look comparable. The pyramidal ones show significant differences. $\mathbf{2^{b'}}$ has a HOMO in the pyr-S_1 geometry similarly to the HOMO in the ground state geometry, localized in the core of the molecule (Figure S103). The LUMO is localized in the right part of the molecule containing the boron center next to the pyramidal phosphorus. On the other hand, the HOMO of $\mathbf{2^{a'}}$ is localized on the right-hand side, containing the pyramidal P atom and the adjacent phenyl ring. In this case, the LUMO looks comparable to the LUMO in the S_0 geometry, which is mainly localized at the core of the molecule. A similar behavior is found for twisted S_1 geometries. Comparing the measured (Table 1 in the manuscript text) and calculated (Table S5) vertical emission energies shows a better agreement of the pyr-S_1 state ($\mathbf{1^{Xyl*}}$: 2.23 eV, $\mathbf{2^{a'}}$: 2.08 eV, $\mathbf{2^{b'}}$: 2.17 eV) with the experiment ($\mathbf{1^{Xyl*}}$: 2.06 eV, $\mathbf{2^{a'}}$: 1.97 eV, $\mathbf{2^{b'}}$: 2.10 eV) in all three cases. On the other hand, the TICT states lead to emission that are significantly bathochromically shifted to the NIR region ($\mathbf{1^{Xyl*}}$: 0.68 eV, $\mathbf{2^{a'}}$: 0.57 eV, $\mathbf{2^{b'}}$: 0.67 eV). We could not measure a second emission event either due to these transitions taking place in the far NIR region that is out of our detector range or to such states relaxing nonradiative to the ground state. The calculated frontier orbitals of $\mathbf{2^a}$ and $\mathbf{2^{b'}}$ indicate, as previously observed for $\mathbf{1^{Xyl*}}$, a π−π* transition from HOMO to LUMO. Similarly to $\mathbf{1^{Xyl*}}$, the HOMO and LUMO are delocalized over the entire π-system along the molecular chain, including the B=P moieties. In conjunction with the photophysical data, this indicates a pronounced conjugation across the oligomer backbone. This explains the experimental bathochromic shift of the absorption maxima of around 0.39 or 0.45 eV by going from $\mathbf{1^{Xyl*}}$ (374 nm / 3.32 eV) to $\mathbf{2^{a'}}$ (423 nm / 2.93 eV) or $\mathbf{2^{b'}}$ (432 nm / 2.87 V), respectively. In comparison, our computations gave similar results with bathochromic shifts of around 0.5 eV considering the respective vertical excitation energies of 3.55 eV (350 nm) for $\mathbf{1^{Xyl*}}$, 3.05 eV (407 nm) for $\mathbf{2^{a'}}$ and 3.08 eV (403 nm) for $\mathbf{2^{b'}}$.

a)

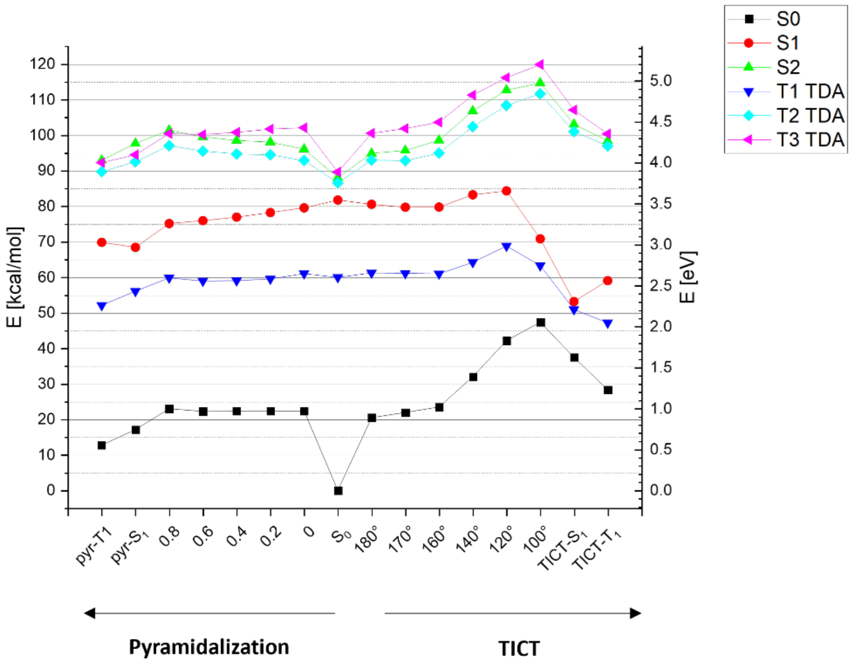

b)

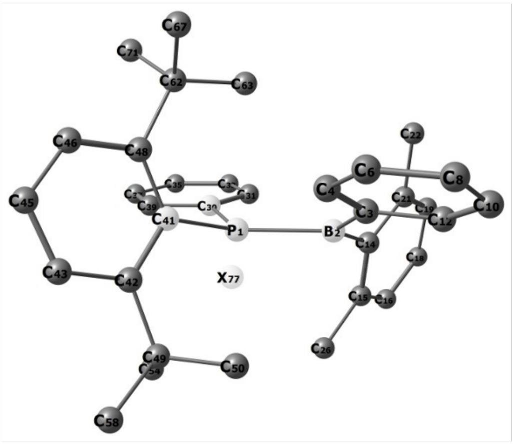

c)

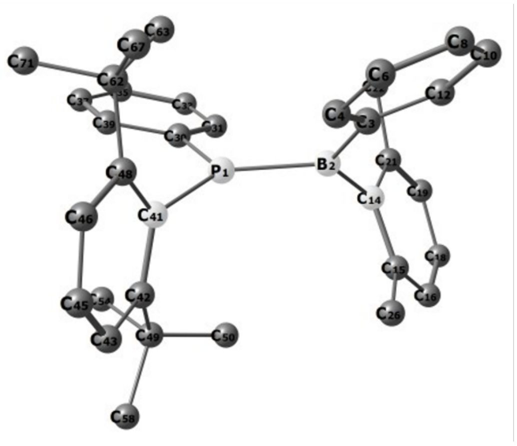

Figure S99. a) Overview of calculated energies for pyramidalized arrangement (left) at which only 20, 40, 60 and 80 % of the total geometry changes had taken place and twisted (right) geometric arrangements at different twist angles (180°, 170°, 160°, 140°, 120° and 100°) for **1^{Xyl*}.** b) Relevant atoms that contribute to the pyramidalization

marked in white. We put a dummy atom X vertical to the C_{41}-B_2-C_{30} plane above the P atom in each geometry. We then performed S_1 geometry optimizations starting from these geometries while freezing the X-P_1-C_{41}, X-P_1-B_2, X-P_1-C_{30} angles. c) **1^{Xyl*}** was twisted stepwise around the white marked C_{41}-P_1-B_2-C_{14} dihedral angle while starting from the S_0 geometry towards the TICT-S1 geometry.

A detailed mechanic explanation including dynamical simulations is out of the scope of the paper. However, already quantum chemical computations can give some insights, which are summarized in Table S6, Figure S98. Details for the calculation are given in the paper. For a vertical excitation the S_1 state lies about 0.95 eV above the T_1 and about 0.21 (0.27) eV below the T_2 and S_2 which are nearly degenerated at this geometry. Figure S98 contains the S_2 as well as T_2-T_3 states, however, they do not play a role for the photo physics since the absorption intensity of the S_2-state vanishes, i.e. it will not be populated by light absorption. In addition, populations would be directly quenched in the S_1, as this is only 0.27 eV away (Kasha's rule). Consequently, the absorption spectra as well as the emission are determined by the shape of the potential energy surface of the S_1-state and its interaction with the T_1 state. Starting from the S_0 geometry a geometry optimization for the S_1 state leads barrierless to the S_1 pyramidal structure which represents a local minimum on the S_1hypersurface. The energy difference to the vertical excitation is about 13 kcal/mol. T_1 also has a local minimum with a pyramidal structure. The geometry is similar to the corresponding S_1 minimum, but they are not identical. To get insights whether a crossing to a triplet state occurs along this relaxation, we first calculated the change in atomic coordinates between the S0 equilibrium geometry and the pyramidalized S_1 geometry (total geometry change). We then calculated the energies for those geometric arrangements at which only 20, 40, 60 and 80 % of the total geometry changes had taken place. Although these calculations only give an approximate insight, as the geometry changes are linearized, our experience shows that very reliable insights are obtained. The corresponding results are depicted as 0.2, 0.4, 0.6, and 0.8 in the graph given above. The graph shows that the $S_1$is separated from all other states during the relaxation to the local minimum S_1-pyramidal and no barrier which may hamper the relaxation occurs. Consequently, parts of the population of the S1 will reach this local minimum leading to emission.

We also investigated the torsional motion around the C_{41}-P_1-B_2-C_{14} dihedral angle which was the main relaxation path for the nitrogen analogion. In these calculations, the dihedral angle was frozen, while the remaining geometry parameters were optimized for the S_1. For the associated T_1 potential, the remaining geometry parameters were optimized for the T_1. For the phosphorus containing compound the corresponding minimum ($S_1$90°) is lower in energy than the pyramidal structure (~ 15 kcal/mol). This relaxation pathway has a small barrier of about 3 kcal/mol at 120°. However, it is so low that relaxation will also take place via this channel. Since the energy profile initially decreases starting from the S0 geometry, this relaxation should also be very efficient. As already found for the nitrogen counterpart this emission from the twisted structure is forbidden. In addition, the S_0 state is only about 20 kcal/mol more stable so that non-radiative decay is expected from this geometry. Since $S_1$and T_1 are nearly degenerate for the twisted S_1 minimum, a transition to the triplet can take place.

Table S6. Adiabatic S_0, S_1, S_2, T_1 (TDA), T_2 (TDA) and T_3 (TDA) energies (in kcal/mol and eV) in the respective geometries of 1Xyl*. Beside the energies for the respective ground and excited state minima, the energies for the geometries that present the stepwise twisting of the B=P bond and the pyramidalization are presented.

Geometry/State	*S_0*		*S_1*		*S_2*		*T_1*		*T_2*		*T_3*	
	kcal/mol	*eV*	*kcal/mol*	*eV*	*kcal/mol*	*eV*	*kcal/mol*	*eV*	*kcal/mol*	*eV*	*kcal/mol*	*eV*
pyr-T1	12.8	0.55	69.9	3.03	93.0	4.03	52.1	2.26	89.8	3.89	92.3	4.00
pyr-S_1	17.1	0.74	68.5	2.97	97.7	4.24	56.1	2.43	92.5	4.01	94.5	4.10
0.8[a]	23.0	1.00	75.1	3.26	101.4	4.40	59.8	2.59	97.1	4.21	100.5	4.36
0.6[a]	22.3	0.97	75.9	3.29	99.5	4.32	59.0	2.56	95.5	4.14	100.2	4.34
0.4[a]	22.3	0.97	77.0	3.34	98.5	4.27	59.1	2.56	94.7	4.11	100.8	4.37
0.2[a]	22.3	0.97	78.2	3.39	98.1	4.25	59.6	2.58	94.5	4.10	101.7	4.41
0.0[a]	22.4	0.97	79.6	3.45	96.1	4.17	61.1	2.65	92.9	4.03	102.1	4.43
S_0	0.0	0.00	81.8	3.55	88.1	3.82	60.0	2.60	86.6	3.76	89.6	3.89
180° (S_0)[b]	20.5	0.89	80.5	3.49	94.9	4.11	61.3	2.66	93.0	4.03	100.6	4.36
170°[b]	21.9	0.95	79.8	3.46	95.7	4.15	61.1	2.65	92.9	4.03	101.9	4.42
160°[b]	23.5	1.02	79.8	3.46	98.6	4.27	61.0	2.64	95.0	4.12	103.7	4.50
140°[b]	32.0	1.39	83.2	3.61	106.9	4.63	64.2	2.79	102.5	4.44	111.3	4.83
120°[b]	42.2	1.83	84.3	3.66	112.7	4.89	68.8	2.98	108.4	4.70	116.2	5.04
100°[b]	47.4	2.05	70.9	3.07	114.7	4.97	63.3	2.75	111.7	4.84	119.9	5.20
TICT-S_1	37.5	1.62	53.2	2.31	103.1	4.47	51.0	2.21	101.0	4.38	107.1	4.65
TICT-T_1	28.4	1.23	59.1	2.56	98.4	4.27	47.2	2.05	97.0	4.21	100.4	4.35

[a]As seen in Figure S94 1^{Xyl*} was pyramidalized stepwise. For that, we subtracted stepwise the geometry difference of the S_0 and pyr-S_1 geometry from the S_0 geometry to see what happens on the way to the pyr-S1 equilibrium ($R_x=R_0-x(R_0-R_{pyr\text{-}S1})$ with $0 < x < 1$). We put a dummy atom X vertical to the C_{41}-B_2-C_{30} plane above the P atom in each geometry. We then performed S_1 geometry optimizations starting from these geometries while freezing the X-P_1-C_{41}, X-P_1-B_2, X-P_1-C_{30} angles.

[b]The molecule was twisted stepwise around the C_{41}-P_1-B_2-C_{14} dihedral angle while starting from the S_0 geometry towards the TICT-S1 geometry. For each step we froze the boron and phosphorous containing dihedrals C_{41}-P_1-B_2-C_{14}, C_{41}-P_1-B_2-C_3, C_{30}-P_1-B_2-C_{14} and C_{30}-P_1-B_2-C_3 and performed a constrained S_1 optimization.

Table S7. Oscillator strengths of the relevant states for $\mathbf{1}^{Xyl*}$.

Geometry	*S_1*	*S_2*
pyr-T1	0.279	0.034
pyr-S_1	0.143	0.057
S_0	0.609	0.000
TICT-S_1	0.000	0.004
TICT-T_1	0.056	0.006

Table S8. Interior angles around the phosphorous atom and their sum for **1^{Xyl*}** in its S_0, pyr-S_1 and pyr-T_1 geometries.

Geometry	C_{41}-P_1-C_{30}	C_{41}-P_1-B_2	B_2-P_1-C_{30}	sum
S_0	109°	127°	124°	360°
pyr-S_1	105°	122°	119°	346°
pyr-T_1	106°	126°	122°	354°

Table S9. Calculated HOMO and LUMO energies (ω_TB97X-D3/def2-SVP, CPCM (THF) for the oligomers **1^{Xyl*}**, **2$^{a'}$ (Xyl, Xyl*)**, **2$^{b'}$ (Xyl, Xyl*)** in the respective geometries.

**Compound**	**Geometry**	HOMO energy (eV)	LUMO energy (eV)	HOMO-LUMO gap (eV)
**1^{Xyl}**	**S_0**	-7.04	-0.25	**6.78**
**1^{Dip}**	**S_0**	-6.89	-0.25	**6.64**
**1^{Xyl*}**	**S_0**	-6.53	-0.12	**6.41**
**1^{Xyl*}**	**pyr-S_1**	-6.00	-0.80	**5.19**
**1^{Xyl*}**	**pyr-T_1**	-6.01	-0.65	**5.36**
**1^{Xyl*}**	**TICT-S_1^{x}**	-5.46	-1.30	**4.16**
**1^{Xyl*}**	**TICT-T_1**	-5.62	-1.05	**4.57**
**2$^{a'}$ (Xyl, Xyl*)**	**S_0**	-6.17	-0.70	**5.47**
**2$^{a'}$ (Xyl, Xyl*)**	**pyr-S_1**	-5.80	-1.23	**4.57**
**2$^{a'}$ (Xyl, Xyl*)**	**pyr-T_1**	-5.75	-1.15	**4.61**
**2$^{a'}$ (Xyl, Xyl*)**	**TICT-S_1^{x}**	-5.19	-1.53	**3.66**
**2$^{a'}$ (Xyl, Xyl*)**	**TICT-T_1**	-5.47	-1.35	**4.12**
**2$^{b'}$ (Xyl, Xyl*)**	**S_0**	-6.00	-0.49	**5.51**
**2$^{b'}$ (Xyl, Xyl*)**	**pyr-S_1^{x}**	-5.63	-0.92	**4.71**
**2$^{b'}$ (Xyl, Xyl*)**	**pyr-T_1**	-5.62	-0.83	**4.79**
**2$^{b'}$ (Xyl, Xyl*)**	**TICT-S_1^{x}**	-5.19	-1.44	**3.75**
**2$^{b'}$ (Xyl, Xyl*)**	**TICT-T_1**	-5.31	-1.23	**4.07**

^{x}Imaginary frequency.

Table S10. Selected bond lengths (Å), bond angles (°), and interplanar angles (°) of the theoretically computed molecules in the ground-state (S_0) geometry. Atom and bond labels are given according to atom assignments in the experimentally determined structures.

Compound	**1^{Xyl}**	**1^{Dip}**	**1^{Xyl*}**	**$2^{a\prime}$ (Xyl, Xyl*)**	**$2^{b\prime}$ (Xyl, Xyl*)**
B1–P1	1.8819	1.8784	1.8134	1.8123	1.8130
B2–P2				1.8123	1.8130
Σ(RB1R)	359.1	359.3	360.0	360.0	360.0
Σ(RB2R)				360.0	360.0
Σ(RP1R)	332.0	332.0	360.0	360.0	360.0
Σ(RP2R)				360.0	360.0
∠Ph-Ph[°]	63.2	64.5	0.0	1.2	14.4
∠Ph-phenylene				0.6, 0.6	7.2, 7.2
∠ phenylene-phenylene	40.0	34.5	0.0	0.2, 0.3	
∠Ph_P-C_2BPC_2	24.1	31.4	0.0		1.9, 1.9
∠Ph_B-C_2BPC_2				0.3, 0.4	5.3, 5.3
∠ phenylene-C_2BPC_2					
∠BR_3-Xyl	86.4	84.9	90.0	90.0, 90.1	87.9, 87.9
∠PR_3-Xyl/Dip/Xyl*	77.7	30.4	90.0	90.1, 89.9	88.2, 88.8

Table S11. Mayer and Wiberg bond orders computed with ωB97X-D/6-31G(d)

	MBO	WBO
B-P bond (XylBPXyl*)	1.24	1.44
B-P bond (XylBPDip)	1.11	1.24
B-P bond (XylBPXyl)	1.12	1.25

Table S12. Calculated phosphorescence for pyr-T_1 and TICT-T_1

Geometry	Calculated phosphorescence (TDA) / nm (eV)
pyr-T_1	726 (1.81)
TICT-T_1	1518 (0.82)

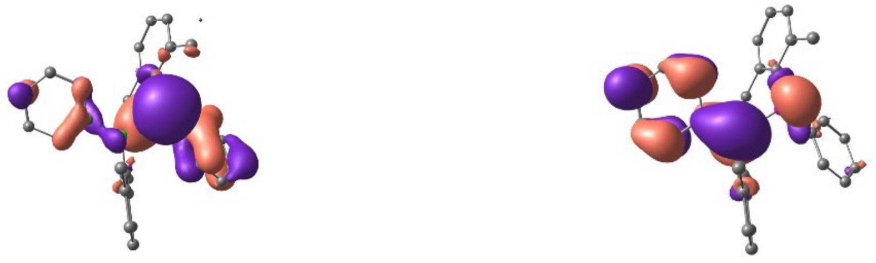

HOMO **LUMO**

Figure S100. Calculated frontier orbitals (isovalue 0.03 a.u.) of **1^{Xyl}** (ω_TB97X-D3/def2-SVP, CPCM (THF), ω_T = 0.135) for the S_1 state in the S_0 geometry.

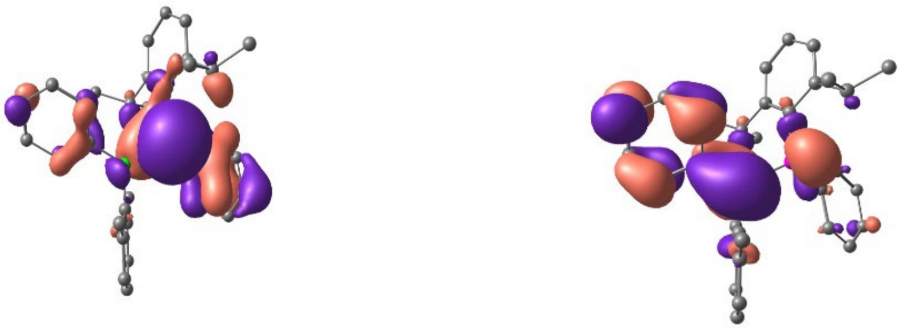

HOMO **LUMO**

Figure S101. Calculated frontier orbitals (isovalue 0.03 a.u.) of **1^{Dip}** (ω_TB97X-D3/def2-SVP, CPCM (THF), ω_T = 0.130) for the S_1 state in the S_0 geometry.

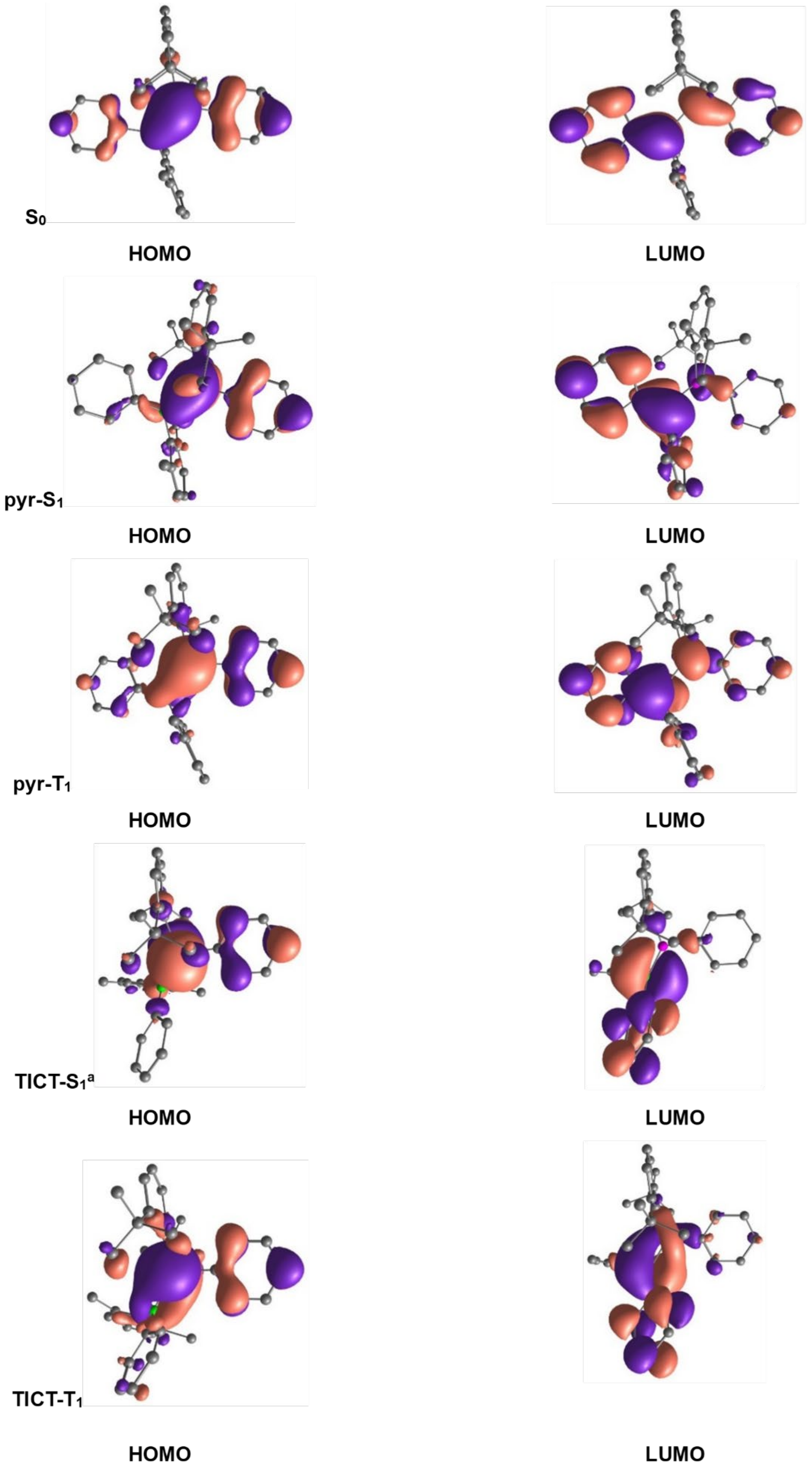

Figure S102. Calculated frontier orbitals (isovalue 0.03 a.u.) of **1^{Xyl*}** (ω_TB97X-D3/def2-SVP, CPCM (THF), ω_T = 0.125) for the S_1 state in different geometries.

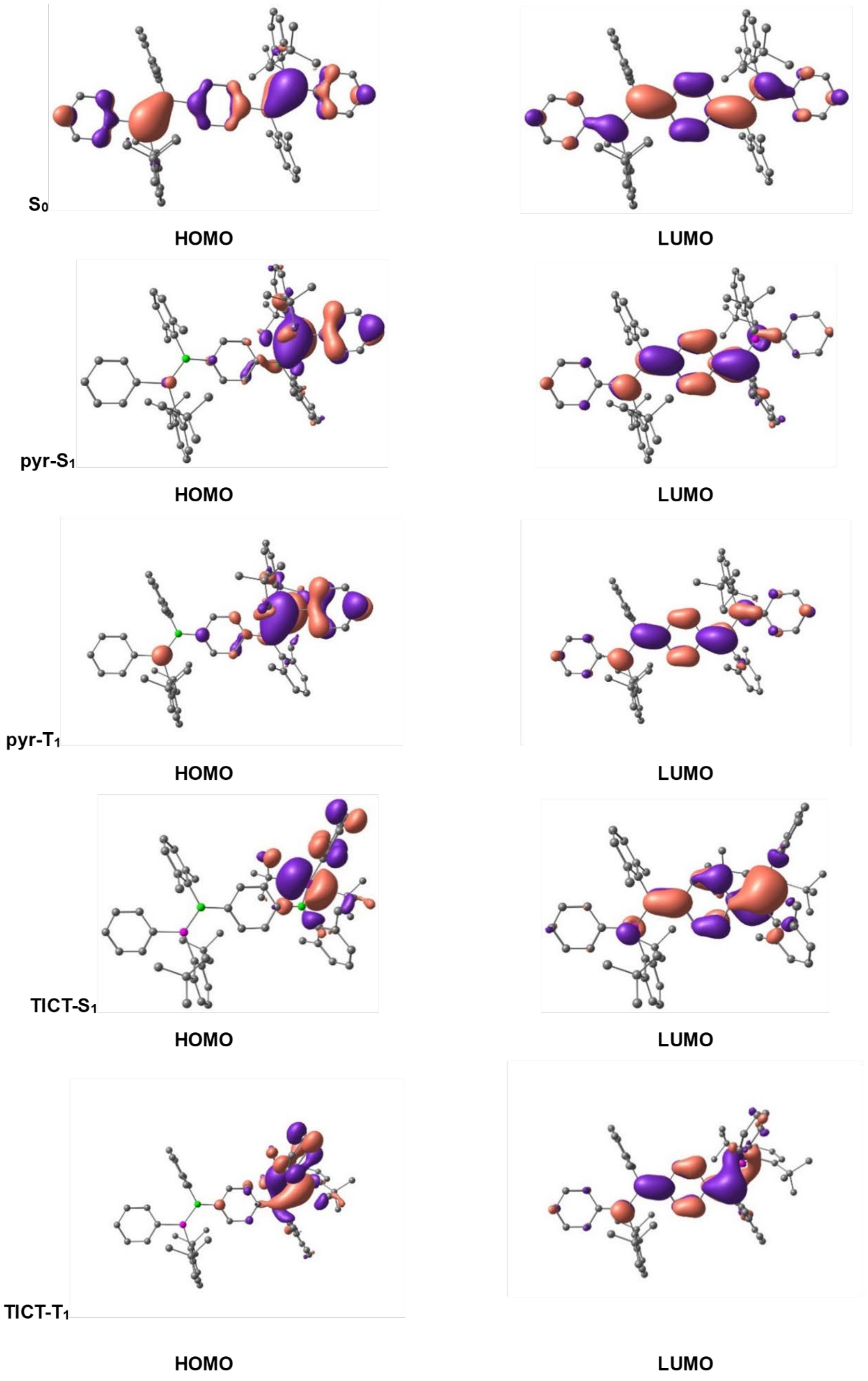

Figure S103. Calculated frontier orbitals (isovalue 0.03 a.u.) of **$2^{a\prime}$ (Xyl, Xyl*)** (ω_TB97X-D3/def2-SVP, CPCM (THF), ω_T = 0.105) for the S_1 state in different geometries.

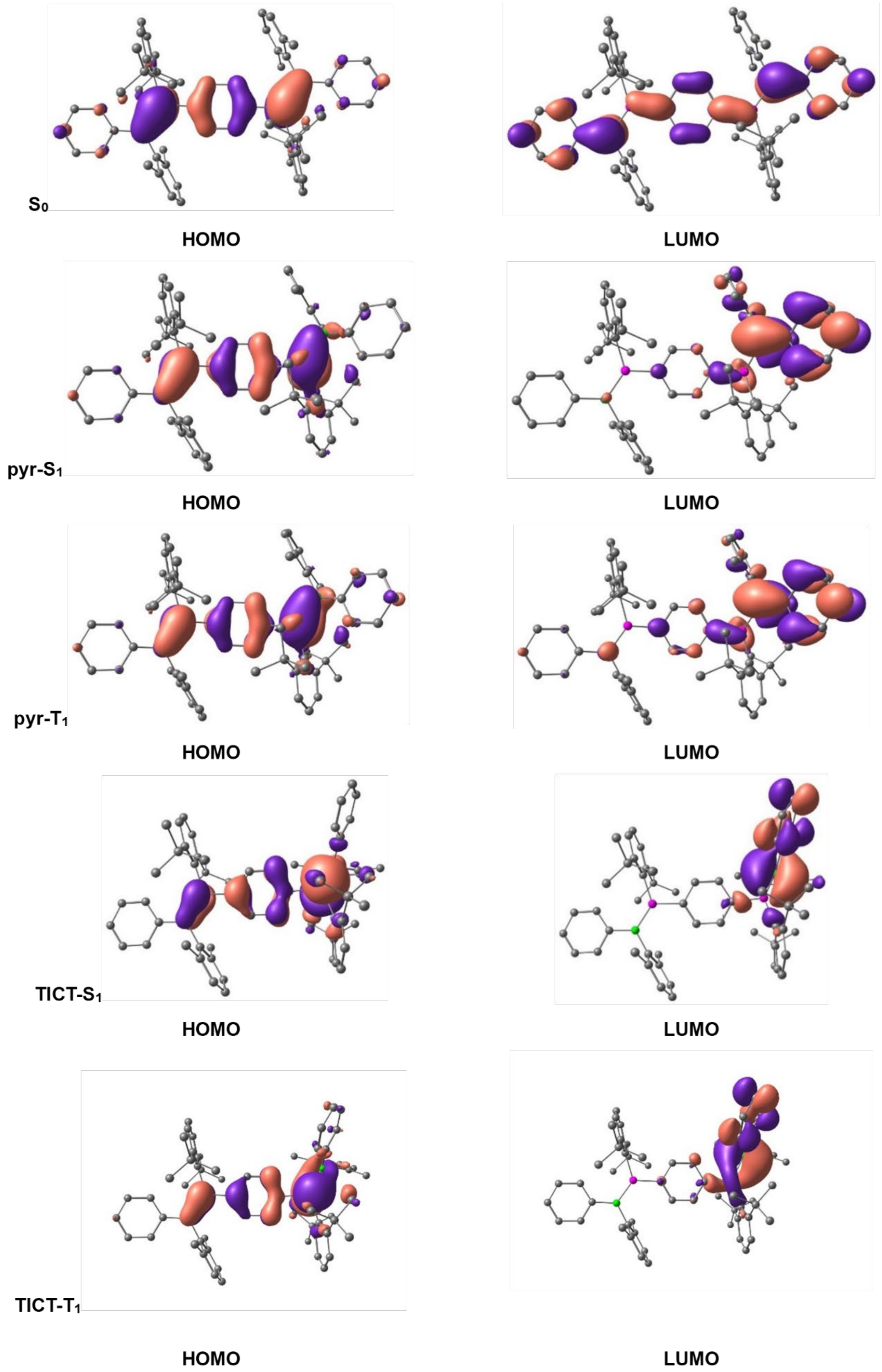

Figure S104. Calculated frontier orbitals (isovalue 0.03 a.u.) of **$2^{b´}$ (Xyl, Xyl*)** (ω_TB97X-D3/def2-SVP, CPCM (THF), ω_T = 0.105) for the S_1 state in different geometries.

Cartesian coordinates (Å) and total energies (hartree) of optimized stationary points

Compound **1^{Xyl} (S_0 geometry):**

Total energy (ωB97X-D/6-31G(d)): -1449.71465672

P	0.440878000	-0.615740000	0.803117000
B	-0.497743000	0.887899000	0.171109000
C	0.185598000	2.275559000	-0.063267000
C	-0.541127000	3.279201000	-0.728637000
H	-1.566795000	3.077665000	-1.027277000
C	0.022247000	4.519073000	-1.011153000
H	-0.558151000	5.273108000	-1.534927000
C	1.326437000	4.794313000	-0.610344000
H	1.768165000	5.764153000	-0.821602000
C	2.063150000	3.824797000	0.067637000
H	3.078663000	4.038027000	0.388372000
C	1.501295000	2.581804000	0.329596000
H	2.096187000	1.838247000	0.851512000
C	-2.073181000	0.768982000	0.134279000
C	-2.751015000	0.292380000	-1.002422000
C	-4.141330000	0.177133000	-0.982081000
H	-4.658242000	-0.190961000	-1.864994000
C	-4.865459000	0.519817000	0.152184000
C	-4.201027000	1.002145000	1.273547000
H	-4.766236000	1.282788000	2.159011000
C	-2.812728000	1.139724000	1.276141000
C	-2.114269000	1.674417000	2.504824000
H	-1.510200000	2.556743000	2.263812000
H	-1.435084000	0.929198000	2.935556000
H	-2.834754000	1.959398000	3.277170000
C	-1.987878000	-0.122336000	-2.236226000
H	-2.654214000	-0.232867000	-3.097027000
H	-1.490885000	-1.086783000	-2.074719000
H	-1.217256000	0.609774000	-2.502594000
C	-0.362335000	-2.156289000	0.232542000
C	-1.686980000	-2.418061000	0.608664000
H	-2.224723000	-1.712917000	1.234728000
C	-2.329701000	-3.569877000	0.170477000
H	-3.362091000	-3.746127000	0.457680000
C	-1.654972000	-4.495111000	-0.622356000
H	-2.157556000	-5.397204000	-0.958386000
C	-0.328899000	-4.260217000	-0.970763000
H	0.211554000	-4.979776000	-1.579143000
C	0.313849000	-3.099195000	-0.549160000
H	1.348487000	-2.927474000	-0.831553000
C	2.198527000	-0.705952000	0.272616000
C	2.617101000	-0.480653000	-1.056850000
C	3.979911000	-0.531543000	-1.355948000
H	4.303660000	-0.356032000	-2.378366000
C	4.920469000	-0.802740000	-0.372388000
C	4.506199000	-1.041783000	0.930157000
H	5.241018000	-1.266727000	1.698412000
C	3.153075000	-1.001814000	1.270519000
H	5.976838000	-0.835671000	-0.623193000
H	-5.947162000	0.419936000	0.160229000
C	1.650190000	-0.182644000	-2.173489000
H	2.144468000	-0.271679000	-3.145139000
H	0.799092000	-0.868426000	-2.162607000
H	1.257305000	0.836424000	-2.090994000
C	2.758146000	-1.285963000	2.701316000
H	2.240652000	-0.434787000	3.154889000
H	2.078777000	-2.142270000	2.767052000
H	3.642279000	-1.510169000	3.305306000

Compound **1^{Dip} (S_0 geometry):**

Total energy (ωB97X-D/6-31G(d)): -1606.92990032

P	-0.287102000	-0.584487000	-0.704047000
B	0.994690000	0.749440000	-0.378157000
C	0.589761000	2.258258000	-0.314962000
C	1.482325000	3.164759000	0.281464000
H	2.450938000	2.806410000	0.621406000
C	1.150433000	4.505058000	0.451236000
H	1.851360000	5.182117000	0.930799000
C	-0.075654000	4.978209000	-0.006933000
H	-0.335582000	6.025911000	0.115261000
C	-0.967239000	4.105924000	-0.629671000
H	-1.921436000	4.472532000	-0.996535000
C	-0.641020000	2.762975000	-0.770015000
H	-1.359191000	2.096446000	-1.238579000
C	2.518070000	0.335714000	-0.404229000
C	3.190419000	-0.131687000	0.741173000
C	4.529479000	-0.512538000	0.647973000
H	5.043319000	-0.872716000	1.535808000
C	5.206267000	-0.445306000	-0.562878000
C	4.549225000	0.024517000	-1.693416000
H	5.078414000	0.087182000	-2.641149000
C	3.214486000	0.424981000	-1.627236000
C	2.523186000	0.937832000	-2.869465000
H	2.134942000	1.951273000	-2.715766000
H	1.669976000	0.306050000	-3.143897000
H	3.208928000	0.964611000	-3.721397000
C	2.480741000	-0.239677000	2.069033000
H	3.169846000	-0.545992000	2.861529000
H	1.674707000	-0.982143000	2.025664000
H	2.036756000	0.717729000	2.368274000
C	0.240492000	-2.196417000	-0.024885000
C	1.472253000	-2.745783000	-0.406702000
H	2.119030000	-2.210055000	-1.094045000
C	1.884298000	-3.972582000	0.100527000

H	2.850205000	-4.372707000	-0.193416000
C	1.064804000	-4.687967000	0.970181000
H	1.387654000	-5.648334000	1.360942000
C	-0.176389000	-4.168390000	1.323061000
H	-0.831693000	-4.722947000	1.988728000
C	-0.587166000	-2.932980000	0.829508000
H	-1.559097000	-2.539047000	1.111149000
C	-1.945875000	-0.233601000	0.021061000
C	-2.132126000	0.314056000	1.312934000
C	-3.432373000	0.578515000	1.749631000
H	-3.584284000	0.995688000	2.741172000
C	-4.530655000	0.318793000	0.946851000
C	-4.347298000	-0.235441000	-0.311283000
H	-5.214609000	-0.445999000	-0.928441000
C	-3.070083000	-0.531427000	-0.788270000
C	-0.999653000	0.610086000	2.282521000
H	-0.062888000	0.468355000	1.745390000
C	-1.016175000	2.058322000	2.788051000
H	-1.863640000	2.242542000	3.458707000
H	-1.072337000	2.771614000	1.961550000
H	-0.100703000	2.264266000	3.354119000
C	-0.992518000	-0.376252000	3.458255000
H	-0.146885000	-0.162830000	4.122165000
H	-0.896664000	-1.409544000	3.112156000
H	-1.912644000	-0.296308000	4.049199000
C	-2.931578000	-1.133937000	-2.183289000
H	-1.993842000	-1.697590000	-2.214574000
C	-4.049183000	-2.121409000	-2.538405000
H	-4.188363000	-2.868019000	-1.749957000
H	-3.793554000	-2.646824000	-3.464540000
H	-5.008833000	-1.620279000	-2.708190000
C	-2.832255000	-0.021749000	-3.236538000
H	-3.727944000	0.610353000	-3.215464000
H	-2.737712000	-0.448137000	-4.241512000
H	-1.958986000	0.614192000	-3.056540000
H	-5.532424000	0.539923000	1.304263000
H	6.246337000	-0.752753000	-0.624488000

Compound **1^{Xyl*} (S_0 geometry):**

Total energy (ωB97X-D/6-31G(d)): -1685.50590955

P	-0.171170000	-0.416497000	-0.000209000
B	1.241871000	0.720108000	0.000008000
C	1.093350000	2.285928000	0.000211000
C	-0.132080000	2.976589000	0.000451000
H	-1.064462000	2.420739000	0.000514000
C	-0.186437000	4.364193000	0.000593000
H	-1.149646000	4.866668000	0.000773000
C	0.990981000	5.109681000	0.000500000
H	0.949957000	6.195351000	0.000614000
C	2.218305000	4.455033000	0.000253000
H	3.141922000	5.026919000	0.000175000
C	2.263884000	3.064280000	0.000106000
H	3.229697000	2.565897000	-0.000088000
C	2.685935000	0.073005000	-0.000190000
C	3.338158000	-0.205804000	1.216073000
C	4.610280000	-0.778769000	1.202025000
H	5.108898000	-0.996637000	2.143421000
C	5.242602000	-1.071869000	-0.000461000
C	4.609997000	-0.778815000	-1.202804000
H	5.108396000	-0.996722000	-2.144306000
C	3.337873000	-0.205842000	-1.216586000
C	2.659328000	0.093110000	-2.531785000
H	3.341378000	-0.056282000	-3.374216000
H	2.298661000	1.127715000	-2.567264000
H	1.788423000	-0.555341000	-2.685362000
C	2.659876000	0.093138000	2.531409000
H	3.342035000	-0.056427000	3.373720000
H	1.788901000	-0.555207000	2.685066000
H	2.299361000	1.127791000	2.567039000
C	-0.017714000	-2.222449000	-0.000459000
C	1.224756000	-2.870787000	0.000136000
H	2.144262000	-2.299393000	0.000746000
C	1.291790000	-4.260240000	-0.000039000
H	2.265837000	-4.740636000	0.000422000
C	0.132345000	-5.028845000	-0.000766000
H	0.191801000	-6.113014000	-0.000884000
C	-1.104875000	-4.391919000	-0.001328000
H	-2.020709000	-4.975962000	-0.001886000
C	-1.181375000	-3.004377000	-0.001184000
H	-2.155538000	-2.525398000	-0.001626000
C	-1.948241000	0.031452000	0.000175000
C	-2.639710000	0.169044000	1.247633000
C	-4.015696000	0.413349000	1.196790000
H	-4.586981000	0.517444000	2.107026000
C	-4.696057000	0.526317000	0.000726000
C	-4.016062000	0.413988000	-1.195607000
H	-4.587627000	0.518550000	-2.105615000
C	-2.640092000	0.169721000	-1.246998000
C	-2.002516000	0.105447000	2.672400000
C	-1.016630000	1.271424000	2.877923000
H	-0.159128000	1.215305000	2.210779000
H	-0.639883000	1.256744000	3.907459000
H	-1.513048000	2.233742000	2.711417000
C	-1.315713000	-1.245033000	2.961081000
H	-0.404850000	-1.396414000	2.384912000
H	-1.990273000	-2.082076000	2.751302000
H	-1.041490000	-1.289894000	4.021197000
C	-3.065020000	0.261376000	3.782896000
H	-3.578895000	1.227351000	3.741547000
H	-2.559240000	0.210671000	4.752080000

H	-3.811584000	-0.540186000	3.763951000
C	-2.003353000	0.106919000	-2.671996000
C	-1.017399000	1.272909000	-2.877148000
H	-0.641216000	1.258966000	-3.906900000
H	-0.159541000	1.216159000	-2.210524000
H	-1.513592000	2.235167000	-2.709630000
C	-3.066198000	0.263651000	-3.782048000
H	-2.560747000	0.213391000	-4.751428000
H	-3.579889000	1.229692000	-3.739983000
H	-3.812901000	-0.537788000	-3.763300000
C	-1.316836000	-1.243478000	-2.961706000
H	-1.042506000	-1.287537000	-4.021828000
H	-1.991639000	-2.080520000	-2.752700000
H	-0.406082000	-1.395558000	-2.385547000
H	6.232203000	-1.520061000	-0.000569000
H	-5.766817000	0.709738000	0.000938000

Compound **1**^{Xyl*} **(pyr-S$_1$ geometry):**

Total energy (ωB97X-D/6-31G(d)): -1685.47876328

P	-0.170102000	-0.460720000	0.503602000
B	1.356853000	0.847388000	0.137268000
C	1.053694000	2.236283000	-0.420867000
C	-0.223411000	2.752375000	-0.761810000
H	-1.097147000	2.114227000	-0.694147000
C	-0.401789000	4.065340000	-1.174887000
H	-1.399988000	4.411599000	-1.431534000
C	0.680173000	4.939475000	-1.253452000
H	0.538651000	5.967269000	-1.573709000
C	1.949543000	4.474596000	-0.897795000
H	2.803544000	5.145892000	-0.937528000
C	2.131838000	3.164290000	-0.492019000
H	3.125933000	2.835193000	-0.199906000
C	2.761206000	0.205660000	0.391023000
C	3.137984000	-0.264808000	1.676662000
C	4.378260000	-0.871256000	1.873883000
H	4.646975000	-1.223399000	2.866888000
C	5.272717000	-1.015221000	0.823224000
C	4.924454000	-0.552917000	-0.442158000
H	5.620577000	-0.676125000	-1.268381000
C	3.689080000	0.047019000	-0.677114000
C	3.341809000	0.443393000	-2.092423000
H	4.149719000	0.174750000	-2.779874000
H	3.155859000	1.517088000	-2.189518000
H	2.430786000	-0.065168000	-2.427616000
C	2.215454000	-0.091573000	2.849511000
H	2.665542000	-0.461123000	3.775625000
H	1.270377000	-0.636861000	2.695742000
H	1.946576000	0.961291000	2.988910000
C	-0.006542000	-2.170409000	-0.032678000
C	1.258039000	-2.706705000	-0.345851000
H	2.129009000	-2.062029000	-0.379912000
C	1.395176000	-4.059445000	-0.630983000
H	2.375159000	-4.451975000	-0.885055000
C	0.291019000	-4.906299000	-0.591355000
H	0.404293000	-5.963252000	-0.812334000
C	-0.962311000	-4.390837000	-0.257772000
H	-1.827390000	-5.046214000	-0.217760000
C	-1.113546000	-3.041387000	0.022530000
H	-2.093835000	-2.653380000	0.281810000
C	-1.922330000	-0.032721000	0.185966000
C	-2.727173000	0.325687000	1.317040000
C	-4.109657000	0.377098000	1.149796000
H	-4.750557000	0.627587000	1.983414000
C	-4.704750000	0.095478000	-0.068316000
C	-3.916819000	-0.170204000	-1.167679000
H	-4.408621000	-0.337287000	-2.118735000
C	-2.517590000	-0.209591000	-1.099414000
C	-2.169290000	0.639673000	2.730463000
C	-1.038991000	1.683664000	2.662530000
H	-0.161547000	1.366670000	2.093808000
H	-0.690675000	1.916941000	3.675079000
H	-1.398439000	2.608797000	2.199322000
C	-1.717954000	-0.659068000	3.429163000
H	-0.943508000	-1.190191000	2.869343000
H	-2.568974000	-1.338461000	3.551986000
H	-1.314663000	-0.430790000	4.422680000
C	-3.241301000	1.266194000	3.647238000
H	-3.672741000	2.174394000	3.212985000
H	-2.769380000	1.544341000	4.595035000
H	-4.051745000	0.570660000	3.888260000
C	-1.848480000	-0.359196000	-2.501718000
C	-0.315800000	-0.343671000	-2.594550000
H	-0.041107000	-0.225410000	-3.649002000
H	0.138350000	-1.273121000	-2.253745000
H	0.135590000	0.497532000	-2.063809000
C	-2.317149000	0.845737000	-3.356283000
H	-1.929734000	0.742401000	-4.375887000
H	-1.926973000	1.782150000	-2.943349000
H	-3.404511000	0.930118000	-3.423311000
C	-2.312215000	-1.677480000	-3.157211000
H	-1.854467000	-1.773001000	-4.148274000
H	-3.396692000	-1.730705000	-3.290349000
H	-1.998721000	-2.537425000	-2.556291000
H	6.238188000	-1.485932000	0.985976000
H	-5.786518000	0.111762000	-0.163355000

Compound **1**^{Xyl*} **(pyr-T$_1$ geometry):**

Total energy (ωB97X-D/6-31G(d)): -1685.41952439

P	-0.170394000	-0.444355000	-0.285438000
B	1.369777000	0.802532000	-0.173886000

C	1.117959000	2.270560000	0.156041000
C	-0.147252000	2.889396000	0.351826000
H	-1.054603000	2.297930000	0.276539000
C	-0.275872000	4.243084000	0.625360000
H	-1.267888000	4.665390000	0.768969000
C	0.849095000	5.064171000	0.707839000
H	0.747141000	6.124240000	0.920151000
C	2.109124000	4.496953000	0.498270000
H	2.998215000	5.121137000	0.545093000
C	2.240492000	3.145190000	0.228514000
H	3.233027000	2.735040000	0.057078000
C	2.770756000	0.086484000	-0.335425000
C	3.628025000	-0.056644000	0.784443000
C	4.861691000	-0.694898000	0.646926000
H	5.507696000	-0.798875000	1.515706000
C	5.268032000	-1.208808000	-0.578663000
C	4.439403000	-1.073774000	-1.684267000
H	4.756613000	-1.464119000	-2.648539000
C	3.206152000	-0.429332000	-1.577269000
C	2.352208000	-0.276741000	-2.810580000
H	2.883385000	-0.611097000	-3.707374000
H	2.055295000	0.767661000	-2.952991000
H	1.427496000	-0.864265000	-2.741515000
C	3.206753000	0.421215000	2.154060000
H	3.997793000	0.247596000	2.890554000
H	2.309541000	-0.110199000	2.493629000
H	2.961822000	1.487636000	2.158589000
C	-0.066090000	-2.139121000	0.282065000
C	1.179894000	-2.694976000	0.630009000
H	2.074612000	-2.083132000	0.608910000
C	1.266699000	-4.028280000	1.008466000
H	2.232511000	-4.440366000	1.283724000
C	0.128806000	-4.831090000	1.034448000
H	0.203274000	-5.873019000	1.330443000
C	-1.107180000	-4.294239000	0.673225000
H	-1.996662000	-4.916801000	0.686421000
C	-1.208552000	-2.963602000	0.295882000
H	-2.174668000	-2.555473000	0.016341000
C	-1.940945000	0.003941000	-0.195482000
C	-2.597745000	0.091758000	1.063901000
C	-3.995270000	0.204838000	1.047323000
H	-4.537181000	0.245917000	1.984612000
C	-4.717080000	0.277075000	-0.123928000
C	-4.055925000	0.268841000	-1.341196000
H	-4.646930000	0.361042000	-2.241436000
C	-2.671593000	0.136183000	-1.419691000
C	-1.998990000	0.126336000	2.502805000
C	-0.472529000	0.096397000	2.663400000
H	-0.036284000	-0.871266000	2.411054000
H	-0.241988000	0.269738000	3.720783000
H	0.031848000	0.888355000	2.105355000
C	-2.549410000	-1.075655000	3.299591000
H	-2.234462000	-2.018435000	2.838704000
H	-3.641147000	-1.078782000	3.367393000
H	-2.154817000	-1.050905000	4.321483000
C	-2.445411000	1.455268000	3.159671000
H	-2.009273000	2.306931000	2.626400000
H	-2.088122000	1.488395000	4.194708000
H	-3.530163000	1.584958000	3.182350000
C	-2.024559000	0.142656000	-2.828097000
C	-0.951177000	1.243985000	-2.920305000
H	-0.508576000	1.248042000	-3.922783000
H	-0.128541000	1.134410000	-2.209535000
H	-1.399226000	2.226302000	-2.737108000
C	-3.046349000	0.469505000	-3.935934000
H	-2.517918000	0.512801000	-4.893628000
H	-3.524385000	1.442373000	-3.781076000
H	-3.823794000	-0.295865000	-4.031821000
C	-1.462271000	-1.250093000	-3.177714000
H	-1.007306000	-1.229849000	-4.174406000
H	-2.266169000	-1.994746000	-3.181681000
H	-0.697546000	-1.597460000	-2.478634000
H	6.228522000	-1.708174000	-0.671514000
H	-5.799436000	0.360118000	-0.093039000

Compound **1$^{Xyl^{*}}$ (TICT-S$_1$ geometry):**

Total energy (ωB97X-D/6-31G(d)): -1685.44481591

P	0.353708000	0.208225000	0.292002000
B	-1.525639000	0.294152000	0.011545000
C	-2.230759000	1.674890000	-0.118591000
C	-1.637227000	2.920875000	0.178064000
H	-0.600303000	2.956895000	0.493677000
C	-2.337674000	4.117806000	0.098011000
H	-1.832828000	5.051641000	0.332094000
C	-3.682284000	4.122764000	-0.262608000
H	-4.234925000	5.055845000	-0.321193000
C	-4.313177000	2.907559000	-0.526596000
H	-5.367439000	2.889289000	-0.790333000
C	-3.605610000	1.716349000	-0.451456000
H	-4.129058000	0.783408000	-0.642048000
C	-2.341076000	-1.070162000	-0.118417000
C	-2.753682000	-1.480217000	-1.408949000
C	-3.467214000	-2.668838000	-1.573663000
H	-3.768802000	-2.974487000	-2.572964000
C	-3.789267000	-3.464165000	-0.482372000
C	-3.418480000	-3.055270000	0.791558000
H	-3.690889000	-3.659864000	1.653609000
C	-2.712928000	-1.866394000	0.985149000
C	-2.379688000	-1.445335000	2.395733000
H	-2.932251000	-2.045276000	3.125793000

H	-2.635958000	-0.392814000	2.556106000
H	-1.313206000	-1.556851000	2.623073000
C	-2.452203000	-0.649066000	-2.636029000
H	-2.560640000	-1.242946000	-3.548959000
H	-1.438116000	-0.235703000	-2.613202000
H	-3.130169000	0.209078000	-2.708573000
C	1.024067000	0.170819000	1.975144000
C	0.221539000	0.711769000	2.989361000
H	-0.767182000	1.086194000	2.743014000
C	0.689628000	0.780878000	4.296746000
H	0.055367000	1.197596000	5.072922000
C	1.968559000	0.327746000	4.604353000
H	2.335845000	0.383935000	5.624587000
C	2.783434000	-0.186637000	3.596746000
H	3.786167000	-0.531335000	3.830850000
C	2.321064000	-0.260697000	2.289700000
H	2.966864000	-0.656180000	1.512769000
C	1.827056000	-0.144057000	-0.770076000
C	2.278497000	-1.494648000	-0.907140000
C	3.581439000	-1.687435000	-1.377245000
H	3.984357000	-2.686594000	-1.460116000
C	4.392386000	-0.632331000	-1.753674000
C	3.897164000	0.657110000	-1.731040000
H	4.533080000	1.456530000	-2.085732000
C	2.615245000	0.939510000	-1.254185000
C	1.407193000	-2.767089000	-0.706232000
C	0.336023000	-2.754815000	-1.811491000
H	-0.270092000	-1.851950000	-1.765420000
H	-0.340771000	-3.609106000	-1.700243000
H	0.804408000	-2.802060000	-2.800919000
C	0.734980000	-2.888262000	0.673165000
H	-0.061933000	-2.161365000	0.825290000
H	1.458936000	-2.793979000	1.488457000
H	0.254077000	-3.868878000	0.751632000
C	2.220004000	-4.065266000	-0.883257000
H	2.651824000	-4.159020000	-1.884421000
H	1.545069000	-4.916032000	-0.748424000
H	3.021000000	-4.159140000	-0.140985000
C	2.129282000	2.407008000	-1.321081000
C	0.779995000	2.466508000	-2.064207000
H	0.383736000	3.487317000	-2.056282000
H	0.006160000	1.824436000	-1.642088000
H	0.921963000	2.159847000	-3.106605000
C	3.082865000	3.301289000	-2.139967000
H	2.633654000	4.295781000	-2.229574000
H	3.238364000	2.917477000	-3.153690000
H	4.057945000	3.431321000	-1.658632000
C	2.075839000	3.040194000	0.084152000
H	1.712053000	4.071603000	0.014078000
H	3.079457000	3.059566000	0.523360000
H	1.430984000	2.510504000	0.785635000
H	-4.338470000	-4.391153000	-0.622480000
H	5.404348000	-0.822009000	-2.099437000

Compound **1^{Xyl*} (TICT-T_1 geometry):**

Total energy (ωB97X-D/6-31G(d)): -1685.42908121

P	-0.323222000	-0.296364000	0.294101000
B	1.552367000	-0.084888000	-0.028515000
C	2.420334000	-1.355544000	-0.184054000
C	1.981103000	-2.679471000	0.048479000
H	0.954387000	-2.853807000	0.360522000
C	2.820707000	-3.774688000	-0.095931000
H	2.437190000	-4.774491000	0.091807000
C	4.152099000	-3.597035000	-0.470845000
H	4.812184000	-4.452441000	-0.581834000
C	4.624964000	-2.303786000	-0.692893000
H	5.661574000	-2.147423000	-0.980536000
C	3.780648000	-1.211184000	-0.551352000
H	4.171754000	-0.212346000	-0.729415000
C	2.127411000	1.384347000	-0.187074000
C	2.272292000	1.941580000	-1.476018000
C	2.783683000	3.230723000	-1.629467000
H	2.885691000	3.651805000	-2.626995000
C	3.153931000	3.981942000	-0.520552000
C	3.043026000	3.432088000	0.750485000
H	3.357561000	4.008340000	1.617695000
C	2.550291000	2.137590000	0.926885000
C	2.510279000	1.542547000	2.315154000
H	2.983430000	2.207395000	3.044932000
H	3.036879000	0.581643000	2.336080000
H	1.486089000	1.351777000	2.657737000
C	1.848244000	1.157886000	-2.696148000
H	2.094560000	1.694569000	-3.617912000
H	0.765787000	0.974701000	-2.694026000
H	2.334562000	0.176795000	-2.727968000
C	-0.905296000	-0.813241000	1.911994000
C	0.061932000	-1.213490000	2.848041000
H	1.113392000	-1.177181000	2.575083000
C	-0.324820000	-1.658989000	4.105599000
H	0.430823000	-1.962750000	4.823420000
C	-1.675408000	-1.721849000	4.441005000
H	-1.976103000	-2.072507000	5.423647000
C	-2.642367000	-1.341183000	3.510606000
H	-3.695815000	-1.394657000	3.768545000
C	-2.265729000	-0.892592000	2.252177000
H	-3.022271000	-0.597552000	1.531603000
C	-1.825787000	0.135537000	-0.676088000
C	-2.467411000	1.396509000	-0.468837000
C	-3.736743000	1.567391000	-1.031055000
H	-4.274564000	2.492436000	-0.882618000

C	-4.342784000	0.586734000	-1.792114000
C	-3.676134000	-0.595240000	-2.052213000
H	-4.164467000	-1.325764000	-2.681396000
C	-2.413988000	-0.860015000	-1.516501000
C	-1.836148000	2.656873000	0.191442000
C	-0.863512000	3.256530000	-0.841614000
H	-0.075001000	2.549474000	-1.099732000
H	-0.377443000	4.150791000	-0.435118000
H	-1.393872000	3.532884000	-1.759573000
C	-1.081711000	2.414707000	1.509263000
H	-0.147847000	1.870611000	1.364864000
H	-1.692541000	1.884570000	2.245840000
H	-0.791166000	3.380985000	1.934845000
C	-2.894656000	3.732405000	0.516246000
H	-3.359064000	4.156227000	-0.378834000
H	-2.402202000	4.562171000	1.032097000
H	-3.682032000	3.349565000	1.175462000
C	-1.755850000	-2.216553000	-1.885240000
C	-0.359177000	-1.994069000	-2.496436000
H	0.103179000	-2.957939000	-2.735012000
H	0.336155000	-1.466245000	-1.843411000
H	-0.444742000	-1.414692000	-3.422666000
C	-2.554199000	-2.976453000	-2.965091000
H	-2.001603000	-3.884200000	-3.227639000
H	-2.673029000	-2.388474000	-3.881280000
H	-3.543149000	-3.292298000	-2.616403000
C	-1.715170000	-3.157676000	-0.664775000
H	-1.250001000	-4.109213000	-0.945510000
H	-2.731827000	-3.362406000	-0.310677000
H	-1.153731000	-2.757584000	0.179244000
H	3.542898000	4.988533000	-0.647817000
H	-5.333460000	0.754623000	-2.204475000

Compound **2ª(Xyl, Xyl*) (S₀ geometry):**

Total energy (ωB97X-D/6-31G(d)): -3138.84957625

P	-4.299726000	0.072778000	0.000002000
C	-1.350564000	0.477703000	-0.012101000
B	-2.798916000	1.088548000	-0.010727000
C	-0.253615000	1.357583000	-0.012728000
H	-0.433599000	2.429521000	-0.013270000
C	1.054217000	0.895449000	-0.012176000
H	1.859062000	1.623663000	-0.011696000
C	-5.979345000	0.751548000	0.003284000
C	-7.070694000	-0.128151000	0.016691000
H	-6.900728000	-1.200401000	0.025118000
C	-8.373065000	0.356742000	0.019311000
H	-9.202737000	-0.344243000	0.029767000
C	-8.613998000	1.727313000	0.008722000
H	-9.631763000	2.105642000	0.010825000
C	-7.536211000	2.606822000	-0.004690000
H	-7.706375000	3.679440000	-0.013184000
C	-6.229341000	2.130422000	-0.007574000
H	-5.410054000	2.838239000	-0.018396000
C	-2.982069000	2.660115000	-0.017153000
C	-3.045267000	3.371586000	1.196072000
C	-3.205349000	4.757504000	1.176393000
H	-3.258352000	5.302948000	2.115542000
C	-3.296469000	5.443558000	-0.028908000
C	-3.212096000	4.746604000	-1.228451000
H	-3.270407000	5.283497000	-2.172215000
C	-3.052012000	3.360576000	-1.236407000
C	-2.956841000	2.640379000	2.514009000
H	-2.898951000	3.339457000	3.353765000
H	-2.072715000	1.993077000	2.550439000
H	-3.831214000	1.997569000	2.671193000
C	-2.971467000	2.617207000	-2.548026000
H	-3.847121000	1.973516000	-2.694245000
H	-2.087955000	1.969016000	-2.583464000
H	-2.918001000	3.308433000	-3.394552000
C	-4.389365000	-1.757208000	0.009511000
C	-4.459460000	-2.465395000	-1.234122000
C	-4.614607000	-3.853863000	-1.175242000
H	-4.679471000	-4.436489000	-2.081859000
C	-4.692695000	-4.532211000	0.024692000
C	-4.602820000	-3.842113000	1.217050000
H	-4.658746000	-4.415530000	2.130125000
C	-4.447117000	-2.453154000	1.260631000
C	-4.343824000	-1.844283000	-2.662764000
C	-5.436277000	-0.794075000	-2.949756000
H	-5.309778000	0.125147000	-2.380911000
H	-6.432517000	-1.192423000	-2.729434000
H	-5.407359000	-0.525628000	-4.011897000
C	-4.512662000	-2.911650000	-3.766728000
H	-4.424251000	-2.417424000	-4.739172000
H	-5.495569000	-3.394557000	-3.735596000
H	-3.736047000	-3.682492000	-3.729290000
C	-2.941646000	-1.243625000	-2.880608000
H	-2.851793000	-0.886672000	-3.913432000
H	-2.165222000	-1.997938000	-2.712476000
H	-2.740876000	-0.402230000	-2.220887000
C	-4.317238000	-1.818030000	2.681814000
C	-4.475361000	-2.874348000	3.797897000
H	-3.699130000	-3.645587000	3.760543000
H	-5.458517000	-3.357466000	3.781205000
H	-4.377473000	-2.370484000	4.764472000
C	-5.406579000	-0.764719000	2.969098000
H	-5.365110000	-0.483384000	4.027483000
H	-6.405196000	-1.166070000	2.765550000
H	-5.287289000	0.147532000	2.387575000
C	-2.912819000	-1.215726000	2.879684000

H	-2.719036000	-0.380262000	2.210443000
H	-2.138216000	-1.971547000	2.709929000
H	-2.812059000	-0.849679000	3.908316000
P	4.299749000	-0.072779000	0.000508000
C	1.350586000	-0.477698000	-0.011944000
B	2.798938000	-1.088546000	-0.010326000
C	0.253638000	-1.357577000	-0.012033000
H	0.433623000	-2.429515000	-0.012027000
C	-1.054193000	-0.895443000	-0.011626000
H	-1.859038000	-1.623657000	-0.010708000
C	5.979368000	-0.751551000	0.003677000
C	7.070716000	0.128139000	0.017762000
H	6.900751000	1.200383000	0.026799000
C	8.373087000	-0.356757000	0.020263000
H	9.202758000	0.344221000	0.031247000
C	8.614020000	-1.727322000	0.008895000
H	9.631785000	-2.105652000	0.010913000
C	7.536235000	-2.606822000	-0.005206000
H	7.706399000	-3.679434000	-0.014337000
C	6.229365000	-2.130418000	-0.008010000
H	5.410081000	-2.838228000	-0.019441000
C	2.982081000	-2.660113000	-0.016483000
C	3.051814000	-3.360750000	-1.235653000
C	3.211874000	-4.746778000	-1.227531000
H	3.270026000	-5.283805000	-2.171230000
C	3.296425000	-5.443564000	-0.027900000
C	3.205508000	-4.757337000	1.177316000
H	3.258647000	-5.302651000	2.116533000
C	3.045455000	-3.371412000	1.196830000
C	2.971085000	-2.617549000	-2.547356000
H	2.917676000	-3.308879000	-3.393800000
H	2.087474000	-1.969491000	-2.582819000
H	3.846633000	-1.973741000	-2.693698000
C	2.957217000	-2.640030000	2.514683000
H	3.831687000	-1.997323000	2.671734000
H	2.073181000	-1.992604000	2.551091000
H	2.899283000	-3.339001000	3.354526000
C	4.389401000	1.757208000	0.009430000
C	4.446915000	2.453592000	1.260320000
C	4.602549000	3.842544000	1.216280000
H	4.658261000	4.416283000	2.129167000
C	4.692641000	4.532225000	0.023696000
C	4.614808000	3.853451000	-1.176015000
H	4.679833000	4.435751000	-2.082830000
C	4.459690000	2.464962000	-1.234434000
C	4.316847000	1.818973000	2.681713000
C	5.406745000	0.766482000	2.969874000
H	5.288409000	-0.146002000	2.388523000
H	6.405195000	1.168480000	2.766786000
H	5.364881000	0.485395000	4.028310000
C	4.473873000	2.875855000	3.797414000
H	4.375981000	2.372324000	4.764163000
H	5.456711000	3.359623000	3.780873000
H	3.697141000	3.646561000	3.759461000
C	2.912730000	1.215873000	2.879296000
H	2.811782000	0.850262000	3.908063000
H	2.137716000	1.971118000	2.708859000
H	2.719751000	0.379961000	2.210383000
C	4.344227000	1.843340000	-2.662867000
C	4.513247000	2.910300000	-3.767195000
H	3.736660000	3.681188000	-3.730138000
H	5.496172000	3.393175000	-3.736114000
H	4.424933000	2.415727000	-4.739471000
C	5.436657000	0.792960000	-2.949340000
H	5.407992000	0.524279000	-4.011429000
H	6.432882000	1.191243000	-2.728835000
H	5.309911000	-0.126122000	-2.380321000
C	2.942046000	1.242661000	-2.880642000
H	2.741141000	0.401564000	-2.220580000
H	2.165640000	1.997089000	-2.712943000
H	2.852313000	0.885262000	-3.913322000
H	-3.423548000	6.522481000	-0.033437000
H	3.423485000	-6.522489000	-0.032296000
H	-4.818152000	-5.611246000	0.030602000
H	4.818064000	5.611266000	0.029249000

Compound **2[a](Xyl, Xyl*) (pyr-S_1 geometry):**

Total energy (ωB97X-D/6-31G(d)): -3138.82518329

C	-7.015808000	0.040762000	-0.307847000
C	-5.900298000	-0.808101000	-0.256187000
C	-6.106852000	-2.189922000	-0.376506000
C	-7.391082000	-2.697416000	-0.542191000
C	-8.491951000	-1.847985000	-0.591951000
C	-8.295301000	-0.475319000	-0.473718000
B	-2.714230000	-1.068438000	0.040771000
C	-2.853490000	-2.640675000	-0.093671000
C	-3.029695000	-3.438907000	1.052481000
C	-3.149074000	-4.822962000	0.920744000
C	-3.089619000	-5.423524000	-0.331215000
C	-2.894500000	-4.640990000	-1.463180000
C	-2.772377000	-3.254909000	-1.358248000
C	-3.108455000	-2.801282000	2.418713000
C	-2.568546000	-2.419937000	-2.599601000
P	-4.252662000	-0.092140000	-0.041126000
C	8.115716000	1.421509000	-1.999111000
C	7.075550000	2.319848000	-1.776310000
C	5.938989000	1.925808000	-1.082153000
C	5.815819000	0.603572000	-0.610940000
C	6.884297000	-0.290939000	-0.825010000
C	8.015960000	0.116724000	-1.514472000

P	4.416775000	0.117589000	0.404235000	H	-5.269636000	-2.875701000	-0.341834000
B	2.746713000	1.278492000	0.400565000	H	-7.525361000	-3.771468000	-0.632881000
C	1.382984000	0.626903000	0.311782000	H	-9.491990000	-2.250793000	-0.721467000
C	0.229155000	1.465030000	0.399271000	H	-9.142659000	0.203493000	-0.510279000
C	-1.049035000	0.960254000	0.339649000	H	-6.882092000	1.114323000	-0.216985000
C	-1.310040000	-0.424865000	0.195163000	H	-3.117170000	-3.556353000	3.210838000
C	-0.174417000	-1.261757000	0.161570000	H	-2.257397000	-2.132931000	2.593284000
C	1.113352000	-0.766312000	0.222938000	H	-4.016608000	-2.195171000	2.521466000
C	-4.405604000	1.730492000	0.083256000	H	-3.290138000	-5.434489000	1.808883000
C	-4.370280000	2.524437000	-1.108917000	H	-2.836188000	-5.110166000	-2.442530000
C	-4.591653000	3.898723000	-0.974740000	H	-1.694964000	-1.765542000	-2.497245000
C	-4.829335000	4.486067000	0.251840000	H	-2.421237000	-3.049343000	-3.482667000
C	-4.835829000	3.716160000	1.397820000	H	-3.432573000	-1.771524000	-2.788114000
C	-4.623954000	2.334203000	1.364345000	H	-5.014191000	4.220795000	2.335576000
C	-4.074803000	2.011005000	-2.554198000	H	-4.584044000	4.541934000	-1.841955000
C	-4.614766000	1.603703000	2.745059000	C	-5.693967000	0.506704000	2.848648000
C	4.348343000	-1.708239000	0.292924000	H	-5.749711000	0.152755000	3.884514000
C	4.258264000	-2.382203000	-0.962067000	H	-6.679200000	0.897105000	2.571565000
C	4.513962000	-3.760159000	-0.966803000	H	-5.487349000	-0.357740000	2.220540000
C	4.782876000	-4.467922000	0.184748000	C	-3.218610000	1.023082000	3.041938000
C	4.763994000	-3.820366000	1.408445000	H	-2.927896000	0.249267000	2.334434000
C	4.547094000	-2.447520000	1.505659000	H	-2.456988000	1.810033000	3.013563000
C	3.823317000	-1.845697000	-2.361863000	H	-3.212027000	0.578833000	4.044498000
C	4.549600000	-1.824527000	2.927230000	C	-4.914639000	2.573866000	3.909189000
C	3.066107000	2.814083000	0.414320000	H	-5.909222000	3.026884000	3.831518000
C	3.694383000	3.420727000	1.529465000	H	-4.888691000	2.006476000	4.844760000
C	3.990415000	4.783552000	1.516383000	H	-4.167421000	3.369427000	3.997103000
C	3.668777000	5.568155000	0.417788000	C	-2.640566000	1.456072000	-2.651094000
C	3.045197000	4.988488000	-0.681692000	H	-1.909884000	2.211828000	-2.342197000
C	2.742334000	3.627691000	-0.703510000	H	-2.490479000	0.574102000	-2.032250000
C	4.027067000	2.608614000	2.751905000	H	-2.424889000	1.177118000	-3.689518000
C	2.142365000	3.041910000	-1.960296000	C	-5.098372000	0.962252000	-3.035235000
H	0.367811000	2.536937000	0.517378000	H	-5.013016000	0.010188000	-2.514723000
H	-1.878100000	1.659296000	0.395158000	H	-6.123543000	1.326184000	-2.906530000
H	-0.315624000	-2.336974000	0.077351000	H	-4.940420000	0.767196000	-4.102222000
H	1.933283000	-1.474406000	0.197226000	C	-4.147711000	3.151031000	-3.594088000
H	4.470043000	5.233495000	2.382167000	H	-5.142878000	3.606063000	-3.648890000
H	2.798574000	5.601740000	-1.544996000	H	-3.407222000	3.936356000	-3.409635000
H	1.220128000	2.489005000	-1.760883000	H	-3.928521000	2.730688000	-4.580602000
H	1.919027000	3.828449000	-2.687517000	C	3.455907000	-0.362125000	-2.506619000
H	2.839604000	2.339559000	-2.432762000	H	2.719272000	-0.030844000	-1.771772000
H	4.457608000	3.230541000	3.542280000	H	4.324964000	0.294446000	-2.475340000
H	3.135596000	2.112048000	3.150684000	H	2.991401000	-0.224165000	-3.489790000
H	4.752745000	1.815334000	2.519213000	C	4.947110000	-2.114149000	-3.385936000
H	6.820086000	-1.308515000	-0.452673000	H	5.860413000	-1.579890000	-3.103247000
H	8.826317000	-0.587802000	-1.676697000	H	5.190156000	-3.175568000	-3.488471000
H	7.146185000	3.338786000	-2.144659000	H	4.635923000	-1.752264000	-4.372295000
H	5.140997000	2.640122000	-0.914971000	C	2.543489000	-2.621253000	-2.764752000
H	4.933729000	-4.410548000	2.297798000	H	1.719557000	-2.380162000	-2.084589000
H	4.483887000	-4.304372000	-1.903201000	H	2.241149000	-2.326107000	-3.775688000

H	2.678345000	-3.705581000	-2.763952000
C	5.832985000	-1.000807000	3.157264000
H	5.822831000	-0.563590000	4.162516000
H	6.716390000	-1.643516000	3.071496000
H	5.943737000	-0.182521000	2.440632000
C	3.275513000	-0.991501000	3.164771000
H	3.161412000	-0.138310000	2.493399000
H	2.382609000	-1.613627000	3.041034000
H	3.280022000	-0.592240000	4.185383000
C	4.536939000	-2.903102000	4.030908000
H	5.455379000	-3.498805000	4.050337000
H	4.459943000	-2.405095000	5.002736000
H	3.679487000	-3.577789000	3.936800000
H	-3.186582000	-6.501734000	-0.423863000
H	3.900802000	6.629442000	0.416755000
H	-5.003681000	5.556486000	0.316287000
H	4.980054000	-5.534741000	0.135776000
H	9.002639000	1.735326000	-2.540931000

Compound **2**^{a}**(Xyl, Xyl*) (pyr-T_1 geometry):**

Total energy (ωB97X-D/6-31G(d)): -3138.76845332

C	-7.012870000	-0.048747000	0.406141000
C	-5.907458000	0.803574000	0.267723000
C	-6.123202000	2.188317000	0.318347000
C	-7.405481000	2.695012000	0.500897000
C	-8.496308000	1.841917000	0.636334000
C	-8.290744000	0.466417000	0.587725000
B	-2.732444000	1.072038000	-0.148083000
C	-2.879130000	2.647589000	-0.062231000
C	-3.096367000	3.404253000	-1.229248000
C	-3.219332000	4.791549000	-1.142874000
C	-3.123214000	5.436453000	0.084572000
C	-2.887514000	4.695160000	1.236423000
C	-2.760995000	3.306721000	1.176566000
C	-3.216105000	2.717549000	-2.568373000
C	-2.511363000	2.517310000	2.439276000
P	-4.260449000	0.089504000	0.038261000
C	8.112487000	-1.469574000	1.984542000
C	7.084399000	-2.373180000	1.726969000
C	5.932953000	-1.961616000	1.068749000
C	5.791434000	-0.620076000	0.666213000
C	6.843024000	0.282871000	0.917949000
C	7.988296000	-0.142144000	1.574512000
P	4.338986000	-0.091711000	-0.234182000
B	2.733192000	-1.260215000	-0.419232000
C	1.371813000	-0.613476000	-0.428535000
C	0.216324000	-1.454872000	-0.493430000
C	-1.062859000	-0.954294000	-0.431801000
C	-1.331479000	0.433606000	-0.319638000
C	-0.195491000	1.276038000	-0.325124000
C	1.091544000	0.784267000	-0.380853000
C	-4.407808000	-1.737280000	-0.028019000
C	-4.324583000	-2.494142000	1.185552000
C	-4.547536000	-3.872336000	1.103471000
C	-4.831383000	-4.498076000	-0.093982000
C	-4.882510000	-3.764292000	-1.262329000
C	-4.671523000	-2.381885000	-1.280230000
C	-3.976987000	-1.934111000	2.601511000
C	-4.714230000	-1.696376000	-2.683109000
C	4.359991000	1.735056000	-0.218709000
C	4.149103000	2.451148000	0.992298000
C	4.392288000	3.831667000	0.965251000
C	4.781039000	4.491182000	-0.180063000
C	4.912683000	3.790364000	-1.367766000
C	4.706219000	2.414442000	-1.430752000
C	3.633673000	1.947904000	2.374062000
C	4.852779000	1.723677000	-2.811232000
C	3.066833000	-2.804832000	-0.433459000
C	3.662381000	-3.419350000	-1.556964000
C	3.958081000	-4.783024000	-1.541536000
C	3.662732000	-5.559641000	-0.429217000
C	3.063324000	-4.971614000	0.678044000
C	2.765053000	-3.608093000	0.692902000
C	3.973344000	-2.614295000	-2.794054000
C	2.169466000	-3.006010000	1.943873000
H	0.357996000	-2.529911000	-0.578733000
H	-1.889186000	-1.658556000	-0.458513000
H	-0.340341000	2.353180000	-0.274115000
H	1.907666000	1.499541000	-0.385719000
H	4.416236000	-5.240971000	-2.414967000
H	2.828129000	-5.578218000	1.549427000
H	1.243194000	-2.461966000	1.735322000
H	1.952666000	-3.780280000	2.686504000
H	2.862939000	-2.290078000	2.401441000
H	4.312696000	-3.256612000	-3.612644000
H	3.091134000	-2.060789000	-3.132844000
H	4.763309000	-1.874485000	-2.610651000
H	6.755808000	1.317595000	0.602498000
H	8.788733000	0.565448000	1.767861000
H	7.175795000	-3.408644000	2.039496000
H	5.143010000	-2.677326000	0.872220000
H	5.185837000	4.342409000	-2.255833000
H	4.263500000	4.412384000	1.870703000
H	-5.294146000	2.877284000	0.215597000
H	-7.546320000	3.771536000	0.536165000
H	-9.495009000	2.244005000	0.778120000
H	-9.129871000	-0.215767000	0.691611000
H	-6.872677000	-1.124749000	0.370534000
H	-3.253193000	3.443373000	-3.386687000
H	-2.368618000	2.045398000	-2.745264000

H	-4.124799000	2.105685000	-2.619656000
H	-3.392473000	5.370443000	-2.047132000
H	-2.800866000	5.198897000	2.196404000
H	-1.639611000	1.861638000	2.329544000
H	-2.336181000	3.178527000	3.293692000
H	-3.365752000	1.873571000	2.680224000
H	-5.095528000	-4.297407000	-2.176871000
H	-4.504893000	-4.488283000	1.989346000
C	-5.803777000	-0.609505000	-2.784428000
H	-5.902859000	-0.294616000	-3.829684000
H	-6.774701000	-0.993868000	-2.453581000
H	-5.577666000	0.278191000	-2.197187000
C	-3.333159000	-1.117991000	-3.046865000
H	-3.020116000	-0.324989000	-2.370902000
H	-2.566964000	-1.900717000	-3.026195000
H	-3.366240000	-0.700321000	-4.060353000
C	-5.047164000	-2.705215000	-3.804688000
H	-6.037486000	-3.157560000	-3.681457000
H	-5.052198000	-2.168658000	-4.758681000
H	-4.300438000	-3.501548000	-3.889828000
C	-2.547681000	-1.358718000	2.623873000
H	-1.819798000	-2.114184000	2.307887000
H	-2.434485000	-0.494411000	1.973132000
H	-2.294598000	-1.045979000	3.644198000
C	-4.995703000	-0.884595000	3.091102000
H	-4.951887000	0.047807000	2.531145000
H	-6.018748000	-1.270743000	3.024481000
H	-4.792134000	-0.646709000	4.141678000
C	-3.992224000	-3.042798000	3.677024000
H	-4.979098000	-3.505229000	3.789742000
H	-3.252360000	-3.826452000	3.482917000
H	-3.734514000	-2.590719000	4.640011000
C	3.269101000	0.464744000	2.523417000
H	2.523840000	0.133576000	1.797010000
H	4.139606000	-0.193238000	2.492553000
H	2.816232000	0.327296000	3.511959000
C	4.708648000	2.243624000	3.441892000
H	5.632572000	1.698617000	3.217687000
H	4.952249000	3.306888000	3.520816000
H	4.351745000	1.913282000	4.423676000
C	2.330385000	2.720192000	2.693196000
H	1.552351000	2.471903000	1.963298000
H	1.967019000	2.428387000	3.684685000
H	2.461003000	3.805118000	2.697078000
C	6.078909000	0.788461000	-2.836270000
H	6.175580000	0.328132000	-3.825963000
H	6.994301000	1.354207000	-2.629814000
H	6.024019000	-0.023229000	-2.106692000
C	3.551423000	0.984328000	-3.178332000
H	3.264212000	0.200115000	-2.474393000
H	2.714833000	1.689324000	-3.225530000
H	3.658387000	0.509008000	-4.159924000
C	5.083958000	2.740766000	-3.947102000
H	6.030889000	3.280816000	-3.841846000
H	5.126755000	2.197403000	-4.896379000
H	4.268136000	3.467001000	-4.022508000
H	-3.223518000	6.516850000	0.142368000
H	3.893111000	-6.621402000	-0.426509000
H	-5.006367000	-5.570089000	-0.118215000
H	4.963223000	5.561440000	-0.157233000
H	9.010102000	-1.797591000	2.499717000

Compound **2^{a}(Xyl, Xyl*) (TICT-S$_1$ geometry):**

Total energy (ωB97X-D/6-31G(d)): -3138.78749494

P	3.923361000	-0.501140000	-0.334060000
C	1.257317000	0.379148000	-1.153127000
B	2.748944000	0.797953000	-1.050706000
C	0.224418000	1.313414000	-0.923202000
H	0.482080000	2.359105000	-0.770884000
C	-1.098778000	0.922988000	-0.824422000
H	-1.840671000	1.677189000	-0.581807000
C	4.973263000	-1.549399000	-1.362116000
C	5.948444000	-2.403995000	-0.827218000
H	6.124553000	-2.423630000	0.243528000
C	6.692540000	-3.222621000	-1.666168000
H	7.447778000	-3.878700000	-1.243948000
C	6.472650000	-3.205638000	-3.043152000
H	7.058536000	-3.846630000	-3.694728000
C	5.492152000	-2.375622000	-3.578037000
H	5.306103000	-2.368526000	-4.647538000
C	4.737301000	-1.559548000	-2.742729000
H	3.956118000	-0.927565000	-3.153772000
C	3.241070000	2.294902000	-1.248096000
C	3.627075000	2.796846000	-2.505571000
C	4.041748000	4.124108000	-2.634535000
H	4.340115000	4.498639000	-3.611075000
C	4.075587000	4.966943000	-1.532258000
C	3.680434000	4.487902000	-0.288928000
H	3.693075000	5.149724000	0.574004000
C	3.262236000	3.165717000	-0.133704000
C	3.631678000	1.905074000	-3.723416000
H	3.643500000	2.493063000	-4.646783000
H	2.751805000	1.253323000	-3.740905000
H	4.519884000	1.260800000	-3.731768000
C	2.842341000	2.684549000	1.236976000
H	3.500177000	1.889670000	1.607533000
H	1.828042000	2.270040000	1.222408000
H	2.863585000	3.502533000	1.964314000
C	4.538810000	-0.707301000	1.390838000
C	5.783887000	-0.114901000	1.751209000

C	6.272094000	-0.388342000	3.039235000
H	7.215464000	0.041233000	3.353045000
C	5.598921000	-1.186967000	3.935059000
C	4.372137000	-1.725248000	3.584163000
H	3.856291000	-2.332370000	4.314523000
C	3.802461000	-1.499255000	2.334292000
C	6.729906000	0.845299000	0.955020000
C	6.382925000	1.227010000	-0.491264000
H	5.400775000	1.688147000	-0.590218000
H	6.476821000	0.394174000	-1.189197000
H	7.102073000	1.987880000	-0.815414000
C	8.139561000	0.213824000	0.886252000
H	8.810373000	0.881804000	0.334863000
H	8.105124000	-0.741413000	0.350745000
H	8.589209000	0.037030000	1.866482000
C	6.783748000	2.183856000	1.729419000
H	7.510816000	2.853387000	1.256688000
H	7.073512000	2.063196000	2.776458000
H	5.807411000	2.679495000	1.701651000
C	2.384857000	-2.073508000	2.086518000
C	1.851903000	-2.888862000	3.282608000
H	1.764728000	-2.287447000	4.193204000
H	2.465872000	-3.770557000	3.497632000
H	0.845827000	-3.242365000	3.033063000
C	2.360638000	-3.056201000	0.899634000
H	1.334942000	-3.395938000	0.719861000
H	2.982600000	-3.930074000	1.124351000
H	2.719689000	-2.631331000	-0.036866000
C	1.405728000	-0.895611000	1.921127000
H	1.719635000	-0.173246000	1.170560000
H	1.313183000	-0.358311000	2.871934000
H	0.412742000	-1.252145000	1.627680000
P	-4.243091000	0.006820000	0.049756000
C	-1.495409000	-0.420342000	-0.990474000
B	-2.931888000	-0.979483000	-0.730811000
C	-0.481170000	-1.328716000	-1.340406000
H	-0.741206000	-2.367878000	-1.528124000
C	0.847782000	-0.941335000	-1.422274000
H	1.593881000	-1.684200000	-1.692872000
C	-5.897864000	-0.621521000	0.430936000
C	-6.815609000	0.219156000	1.076341000
H	-6.528469000	1.233594000	1.335776000
C	-8.093473000	-0.229647000	1.387463000
H	-8.786935000	0.440888000	1.886943000
C	-8.482960000	-1.525703000	1.063092000
H	-9.481471000	-1.876204000	1.306393000
C	-7.578217000	-2.366443000	0.422552000
H	-7.865881000	-3.380623000	0.161019000
C	-6.297663000	-1.925090000	0.105962000
H	-5.615628000	-2.600745000	-0.395084000
C	-3.261718000	-2.493132000	-1.058019000
C	-3.021741000	-3.500897000	-0.103903000
C	-3.318751000	-4.829051000	-0.412051000
H	-3.136200000	-5.603470000	0.329217000
C	-3.844627000	-5.169188000	-1.652747000
C	-4.063391000	-4.179895000	-2.604066000
H	-4.463350000	-4.445314000	-3.579794000
C	-3.773857000	-2.844618000	-2.321279000
C	-2.456076000	-3.152394000	1.252292000
H	-2.230071000	-4.052699000	1.832215000
H	-1.532899000	-2.568018000	1.156835000
H	-3.158600000	-2.543876000	1.833701000
C	-4.029350000	-1.779707000	-3.360457000
H	-4.841235000	-1.110266000	-3.051156000
H	-3.141461000	-1.155690000	-3.514894000
H	-4.306042000	-2.220404000	-4.323073000
C	-4.137449000	1.772958000	0.526305000
C	-4.541766000	2.780997000	-0.408776000
C	-4.510442000	4.110111000	0.025048000
H	-4.811383000	4.909292000	-0.635523000
C	-4.100318000	4.456534000	1.297007000
C	-3.693884000	3.479212000	2.183154000
H	-3.372666000	3.797991000	3.163456000
C	-3.693676000	2.122841000	1.842866000
C	-4.981111000	2.550627000	-1.890901000
C	-6.205401000	1.621270000	-2.020173000
H	-5.979581000	0.580646000	-1.796170000
H	-7.016003000	1.940863000	-1.356620000
H	-6.577803000	1.658519000	-3.050224000
C	-5.405381000	3.870096000	-2.573602000
H	-5.702846000	3.644623000	-3.602388000
H	-6.264864000	4.338284000	-2.081105000
H	-4.588681000	4.596964000	-2.630954000
C	-3.804081000	2.013096000	-2.727847000
H	-4.112604000	1.918547000	-3.775816000
H	-2.951645000	2.699863000	-2.685637000
H	-3.465830000	1.034577000	-2.392354000
C	-3.175926000	1.137932000	2.939304000
C	-2.831650000	1.871962000	4.254196000
H	-2.024172000	2.600922000	4.129517000
H	-3.700099000	2.374503000	4.694178000
H	-2.482998000	1.130757000	4.980155000
C	-4.227479000	0.080954000	3.333292000
H	-3.865881000	-0.480967000	4.202164000
H	-5.175782000	0.554897000	3.609211000
H	-4.429713000	-0.639228000	2.542571000
C	-1.866628000	0.460924000	2.491327000
H	-2.004434000	-0.180474000	1.623902000
H	-1.106531000	1.207594000	2.236027000
H	-1.474656000	-0.158491000	3.307200000

H	4.401095000	5.997742000	-1.641977000
H	-4.075287000	-6.205943000	-1.881295000
H	6.020035000	-1.381760000	4.916953000
H	-4.091804000	5.500150000	1.598722000

Compound **2^{a}(Xyl, Xyl*) (TICT-T_1 geometry):**

Total energy (ωB97X-D/6-31G(d)): -3138.77681044

P	4.056278000	0.512347000	0.271872000
C	1.208240000	-0.257124000	0.150675000
B	2.643106000	-0.785190000	0.185999000
C	0.136010000	-1.183236000	0.037270000
H	0.367223000	-2.243191000	-0.039347000
C	-1.183034000	-0.779593000	0.015919000
H	-1.951434000	-1.541537000	-0.075065000
C	4.379088000	1.461113000	1.752296000
C	5.493906000	2.305938000	1.878108000
H	6.217463000	2.367725000	1.070083000
C	5.672334000	3.049518000	3.035743000
H	6.538650000	3.697020000	3.131671000
C	4.743315000	2.966779000	4.073821000
H	4.887253000	3.551186000	4.977430000
C	3.628573000	2.140647000	3.948535000
H	2.900650000	2.081095000	4.751613000
C	3.439171000	1.393290000	2.792829000
H	2.564336000	0.757361000	2.683597000
C	3.045029000	-2.317918000	0.171747000
C	2.973105000	-3.090586000	1.350318000
C	3.316724000	-4.442760000	1.323679000
H	3.264851000	-5.027015000	2.239466000
C	3.718174000	-5.048272000	0.139337000
C	3.768444000	-4.302793000	-1.032024000
H	4.067113000	-4.778513000	-1.963339000
C	3.437980000	-2.945851000	-1.028630000
C	2.538191000	-2.456609000	2.650121000
H	2.474073000	-3.198155000	3.452695000
H	1.558550000	-1.977249000	2.545604000
H	3.244170000	-1.678822000	2.969022000
C	3.499366000	-2.164954000	-2.320461000
H	4.322543000	-1.439823000	-2.314715000
H	2.574942000	-1.600129000	-2.482043000
H	3.650345000	-2.829522000	-3.177245000
C	5.471601000	0.646120000	-0.874488000
C	6.637461000	-0.145480000	-0.674258000
C	7.638779000	-0.065654000	-1.654185000
H	8.533161000	-0.667883000	-1.552542000
C	7.529346000	0.748958000	-2.757777000
C	6.398881000	1.531753000	-2.927099000
H	6.352699000	2.167622000	-3.799240000
C	5.346162000	1.508948000	-2.015868000
C	6.998335000	-1.141636000	0.471512000
C	6.050765000	-1.264379000	1.671795000
H	5.045079000	-1.576886000	1.387804000
H	6.007460000	-0.354388000	2.271778000
H	6.434829000	-2.059071000	2.321293000
C	8.360314000	-0.722181000	1.073180000
H	8.617679000	-1.397903000	1.896037000
H	8.304539000	0.294890000	1.477576000
H	9.184304000	-0.755878000	0.356271000
C	7.099922000	-2.554611000	-0.144802000
H	7.429619000	-3.267721000	0.619050000
H	7.810428000	-2.602979000	-0.974938000
H	6.122459000	-2.885107000	-0.510388000
C	4.118148000	2.410388000	-2.324797000
C	4.345358000	3.291423000	-3.570888000
H	4.482658000	2.700281000	-4.481902000
H	5.199712000	3.967177000	-3.454057000
H	3.455782000	3.910553000	-3.723228000
C	3.835186000	3.401862000	-1.178184000
H	3.023781000	4.077314000	-1.469781000
H	4.723412000	4.006490000	-0.963016000
H	3.526690000	2.925022000	-0.247824000
C	2.884505000	1.543990000	-2.643399000
H	2.596476000	0.872819000	-1.833198000
H	3.077519000	0.927314000	-3.528233000
H	2.020069000	2.184368000	-2.851129000
P	-4.475773000	0.031882000	-0.043866000
C	-1.555759000	0.580631000	0.102685000
B	-3.017938000	1.116256000	0.084208000
C	-0.495493000	1.504756000	0.218381000
H	-0.726774000	2.564800000	0.293457000
C	0.827375000	1.105321000	0.247928000
H	1.594510000	1.870579000	0.354899000
C	-6.185836000	0.626916000	-0.071167000
C	-7.232099000	-0.302000000	-0.167683000
H	-7.009282000	-1.362922000	-0.225514000
C	-8.556590000	0.117924000	-0.191032000
H	-9.348430000	-0.621943000	-0.266661000
C	-8.867991000	1.472263000	-0.118430000
H	-9.903070000	1.800021000	-0.136654000
C	-7.836369000	2.400801000	-0.022438000
H	-8.060258000	3.462141000	0.034918000
C	-6.507823000	1.989590000	0.001195000
H	-5.726846000	2.735814000	0.075935000
C	-3.290498000	2.673270000	0.183639000
C	-3.343351000	3.459356000	-0.983200000
C	-3.584316000	4.830162000	-0.883588000
H	-3.629483000	5.432230000	-1.788101000
C	-3.766981000	5.429633000	0.357024000
C	-3.694713000	4.660341000	1.512347000
H	-3.826233000	5.129393000	2.484630000

C	-3.455108000	3.287620000	1.439621000
C	-3.159033000	2.821802000	-2.339314000
H	-3.103931000	3.576490000	-3.129955000
H	-2.241334000	2.223277000	-2.373929000
H	-3.989876000	2.146327000	-2.576120000
C	-3.391771000	2.464380000	2.703600000
H	-4.236726000	1.768170000	2.766006000
H	-2.477504000	1.860585000	2.736960000
H	-3.413263000	3.099964000	3.594250000
C	-4.479274000	-1.796362000	-0.179711000
C	-4.558139000	-2.591620000	1.009506000
C	-4.662065000	-3.977350000	0.852690000
H	-4.732379000	-4.622435000	1.715718000
C	-4.681848000	-4.573944000	-0.392132000
C	-4.579092000	-3.800870000	-1.531340000
H	-4.586551000	-4.312208000	-2.482302000
C	-4.470801000	-2.407712000	-1.475552000
C	-4.507270000	-2.065468000	2.479270000
C	-5.663265000	-1.098091000	2.805791000
H	-5.578600000	-0.141133000	2.294293000
H	-6.630444000	-1.538314000	2.539811000
H	-5.669582000	-0.894180000	3.882769000
C	-4.641014000	-3.213077000	3.504581000
H	-4.591619000	-2.783112000	4.509905000
H	-5.598798000	-3.738860000	3.423260000
H	-3.827514000	-3.942049000	3.428073000
C	-3.145956000	-1.406573000	2.774266000
H	-3.101040000	-1.115978000	3.830787000
H	-2.325294000	-2.105183000	2.577595000
H	-2.975441000	-0.513625000	2.176749000
C	-4.316811000	-1.672158000	-2.844907000
C	-4.377971000	-2.655947000	-4.034434000
H	-3.571318000	-3.395974000	-4.010117000
H	-5.338865000	-3.178634000	-4.097493000
H	-4.258584000	-2.083855000	-4.959846000
C	-5.445526000	-0.654312000	-3.107312000
H	-5.379579000	-0.302426000	-4.143299000
H	-6.429955000	-1.114386000	-2.969031000
H	-5.391487000	0.221153000	-2.463075000
C	-2.937331000	-0.992813000	-2.943134000
H	-2.807238000	-0.202059000	-2.207525000
H	-2.133466000	-1.722782000	-2.798334000
H	-2.818206000	-0.547166000	-3.938041000
H	3.981838000	-6.102261000	0.127089000
H	-3.957033000	6.497303000	0.423945000
H	8.327258000	0.780258000	-3.493833000
H	-4.770057000	-5.653592000	-0.475159000

Compound **2**^{b}**(Xyl, Xyl*) (S$_0$ geometry):**

Total energy (ωB97X-D/6-31G(d)): -3138.85109619

C	6.642570000	2.334983000	-0.241523000
C	5.890460000	1.156437000	-0.096266000
C	6.607162000	-0.044105000	0.057557000
C	7.995601000	-0.067353000	0.065851000
C	8.715030000	1.117161000	-0.080938000
C	8.033985000	2.320305000	-0.235103000
B	4.322767000	1.266254000	-0.118203000
C	3.632669000	2.683645000	-0.256430000
C	3.299250000	3.195009000	-1.524832000
C	2.673504000	4.438203000	-1.625394000
C	2.380777000	5.179219000	-0.486804000
C	2.728476000	4.687096000	0.765857000
C	3.354906000	3.447027000	0.894014000
C	3.593575000	2.397041000	-2.772315000
C	3.703630000	2.915922000	2.263621000
P	3.219060000	-0.167558000	-0.004937000
C	1.415569000	-0.046183000	-0.013161000
C	0.649957000	-1.218596000	-0.009813000
C	-0.736536000	-1.178542000	-0.008047000
C	-1.415519000	0.046191000	-0.013166000
C	-0.649909000	1.218603000	-0.009750000
C	0.736586000	1.178550000	-0.007982000
P	-3.219011000	0.167550000	-0.004913000
B	-4.322713000	-1.266278000	-0.118206000
C	-5.890404000	-1.156452000	-0.096290000
C	-6.642523000	-2.334978000	-0.241658000
C	-8.033938000	-2.320283000	-0.235277000
C	-8.714973000	-1.117143000	-0.081038000
C	-7.995534000	0.067351000	0.065865000
C	-6.607096000	0.044087000	0.057608000
C	3.691524000	-1.928356000	0.176944000
C	3.800433000	-2.493849000	1.488893000
C	4.053924000	-3.866084000	1.577951000
C	4.204226000	-4.657761000	0.456787000
C	4.120379000	-4.098043000	-0.802485000
C	3.866736000	-2.736154000	-0.993283000
C	3.695103000	-1.721295000	2.842600000
C	3.827365000	-2.241122000	-2.474082000
C	-3.691514000	1.928342000	0.176974000
C	-3.800438000	2.493817000	1.488933000
C	-4.053932000	3.866052000	1.578011000
C	-4.204217000	4.657750000	0.456863000
C	-4.120386000	4.098046000	-0.802416000
C	-3.866764000	2.736158000	-0.993239000
C	-3.695171000	1.721258000	2.842644000
C	-3.827488000	2.241164000	-2.474052000
C	-3.632626000	-2.683667000	-0.256505000
C	-3.299290000	-3.194989000	-1.524947000
C	-2.673512000	-4.438162000	-1.625590000
C	-2.380679000	-5.179198000	-0.487042000

C	-2.728306000	-4.687120000	0.765658000
C	-3.354759000	-3.447073000	0.893896000
C	-3.593717000	-2.396993000	-2.772389000
C	-3.703381000	-2.916007000	2.263544000
H	-6.122468000	-3.281723000	-0.362165000
H	-8.585822000	-3.248854000	-0.350501000
H	-9.801271000	-1.100453000	-0.074872000
H	-8.519056000	1.011482000	0.187268000
H	-6.071186000	0.981102000	0.173417000
H	-2.412936000	-4.827375000	-2.606890000
H	-2.510079000	-5.270772000	1.656761000
H	-4.749503000	-2.592213000	2.311349000
H	-3.548221000	-3.675158000	3.036097000
H	-3.086894000	-2.045598000	2.519436000
H	-3.423953000	-2.992774000	-3.674357000
H	-4.632935000	-2.048684000	-2.786886000
H	-2.956214000	-1.506972000	-2.832825000
H	-1.138549000	2.187789000	-0.003891000
H	1.280698000	2.114201000	0.000046000
H	1.138586000	-2.187785000	-0.004001000
H	-1.280638000	-2.114196000	-0.000073000
H	-4.253498000	4.754352000	-1.649323000
H	-4.135635000	4.346605000	2.541378000
H	6.071261000	-0.981138000	0.173270000
H	8.519129000	-1.011487000	0.187195000
H	9.801328000	1.100483000	-0.074746000
H	8.585859000	3.248892000	-0.350239000
H	6.122507000	3.281731000	-0.361972000
H	3.423693000	2.992828000	-3.674257000
H	4.632804000	2.048766000	-2.786922000
H	2.956101000	1.506998000	-2.832699000
H	2.412867000	4.827453000	-2.606663000
H	2.510328000	5.270727000	1.656992000
H	4.749759000	2.592138000	2.311342000
H	3.548518000	3.675048000	3.036209000
H	3.087173000	2.045500000	2.519534000
H	4.253478000	-4.754343000	-1.649398000
H	4.135602000	-4.346658000	2.541310000
C	2.466205000	-1.626350000	-2.858606000
H	2.452688000	-1.427569000	-3.936363000
H	1.646042000	-2.316363000	-2.632527000
H	2.261262000	-0.684457000	-2.352931000
C	4.971243000	-1.245703000	-2.746735000
H	4.869522000	-0.325995000	-2.174791000
H	5.941261000	-1.691853000	-2.501460000
H	4.979300000	-0.977824000	-3.809852000
C	4.041490000	-3.400012000	-3.472518000
H	3.256824000	-4.161494000	-3.405517000
H	4.009222000	-2.990202000	-4.486811000
H	5.017044000	-3.882850000	-3.353170000
C	4.845257000	-0.704749000	2.976670000
H	5.816641000	-1.204363000	2.892024000
H	4.801583000	0.077198000	2.221260000
H	4.796015000	-0.221627000	3.959698000
C	2.330055000	-1.027091000	3.032494000
H	2.196217000	-0.159712000	2.389015000
H	1.504368000	-1.720031000	2.838354000
H	2.243901000	-0.679872000	4.068385000
C	3.836810000	-2.668464000	4.054629000
H	3.039613000	-3.418866000	4.094693000
H	4.806008000	-3.177229000	4.080677000
H	3.765907000	-2.070395000	4.968391000
C	-4.845329000	0.704713000	2.976677000
H	-5.816710000	1.204336000	2.892049000
H	-4.801664000	-0.077210000	2.221243000
H	-4.796088000	0.221562000	3.959691000
C	-2.330125000	1.027071000	3.032612000
H	-2.196240000	0.159678000	2.389164000
H	-1.504443000	1.720022000	2.838494000
H	-2.244012000	0.679878000	4.068516000
C	-3.836937000	2.668428000	4.054668000
H	-4.806173000	3.177124000	4.080717000
H	-3.765986000	2.070371000	4.968433000
H	-3.039795000	3.418889000	4.094726000
C	-2.466346000	1.626402000	-2.858656000
H	-2.452839000	1.427721000	-3.936431000
H	-1.646166000	2.316377000	-2.632514000
H	-2.261418000	0.684462000	-2.353067000
C	-4.971383000	1.245757000	-2.746670000
H	-4.869624000	0.326047000	-2.174732000
H	-5.941390000	1.691903000	-2.501345000
H	-4.979488000	0.977888000	-3.809789000
C	-4.041669000	3.400084000	-3.472433000
H	-3.257024000	4.161588000	-3.405418000
H	-4.009409000	2.990313000	-4.486742000
H	-5.017235000	3.882887000	-3.353040000
H	1.889993000	6.144289000	-0.575951000
H	-1.889871000	-6.144250000	-0.576249000
H	4.393926000	-5.721850000	0.565820000
H	-4.393916000	5.721838000	0.565913000

Compound **2^b(Xyl, Xyl*) (pyr-S$_1$ geometry):**

Total energy (ωB97X-D/6-31G(d)): -3138.82909787

C	6.369904000	-2.402070000	-0.598497000
C	5.659833000	-1.201104000	-0.424205000
C	6.365326000	-0.002560000	-0.637276000
C	7.705166000	-0.002591000	-1.001046000
C	8.383740000	-1.208839000	-1.166278000
C	7.711981000	-2.410452000	-0.964788000
B	4.150525000	-1.286665000	-0.010127000

C	3.462587000	-2.688583000	0.235768000
C	3.497858000	-3.278876000	1.513633000
C	2.831910000	-4.485478000	1.734429000
C	2.137256000	-5.110278000	0.705336000
C	2.126591000	-4.544721000	-0.564398000
C	2.789331000	-3.342923000	-0.813807000
C	4.213976000	-2.592932000	2.652851000
C	2.767292000	-2.743871000	-2.198748000
P	3.120795000	0.182640000	0.328499000
C	1.365983000	0.072723000	0.695490000
C	0.581012000	1.237322000	0.672925000
C	-0.796698000	1.173670000	0.761916000
C	-1.452568000	-0.072374000	0.854566000
C	-0.654198000	-1.217387000	1.051050000
C	0.721903000	-1.148717000	0.973663000
P	-3.228976000	-0.174208000	0.775318000
B	-4.210987000	1.396208000	-0.039344000
C	-5.253905000	1.174121000	-1.134060000
C	-5.965601000	2.307832000	-1.620482000
C	-6.980902000	2.194298000	-2.553951000
C	-7.350269000	0.942867000	-3.055215000
C	-6.689706000	-0.190875000	-2.586075000
C	-5.674201000	-0.080447000	-1.646769000
C	3.532667000	1.934897000	-0.010800000
C	3.298917000	2.501601000	-1.307389000
C	3.501090000	3.878190000	-1.446357000
C	3.922649000	4.670413000	-0.396499000
C	4.175031000	4.107554000	0.838251000
C	3.992925000	2.742237000	1.078526000
C	2.876884000	1.735868000	-2.603627000
C	4.322699000	2.239588000	2.519973000
C	-3.560845000	-1.910927000	0.277055000
C	-2.930948000	-2.508770000	-0.859530000
C	-2.999741000	-3.905076000	-0.964000000
C	-3.684454000	-4.687096000	-0.059323000
C	-4.380474000	-4.086368000	0.975164000
C	-4.349748000	-2.707187000	1.171563000
C	-2.239957000	-1.858466000	-2.104567000
C	-5.173140000	-2.142464000	2.360326000
C	-3.711247000	2.781049000	0.505048000
C	-3.905079000	3.158486000	1.857234000
C	-3.439748000	4.388630000	2.321517000
C	-2.786332000	5.268879000	1.470286000
C	-2.587814000	4.914782000	0.140120000
C	-3.031913000	3.688840000	-0.352745000
C	-4.638895000	2.247990000	2.804167000
C	-2.688451000	3.316934000	-1.775871000
H	-5.711606000	3.291365000	-1.233366000
H	-7.494674000	3.088689000	-2.897114000
H	-8.146005000	0.855207000	-3.788840000

H	-6.972907000	-1.175169000	-2.951101000
H	-5.202306000	-0.989796000	-1.292445000
H	-3.602674000	4.661510000	3.361474000
H	-2.062975000	5.596466000	-0.525092000
H	-3.570536000	3.039655000	-2.359985000
H	-2.187679000	4.146012000	-2.285650000
H	-2.011628000	2.452840000	-1.794937000
H	-4.752388000	2.703613000	3.792554000
H	-5.634960000	2.000980000	2.420617000
H	-4.106104000	1.294895000	2.935199000
H	-1.118760000	-2.184448000	1.212878000
H	1.291766000	-2.061106000	1.085689000
H	1.049702000	2.205172000	0.527523000
H	-1.370773000	2.089927000	0.698762000
H	-4.947081000	-4.720068000	1.642681000
H	-2.511915000	-4.400085000	-1.794920000
H	5.857765000	0.950130000	-0.520491000
H	8.222632000	0.939548000	-1.157784000
H	9.431967000	-1.210165000	-1.451658000
H	8.233135000	-3.354928000	-1.092167000
H	5.855739000	-3.346729000	-0.441327000
H	4.299105000	-3.251359000	3.522467000
H	5.223978000	-2.283086000	2.361081000
H	3.680087000	-1.687873000	2.968579000
H	2.854053000	-4.935066000	2.724119000
H	1.596210000	-5.039775000	-1.374199000
H	3.782000000	-2.532679000	-2.556167000
H	2.287827000	-3.417432000	-2.915418000
H	2.216799000	-1.796944000	-2.214588000
H	4.513476000	4.762918000	1.626747000
H	3.326808000	4.362641000	-2.395190000
C	3.080289000	1.666683000	3.235525000
H	3.312496000	1.511551000	4.295238000
H	2.237907000	2.364292000	3.173316000
H	2.754760000	0.708794000	2.832135000
C	5.470692000	1.210811000	2.501841000
H	5.212285000	0.297478000	1.968693000
H	6.364705000	1.636553000	2.033123000
H	5.727893000	0.931701000	3.530261000
C	4.817667000	3.388420000	3.426031000
H	4.055819000	4.160526000	3.578219000
H	5.056588000	2.972643000	4.409720000
H	5.730343000	3.858988000	3.045625000
C	3.966224000	0.725343000	-3.007235000
H	4.933027000	1.225095000	-3.132345000
H	4.092300000	-0.067970000	-2.274747000
H	3.699030000	0.256749000	-3.961637000
C	1.506319000	1.038877000	-2.482506000
H	1.500229000	0.212804000	-1.774448000
H	0.729168000	1.748038000	-2.177659000

H	1.222879000	0.631117000	-3.459774000
C	2.731057000	2.688412000	-3.811206000
H	1.943837000	3.435632000	-3.663575000
H	3.666733000	3.200166000	-4.058999000
H	2.450843000	2.092948000	-4.685583000
C	-2.069174000	-0.333433000	-2.160128000
H	-2.987026000	0.215577000	-1.937252000
H	-1.268620000	0.022951000	-1.513302000
H	-1.791454000	-0.063505000	-3.185616000
C	-0.823820000	-2.450601000	-2.289804000
H	-0.205786000	-2.274093000	-1.404473000
H	-0.825980000	-3.525742000	-2.491689000
H	-0.337375000	-1.961882000	-3.141860000
C	-3.112923000	-2.218733000	-3.333411000
H	-4.086365000	-1.721401000	-3.272864000
H	-2.616511000	-1.872494000	-4.246966000
H	-3.284248000	-3.293486000	-3.435123000
C	-4.242619000	-1.706499000	3.510364000
H	-4.835425000	-1.307708000	4.341915000
H	-3.668865000	-2.564593000	3.878311000
H	-3.531677000	-0.933719000	3.207205000
C	-6.096676000	-1.001059000	1.893917000
H	-5.574145000	-0.137791000	1.476324000
H	-6.785305000	-1.359240000	1.121019000
H	-6.688451000	-0.634334000	2.740360000
C	-6.119217000	-3.202443000	2.963687000
H	-5.580988000	-4.023232000	3.448940000
H	-6.730591000	-2.723164000	3.735082000
H	-6.800230000	-3.620240000	2.214597000
H	1.613515000	-6.043788000	0.890250000
H	-2.430672000	6.226473000	1.840051000
H	4.061704000	5.737410000	-0.545171000
H	-3.702402000	-5.766865000	-0.174898000

Compound **2^{b}(Xyl, Xyl*) (pyr-T$_1$ geometry):**

Total energy (ωB97X-D/6-31G(d)): -3138.76780796

C	6.357129000	2.393746000	0.626273000
C	5.650306000	1.191554000	0.449218000
C	6.355237000	-0.005837000	0.669106000
C	7.692611000	-0.003591000	1.041962000
C	8.368275000	1.203885000	1.209774000
C	7.696674000	2.404435000	1.001882000
B	4.143760000	1.273647000	0.023192000
C	3.456140000	2.674280000	-0.230500000
C	3.501360000	3.262268000	-1.509144000
C	2.833790000	4.466563000	-1.737960000
C	2.128221000	5.091270000	-0.716288000
C	2.108310000	4.528319000	0.554556000
C	2.772252000	3.328917000	0.812097000
C	4.230333000	2.577373000	-2.640927000
C	2.738548000	2.732339000	2.197845000
P	3.127297000	-0.199640000	-0.339786000
C	1.368105000	-0.087958000	-0.704896000
C	0.585386000	-1.254969000	-0.689986000
C	-0.793260000	-1.191844000	-0.765038000
C	-1.445319000	0.054314000	-0.845583000
C	-0.653583000	1.202898000	-1.035302000
C	0.724511000	1.134438000	-0.968102000
P	-3.207114000	0.170139000	-0.644840000
B	-4.242053000	-1.368494000	0.049977000
C	-5.334046000	-1.136082000	1.090599000
C	-6.034007000	-2.267604000	1.600321000
C	-7.053395000	-2.150114000	2.530297000
C	-7.443587000	-0.895976000	3.007596000
C	-6.794305000	0.237486000	2.516850000
C	-5.775149000	0.122551000	1.583174000
C	3.527987000	-1.950415000	0.020244000
C	3.274485000	-2.508990000	1.316943000
C	3.466219000	-3.886108000	1.464676000
C	3.896735000	-4.686079000	0.424341000
C	4.169196000	-4.131014000	-0.809612000
C	3.997839000	-2.765934000	-1.058875000
C	2.842066000	-1.735233000	2.605178000
C	4.346655000	-2.272275000	-2.498961000
C	-3.525980000	1.935855000	-0.275028000
C	-2.967063000	2.554771000	0.881581000
C	-3.054179000	3.952118000	0.957641000
C	-3.680411000	4.710887000	-0.006807000
C	-4.285218000	4.088142000	-1.085467000
C	-4.234268000	2.705997000	-1.252444000
C	-2.310022000	1.925150000	2.152078000
C	-4.952812000	2.103808000	-2.488042000
C	-3.731991000	-2.767749000	-0.487180000
C	-3.966437000	-3.182440000	-1.817404000
C	-3.496624000	-4.417211000	-2.268177000
C	-2.802203000	-5.266312000	-1.417369000
C	-2.573264000	-4.879154000	-0.102368000
C	-3.022222000	-3.645179000	0.370423000
C	-4.744637000	-2.304888000	-2.765725000
C	-2.678612000	-3.240411000	1.784607000
H	-5.760786000	-3.256356000	1.239101000
H	-7.554321000	-3.045841000	2.889729000
H	-8.242513000	-0.804723000	3.737454000
H	-7.091621000	1.225444000	2.861471000
H	-5.316400000	1.035237000	1.215529000
H	-3.687503000	-4.718985000	-3.295520000
H	-2.027576000	-5.540324000	0.567082000
H	-3.565241000	-2.948625000	2.355432000
H	-2.180128000	-4.057183000	2.316798000
H	-2.002126000	-2.376143000	1.787336000

H	-4.943895000	-2.818617000	-3.711635000
H	-5.701920000	-2.007348000	-2.324701000
H	-4.203179000	-1.380060000	-3.003224000
H	-1.120774000	2.171260000	-1.179759000
H	1.292927000	2.048127000	-1.074624000
H	1.056233000	-2.223281000	-0.556333000
H	-1.369362000	-2.107313000	-0.700163000
H	-4.797831000	4.707158000	-1.808066000
H	-2.623981000	4.465471000	1.809039000
H	5.849309000	-0.959243000	0.550843000
H	8.210267000	-0.944707000	1.203882000
H	9.414506000	1.206991000	1.502320000
H	8.215857000	3.349664000	1.131377000
H	5.842885000	3.337452000	0.463802000
H	4.327097000	3.237569000	-3.507938000
H	5.236266000	2.265549000	-2.337543000
H	3.699300000	1.673696000	-2.965181000
H	2.863968000	4.914711000	-2.728079000
H	1.570065000	5.023744000	1.358909000
H	3.749653000	2.515393000	2.561817000
H	2.259348000	3.410210000	2.910569000
H	2.181697000	1.789074000	2.212558000
H	4.514061000	-4.792587000	-1.590047000
H	3.276267000	-4.365157000	2.413182000
C	3.112748000	-1.704103000	-3.232999000
H	3.357698000	-1.554281000	-4.290591000
H	2.270599000	-2.402630000	-3.177693000
H	2.781922000	-0.744057000	-2.838783000
C	5.495372000	-1.244282000	-2.473586000
H	5.232813000	-0.327288000	-1.948849000
H	6.383423000	-1.668715000	-1.992511000
H	5.764728000	-0.971667000	-3.500606000
C	4.852137000	-3.427003000	-3.391687000
H	4.091094000	-4.198486000	-3.550609000
H	5.105907000	-3.017185000	-4.374158000
H	5.758245000	-3.897201000	-2.995490000
C	3.932502000	-0.728851000	3.015788000
H	4.895292000	-1.233177000	3.152842000
H	4.070514000	0.060716000	2.281564000
H	3.658177000	-0.254918000	3.965486000
C	1.476623000	-1.031293000	2.467211000
H	1.483087000	-0.208009000	1.756001000
H	0.698478000	-1.737424000	2.158034000
H	1.185929000	-0.617560000	3.439761000
C	2.678205000	-2.681498000	3.815426000
H	1.887987000	-3.424548000	3.663193000
H	3.608231000	-3.197611000	4.075038000
H	2.392641000	-2.080455000	4.684205000
C	-2.152720000	0.399874000	2.230219000
H	-3.083244000	-0.139857000	2.038650000
H	-1.366837000	0.021988000	1.575493000
H	-1.854420000	0.145285000	3.253677000
C	-0.893732000	2.514398000	2.341826000
H	-0.261705000	2.300765000	1.473757000
H	-0.893353000	3.596862000	2.500078000
H	-0.424580000	2.055819000	3.219859000
C	-3.205191000	2.304484000	3.357487000
H	-4.182947000	1.818366000	3.273209000
H	-2.735613000	1.956620000	4.284353000
H	-3.365187000	3.381695000	3.451278000
C	-3.931911000	1.547866000	-3.501909000
H	-4.456021000	1.135715000	-4.371689000
H	-3.266501000	2.346728000	-3.847989000
H	-3.304607000	0.751770000	-3.092523000
C	-5.975936000	1.038933000	-2.047625000
H	-5.545451000	0.194482000	-1.504351000
H	-6.729688000	1.487152000	-1.391613000
H	-6.484767000	0.627233000	-2.926625000
C	-5.768331000	3.162034000	-3.258810000
H	-5.137178000	3.937722000	-3.705333000
H	-6.295090000	2.664275000	-4.079259000
H	-6.523660000	3.640572000	-2.626760000
H	1.603793000	6.023114000	-0.907511000
H	-2.443638000	-6.227496000	-1.775317000
H	4.026824000	-5.753164000	0.580057000
H	-3.717991000	5.791992000	0.089333000

Compound **2[b](Xyl, Xyl*) (TICT-S_1 geometry):**

Total energy (ωB97X-D/6-31G(d)): -3138.79103897

C	-5.388177000	-3.414079000	0.478788000
C	-4.588963000	-2.316949000	0.879341000
C	-4.135055000	-2.349113000	2.215788000
C	-4.488355000	-3.361431000	3.099019000
C	-5.301337000	-4.409989000	2.677615000
C	-5.739912000	-4.431697000	1.353961000
B	-4.183546000	-1.249831000	-0.177269000
C	-4.561564000	-1.412146000	-1.718785000
C	-5.904357000	-1.191594000	-2.107495000
C	-6.278930000	-1.314650000	-3.447016000
C	-5.349374000	-1.659717000	-4.418123000
C	-4.035484000	-1.909262000	-4.044476000
C	-3.636107000	-1.801365000	-2.711443000
C	-6.971494000	-0.832894000	-1.097884000
C	-2.203494000	-2.125421000	-2.361625000
P	-3.212032000	0.291855000	0.362705000
C	-1.407648000	0.239225000	0.324278000
C	-0.580487000	1.361961000	0.198070000
C	0.798108000	1.227339000	0.151950000
C	1.397660000	-0.038520000	0.247307000
C	0.570590000	-1.153184000	0.444166000
C	-0.807242000	-1.016323000	0.483775000

P	3.180548000	-0.286991000	0.108883000	H	4.131647000	-4.536839000	2.510968000
B	4.358240000	1.060044000	-0.216381000	H	-3.476289000	-1.565348000	2.573614000
C	5.902716000	0.829840000	-0.379756000	H	-4.115933000	-3.336902000	4.120046000
C	6.721623000	1.949824000	-0.605810000	H	-5.576461000	-5.205853000	3.363501000
C	8.098834000	1.828401000	-0.762199000	H	-6.359344000	-5.252177000	1.001012000
C	8.697047000	0.574250000	-0.695404000	H	-5.726793000	-3.469260000	-0.552173000
C	7.910077000	-0.554071000	-0.472142000	H	-7.820264000	-0.339380000	-1.581745000
C	6.536291000	-0.424528000	-0.317776000	H	-7.347942000	-1.726993000	-0.587357000
C	-3.582223000	2.079416000	0.670559000	H	-6.588409000	-0.171869000	-0.313548000
C	-3.681608000	2.541413000	2.013903000	H	-7.313400000	-1.131826000	-3.729190000
C	-3.560118000	3.913615000	2.245420000	H	-3.307160000	-2.204235000	-4.796464000
C	-3.371171000	4.808248000	1.209652000	H	-2.155285000	-2.789646000	-1.492488000
C	-3.390961000	4.359907000	-0.098725000	H	-1.697221000	-2.619216000	-3.197465000
C	-3.526323000	3.005422000	-0.417203000	H	-1.623746000	-1.229176000	-2.112431000
C	-3.956340000	1.625981000	3.231378000	H	-3.300851000	5.094431000	-0.886414000
C	-3.728318000	2.642024000	-1.913713000	H	-3.604353000	4.299834000	3.254569000
C	3.534256000	-2.080219000	0.220519000	C	-2.694839000	1.650938000	-2.477100000
C	3.788451000	-2.658703000	1.506307000	H	-2.863814000	1.526275000	-3.551842000
C	3.945701000	-4.046726000	1.567132000	H	-1.670841000	2.007870000	-2.326670000
C	3.863498000	-4.841967000	0.441590000	H	-2.781188000	0.654348000	-2.047269000
C	3.637797000	-4.271320000	-0.795322000	C	-5.148962000	2.066471000	-2.051122000
C	3.471146000	-2.892665000	-0.958544000	H	-5.290680000	1.201170000	-1.406064000
C	3.929626000	-1.885647000	2.856156000	H	-5.895315000	2.821088000	-1.778610000
C	3.263519000	-2.391631000	-2.423857000	H	-5.334998000	1.741094000	-3.080455000
C	3.762584000	2.522585000	-0.298864000	C	-3.658160000	3.878942000	-2.830174000
C	3.350301000	3.050341000	-1.537375000	H	-2.669101000	4.351068000	-2.818201000
C	2.807766000	4.334409000	-1.593816000	H	-3.855275000	3.557839000	-3.857791000
C	2.675524000	5.099292000	-0.440799000	H	-4.412920000	4.630756000	-2.578553000
C	3.102441000	4.589432000	0.779650000	C	-5.187798000	0.742846000	2.945783000
C	3.648756000	3.308263000	0.863980000	H	-6.077014000	1.375142000	2.844808000
C	3.467771000	2.224578000	-2.795670000	H	-5.112245000	0.144531000	2.036913000
C	4.090495000	2.762737000	2.200887000	H	-5.354967000	0.043686000	3.772000000
H	6.266153000	2.935158000	-0.659624000	C	-2.703510000	0.811263000	3.612705000
H	8.704332000	2.713289000	-0.936097000	H	-2.326916000	0.175068000	2.811771000
H	9.771993000	0.474344000	-0.816768000	H	-1.888935000	1.488191000	3.893379000
H	8.369323000	-1.537048000	-0.418868000	H	-2.925812000	0.167423000	4.471441000
H	5.946221000	-1.319433000	-0.146179000	C	-4.323422000	2.434071000	4.493477000
H	2.485498000	4.736957000	-2.551037000	H	-3.483516000	3.022366000	4.877789000
H	3.010796000	5.191774000	1.680155000	H	-5.172669000	3.103445000	4.320598000
H	5.107938000	2.358004000	2.149576000	H	-4.611199000	1.732503000	5.283358000
H	4.073844000	3.538745000	2.971941000	C	5.144772000	-0.938723000	2.820767000
H	3.437426000	1.946257000	2.532152000	H	6.061408000	-1.492751000	2.590684000
H	3.236537000	2.818824000	-3.684695000	H	5.037536000	-0.145050000	2.084334000
H	4.479237000	1.818851000	-2.913519000	H	5.270748000	-0.466592000	3.802146000
H	2.779817000	1.370730000	-2.775282000	C	2.646932000	-1.115905000	3.234745000
H	1.005123000	-2.141473000	0.551672000	H	2.456829000	-0.255572000	2.595160000
H	-1.431589000	-1.892881000	0.629786000	H	1.769733000	-1.770618000	3.192226000
H	-1.013321000	2.353651000	0.116902000	H	2.739700000	-0.743318000	4.261146000
H	1.402944000	2.116031000	0.028544000	C	4.188273000	-2.843478000	4.040052000
H	3.589077000	-4.932870000	-1.646990000	H	5.117941000	-3.410579000	3.926218000

H	4.288100000	-2.245405000	4.951086000
H	3.361543000	-3.543832000	4.201031000
C	1.919871000	-1.660322000	-2.623900000
H	1.757422000	-1.492502000	-3.694532000
H	1.084158000	-2.260072000	-2.247363000
H	1.882624000	-0.688096000	-2.136261000
C	4.439465000	-1.498582000	-2.862889000
H	4.506146000	-0.579742000	-2.284513000
H	5.391078000	-2.031794000	-2.761532000
H	4.316183000	-1.220358000	-3.916097000
C	3.235692000	-3.562620000	-3.431076000
H	2.403412000	-4.251415000	-3.249922000
H	3.100683000	-3.148800000	-4.435096000
H	4.171878000	-4.130266000	-3.442705000
H	-5.649372000	-1.746002000	-5.458879000
H	2.248586000	6.096713000	-0.494883000
H	-3.245721000	5.866504000	1.419255000
H	3.980934000	-5.918336000	0.528410000

Compound **2^b(Xyl, Xyl*) (TICT-T$_1$ geometry):**

Total energy (ωB97X-D/6-31G(d)): -3138.77498367

C	-5.030471000	-3.498562000	1.036233000
C	-4.210524000	-2.351425000	1.174139000
C	-3.436079000	-2.286699000	2.355787000
C	-3.489458000	-3.271224000	3.332106000
C	-4.317281000	-4.381577000	3.169049000
C	-5.084374000	-4.487223000	2.008865000
B	-4.188724000	-1.312476000	0.028712000
C	-4.993624000	-1.496545000	-1.325222000
C	-6.367679000	-1.174882000	-1.368534000
C	-7.086536000	-1.324983000	-2.554708000
C	-6.463185000	-1.791320000	-3.706021000
C	-5.119374000	-2.141507000	-3.666014000
C	-4.383874000	-2.015117000	-2.485689000
C	-7.062090000	-0.637917000	-0.138803000
C	-2.945698000	-2.478799000	-2.457843000
P	-3.172136000	0.295892000	0.262269000
C	-1.390762000	0.222472000	0.171756000
C	-0.557913000	1.353178000	0.197790000
C	0.819030000	1.222458000	0.139022000
C	1.417319000	-0.046829000	0.060371000
C	0.583549000	-1.177120000	0.061875000
C	-0.792376000	-1.047519000	0.119985000
P	3.202974000	-0.278065000	-0.036953000
B	4.393079000	1.096385000	-0.089805000
C	5.945965000	0.884972000	-0.182816000
C	6.770560000	2.022798000	-0.224074000
C	8.155519000	1.918938000	-0.307400000
C	8.756175000	0.664946000	-0.351547000
C	7.963771000	-0.480848000	-0.312003000
C	6.582328000	-0.368729000	-0.229178000
C	-3.582898000	2.085772000	0.410707000
C	-3.711176000	2.659610000	1.710996000
C	-3.714088000	4.053443000	1.808807000
C	-3.603668000	4.859981000	0.692756000
C	-3.561191000	4.292944000	-0.567449000
C	-3.577206000	2.908499000	-0.758840000
C	-3.848742000	1.853081000	3.028962000
C	-3.725394000	2.402248000	-2.221489000
C	3.562655000	-2.072421000	-0.090944000
C	3.726433000	-2.790370000	1.138065000
C	3.885478000	-4.176986000	1.054678000
C	3.892663000	-4.841776000	-0.155427000
C	3.756663000	-4.136549000	-1.334693000
C	3.591410000	-2.748252000	-1.354148000
C	3.771839000	-2.173543000	2.572484000
C	3.482251000	-2.084374000	-2.763964000
C	3.795850000	2.559417000	-0.032623000
C	3.453265000	3.233142000	-1.220435000
C	2.911037000	4.516969000	-1.153278000
C	2.711149000	5.138854000	0.073532000
C	3.069049000	4.484758000	1.246562000
C	3.613238000	3.200618000	1.207623000
C	3.646348000	2.564605000	-2.560317000
C	3.978016000	2.495908000	2.492118000
H	6.313424000	3.008222000	-0.189873000
H	8.765184000	2.817435000	-0.337671000
H	9.837186000	0.578760000	-0.416489000
H	8.424845000	-1.463823000	-0.346008000
H	5.988235000	-1.276854000	-0.200302000
H	2.642928000	5.032768000	-2.072089000
H	2.924380000	4.975231000	2.206071000
H	4.999340000	2.099706000	2.453301000
H	3.910042000	3.171803000	3.349753000
H	3.311446000	1.645742000	2.681499000
H	3.473196000	3.264987000	-3.382821000
H	4.661837000	2.165121000	-2.664022000
H	2.955885000	1.721937000	-2.686578000
H	1.016067000	-2.171150000	0.017069000
H	-1.420048000	-1.934499000	0.130023000
H	-0.990984000	2.346864000	0.255755000
H	1.426947000	2.117617000	0.152391000
H	3.776677000	-4.698704000	-2.256165000
H	4.002624000	-4.769743000	1.949366000
H	-2.767110000	-1.444025000	2.508717000
H	-2.876484000	-3.176038000	4.225061000
H	-4.359396000	-5.154636000	3.930897000
H	-5.731035000	-5.348846000	1.863005000
H	-5.636033000	-3.604309000	0.139281000

H	-8.131196000	-0.488011000	-0.320264000
H	-6.949132000	-1.319075000	0.711631000
H	-6.635579000	0.325361000	0.170012000
H	-8.142894000	-1.067283000	-2.577762000
H	-4.636725000	-2.533790000	-4.558384000
H	-2.790442000	-3.197629000	-1.645280000
H	-2.668432000	-2.963378000	-3.399666000
H	-2.241631000	-1.654888000	-2.291344000
H	-3.518911000	4.958233000	-1.417668000
H	-3.791605000	4.530544000	2.775705000
C	-2.719144000	1.315036000	-2.640781000
H	-2.792758000	1.157147000	-3.722029000
H	-1.688003000	1.593567000	-2.402484000
H	-2.935173000	0.347433000	-2.187316000
C	-5.158545000	1.858632000	-2.365977000
H	-5.351660000	1.054745000	-1.656635000
H	-5.893250000	2.652855000	-2.193106000
H	-5.314935000	1.447374000	-3.369677000
C	-3.562228000	3.538903000	-3.250465000
H	-2.571219000	4.004435000	-3.200007000
H	-3.679697000	3.117674000	-4.253582000
H	-4.324669000	4.316573000	-3.144488000
C	-4.981895000	0.814633000	2.917967000
H	-5.938322000	1.322852000	2.750690000
H	-4.851420000	0.090201000	2.113666000
H	-5.055775000	0.240540000	3.847821000
C	-2.501591000	1.211861000	3.418745000
H	-2.104564000	0.534203000	2.663203000
H	-1.747425000	1.989780000	3.582926000
H	-2.616157000	0.641411000	4.347426000
C	-4.242103000	2.752148000	4.220011000
H	-3.455050000	3.462905000	4.492829000
H	-5.167801000	3.305646000	4.029894000
H	-4.412814000	2.114272000	5.093166000
C	4.989014000	-1.241374000	2.725695000
H	5.918377000	-1.776800000	2.502592000
H	4.936823000	-0.372435000	2.072975000
H	5.045739000	-0.878293000	3.758579000
C	2.469104000	-1.436088000	2.946965000
H	2.332069000	-0.502647000	2.403847000
H	1.592783000	-2.066959000	2.763062000
H	2.489048000	-1.190205000	4.014671000
C	3.944456000	-3.259857000	3.657055000
H	4.878499000	-3.820246000	3.546023000
H	3.981424000	-2.767790000	4.633837000
H	3.106534000	-3.964992000	3.680604000
C	2.146620000	-1.340589000	-2.972142000
H	2.058626000	-1.046842000	-4.024202000
H	1.294613000	-1.985264000	-2.730499000
H	2.062292000	-0.433647000	-2.376053000
C	4.674466000	-1.141520000	-3.015308000
H	4.687456000	-0.292351000	-2.335450000
H	5.623522000	-1.677515000	-2.905396000
H	4.622588000	-0.748330000	-4.037356000
C	3.539065000	-3.132928000	-3.896788000
H	2.705349000	-3.842138000	-3.853813000
H	3.468780000	-2.608359000	-4.854636000
H	4.481068000	-3.690919000	-3.906343000
H	-7.027687000	-1.896868000	-4.628498000
H	2.285160000	6.137271000	0.115039000
H	-3.578046000	5.940248000	0.802606000
H	4.010001000	-5.921364000	-0.180027000

Compound 1^{Xyl^*} ($S_0 \rightarrow$pyr-S_1 geometry, x = 0.0)

Total energy (ωB97X-D/6-31G(d)): -1685.47824371

P	-0.203659000	-0.387980000	-0.008816000
B	1.367872000	0.872418000	-0.066097000
C	1.102160000	2.356753000	0.139025000
C	-0.177756000	2.946579000	0.316793000
H	-1.064200000	2.320899000	0.326314000
C	-0.339423000	4.317522000	0.462532000
H	-1.336771000	4.726778000	0.602119000
C	0.762202000	5.169073000	0.418660000
H	0.633447000	6.241958000	0.527102000
C	2.034950000	4.626440000	0.208167000
H	2.900068000	5.281593000	0.148790000
C	2.198618000	3.261277000	0.062620000
H	3.191749000	2.862538000	-0.129429000
C	2.752361000	0.156789000	-0.180182000
C	3.639485000	0.200249000	0.932254000
C	4.844125000	-0.499577000	0.886341000
H	5.505207000	-0.472251000	1.749361000
C	5.203487000	-1.246763000	-0.230488000
C	4.349099000	-1.292077000	-1.323388000
H	4.625216000	-1.867762000	-2.203516000
C	3.137979000	-0.601986000	-1.313705000
C	2.258101000	-0.653015000	-2.531441000
H	2.728049000	-1.215640000	-3.343596000
H	2.031595000	0.354578000	-2.897224000
H	1.298405000	-1.136376000	-2.308468000
C	3.286764000	0.935646000	2.204141000
H	3.986632000	0.681052000	3.005918000
H	2.274808000	0.685935000	2.543011000
H	3.306834000	2.022356000	2.071158000
C	0.024845000	-2.135973000	0.145631000
C	1.270413000	-2.676393000	0.540904000
H	2.094923000	-2.021942000	0.796137000
C	1.440842000	-4.052131000	0.619536000
H	2.406098000	-4.446239000	0.923212000
C	0.393688000	-4.919093000	0.319864000

H	0.536265000	-5.993464000	0.382799000
C	-0.846060000	-4.395260000	-0.057850000
H	-1.671163000	-5.063004000	-0.287974000
C	-1.034141000	-3.028115000	-0.147138000
H	-2.001611000	-2.633285000	-0.442126000
C	-1.984957000	0.006387000	-0.097114000
C	-2.714711000	0.042599000	1.138298000
C	-4.098260000	0.228858000	1.055982000
H	-4.699632000	0.235883000	1.953282000
C	-4.745591000	0.425008000	-0.148108000
C	-4.021304000	0.456809000	-1.324443000
H	-4.558747000	0.638621000	-2.244152000
C	-2.641021000	0.249521000	-1.345564000
C	-2.134550000	-0.118889000	2.581209000
C	-0.843491000	0.683126000	2.832766000
H	0.003949000	0.351471000	2.224319000
H	-0.543666000	0.557524000	3.879227000
H	-0.981643000	1.750866000	2.638215000
C	-1.895751000	-1.607618000	2.905373000
H	-1.057064000	-2.027641000	2.350237000
H	-2.786955000	-2.203032000	2.677905000
H	-1.677063000	-1.718349000	3.974020000
C	-3.130642000	0.404258000	3.643163000
H	-3.451419000	1.430882000	3.436877000
H	-2.634233000	0.400419000	4.618373000
H	-4.016805000	-0.230200000	3.742934000
C	-1.945025000	0.272746000	-2.731544000
C	-0.792581000	1.289606000	-2.759414000
H	-0.354506000	1.327858000	-3.763173000
H	0.018396000	1.049970000	-2.069208000
H	-1.148686000	2.291431000	-2.496882000
C	-2.902254000	0.710087000	-3.859772000
H	-2.338631000	0.744055000	-4.797593000
H	-3.314504000	1.710132000	-3.687961000
H	-3.728440000	0.007013000	-4.008703000
C	-1.464904000	-1.141610000	-3.107881000
H	-0.954851000	-1.118299000	-4.077760000
H	-2.318223000	-1.824783000	-3.186676000
H	-0.770521000	-1.560957000	-2.379435000
H	6.143498000	-1.790913000	-0.245529000
H	-5.821632000	0.571298000	-0.168070000
X	-0.234647000	-0.303858000	0.987575000

Compound 1^{Xyl*} ($S_0 \rightarrow$pyr-S_1 geometry, x = 0.2)

Total energy (ωB97X-D/6-31G(d)): -1685.47869697

P	0.003453000	0.034006000	0.019550000
B	1.532488000	1.317000000	0.000599000
C	1.241347000	2.766215000	-0.386615000
C	-0.036400000	3.286763000	-0.721130000
H	-0.900554000	2.630031000	-0.737092000
C	-0.226753000	4.629095000	-1.018829000
H	-1.221086000	4.983942000	-1.278037000
C	0.841334000	5.522416000	-0.972708000
H	0.689724000	6.573972000	-1.197766000
C	2.107535000	5.051726000	-0.610108000
H	2.945318000	5.741463000	-0.550518000
C	2.300548000	3.712907000	-0.319802000
H	3.287930000	3.371032000	-0.018994000
C	2.934388000	0.654672000	0.202581000
C	3.310100000	-0.020605000	1.391305000
C	4.543766000	-0.663182000	1.481231000
H	4.810792000	-1.173155000	2.403665000
C	5.431636000	-0.652182000	0.414657000
C	5.079339000	0.004751000	-0.759607000
H	5.764002000	-0.002104000	-1.604466000
C	3.852351000	0.653437000	-0.886667000
C	3.506817000	1.271554000	-2.221583000
H	4.231215000	0.972282000	-2.985226000
H	3.491924000	2.365537000	-2.180562000
H	2.510356000	0.960649000	-2.556186000
C	2.391785000	-0.032182000	2.579737000
H	2.827409000	-0.577877000	3.421981000
H	1.434613000	-0.510275000	2.331553000
H	2.159845000	0.985903000	2.911577000
C	0.217924000	-1.702710000	-0.266120000
C	1.478800000	-2.223878000	-0.634531000
H	2.318978000	-1.558101000	-0.795929000
C	1.648590000	-3.592670000	-0.794910000
H	2.625281000	-3.973609000	-1.078405000
C	0.586031000	-4.470465000	-0.597630000
H	0.727714000	-5.539521000	-0.722568000
C	-0.665629000	-3.964946000	-0.236674000
H	-1.501009000	-4.641592000	-0.081552000
C	-0.854204000	-2.603504000	-0.070278000
H	-1.830232000	-2.224584000	0.217417000
C	-1.778078000	0.394012000	0.067575000
C	-2.474241000	0.629147000	1.296673000
C	-3.863939000	0.710221000	1.251550000
H	-4.428626000	0.881461000	2.157420000
C	-4.566578000	0.551055000	0.067621000
C	-3.883343000	0.372025000	-1.115520000
H	-4.459598000	0.274826000	-2.027935000
C	-2.483079000	0.329910000	-1.171091000
C	-1.792636000	0.755864000	2.679561000
C	-0.683657000	1.821041000	2.652987000
H	0.137148000	1.586743000	1.971562000
H	-0.245957000	1.929228000	3.651765000
H	-1.087555000	2.791202000	2.344046000
C	-1.267307000	-0.623467000	3.124296000
H	-0.597911000	-1.076300000	2.390248000

H	-2.104744000	-1.313317000	3.278213000
H	-0.718529000	-0.533632000	4.068908000
C	-2.774544000	1.217044000	3.776013000
H	-3.235677000	2.181195000	3.535975000
H	-2.218753000	1.340058000	4.711188000
H	-3.565862000	0.485581000	3.969688000
C	-1.928927000	0.246749000	-2.630463000
C	-0.409965000	0.343683000	-2.874783000
H	-0.256670000	0.412530000	-3.959029000
H	0.139184000	-0.539576000	-2.544302000
H	0.039251000	1.241246000	-2.440914000
C	-2.524584000	1.436540000	-3.423946000
H	-2.192569000	1.385653000	-4.466515000
H	-2.172056000	2.386109000	-3.005319000
H	-3.616507000	1.455124000	-3.430612000
C	-2.386289000	-1.091680000	-3.247925000
H	-2.040465000	-1.160467000	-4.285630000
H	-3.474663000	-1.203135000	-3.252384000
H	-1.960490000	-1.933938000	-2.691447000
H	6.390366000	-1.156805000	0.493045000
H	-5.652086000	0.585100000	0.070794000
X	0.045909000	-0.063245000	1.013908000

Compound 1^{Xyl*} (S_0→pyr-S_1 geometry, x = 0.4)

Total energy (ωB97X-D/6-31G(d)): -1685.47886740

P	-0.164118000	-0.411655000	0.215387000
B	1.357840000	0.887596000	0.106905000
C	1.061456000	2.326023000	-0.316297000
C	-0.216505000	2.844631000	-0.652271000
H	-1.081320000	2.189120000	-0.660002000
C	-0.406868000	4.183729000	-0.963889000
H	-1.402193000	4.535786000	-1.223210000
C	0.661775000	5.077150000	-0.933464000
H	0.510195000	6.125971000	-1.170751000
C	1.928899000	4.608526000	-0.572747000
H	2.768425000	5.297202000	-0.526236000
C	2.122257000	3.272612000	-0.268739000
H	3.111250000	2.934148000	0.030568000
C	2.760831000	0.228219000	0.309017000
C	3.136667000	-0.416189000	1.515706000
C	4.369904000	-1.057655000	1.621669000
H	4.637347000	-1.543950000	2.556667000
C	5.256922000	-1.073597000	0.554770000
C	4.905788000	-0.445216000	-0.635756000
H	5.591741000	-0.472534000	-1.479094000
C	3.679171000	0.200225000	-0.779460000
C	3.334305000	0.785716000	-2.129137000
H	4.078926000	0.496837000	-2.877147000
H	3.284760000	1.878940000	-2.106319000
H	2.353843000	0.437308000	-2.473591000
C	2.219353000	-0.394861000	2.704624000
H	2.657746000	-0.911877000	3.563285000
H	1.262395000	-0.884202000	2.471599000
H	1.981600000	0.632062000	3.003527000
C	0.034856000	-2.144584000	-0.128876000
C	1.294389000	-2.670215000	-0.491373000
H	2.140316000	-2.008158000	-0.638329000
C	1.455695000	-4.038307000	-0.667937000
H	2.431351000	-4.423249000	-0.949562000
C	0.385000000	-4.910036000	-0.489585000
H	0.519478000	-5.978484000	-0.627167000
C	-0.865166000	-4.399931000	-0.130794000
H	-1.706657000	-5.072174000	0.009695000
C	-1.044581000	-3.038743000	0.050174000
H	-2.020094000	-2.655782000	0.334469000
C	-1.942179000	-0.030175000	0.170099000
C	-2.668139000	0.234869000	1.375477000
C	-4.057211000	0.304249000	1.296391000
H	-4.644350000	0.494732000	2.183940000
C	-4.730343000	0.110349000	0.100661000
C	-4.017958000	-0.089815000	-1.062007000
H	-4.570909000	-0.208751000	-1.986376000
C	-2.616841000	-0.125993000	-1.082890000
C	-2.018897000	0.409309000	2.769687000
C	-0.898224000	1.462569000	2.731026000
H	-0.062494000	1.197015000	2.079377000
H	-0.484576000	1.603095000	3.736041000
H	-1.284552000	2.424565000	2.377236000
C	-1.518150000	-0.955407000	3.283605000
H	-0.827190000	-1.439556000	2.590041000
H	-2.364841000	-1.634161000	3.436399000
H	-1.000183000	-0.831888000	4.241787000
C	-3.024467000	0.923296000	3.820558000
H	-3.475516000	1.876813000	3.525293000
H	-2.490710000	1.087109000	4.762265000
H	-3.823506000	0.204362000	4.028796000
C	-2.025650000	-0.226745000	-2.524991000
C	-0.500673000	-0.136662000	-2.721684000
H	-0.310432000	-0.066921000	-3.799693000
H	0.032098000	-1.022352000	-2.373149000
H	-0.062840000	0.758716000	-2.272477000
C	-2.595200000	0.958840000	-3.343500000
H	-2.238835000	0.895475000	-4.377380000
H	-2.246999000	1.910592000	-2.926399000
H	-3.686711000	0.982809000	-3.375516000
C	-2.470282000	-1.567215000	-3.146850000
H	-2.093011000	-1.643711000	-4.172970000
H	-3.558511000	-1.673861000	-3.184807000
H	-2.065618000	-2.407865000	-2.572792000
H	6.215653000	-1.576179000	0.645693000

H	-5.815853000	0.136104000	0.077448000
X	-0.133737000	-0.483003000	1.212375000

Compound 1^{Xyl*} ($S_0 \rightarrow$pyr-S_1 geometry, x = 0.6)

Total energy (ωB97X-D/6-31G(d)): -1685.47935785

P	-0.167265000	-0.429317000	0.309771000
B	1.358544000	0.874682000	0.115084000
C	1.066866000	2.295871000	-0.364484000
C	-0.210031000	2.817603000	-0.699489000
H	-1.080986000	2.171313000	-0.674292000
C	-0.391402000	4.147895000	-1.051099000
H	-1.387095000	4.501275000	-1.307339000
C	0.686036000	5.031194000	-1.065271000
H	0.541649000	6.073128000	-1.334956000
C	1.953223000	4.560433000	-0.708360000
H	2.800858000	5.240650000	-0.697158000
C	2.138083000	3.232822000	-0.364970000
H	3.128529000	2.894587000	-0.070401000
C	2.760656000	0.218081000	0.340928000
C	3.135614000	-0.353415000	1.584094000
C	4.371041000	-0.983077000	1.730181000
H	4.638637000	-1.412362000	2.692659000
C	5.260656000	-1.057057000	0.668045000
C	4.911139000	-0.499311000	-0.557550000
H	5.600851000	-0.570338000	-1.395218000
C	3.682052000	0.131285000	-0.741151000
C	3.337283000	0.636675000	-2.122715000
H	4.113616000	0.356880000	-2.841378000
H	3.226274000	1.724912000	-2.151186000
H	2.387193000	0.215918000	-2.471489000
C	2.215710000	-0.266216000	2.768463000
H	2.665690000	-0.707474000	3.662736000
H	1.270650000	-0.795119000	2.574524000
H	1.952904000	0.773894000	2.990286000
C	0.011519000	-2.156941000	-0.100978000
C	1.271344000	-2.690260000	-0.445869000
H	2.128897000	-2.035712000	-0.555576000
C	1.419404000	-4.055513000	-0.654125000
H	2.395411000	-4.446822000	-0.925405000
C	0.333780000	-4.916520000	-0.518873000
H	0.457181000	-5.982958000	-0.680533000
C	-0.915880000	-4.399580000	-0.170166000
H	-1.768142000	-5.064062000	-0.061425000
C	-1.081075000	-3.040031000	0.039091000
H	-2.057204000	-2.651099000	0.312979000
C	-1.938727000	-0.023184000	0.174699000
C	-2.689274000	0.271049000	1.358382000
C	-4.076046000	0.347420000	1.250039000
H	-4.679823000	0.558458000	2.121611000
C	-4.724644000	0.135989000	0.044088000
C	-3.988432000	-0.088434000	-1.099287000
H	-4.522553000	-0.216887000	-2.033393000
C	-2.587574000	-0.136704000	-1.090414000
C	-2.065679000	0.477573000	2.760653000
C	-0.952487000	1.539579000	2.716637000
H	-0.107467000	1.273910000	2.076725000
H	-0.550522000	1.698777000	3.723671000
H	-1.344804000	2.492611000	2.345551000
C	-1.562681000	-0.870735000	3.314162000
H	-0.843978000	-1.353158000	2.646966000
H	-2.403762000	-1.559070000	3.454538000
H	-1.073943000	-0.722670000	4.284177000
C	-3.092875000	1.005809000	3.782931000
H	-3.544248000	1.950625000	3.461421000
H	-2.576982000	1.191492000	4.730529000
H	-3.891175000	0.285789000	3.990535000
C	-1.969109000	-0.259474000	-2.518686000
C	-0.440317000	-0.192308000	-2.680002000
H	-0.222023000	-0.134152000	-3.753122000
H	0.070100000	-1.081788000	-2.310418000
H	-0.001921000	0.701030000	-2.228643000
C	-2.504822000	0.927740000	-3.357712000
H	-2.129000000	0.849818000	-4.383727000
H	-2.150616000	1.877856000	-2.942361000
H	-3.595201000	0.966600000	-3.410937000
C	-2.419640000	-1.596873000	-3.143197000
H	-2.021605000	-1.682577000	-4.160685000
H	-3.508110000	-1.689023000	-3.204766000
H	-2.038753000	-2.440762000	-2.558032000
H	6.221486000	-1.548863000	0.790611000
H	-5.809198000	0.168297000	-0.003387000
X	-0.147314000	-0.478357000	1.308369000

Compound 1^{Xyl*} ($S_0 \rightarrow$pyr-S_1 geometry, x = 0.8)

Total energy (ωB97X-D/6-31G(d)): -1685.47865255

P	-0.167100000	-0.445963000	0.406572000
B	1.359170000	0.864193000	0.127884000
C	1.064068000	2.266847000	-0.400622000
C	-0.213041000	2.783927000	-0.740546000
H	-1.085240000	2.141477000	-0.689194000
C	-0.392757000	4.103536000	-1.130905000
H	-1.389753000	4.452099000	-1.388887000
C	0.687425000	4.982089000	-1.183130000
H	0.544716000	6.015497000	-1.484431000
C	1.955734000	4.516327000	-0.824538000
H	2.806776000	5.192180000	-0.843508000
C	2.139188000	3.199050000	-0.442335000
H	3.131640000	2.866683000	-0.148028000
C	2.761327000	0.213641000	0.371741000
C	3.133756000	-0.301106000	1.640613000

C	4.369719000	-0.921807000	1.818445000
H	4.635159000	-1.308071000	2.799562000
C	5.263126000	-1.038979000	0.763473000
C	4.917307000	-0.535483000	-0.486635000
H	5.611159000	-0.639871000	-1.317353000
C	3.686760000	0.081870000	-0.702012000
C	3.342451000	0.522931000	-2.105223000
H	4.139333000	0.249370000	-2.803516000
H	3.186514000	1.603544000	-2.175084000
H	2.415961000	0.048870000	-2.449321000
C	2.209322000	-0.161207000	2.816167000
H	2.646305000	-0.581268000	3.726912000
H	1.255169000	-0.679185000	2.631545000
H	1.961923000	0.889727000	3.002285000
C	0.001076000	-2.164808000	-0.071913000
C	1.263934000	-2.701348000	-0.395245000
H	2.130400000	-2.052809000	-0.461195000
C	1.404503000	-4.060987000	-0.642791000
H	2.383124000	-4.454642000	-0.900664000
C	0.306796000	-4.913415000	-0.562108000
H	0.423545000	-5.975638000	-0.753913000
C	-0.945401000	-4.394952000	-0.227207000
H	-1.806281000	-5.053563000	-0.158355000
C	-1.101673000	-3.039767000	0.018182000
H	-2.080558000	-2.649444000	0.279668000
C	-1.930694000	-0.025824000	0.181853000
C	-2.707996000	0.298359000	1.341002000
C	-4.092328000	0.371951000	1.201689000
H	-4.714497000	0.601847000	2.055258000
C	-4.714266000	0.137142000	-0.013441000
C	-3.952328000	-0.108529000	-1.135404000
H	-4.465424000	-0.247273000	-2.079757000
C	-2.552349000	-0.163583000	-1.094995000
C	-2.116480000	0.547473000	2.751827000
C	-0.998157000	1.604813000	2.701950000
H	-0.138017000	1.323349000	2.089943000
H	-0.619378000	1.791579000	3.713241000
H	-1.380489000	2.547771000	2.296365000
C	-1.635997000	-0.782074000	3.368215000
H	-0.893003000	-1.288881000	2.746807000
H	-2.483043000	-1.464552000	3.501200000
H	-1.183703000	-0.600591000	4.350205000
C	-3.166541000	1.115949000	3.729055000
H	-3.608345000	2.048056000	3.360748000
H	-2.672374000	1.337645000	4.680463000
H	-3.970912000	0.405589000	3.946180000
C	-1.907881000	-0.300355000	-2.510251000
C	-0.375799000	-0.285132000	-2.635595000
H	-0.126971000	-0.178101000	-3.697895000
H	0.087905000	-1.211868000	-2.298828000
H	0.087644000	0.562914000	-2.125890000
C	-2.390509000	0.909282000	-3.349674000
H	-2.017917000	0.814354000	-4.375553000
H	-1.995508000	1.843241000	-2.935091000
H	-3.478607000	0.993593000	-3.400958000
C	-2.384799000	-1.616953000	-3.159721000
H	-1.950232000	-1.710517000	-4.161380000
H	-3.472217000	-1.670071000	-3.266524000
H	-2.057185000	-2.477786000	-2.567261000
H	6.224962000	-1.522036000	0.910717000
H	-5.797381000	0.170414000	-0.086266000
X	-0.156924000	-0.473116000	1.406155000

Compound 1^{Xyl^*} ($S_0 \rightarrow$TICT-S_1 geometry, 180°)

Total energy (ωB97X-D/6-31G(d)): -1685.48147016

P	-0.199761000	-0.437736000	-0.009207000
B	1.364897000	0.854052000	0.080526000
C	1.079239000	2.341119000	0.108583000
C	-0.200910000	2.952912000	-0.003020000
H	-1.085480000	2.336650000	-0.124371000
C	-0.360444000	4.330121000	0.041679000
H	-1.357130000	4.755199000	-0.047563000
C	0.741491000	5.169149000	0.198323000
H	0.612973000	6.246838000	0.234368000
C	2.016602000	4.602408000	0.316789000
H	2.884034000	5.244063000	0.448031000
C	2.180901000	3.230992000	0.278961000
H	3.177446000	2.811106000	0.393327000
C	2.765361000	0.155900000	0.112831000
C	3.191637000	-0.585631000	1.241256000
C	4.427267000	-1.232140000	1.229333000
H	4.741025000	-1.799342000	2.102327000
C	5.258190000	-1.152231000	0.119611000
C	4.859337000	-0.409011000	-0.986555000
H	5.509710000	-0.346265000	-1.855709000
C	3.629780000	0.247916000	-1.006109000
C	3.224811000	1.013547000	-2.243338000
H	4.002994000	0.957114000	-3.010758000
H	3.039081000	2.069970000	-2.023257000
H	2.299480000	0.611859000	-2.672384000
C	2.324353000	-0.665813000	2.470010000
H	2.829639000	-1.195037000	3.283455000
H	1.383917000	-1.196376000	2.267543000
H	2.056281000	0.336121000	2.824643000
C	-0.014834000	-2.196465000	-0.047919000
C	1.248001000	-2.794405000	-0.274486000
H	2.122940000	-2.178368000	-0.439310000
C	1.371927000	-4.176394000	-0.306844000
H	2.351956000	-4.612533000	-0.476397000
C	0.260294000	-4.997448000	-0.136862000

H	0.365793000	-6.077494000	-0.165680000
C	-0.996672000	-4.417705000	0.058540000
H	-1.872729000	-5.048048000	0.180836000
C	-1.140176000	-3.043131000	0.101419000
H	-2.122484000	-2.608033000	0.255996000
C	-1.974066000	-0.006551000	-0.061654000
C	-2.715818000	0.156854000	1.150960000
C	-4.096310000	0.343117000	1.039143000
H	-4.704301000	0.457885000	1.924931000
C	-4.731149000	0.384824000	-0.187256000
C	-3.996362000	0.278038000	-1.352301000
H	-4.528517000	0.344290000	-2.290138000
C	-2.610635000	0.095997000	-1.339741000
C	-2.124681000	0.129326000	2.590158000
C	-0.943912000	1.101408000	2.745494000
H	-0.086699000	0.847670000	2.118497000
H	-0.592019000	1.087094000	3.783300000
H	-1.233881000	2.125717000	2.490316000
C	-1.702069000	-1.299450000	2.980890000
H	-0.880488000	-1.675721000	2.371198000
H	-2.543309000	-1.994795000	2.882590000
H	-1.370814000	-1.311929000	4.025643000
C	-3.161398000	0.573129000	3.644387000
H	-3.557800000	1.573116000	3.438695000
H	-2.668383000	0.609348000	4.620875000
H	-3.997526000	-0.127953000	3.736491000
C	-1.900280000	0.026949000	-2.725346000
C	-0.707621000	0.996192000	-2.817403000
H	-0.281732000	0.955053000	-3.826427000
H	0.104306000	0.757622000	-2.123947000
H	-1.010612000	2.027888000	-2.611478000
C	-2.850247000	0.446411000	-3.868971000
H	-2.279583000	0.471044000	-4.802461000
H	-3.270447000	1.445692000	-3.713284000
H	-3.669865000	-0.264256000	-4.017653000
C	-1.449618000	-1.411585000	-3.051120000
H	-1.122794000	-1.463504000	-4.096157000
H	-2.276130000	-2.118246000	-2.917994000
H	-0.618420000	-1.745428000	-2.430112000
H	6.217382000	-1.662258000	0.116868000
H	-5.807796000	0.519922000	-0.236030000

Compound 1^{Xyl*} ($S_0 \rightarrow$TICT-S_1 geometry, 170°)

Total energy (ωB97X-D/6-31G(d)): -1685.47902777

P	-0.197596000	-0.410042000	-0.027676000
B	1.346253000	0.889936000	0.035372000
C	1.033376000	2.375652000	-0.060650000
C	-0.262379000	2.939495000	-0.207910000
H	-1.130518000	2.291210000	-0.265987000
C	-0.462728000	4.311731000	-0.262395000
H	-1.471074000	4.700740000	-0.379151000
C	0.614041000	5.189815000	-0.158499000
H	0.454622000	6.263395000	-0.196235000
C	1.901638000	4.670788000	0.017763000
H	2.747895000	5.344786000	0.121527000
C	2.104048000	3.304245000	0.074375000
H	3.108218000	2.922417000	0.241573000
C	2.752731000	0.223488000	0.180707000
C	3.145615000	-0.533633000	1.312346000
C	4.386333000	-1.169178000	1.338970000
H	4.668469000	-1.744970000	2.217157000
C	5.262614000	-1.069263000	0.267266000
C	4.896737000	-0.319744000	-0.845882000
H	5.576164000	-0.246996000	-1.691854000
C	3.663043000	0.325560000	-0.908301000
C	3.305369000	1.074381000	-2.171072000
H	4.044915000	0.888349000	-2.955964000
H	3.253216000	2.155957000	-2.006888000
H	2.322483000	0.771327000	-2.550741000
C	2.245942000	-0.639854000	2.512080000
H	2.727673000	-1.188554000	3.326879000
H	1.314061000	-1.165428000	2.268310000
H	1.967248000	0.352033000	2.884262000
C	0.076941000	-2.150058000	-0.186407000
C	1.345628000	-2.655310000	-0.555150000
H	2.161532000	-1.978045000	-0.775887000
C	1.550203000	-4.025245000	-0.651059000
H	2.532564000	-4.392208000	-0.933121000
C	0.515512000	-4.920771000	-0.395240000
H	0.684874000	-5.990409000	-0.471061000
C	-0.746443000	-4.431753000	-0.044961000
H	-1.561832000	-5.122070000	0.150571000
C	-0.968923000	-3.071133000	0.061021000
H	-1.953085000	-2.703548000	0.335121000
C	-1.990107000	-0.063451000	0.059509000
C	-2.664461000	0.121094000	1.307966000
C	-4.050231000	0.288315000	1.278560000
H	-4.602052000	0.423661000	2.197730000
C	-4.761662000	0.275802000	0.094144000
C	-4.097233000	0.139084000	-1.108985000
H	-4.689572000	0.160474000	-2.012007000
C	-2.708561000	-0.007472000	-1.181793000
C	-1.982647000	0.125816000	2.701295000
C	-0.859325000	1.172794000	2.765417000
H	-0.041608000	0.976318000	2.070242000
H	-0.424992000	1.191424000	3.771362000
H	-1.241952000	2.172208000	2.532904000
C	-1.464967000	-1.283343000	3.045784000
H	-0.746582000	-1.660719000	2.317819000
H	-2.297673000	-1.994391000	3.091855000

H	-0.971240000	-1.271477000	4.024276000
C	-2.961935000	0.506762000	3.830893000
H	-3.403039000	1.497703000	3.679196000
H	-2.407269000	0.535270000	4.774224000
H	-3.767554000	-0.224342000	3.955768000
C	-2.104897000	-0.103917000	-2.620306000
C	-0.862361000	0.782501000	-2.830309000
H	-0.535824000	0.700486000	-3.873092000
H	-0.006677000	0.493348000	-2.211373000
H	-1.072920000	1.834635000	-2.615438000
C	-3.116288000	0.382198000	-3.684709000
H	-2.610750000	0.416549000	-4.654641000
H	-3.490504000	1.388687000	-3.469471000
H	-3.968079000	-0.295431000	-3.800230000
C	-1.773758000	-1.567233000	-2.977500000
H	-1.540141000	-1.638135000	-4.046331000
H	-2.629277000	-2.220516000	-2.773165000
H	-0.915963000	-1.951201000	-2.425904000
H	6.225305000	-1.571544000	0.296802000
H	-5.841581000	0.390987000	0.107443000

Compound 1^{Xyl*} ($S_0 \rightarrow$ TICT-S_1 geometry, 160°)

Total energy (ωB97X-D/6-31G(d)): -1685.47645210

P	-0.206644000	-0.373663000	-0.029459000
B	1.416688000	0.787418000	0.011328000
C	1.185268000	2.286157000	-0.183623000
C	-0.074517000	2.888716000	-0.438881000
H	-0.965562000	2.271566000	-0.504232000
C	-0.214236000	4.261368000	-0.589929000
H	-1.196680000	4.681177000	-0.790355000
C	0.890865000	5.101073000	-0.469540000
H	0.779370000	6.175954000	-0.577712000
C	2.141977000	4.545304000	-0.182981000
H	3.007789000	5.191654000	-0.066048000
C	2.283542000	3.176743000	-0.037578000
H	3.259853000	2.765611000	0.207166000
C	2.790226000	0.074314000	0.238348000
C	3.138689000	-0.680343000	1.385057000
C	4.363178000	-1.343453000	1.451268000
H	4.607858000	-1.913731000	2.344219000
C	5.270028000	-1.278757000	0.402834000
C	4.943153000	-0.547018000	-0.733427000
H	5.639091000	-0.510781000	-1.568350000
C	3.725758000	0.123112000	-0.836531000
C	3.406523000	0.827476000	-2.135546000
H	4.133390000	0.561038000	-2.909091000
H	3.412798000	1.917482000	-2.029698000
H	2.407284000	0.557372000	-2.499098000
C	2.207788000	-0.754827000	2.560168000
H	2.602205000	-1.403503000	3.348003000
H	1.230897000	-1.150327000	2.259019000
H	2.031442000	0.237523000	2.989129000
C	-0.062412000	-2.109537000	-0.344480000
C	1.168021000	-2.653784000	-0.778025000
H	2.028896000	-2.010671000	-0.922043000
C	1.282903000	-4.016806000	-1.016852000
H	2.236889000	-4.416516000	-1.347615000
C	0.194923000	-4.865930000	-0.833273000
H	0.294088000	-5.931152000	-1.017889000
C	-1.026313000	-4.337810000	-0.406148000
H	-1.880093000	-4.992879000	-0.259103000
C	-1.160034000	-2.981676000	-0.161957000
H	-2.112113000	-2.584567000	0.176787000
C	-1.966208000	0.027453000	0.214266000
C	-2.579884000	0.206757000	1.495464000
C	-3.962911000	0.361182000	1.540217000
H	-4.463075000	0.495009000	2.489431000
C	-4.740355000	0.322528000	0.393661000
C	-4.135670000	0.193781000	-0.837558000
H	-4.768070000	0.188031000	-1.717249000
C	-2.744715000	0.090013000	-0.981993000
C	-1.816565000	0.196002000	2.838089000
C	-0.670077000	1.219680000	2.819799000
H	0.086326000	1.018102000	2.059429000
H	-0.156833000	1.226311000	3.787842000
H	-1.056887000	2.225739000	2.624967000
C	-1.330234000	-1.235913000	3.135477000
H	-0.751758000	-1.665338000	2.315922000
H	-2.189512000	-1.893415000	3.309320000
H	-0.702301000	-1.247212000	4.033727000
C	-2.708453000	0.603884000	4.027988000
H	-3.134547000	1.604710000	3.899040000
H	-2.092857000	0.621075000	4.933283000
H	-3.522307000	-0.105839000	4.208888000
C	-2.286740000	0.079480000	-2.479500000
C	-0.784329000	0.126286000	-2.830161000
H	-0.710305000	0.243875000	-3.918964000
H	-0.252229000	-0.795375000	-2.586858000
H	-0.260817000	0.980003000	-2.390104000
C	-2.877356000	1.340903000	-3.158132000
H	-2.611765000	1.345419000	-4.220732000
H	-2.459817000	2.246635000	-2.703706000
H	-3.965655000	1.403762000	-3.095231000
C	-2.845014000	-1.197751000	-3.142673000
H	-2.574825000	-1.215662000	-4.204713000
H	-3.934859000	-1.264884000	-3.075807000
H	-2.420187000	-2.089936000	-2.669215000
H	6.220645000	-1.800555000	0.464969000
H	-5.820448000	0.410513000	0.464851000

Compound 1^{Xyl*} (S_0→TICT-S_1 geometry, 140°)

Total energy (ωB97X-D/6-31G(d)): -1685.46365549

P	-0.273539000	-0.328953000	-0.069584000
B	1.494953000	0.594771000	-0.038035000
C	1.456139000	2.102911000	-0.322616000
C	0.268180000	2.875428000	-0.353913000
H	-0.684902000	2.402852000	-0.131313000
C	0.286100000	4.237802000	-0.623072000
H	-0.647293000	4.794540000	-0.641676000
C	1.494342000	4.894633000	-0.840938000
H	1.510444000	5.962178000	-1.040446000
C	2.689679000	4.172940000	-0.768879000
H	3.638914000	4.681272000	-0.914797000
C	2.670814000	2.813735000	-0.507497000
H	3.610901000	2.272052000	-0.440300000
C	2.826070000	-0.199517000	0.262206000
C	3.339641000	-0.555056000	1.526388000
C	4.533196000	-1.270278000	1.625366000
H	4.909407000	-1.535913000	2.610498000
C	5.252883000	-1.629279000	0.493849000
C	4.787052000	-1.243840000	-0.755286000
H	5.355123000	-1.497367000	-1.647394000
C	3.595873000	-0.529151000	-0.886962000
C	3.172225000	-0.096185000	-2.274401000
H	3.682698000	-0.685855000	-3.042510000
H	3.409461000	0.959642000	-2.449620000
H	2.091530000	-0.196422000	-2.430674000
C	2.645397000	-0.130178000	2.790882000
H	3.283052000	-0.289806000	3.666006000
H	1.721309000	-0.690603000	2.949561000
H	2.381589000	0.931812000	2.751149000
C	-0.389212000	-1.949274000	-0.809245000
C	0.758393000	-2.569195000	-1.342757000
H	1.724251000	-2.086477000	-1.263505000
C	0.675002000	-3.824645000	-1.931672000
H	1.573599000	-4.279502000	-2.337913000
C	-0.538591000	-4.503182000	-1.985618000
H	-0.598266000	-5.486350000	-2.442258000
C	-1.675265000	-3.916846000	-1.426712000
H	-2.624315000	-4.444927000	-1.444358000
C	-1.607033000	-2.662480000	-0.841487000
H	-2.500136000	-2.227939000	-0.405561000
C	-1.961805000	0.075668000	0.508977000
C	-2.389630000	-0.015124000	1.871227000
C	-3.735809000	0.192620000	2.156479000
H	-4.095576000	0.111395000	3.172873000
C	-4.652750000	0.494431000	1.162809000
C	-4.222823000	0.634202000	-0.138615000
H	-4.959174000	0.900574000	-0.887167000
C	-2.884070000	0.455167000	-0.518284000
C	-1.435337000	-0.279538000	3.053225000
C	-0.475448000	0.920137000	3.156532000
H	0.126970000	1.065272000	2.255005000
H	0.211441000	0.796394000	4.000378000
H	-1.050775000	1.838779000	3.315004000
C	-0.710560000	-1.630547000	2.903214000
H	-0.061065000	-1.682720000	2.026166000
H	-1.442141000	-2.441624000	2.818018000
H	-0.090302000	-1.824404000	3.785717000
C	-2.171086000	-0.353763000	4.405644000
H	-2.686461000	0.580084000	4.652527000
H	-1.433240000	-0.533635000	5.194343000
H	-2.894382000	-1.175868000	4.439559000
C	-2.658906000	0.741689000	-2.047244000
C	-1.259186000	0.606900000	-2.683852000
H	-1.344871000	0.953578000	-3.721661000
H	-0.907931000	-0.425091000	-2.728042000
H	-0.497292000	1.229795000	-2.210617000
C	-3.074312000	2.211281000	-2.303089000
H	-3.015874000	2.430708000	-3.374905000
H	-2.393278000	2.895534000	-1.785235000
H	-4.091885000	2.435850000	-1.974601000
C	-3.562621000	-0.213159000	-2.861715000
H	-3.442439000	-0.008723000	-3.931553000
H	-4.625180000	-0.111180000	-2.626978000
H	-3.273185000	-1.254671000	-2.684841000
H	6.178701000	-2.189681000	0.586679000
H	-5.700350000	0.637038000	1.410729000

Compound 1^{Xyl*} (S_0→TICT-S_1 geometry, 120°)

Total energy (ωB97X-D/6-31G(d)): -1685.44756999

P	0.262196000	-0.273249000	0.325035000
B	-1.439226000	0.662903000	0.008160000
C	-1.488727000	2.205398000	0.086976000
C	-0.385852000	3.028521000	-0.219807000
H	0.554140000	2.565398000	-0.504815000
C	-0.487646000	4.415157000	-0.228799000
H	0.379675000	5.019675000	-0.481732000
C	-1.701954000	5.029484000	0.062087000
H	-1.783988000	6.112592000	0.053227000
C	-2.821425000	4.241786000	0.338316000
H	-3.777690000	4.712911000	0.548697000
C	-2.717881000	2.859690000	0.337579000
H	-3.603564000	2.261400000	0.533078000
C	-2.740685000	-0.170815000	-0.324094000
C	-3.071075000	-0.448381000	-1.668931000
C	-4.173038000	-1.245400000	-1.969514000
H	-4.405030000	-1.471204000	-3.007897000
C	-4.991411000	-1.731077000	-0.954580000
C	-4.731096000	-1.379272000	0.362082000

H	-5.397675000	-1.713929000	1.154193000
C	-3.625070000	-0.589737000	0.690619000
C	-3.465631000	-0.139387000	2.127403000
H	-3.656156000	-0.955527000	2.833567000
H	-4.183573000	0.658188000	2.354216000
H	-2.467888000	0.261912000	2.329867000
C	-2.303342000	0.216662000	-2.788118000
H	-2.389781000	-0.339167000	-3.727478000
H	-1.240051000	0.335492000	-2.555756000
H	-2.700685000	1.225083000	-2.957268000
C	0.435273000	-1.461256000	1.651229000
C	-0.705628000	-2.080230000	2.187302000
H	-1.680099000	-1.861107000	1.769801000
C	-0.586709000	-3.019634000	3.205971000
H	-1.481604000	-3.485920000	3.607465000
C	0.666439000	-3.381451000	3.688740000
H	0.756679000	-4.121750000	4.477711000
C	1.810989000	-2.805719000	3.133271000
H	2.794868000	-3.098016000	3.488200000
C	1.699894000	-1.859350000	2.127989000
H	2.594653000	-1.432956000	1.684551000
C	1.822314000	-0.088842000	-0.619838000
C	2.007400000	-0.849733000	-1.827643000
C	2.929596000	-0.369995000	-2.757509000
H	3.061782000	-0.869355000	-3.707036000
C	3.730710000	0.727623000	-2.490877000
C	3.663308000	1.338507000	-1.255953000
H	4.372844000	2.128259000	-1.043336000
C	2.716880000	0.976632000	-0.288315000
C	1.383277000	-2.251788000	-2.107848000
C	-0.150372000	-2.283973000	-2.194676000
H	-0.646914000	-1.912946000	-1.294277000
H	-0.484695000	-3.318113000	-2.336927000
H	-0.502786000	-1.697668000	-3.046813000
C	1.860566000	-3.225546000	-1.010574000
H	1.414202000	-3.008454000	-0.041398000
H	2.950373000	-3.187126000	-0.903861000
H	1.580119000	-4.250661000	-1.279377000
C	1.878387000	-2.840767000	-3.445381000
H	1.583396000	-2.229417000	-4.305253000
H	1.423317000	-3.827388000	-3.579464000
H	2.964431000	-2.978627000	-3.464878000
C	2.905995000	1.735392000	1.069846000
C	1.825730000	1.607834000	2.158761000
H	2.144933000	2.217554000	3.013002000
H	1.712403000	0.591449000	2.534230000
H	0.850950000	1.985902000	1.844077000
C	3.079932000	3.255649000	0.838688000
H	3.221586000	3.749856000	1.805621000
H	2.192899000	3.692260000	0.374086000
H	3.946726000	3.509631000	0.224662000
C	4.217980000	1.190301000	1.682508000
H	4.431428000	1.705187000	2.626545000
H	5.072832000	1.337131000	1.015350000
H	4.140706000	0.118836000	1.894758000
H	-5.845573000	-2.358173000	-1.193984000
H	4.443237000	1.075323000	-3.233122000

Compound 1^{Xyl*} (S_0→TICT-S_1 geometry, 100°)

Total energy (ωB97X-D/6-31G(d)): -1685.44049665

P	0.353903000	0.009345000	0.275863000
B	-1.537061000	0.459508000	0.059135000
C	-2.039450000	1.920605000	-0.092186000
C	-1.285294000	3.079708000	0.185800000
H	-0.262943000	2.975478000	0.524014000
C	-1.807171000	4.358439000	0.048166000
H	-1.185467000	5.220319000	0.276157000
C	-3.118480000	4.536386000	-0.385310000
H	-3.526414000	5.535016000	-0.511874000
C	-3.906032000	3.414141000	-0.637871000
H	-4.939850000	3.535106000	-0.950022000
C	-3.385822000	2.138917000	-0.469909000
H	-4.036030000	1.284773000	-0.628432000
C	-2.561024000	-0.770905000	0.044968000
C	-3.201359000	-1.168900000	-1.155398000
C	-4.070314000	-2.262726000	-1.171624000
H	-4.535949000	-2.557585000	-2.109803000
C	-4.348755000	-2.964766000	-0.009854000
C	-3.772303000	-2.552454000	1.183836000
H	-4.012688000	-3.074626000	2.106949000
C	-2.890107000	-1.472467000	1.227698000
C	-2.341446000	-1.074399000	2.574844000
H	-2.972745000	-1.465153000	3.379393000
H	-2.299495000	0.013497000	2.682596000
H	-1.330920000	-1.462655000	2.733612000
C	-3.014347000	-0.436926000	-2.468939000
H	-2.726580000	-1.129749000	-3.267296000
H	-2.255066000	0.346078000	-2.412884000
H	-3.952804000	0.038743000	-2.777326000
C	1.061513000	-0.364448000	1.903308000
C	0.540839000	0.262673000	3.046487000
H	-0.265846000	0.983153000	2.938954000
C	1.044012000	-0.024082000	4.307398000
H	0.628611000	0.474785000	5.177849000
C	2.077490000	-0.947518000	4.453444000
H	2.465555000	-1.182293000	5.439871000
C	2.615508000	-1.558790000	3.324962000
H	3.431155000	-2.268577000	3.427312000
C	2.127412000	-1.260885000	2.057015000
H	2.581781000	-1.723775000	1.188125000

C	1.832661000	-0.172960000	-0.835468000
C	2.108392000	-1.465752000	-1.377526000
C	3.410292000	-1.745408000	-1.796845000
H	3.663707000	-2.726565000	-2.175539000
C	4.406304000	-0.788125000	-1.728854000
C	4.093222000	0.498628000	-1.332891000
H	4.878205000	1.241413000	-1.362890000
C	2.811164000	0.859807000	-0.905492000
C	1.025205000	-2.556028000	-1.592836000
C	-0.130306000	-1.931039000	-2.388320000
H	-0.549497000	-1.054357000	-1.892342000
H	-0.945770000	-2.652493000	-2.507909000
H	0.211118000	-1.614747000	-3.380261000
C	0.503522000	-3.184161000	-0.284868000
H	-0.032556000	-2.477402000	0.352727000
H	1.324108000	-3.618657000	0.295994000
H	-0.204575000	-3.984256000	-0.526799000
C	1.550282000	-3.727743000	-2.445135000
H	1.960563000	-3.390508000	-3.402775000
H	0.715505000	-4.401816000	-2.663178000
H	2.312283000	-4.316713000	-1.923024000
C	2.542215000	2.362823000	-0.655252000
C	1.386536000	2.792899000	-1.581913000
H	1.120779000	3.840985000	-1.405565000
H	0.484936000	2.193381000	-1.451492000
H	1.700979000	2.692066000	-2.626806000
C	3.741348000	3.247837000	-1.054767000
H	3.446968000	4.296688000	-0.943854000
H	4.037493000	3.100163000	-2.098395000
H	4.614765000	3.088514000	-0.413044000
C	2.284530000	2.680967000	0.829299000
H	2.131996000	3.759134000	0.957438000
H	3.147300000	2.381961000	1.434706000
H	1.416410000	2.171027000	1.241961000
H	-5.024979000	-3.814974000	-0.027866000
H	5.421304000	-1.033177000	-2.027699000

3. References

[1] A. Schulz, M. Thomas, A. Villinger, *Dalton Trans* **2018**, *48*, 125-132.

[2] P. Gupta, J. E. Siewert, T. Wellnitz, M. Fischer, W. Baumann, T. Beweries, C. Hering-Junghans, *Dalton Trans.* **2021**, *50*, 1838-1844.

[3] C.-H. Zhao, A. Wakamiya, S. Yamaguchi, *Macromolecules* **2007**, *40*, 3898-3900.

[4] G. M. Sheldrick, *Acta Crystallogr. A* **2015**, *71*, 3-8.

[5] G. M. Sheldrick, *Acta Crystallogr. C* **2015**, *71*, 3-8.

[6] C. B. Hübschle, G. M. Sheldrick, B. Dittrich, *J. Appl. Crystallogr.* **2011**, *44*, 1281-1284.

[7] O. V. Dolomanov, L. J. Bourhis, R. J. Gildea, J. A. K. Howard, H. Puschmann, *J. Appl. Cryst.* **2009**, *42*, 339-341.

[8] A. Saha, S. S. Nia, J. A. Rodríguez, *Chem. Rev.* **2022**, *122*, 13883-13914.

[9] S. Ito, F. J. White, E. Okunishi, Y. Aoyama, A. Yamano, H. Sato, J. D. Ferrara, M. Jasnowski, M. Meyer, *CrystEngComm* **2021**, *23*, 8622-8630.

[10] K.-N. Truong, S. Ito, J. M. Wojciechowski, C. R. Göb, C. J. Schürmann, A. Yamano, M. Del Campo, E. Okunishi, Y. Aoyama, T. Mihira, N. Hosogi, J. Benet-Buchholz, E. C. Escuerdo-Alan, F. J. White, J. D. Ferrara, R. Bücker, *Symmetry* **2023**, *15*, 1555.

[11] M. J. Frisch, G. W. Trucks, H. B. Schlegel, G. E. Scuseria, M. A. Robb, J. R. Cheeseman, G. Scalmani, V. Barone, G. A. Petersson, H. Nakatsuji, X. Li, M. Caricato, A. V. Marenich, J. Bloino, B. G. Janesko, R. Gomperts, B. Mennucci, H. P. Hratchian, J. V. Ortiz, A. F. Izmaylov, J. L. Sonnenberg, D. Williams-Young, F. Ding, F. Lipparini, F. Egidi, J. Goings, B. Peng, A. Petrone, T. Henderson, D. Ranasinghe, V. G. Zakrzewski, J. Gao, N. Rega, G. Zheng, W. Liang, M. Hada, M. Ehara, K. Toyota, R. Fukuda, J. Hasegawa, M. Ishida, T. Nakajima, Y. Honda, O. Kitao, H. Nakai, T. Vreven, K. Throssell, J. A. Montgomery, Jr., J. E. Peralta, F. Ogliaro, M. J. , J. J. Heyd, E. N. Brothers, K. N. Kudin, V. N. Staroverov, T. A. Keith, R. Kobayashi, J. Normand, K. Raghavachari, A. P. Rendell, J. C. Burant, S. S. Iyengar, J. Tomasi, M. Cossi, J. M. Millam, M. Klene, C. Adamo, R. Cammi, J. W. Ochterski, R. L. Martin, K. Morokuma, O. Farkas, J. B. Foresman, D. J. Fox, Gaussian 16 Revision C.01. *Gaussian, Inc.*, Wallingford CT, 2016.

[12] J.-D. Chai, M. Head-Gordon, *Phys. Chem. Chem. Phys.* **2008**, *10*, 6615–6620.

[13] a) J. D. Dill, J. A. Pople, *J. Chem. Phys.* **1975**, *62*, 2921-2923; b) R. Ditchfield, W. J. Hehre, J. A. Pople, *J. Chem. Phys.* **1971**, *54*, 724-728; c) M. M. Francl, W. J. Pietro, W. J. Hehre, J. S. Binkley, M. S. Gordon, D. J. DeFrees, J. A. Pople, *J. Chem. Phys.* **1982**, *77*, 3654-3665; d) M. J. Frisch, G. W. Trucks, H. B. Schlegel, G. E. Scuseria, M. A. Robb, J. R. Cheeseman, G. Scalmani, V. Barone, G. A. Petersson, H. Nakatsuji, X. Li, M. Caricato, A. V. Marenich, J. Bloino, B. G. Janesko, R. Gomperts, B. Mennucci, H. P. Hratchian, J. V. Ortiz, A. F. Izmaylov, J. L. Sonnenberg, D. Williams-Young, F. Ding, F. Lipparini, F. Egidi, J. Goings, B. Peng, A. Petrone, T. Henderson, D. Ranasinghe, V. G. Zakrzewski, J. Gao, N. Rega, G. Zheng, W. Liang, M. Hada, M. Ehara, K. Toyota, R. Fukuda, J. Hasegawa, M. Ishida, T. Nakajima, Y. Honda, O. Kitao, H. Nakai, T. Vreven, K. Throssell, J. A. Montgomery, J. E. Peralta, F. Ogliaro, M. J. Bearpark, J. J. Heyd, E. N. Brothers, K. N. Kudin, V. N. Staroverov, T. A. Keith, R. Kobayashi, J. Normand, K. Raghavachari, A. P. Rendell, J. C. Burant, S. S. Iyengar, J. Tomasi, M. Cossi, J. M. Millam, M. Klene, C. Adamo, R. Cammi, J. W. Ochterski, R. L. Martin, K. Morokuma, O. Farkas, J. B. Foresman, Fox, D. J. Gaussian 09 Revision E.01. *Gaussian, Inc.*, Wallingford CT, 2009; e) M. S. Gordon, J. S. Binkley, J. A. Pople, W. J. Pietro, W. J. Hehre, *J. Am. Chem. Soc.* **1982**, *104*, 2797-2803; f) P. C. Hariharan, J. A. Pople, *Theor. Chim. Acta* **1973**, *28*, 213-222; g) W. J. Hehre, R. Ditchfield, J. A. Pople, *J. Chem. Phys.* **1972**, *56*, 2257-2261.

[14] a) F. Neese, F. Wennmohs, U. Becker, C. Riplinger, *J. Chem. Phys.* **2020**, *152*, 224108; b) F. Neese, *Comput. Mol. Sci.* **2022**, *12*, e1606.

[15] Y. S. Lin, G. D. Li, S. P. Mao, J. D. Chai, *J. Chem. Theory Comput.* **2013**, *9*, 263-272.

[16] a) T. Stein, L. Kronik, R. Baer, *J. Am. Chem. Soc.* **2009**, *131*, 2818-2820; b) T. Stein, H. Eisenberg, L. Kronik, R. Baer, *Phys. Rev. Lett.* **2010**, *105*, 266802; c) A. Karolewski, L. Kronik, S. Kummel, *J. Chem. Phys.* **2013**, *138*, 204115.

[17] F. Weigend, R. Ahlrichs, *Phys. Chem. Chem. Phys.* **2005**, *7*, 3297-3305.

[18] V. Barone, M. Cossi, *J. Phys. Chem. A* **1998**, *102*, 1995-2001.

[19] K. A. Wiberg, *Tetrahedron* **1968**, *24*, 1083.

[20] a) I. Mayer, *Chem. Phys. Lett.* **1983**, *97*, 270; b) I. Mayer, *Int. J. Quantum Chem.* **1984**, *26*, 151-154.

[21] a) T. Lu, F. Chen, *J. Comput. Chem.* **2012**, *33*, 580-592; b) T. Lu, *J. Chem. Phys.* **2024**, *161*, 082503.
